# Mitochondrial Genomes Assembled from Non-Invasive eDNA Metagenomic Scat Samples in Critically Endangered Mammals

**DOI:** 10.3390/genes14030657

**Published:** 2023-03-05

**Authors:** J. Antonio Baeza, Ryan Barata, Dilani Rajapakse, Jayra Penaloza, Preston Harrison, Adam Haberski

**Affiliations:** 1 Department of Biological Sciences, 132 Long Hall, Clemson University, Clemson, SC 29634, USA; 2 Smithsonian Marine Station at Fort Pierce, 701 Seaway Drive, Fort Pierce, FL 34949, USA; 3 Department de Biología Marina, Facultad de Ciencias del Mar, Universidad Católica del Norte, Larrondo 1281, Coquimbo, Chile; 4 Department of Plant and Environmental Sciences, 277 Poole Agricultural Center, Clemson University, Clemson, SC 29634, USA

**Keywords:** mitochondrial genome, eDNA, iDNA, metagenomics

## Abstract

The abundance of many large-bodied vertebrates, both in marine and terrestrial environments, has declined substantially due to global and regional climate stressors that define the Anthropocene. The development of genetic tools that can serve to monitor population’s health non-intrusively and inform strategies for the recovery of these species is crucial. In this study, we formally evaluate whether whole mitochondrial genomes can be assembled from environmental DNA (eDNA) metagenomics scat samples. Mitogenomes of four different large vertebrates, the panda bear (*Ailuropoda melanoleuca*), the moon bear (*Ursus thibetanus*), the Java pangolin (*Manis javanica*), and the the North Atlantic right whale (*Eubalaena glacialis*) were assembled and circularized using the pipeline GetOrganelle with a coverage ranging from 12x to 480x in 14 out of 18 different eDNA samples. Partial mitochondrial genomes were retrieved from three other eDNA samples. The complete mitochondrial genomes of the studied species were AT-rich and comprised 13 protein coding genes, 22 transfer RNA genes, two ribosomal RNA genes, and a putative D-loop/control region. Synteny observed in all assembled mitogenomes was identical to that reported for specimens of the same and other closely related species. This study demonstrates that it is possible to assemble accurate whole mitochondrial chromosomes from eDNA samples (scats) using forthright bench and bioinformatics workflows. The retrieval of mitochondrial genomes from eDNA samples represents a tool to support bioprospecting, bio-monitoring, and other non-intrusive conservation strategies in species considered ‘vulnerable’, ‘endangered’, and/or ‘critically endangered’ by the IUCN Red List of Threatened Species.

## 1. Introduction

The abundance of many vertebrates, most notably large-bodied species in terrestrial and marine environments, has been substantially impacted by both global and regional climate stressors that define the Anthropocene [1,2]. One of the most important but difficult aims in conservation and wildlife management is to reinstate the population abundance of these large vertebrates whose populations are, for the most part, declining due to human-related activities [3,4,5]. The fact that many large vertebrates, particularly those that have become vulnerable, endangered, or otherwise in danger of extinction, have grown elusive in recent decades is also worrisome [6], as it is likely to impact conservation efforts. Traditional ‘invasive’ bioprospecting and bio-monitoring strategies may need to be avoided when studying these imperiled species; invasive sampling can disturb and/or stress individuals and populations that are already facing major anthropogenic impact [6].

Environmental DNA (eDNA) has emerged as a sound alternative to intrusive methods for the bioprospecting and biomonitoring of endangered species. The term “eDNA” refers to the genetic material found in environmental samples including, but not limited to, water, air, soil, sediment, saliva, skin, blood, and feces [7]. eDNA has proved useful in situations in which invasive sampling of elusive species is logistically difficult and/or ethically fraught [8,9], including the estimation of their abundance, genetic diversity, and population structure [10,11]. Most recently, eDNA extracted from scats, in particular, has been employed successfully for bioprospecting and biomonitoring [12,13,14]. Still, the difficulty of isolating high quality (i.e., high molecular weight) genomic DNA from scats has, until recently, constrained the sequencing and assembly of relatively long sequences such as bacterial or mitochondrial genomes directly from scats. Nonetheless, thanks to advances in DNA extraction protocols and bioinformatic tools, various recent studies have shown that mitochondrial, as well as full or almost complete bacterial genomes, can be reconstructed from scat eDNA [15,16,17]. In this study, we explore if complete or nearly complete mitochondrial genomes can be retrieved from eDNA metagenomic scat samples using, as an example, various iconic species that are currently experiencing considerable anthropogenically driven impacts: the giant panda *Ailuropoda melanoleuca*, the pangolin *Manis javanica*, the moon bear *Ursus thibetanus*, and the North Atlantic right whale *Eubalaena glacialis*.

The panda bear or giant panda (*A. melanoleuca*) is a folivore that lives in dense bamboo forests endemic to central Asia (China) and whose distribution shrunk considerably during the last decades due to a combination of farming, deforestation, poaching, and urban expansion, among various others [18,19]. Throughout the years, estimates of population abundance have varied considerably, but there is consensus regarding steady increases in its abundance in the wild during the last decade. Thus, in 2016, the Red list of the International Union for Conservation of Nature (IUCN) reclassified the panda bear from “endangered” to “vulnerable” [20]. In turn, the Asian black bear, moon bear, or white-chested bear (*U. thibetanus*) is an arboreal omnivore mostly restricted to southeastern continental Asia. The moon bear is currently threatened by deforestation and poaching [21] that, together with other human-mediated impacts, have resulted in a patchy distribution in its native range. Furthermore, due to recent findings indicating a decreasing population trend [22], the IUCN Red list has classified the Asiatic black bear as “vulnerable” [23]. Another mammal of smaller body size, the Sunda, Malayan, or Javan pangolin (*M. javanica*) is a myrmecophagous scale-covered species (fam. Pholidota) found throughout continental southeast Asia as well as in the islands of Borneo, Java, Sumatra, and the Lesser Sunda Islands [24]. The IUCN Red list has classified this species as “critically endangered” given its dwindling numbers in the wild, driven by heavy poaching for its skin, scales, and meat that are used for clothing and traditional medicine [25]. Indeed, *M. javanica* and other species of pangolins are threatened at such a level that they are considered the most heavily trafficked wild mammals on earth [26]. Lastly, the North Atlantic right whale (*E. glacialis*), restricted to the eastern coast of the USA and Canada [27], is one of three right whale species that have been historically targeted by a lucrative whaling industry. *Eubalaena glacialis* is among the most endangered marine mammals in the world [28], with no more than 400 individuals surviving in the wild; mortality from collisions with vessels and entanglement in commercial fishing gear are currently threatening its survival and potential recovery [29,30].

The number of genomic resources to support conservation efforts for all aforementioned species has increased steadily during the last decade [31,32,33]. Still, the development of genetic tools that can serve to monitor the population’s health non-invasively and inform strategies for the recovery of these and other renowned endangered species is crucial.

In this study, we formally evaluate whether whole mitochondrial genomes can be assembled from eDNA metagenomic scat samples. If effective, this type of genomic resource can be used to study population genetics in iconic endangered species such as pandas and moon bears, pangolins, and whales, as well as to conduct biomonitoring and bioprospecting by utilizing reasonably priced molecular markers and straightforward bioinformatics workflows.

## 2. Materials and Methods

### 2.1. Sequencing of Mitochondrial Genomes

The raw sequence datasets used to assemble the mitochondrial genome of the different studied species from eDNA were generated by [31,32,33] and were employed by these authors to describe the gut microbiome. A total of 18 different sets of sequences were retrieved from GenBank (see SRA Accession numbers in Table 1) and detailed information on sampling, DNA extraction, and sequencing methods can be found in the aforementioned studies. The amount of data available in FASTQ format varied between 7,013,164 (ERR4056911) and 24,216,055 (SRR7524043) PE reads per sample (Table 1). The totality of the available reads generated for each fecal sample was used for mitochondrial genome assembly.

### 2.2. Mitochondrial Genome Assembly

Assemblage of the mitochondrial genome from scat samples belonging to the different studied species was attempted using the target-restricted-assembly software GetOrganelle v1.2.3 [34]. GetOrganelle uses a seed-and-extend algorithm that assembles organelle and mitochondrial genomes from whole genome sequencing (WGS), including metagenomics and datasets starting from a related or distant single ‘seed’ sequence [34]. Different previously published mitochondrial genomes belonging to *U. thibetanus* (KT964290), *A. melanoleuca* (NC009492), *M. javanica* (MN365836), and *E. glacialis* (MF459656) retrieved from GenBank were used as the ‘seed’ (=reference) to assemble the mitochondrial genomes from scat samples of the same species. All the different runs used k-mer sizes of 21, 55, 85, and 115. Reads were not quality trimmed prior to assembly in GetOrganelle, following the developer’s suggestions [34]. The program Bandage [35] was employed to visualize the assembly graph generated by GetOrganelle, and assembled contigs (if any) were compared to the nucleotide non-redundant database in NCBI’s GenBank in order to determine if these contigs belonged to the mitochondrial genome of the target species. We predicted that a circularized (or linear) sequence of ~17–19 kpb in length, depending upon the studied species, would be observed among the contigs if the pipeline above successfully assembled (either completely or partially) the mitochondrial genome of the target species.

### 2.3. Mitochondrial Genome Annotation and Analysis 

Mitochondrial genomes assembled from scat samples were first annotated in silico using the web servers MITOS (http://mitos.bioinf.uni-leipzig.de (accessed on 28 August 2022)—[36]) and MITOS2 (http://mitos2.bioinf.uni-leipzig.de (accessed on 28 August 2022)—[37]) with the vertebrate genetic code (code 2). Next, manual curation of the in silico annotations, including start and stop codon corrections, were conducted while visualizing the mitochondrial genomes with the software MEGA7 [38] and translating specific regions with the ExPASy translate tool in the web server ExPASy (https://web.expasy.org/ (accessed on 28 August 2022) [39]). Visualization of the assembled mitochondrial genomes was conducted with GenomeVx (http://wolfe.ucd.ie/GenomeVx/ (accessed on 28 August 2022)) [40].

The nucleotide composition of the entire mitochondrial genome and codon usage profiles of the protein coding genes (PCGs) were analyzed; MEGA X was used to estimate nucleotide composition, while codon usage and relative synonymous codon usage (RSCU) were predicted using the invertebrate mitochondrial code in the web server Codon Usage (http://www.bioinformatics.org/sms2/codon_usage.html (accessed on 28 August 2022)).

tRNA genes were identified and their secondary structures were predicted with the program MiTFi [41], as implemented in the MITOS2 web server. The secondary structure of each tRNA was then visualized in the web server, Forna (http://rna.tbi.univie.ac.at/forna/ (accessed on 28 August 2022)) [42]. 

The RNAfold structure web server (http://rna.tbi.univie.ac.at//cgi-bin/RNAWebSuite/RNAfold.cgi (accessed on 28 August 2022)) [43] was used to predict the lowest free energy secondary structure of the putative D-loop/control region (CR), with particular attention placed on the presence of ‘stem and loops’ in each species. The Tandem Repeats Finder webserver (https://tandem.bu.edu/trf/trf.basic.submit.html (accessed on 28 August 2022)) [44] was utilized to ascertain the location of tandem repeats within the control region. Moreover, microsatellites were found using the microsatellite repeats finder web server (http://insilico.ehu.es/mini_tools/microsatellites/ (accessed on 28 August 2022)). The three functional domains of the CR found in mammals [45] were also detected in the CR of each studied species after a multiple alignment of this region with that of closely related species. Lastly, the secondary structure of tandem repeats (if any) located in the CSB domain was predicted and visualized using the FORNA web server [42].

## 3. Results and Discussion

The software GetOrganelle assembled and circularized the mitochondrial genomes in 14 out of the 18 tested eDNA metagenomics scat samples (Table 1). Partial mitochondrial genomes were retrieved from three other eDNA samples, while no mitochondrial contigs were retrieved from a single sample (Table 1). No obvious association was observed between the total number of raw reads comprising a metagenomic dataset and success (or not) in assembling a mitochondrial genome. For instance, GetOrganelle only assembled a partial mitochondrial genome (4290 bp, 717x) using a relatively large set of PE reads (20,539,115 PE reads from *U. thibetanus* SRR6109213), while the same software assembled a complete mitochondrial genome only using 8,678,339 PE reads from a different sample belonging to the same species (16,725 bp, 79x, sample SRR6109214) (Table 1). Additionally, considering only samples from which complete mitochondrial genomes were assembled, a moderate statistically significant relationship between the number of raw reads used for the assembly and coverage of the assembled complete mitochondrial genome was observed (regression analysis: r^2^ = 0.3705, d.f. = 1,12, *p* = 0.0206).

**Figure 1:**
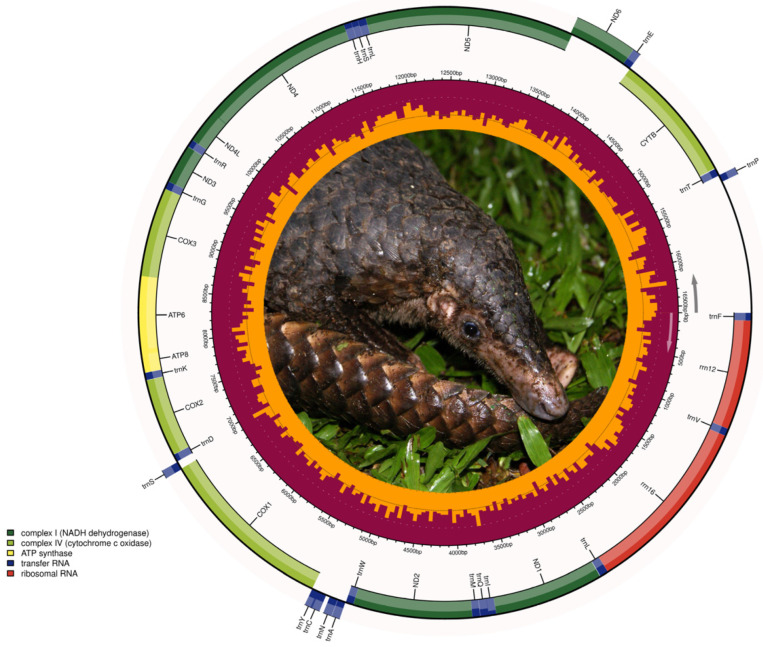
Circular DNA mitochondrial genome map of the Java pangolin (*Manis javanica*) assembled from eDNA scats. The annotated map depicts 13 protein-coding genes (PCGs), two ribosomal RNA genes (rrnS: 12 S ribosomal RNA and rrnL: 16 S ribosomal RNA), 22 transfer RNA (tRNA) genes, and the putative control region (not annotated). Mitochondrial genome structure was most similar among the different species analyzed. Photo credit: Firman/INaturalist, CC BY. https://www.inaturalist.org/guide_taxa/178736 (accessed on 28 August 2022).

Considering only assemblies reported as complete and circular by GetOrganelle, the mitochondrial genomes had a coverage that varied between 12x in *E. glacialis* and 480x in *M. javanica,* and varied in length between 16,387 bp in *E. glacialis* and 16,852 bp in *A. melanoleuca* (Table 1, Supplementary Materials Tables S1–S14). These mitochondrial genomes contained few intergenic spaces and overlaps between gene junctions. All of the mitochondrial genomes from the examined species contained 13 PCGs, two ribosomal RNA genes (rrnS [12S ribosomal RNA] and rrnL [16S ribosomal RNA]), and 22 tRNA genes (Figure 1). A single long intergenic space was assumed to be the CR in all the examined species. The CRs ranged from 564 bp (*M. javanica*) to 1452 bp (*A. melanoleuca*) in length.

Mitochondrial synteny in *U. thibetanus* and *A. melanoleuca* was identical to each other and to all other extant species of bears in the genus *Ursus,* whose mitochondrial genomes have been assembled and annotated and are available in GenBank [46]. In turn, the pangolin *M. javanica* also displayed identical mitochondrial synteny to other congeneric and confamilial species, including *M. pentadactyla*, *Smutsia temmnickii*, *S. gigantea*, *Phataginus tricuspis*, and *P. tetradactyla* [47,48]. Likewise, *E. glacialis* exhibited identical mitochondrial synteny to that of the congenerics southern right whale (*E. australis*) and North Pacific right whale (*E. japonica*) [49,50]. Additionally, *E. glacialis* exhibited identical mitochondrial gene synteny to the bowhead whale (*Balaena mysticetus*), a related species of baleen whale [51] (Figure 1).

**Figure 2:**
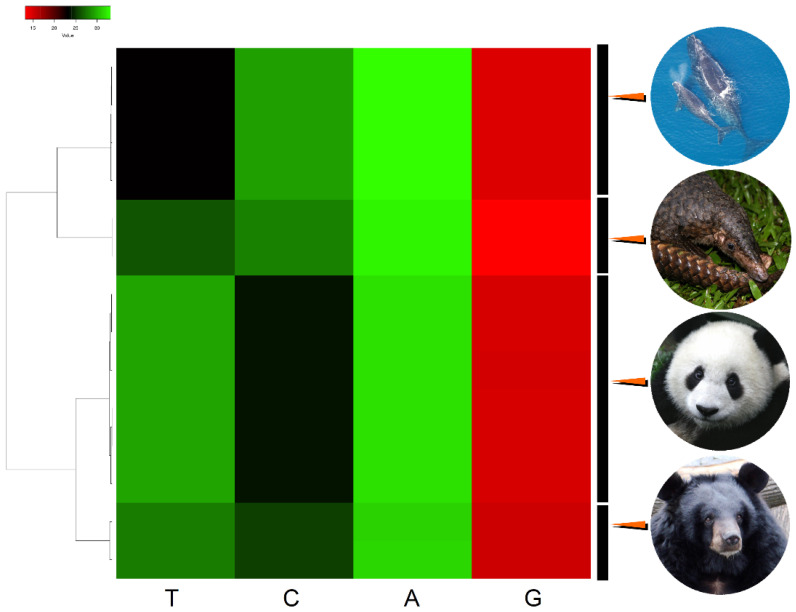
Heatmap of hierarchically clustered data showing nucleotide use in mitochondrial genomes of the panda bear (*Ailuropoda melanoleuca*; *n* = 6 specimens), the moon bear (*Ursus thibetanus*; *n* = 2), the Java pangolin (*Manis javanica*; *n* = 4), and the the North Atlantic right whale (*Eubalaena glacialis*; *n* = 2). Photo credits: Pangolin, Firman/INaturalist, CC BY. https://www.inaturalist.org/guide_taxa/178736 (accessed on 28 August 2022). Moon bear, Guérin Nicolas/CC BY 3.0. https://en.wikipedia.org/wiki/File:Ursus_thibetanus_3_(Wroclaw_zoo).JPG. (accessed on 28 August 2022) Panda bear, Sheila Lau/CC BY 3.0. https://commons.wikimedia.org/wiki/File:Panda_Cub_from_Wolong,_Sichuan,_China.JPG. (accessed on 28 August 2022) North Atlantic right whale, NOAA/CC BY 3.0. https://upload.wikimedia.org/wikipedia/commons/6/6a/Eubalaena_glacialis_with_calf.jpg (accessed on 28 August 2022).

All studied mitochondrial genomes were AT-rich. Their nucleotide usage (i.e., composition) ranges were A = 30.53–32.36%, T = 26.64–28.94%, C = 24.89–26.01%, and G = 13.85–17.29% (*U. thibetanus*), A = 29.93–33.67%, T = 28.22–31.34%, C = 23.06–24.33%, and G = 14.71–15.10% (*A. melanoleuca*), A = 33.01–33.21%, T = 22.30–24.51%, C = 27.99–30.15%, and G = 13.07–16.38% (*M. javanica*), and A = 32.18–33.21%, T = 25.08–26.79%, C = 27.43–28.79%, and G = 12.50–13.95% (*E. glacialis*). These AT-contents all fall within known ranges reported for closely related species. For example, *E. glacialis* has an AT-content between 57.26% and 60.00%, while *E. japonica* and *E. australis*, have AT-contents of 58.88% and 58.78%, respectively [50]. Of the studied species, *A. melanoleuca* has the richest AT mitochondrial genome, while *M. javanica* has the least AT-rich genome. The mitochondrial genomes retrieved from the different samples segregated according to phylogenetic relatedness in a cluster analysis based on nucleotide use. As expected, *U. thibetanus* and *A. melanoleuca* are most similar to each other compared to the other studied species in terms of nucleotide usage (Figure 2).

In all but one of the studied species, all 13 PCGs exhibited conventional vertebrate and mammalian start codons (ATA, ATG, ATC, ATT). The exception was *E. glacialis*, which exhibited a GTG start codon in *nad4l*. This unconventional start codon was previously identified in the fin whale (*Balaenoptera physalus*) and the blue whale (*Balaenoptera musculus*) [52], as well as in more distantly related species (i.e., in the Ganges River dolphin *Platanista gangetica* [53]). The start codons observed in *A. melanoleuca* agree with previous studies [54]. 

Most of the PCGs in the species examined ended with complete and conventional stop codons (TAA, TAG, or AGA). The few exceptions included *nad3*, which ended with an incomplete stop codon, TA, in all studied species, and nad4, which ended with an incomplete stop codon, T, in all studied species. Another gene, *cox3*, ended with an incomplete TA stop codon in *M. javanica*, and T in *A. melanoleuca* and *U. thibetanus*. Truncated stop codons are often observed in mammals and other vertebrates and are thought to be completed through post-transcriptional poly-adenylation [55].

**Figure 3:**
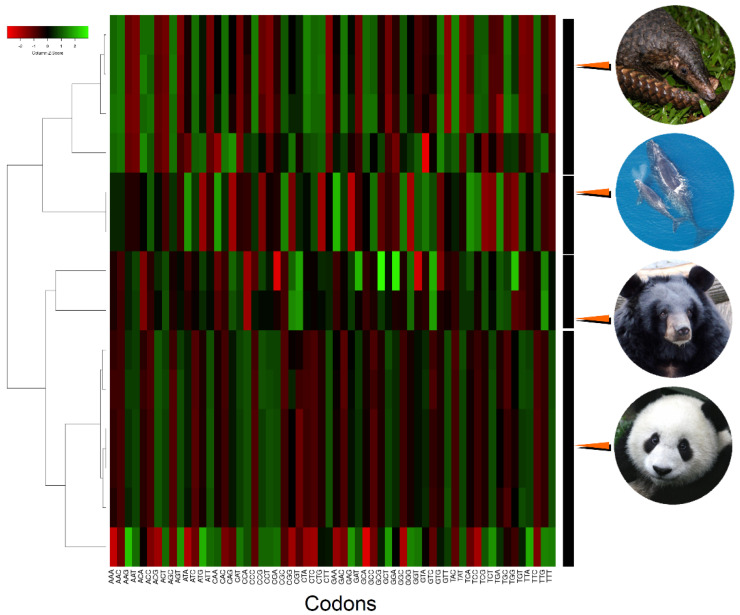
Heatmap of hierarchically clustered data showing relative synonymous codon usage in mitochondrial genomes of the panda bear (*Ailuropoda melanoleuca*; *n* = 6 specimens), the moon bear (*Ursus thibetanus*; *n* = 2), the Java pangolin (*Manis javanica*; *n* = 4), and the the North Atlantic right whale (*Eubalaena glacialis*; *n* = 2). Photo credits: Pangolin, Firman/INaturalist, CC BY. https://www.inaturalist.org/guide_taxa/178736 (accessed on 28 August 2022). Moon bear, Guérin Nicolas/CC BY 3.0. https://en.wikipedia.org/wiki/File:Ursus_thibetanus_3_(Wroclaw_zoo).JPG (accessed on 28 August 2022). panda bear, Sheila Lau /CCBY3.0.https://commons.wikimedia.org/wiki/File:panda_Cub_from_Wolong,_Sichuan,_China.JPG (accessed on 28 August 2022). North Atlantic Right Whale, NOAA/CC BY 3.0. https://upload.wikimedia.org/wikipedia/commons/6/6a/Eubalaena_glacialis_with_calf.jpg (accessed on 28 August 2022).

The most frequently used codon within the PCGs of all studied species was CTA, while the second most frequently used codons varied by species but were all AT rich, and included ATT (in *A. melanoleuca*), ATA (in *U. thibetanus*), and ATC (in *M. javanica* and *E. glacialis*). The codons with the lowest frequencies included CGG, TGT, and CCG, among a few others. Most notably, CGG appeared least frequently in *M. javanica* and *E. glacialis* and was the second-least frequently used codon in *A. melanoleuca* and *U. thibetanus* (Figure 3). The observed disproportionate use of codons (and RSCU) is in line with that observed before in the same [56] and other closely related species of bears, pangolins, and whales [54].

**Figure 4:**
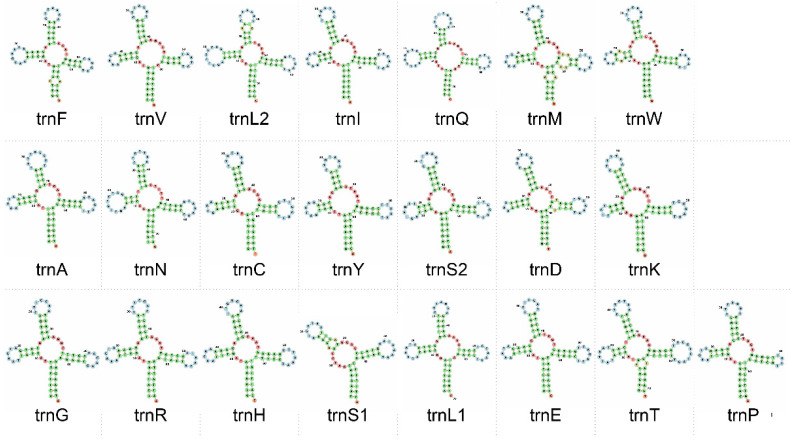
Secondary structure of tRNAs in the mitochondrial genome of the panda bear (*Ailuropoda melanoleuca)*. Out of 22 tRNA genes, 21 exhibit a ‘cloverleaf’ secondary structure. tRNA-Ser1 invariably lacked the entire dihydroxyuridine (DHU) arm (stem + loop). Secondary structures visualized in the Forna web server. The secondary structure of tRNA genes was similar in *U. thibetanus* and *E. glacialis* to that found in *A. melanoleuca*.

**Figure 5:**
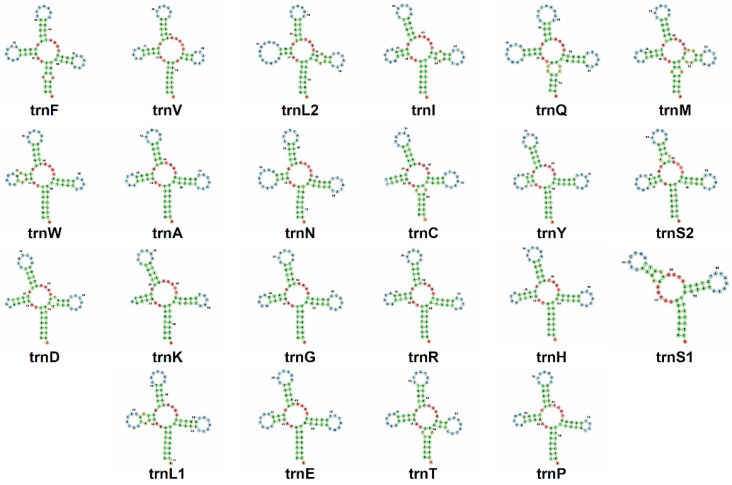
Secondary structure of tRNAs in the mitochondrial genome of the Java pangolin (*Manis javanica)*. Out of 22 tRNA genes, 19 exhibit a ‘cloverleaf’ secondary structure. tRNA-Ser1 invariably lacked the entire dihydroxyuridine (DHU) arm (stem + loop), while tRNA-C and tRNA-K lacked the dihydroxyuridine (DHU) loop but not the stem. Secondary structures visualized in the Forna web server.

In the mitochondrial genome of *A. melanoleuca*, *U. thibetanus*, and *E. glacialis*, 21 out of the 22 tRNA genes exhibit a ‘cloverleaf’ secondary structure (Figure 4). In all these species, tRNA-S1 invariably lacked the entire dihydroxyuridine (DHU) arm (loop + stem) in agreement with previous studies of *A. melanoleuca* [54], three Asiatic black bears [46], and Cuvier’s beaked whale (*Ziphius cavirostris*) [57]. Indeed, truncation of tRNA-Ser1 is characteristic of eumetazoans [36] and such arm deletion in this tRNA gene might impact proper interaction with enzymatic machinery, as both D and T loops are known to form tertiary protein interactions in tRNA [58]. In the pangolin *M. javanica*, 3 out of the 22 tRNAs differed from the canonical cloverleaf shape (arms and/or acceptor stem were either truncated or missing). tRNA-S1 lacked the D arm as observed in the other studied species, and tRNA-C and tRNA-K lacked the D arm loop but not the stem (Figure 5). Previous studies in *M. pentadactyla* found that tRNA-S1 exhibited a loss of the entire DHU arm [47,59]. However, contrary to our results, [19] found that in *M. javanica,* 21 tRNA genes, including tRNA-C and tRNA-K fold into the expected cloverleaf secondary structure. 

In the studied species the rrnS, gene length varied between 961 bp in *M. javanica* and 974 bp in *E. glacialis*. In turn, the rrnL gene length varied between 1571 bp in *M. javanica* and 1580 bp in both *U. thibetanus* and *A. melanoleuca*. These genes were in proximity to the CR, preceded by tRNA-F, but separated by tRNA-V in all species. No major differences in nucleotide usage in the rrnS and rrnL genes were observed in the different studied species. As shown to occur in other mammals [60], the two genes were highly AT-rich. The base composition ranges of the rrnS gene was A = 35.58%, T = 23.16–23.27 %, G = 18.72%, C = 22.44–22.54% (*U. thibetanus*), A = 35.40%, T = 24.43%, G = 18.43%, C = 21.74% (*A. melanoleuca*), A = 35.69%, T = 19.88–20.08%, G = 18.83–18.94%, C = 25.39–25.49% (*M. javanica*), and A = 35.32%, T = 21.46%, G = 18.69%, C = 24.43% (*E. glacialis*).

The base composition ranges of the rrnL gene were A = 36.14–36.20%, T = 23.80%, G = 18.30–18.35%, C = 21.71% (*U. thibetanus*), A = 36.27%, T = 25.44–25.63%, G = 18.42%. C = 19.68–19.87% (*A. melanoleuca*), A = 38.00- 38.19%, T = 21.71%, G = 17.70–17.89%, C = 24.41% (*M. javanica*), and A = 36.66%, T = 24.33%, G = 17.22%, C = 21.79% (*E. glacialis*).

The putative CR, located between tRNA-P and tRNA-F in the mitochondrial genomes of all the studied species, varied in length from 919 bp in *E. glacialis* to 1335 bp in *A. melanoleuca*. The region contained a clear AT-bias, with an overall base composition range of A = 26.22–33.19%, T = 29.21–30.46%, C = 20.81–25.54%, and G = 15.99–19.03%. The CR of *A. melanoleuca, U. thibetanus*, and *M. javanica* contain microsatellites, short tandem repeats, and hairpin secondary structures. The same region in *E. glacialis* contains microsatellites and hairpin secondary structures but no short tandem repeats. The different specimens of *A. melanoleuca, U. thibetanus,* and *E. glacialis* contained between 9 and 11 microsatellites in the CR, and most were CC and TT dinucleotide repeats. In turn, the analyzed specimens of *M. javanica* contained between 6 and 11 microsatellites in the CR, and most were TAT trinucleotide repeats. The presence and number of tandem repeats agree with previous findings in the same and other closely related species [33,56,61]. The RNA-structure web server estimated secondary structures that contained variable numbers and sizes of stem-loops throughout the entire CR sequence of each species. The latter observation is also in line with previous studies in the same and other closely related species [60,62,63].

**Figure 6:**
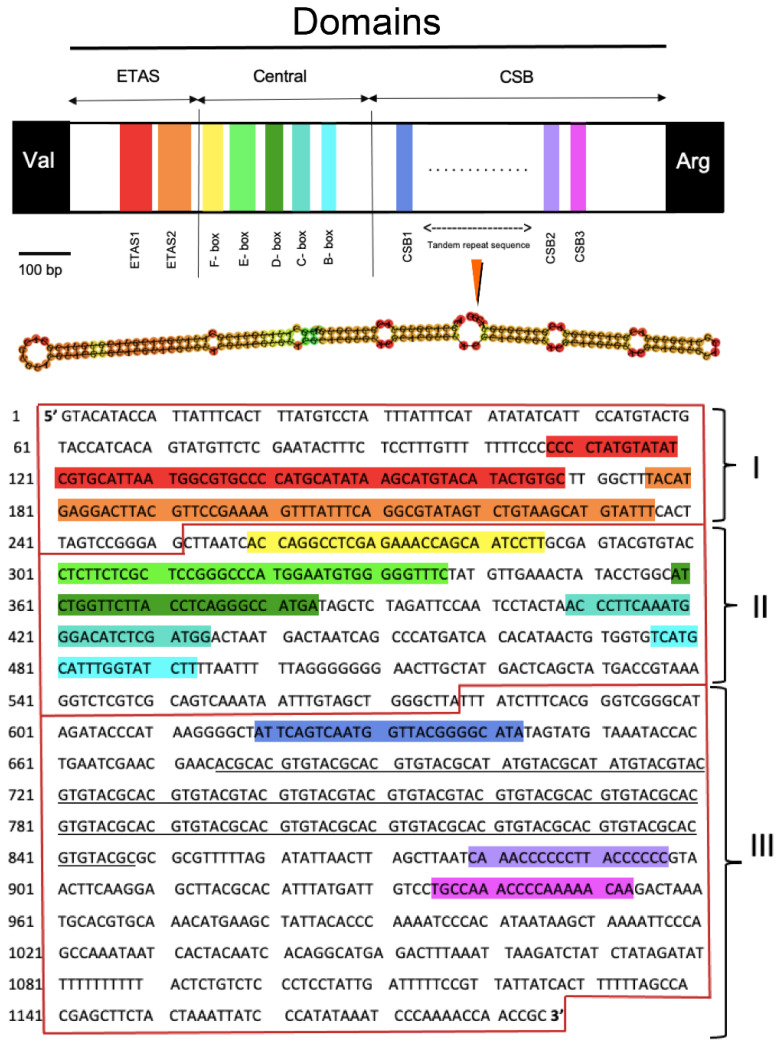
Visual representation of the D-loop/control region (CR) in the mitochondrial genome of the moon bear (*Ursus thibetanus)*. The CR is divided into the extended terminal association sequence (ETAS), central, and conserved sequence block (CSB) domains. Locations of the ETAS 1 and ETAS 2, CSB1, CSB2, CSB3 blocks, as well as the large highly conserved regions within the central domain are shown. The long repetitive motif is underlined, and its secondary structure is depicted immediately below the visual representation of the CR. The CR of *Ailuropoda melanoleuca*, *Manis javanica*, and *Eubalaena glacialis* were similar. Nonetheless, no tandem repeat located after the CSB1 motif was observed in *E. glacialis*. Nucleotides are highlighted with different colors to match features in the graphical representation of the CR.

The extended terminal association sequences, central, and conserved sequence block (CSB) domains which make up the three functional domains of the CR in mammals [45], were also identified in all studied specimens. *Ailuropoda melanoleuca*, *U. thibetanus*, and *M. javanica* contained a relatively long tandem repeat 10 to 78 bp in length located in the CSB domain, between the CSB1 and CSB2 motifs, that folded into a hairpin structure (Figure 6) in accordance with that observed in other mammals [64,65,66]. The organization of the CR has not been examined in detail in whales, including the genus *Eubalaena* [45,64]. The absence of tandem repeats that form hairpin secondary structures in the CSB domain of *E. glacialis* is inconsistent, nonetheless, with previous studies that have detected tandem repeats in other species of cetaceans, including the Ganges River dolphin *Platanista gangetica* [53].

## 4. Conclusions

Using metagenomic libraries and a straightforward bioinformatics workflow, we have assembled for the first time the mitochondrial genomes of various vulnerable and critically endangered large-bodied vertebrates from eDNA (from scats) metagenomic datasets. The annotation and thorough description provided above show that the mitochondrial genome assemblies recovered from the eDNA are accurate. This strategy to assemble mitochondrial genomes represents a tool in conservation biology for these endangered species. With additional research, eDNA from scats may aid in bioprospecting, biomonitoring, and ultimately in helping us to understand population structures and the genomic underpinnings of adaptability to the anthropogenic influences and climatic changes that this iconic and endangered, large-bodied and long-living vertebrates are and will be exposed to.

## Figures and Tables

**Table 1:** Metagenomic datasets employed to assemble mitochondrial genomes. * = contigs total length in partially assembled mitochondrial genomes. A = assembly number in Supplementary Materials.

Species	NCBI SRA	Read N	Assembly	Coverage	Length (bp)	A
*Ursus thibetanus*	SRR6109212	10,906,235	**Circular**	122x	16,634	1
*Ursus thibetanus*	SRR6109213	20,539,115	Partial (1)	717x	4.290 *	–
*Ursus thibetanus*	SRR6109214	8,678,339	**Circular**	79x	16,725	2
*Ursus thibetanus*	SRR6109215	8,052,349	None	–	–	–
*Ailuropoda melanoleuca*	SRR6109207	12,404,708	**Circular**	40x	16,615	3
*Ailuropoda melanoleuca*	SRR6109208	11,477,287	**Circular**	246x	16,786	4
*Ailuropoda melanoleuca*	SRR6109209	11,245,869	**Circular**	34x	16,677	5
*Ailuropoda melanoleuca*	SRR6109210	11,944,071	**Circular**	224x	16,852	6
*Ailuropoda melanoleuca*	SRR6109211	12,489,755	**Circular**	56x	16,617	7
*Ailuropoda melanoleuca*	SRR6109216	12,611,226	**Circular**	127x	16,767	8
*Manis javanica*	SRR7477311	23,833,983	**Circular**	75x	16,576	9
*Manis javanica*	SRR7498027	23,619,748	**Circular**	183x	16,576	10
*Manis javanica*	SRR7507293	23,879,794	**Circular**	480x	16,576	11
*Manis javanica*	SRR7524043	24,216,055	**Circular**	294x	16,576	12
*Eubalena glacialis*	ERR4056911	7,013,164	Partial (7)	6x	15,573 *	–
*Eubalena glacialis*	ERR4056912	9,384,989	**Circular**	43x	16,387	13
*Eubalena glacialis*	ERR4056913	10,423,814	Partial (4)	11x	16,291 *	–
*Eubalena glacialis*	ERR4056914	7,194,209	**Circular**	12x	16,387	14

## Data Availability

The totality of the available reads generated from each fecal sample was used for mitochondrial genome assembly, and these are available in the short-read archive (SRA) repository at GenBank. See Table 1 for details.

## Supplementary Materials

The following supporting information can be downloaded at: https://www.mdpi.com/article/10.3390/genes14030657/s1, Suppl. Mat. Table S1–S14. Mitochondrial Genome Annotations and assemblies.
