# Supplementary material for: Mitochondrial Genomes Assembled from Non-Invasive eDNA Metagenomic Scat Samples in Critically Endangered Mammals
Source: Genes (Basel). 2023 Mar 5;14(3):657. doi: 10.3390/genes14030657 (PMC10048355; doi:10.3390/genes14030657)
Supplement: Supplementary file 1 [file genes-14-00657-s001.zip › genes-2242558-supplementary.pdf]

Supplementary Materials

Table S1. Mitochondrial genome of *Ursus thibetanus* assembled from scat sample SRR6109212. Features and annotation.

Name	Type	Start	Stop	Strand	Length (bp)	Start	Stop	Anticodon	Continuity
C. Region1		1	248	+	248				
OH		249	577	+	329				0
C.Region 2		578	1185	+	608				0
trnF(gaa)	tRNA	1186	1253	+	68			GAA	0
rrnS	rRNA	1254	2220	+	967				0
trnV(tac)	tRNA	2221	2286	+	66			TAC	-1
rrnL	rRNA	2286	3867	+	1580				1
trnL2(taa)	tRNA	3869	3943	+	75			TAA	2
nad1	PCG	3946	4902	+	957	ATG	TAA		-1
trnI(gat)	tRNA	4902	4970	+	69			GAT	-3
trnQ(ttg)	tRNA	4968	5040	-	73			TTG	1
trnM(cat)	tRNA	5042	5110	+	69			CAT	0
nad2	PCG	5111	6154	+	1044	ATT	TAG		-2
trnW(tca)	tRNA	6153	6219	+	67			TCA	8
trnA(tgc)	tRNA	6228	6296	-	69			TGC	0
trnN(gtt)	tRNA	6297	6369	-	73			GTT	2
OL		6372	6403	+	32				-1
trnC(gca)	tRNA	6403	6469	-	67			GCA	0
trnY(gta)	tRNA	6470	6536	-	67			GTA	1
cox1	PCG	6538	8082	+	1545	ATG	TAA		-3
trnS2	tRNA	8080	8148	-	69				6
trnD(gtc)	tRNA	8155	8221	+	67			GTC	0
cox2	PCG	8222	8905	+	684	ATG	TAG		3
trnK(ttt)	tRNA	8909	8976	+	68			TTT	2
atp8	PCG	8979	9182	+	204	ATG	TAA		-43
atp6	PCG	9140	9820	+	680	ATG	TAA		-1
cox3	PCG	9820	10603	+	784	ATG	T--		0
trnG(tcc)	tRNA	10604	10672	+	69			TCC	0
nad3	PCG	10673	11029	+	357	ATC	TAG		-10
trnR(tcg)	tRNA	11020	11088	+	69			TCG	0
nad4l	PCG	11089	11385	+	297	ATG	TAA		-7
nad4	PCG	11379	12756	+	1378	ATG	T- -		0
trnH(gtg)	tRNA	12757	12825	+	69			GTG	0
trnS1	tRNA	12826	12884	+	59				0
trnL1(tag)	tRNA	12885	12954	+	70			TAG	-9
nad5	PCG	12946	14757	+	1812	ATA	TA-		1
nad6	PCG	14759	15283	-	525	ATA	TAA		3
trnE(tcc)	tRNA	15287	15355	-	69			TCC	4
cob	PCG	15360	16499	+	1140	ATG	AGA		0
trnT(tgt)	tRNA	16500	16569	+	70			TGT	-1
trnP(tgg)	tRNA	16569	16634	-	66			TGG	

Table S2. Mitochondrial genome of *Ursus thibetanus* assembled from scat sample SRR6109214. Features and annotation.

Name	Type	Start	Stop	Strand	Length (bp)	Start	Stop	Anticodon	Continuity
Control Region		1	249	+	250				0
OH	origin	250	578	+	329				0
Control Region		579	1276	+	698				0
trnF(gaa)	tRNA	1277	1344	+	68				0
rrnS	rRNA	1345	2311	+	967				0
trnV(tac)	tRNA	2312	2377	+	66				1
rrnL	rRNA	2379	3958	+	1579				1
trnL2(taa)	tRNA	3960	4034	+	74				2
nad1	PCG	4037	4993	+	957	ATG	TAA		-1
trnI(gat)	tRNA	4993	5061	+	69				-3
trnQ(ttg)	tRNA	5059	5131	-	73				1
trnM(cat)	tRNA	5133	5201	+	69				-27
nad2	PCG	5175	6245	+	1071	ATA	TAG		-2
trnW(tca)	tRNA	6244	6310	+	67				8
trnA(tgc)	tRNA	6319	6387	-	69				0
trnN(gtt)	tRNA	6388	6460	-	73				2
OL	origin	6463	6494	+	32				-1
trnC(gca)	tRNA	6494	6560	-	67				0
trnY(gta)	tRNA	6561	6627	-	67				1
cox1	PCG	6629	8173	+	1545	ATG	TAA		-3
trnS2(tga)	tRNA	8171	8239	-	69				6
trnD(gtc)	tRNA	8246	8312	+	67				0
cox2	PCG	8313	8996	+	684	ATG	TAG		3
trnK(ttt)	tRNA	9000	9067	+	68				2
atp8	PCG	9070	9273	+	204	ATG	TAA		-41
atp6	PCG	9231	9911	+	680	ATG	TAA		-1
cox3	PCG	9911	10694	+	738	ATG	T		0
trnG(tcc)	tRNA	10695	10763	+	69				9
nad3	PCG	10773	11110	+	314	ATC	TA		0
trnR(tcg)	tRNA	11111	11179	+	69				0
nad4l	PCG	11180	11476	+	297	ATG	TAA		-7
nad4	PCG	11470	12847	+	1377	ATG	T		0
trnH(gtg)	tRNA	12848	12916	+	69				0
trnS1(gct)	tRNA	12917	12975	+	59				0
trnL1(tag)	tRNA	12976	13045	+	70				9
nad5	PCG	13037	14875	+	1780	ATA	TAA		-24
nad6	PCG	14850	15371	-	522	ATG	TAA		6
trnE(ttc)	tRNA	15378	15446	-	69				4
cob	PCG	15451	16590	+	1140	ATG	AGA		0
trnT(tgt)	tRNA	16591	16660	+	70				-1
trnP(tgg)	tRNA	16660	16725	-	66				

Table S3. Mitochondrial genome of panda bear *Ailuropoda melanoleuca* assembled from scat sample SRR6109207. Features and annotation.

Name	Type	Start	Stop	Strand	Length (bp)	Start Codon	Stop Codon	Anticodon	Continuity
CR		1	1164	+	1165				
OH		271	666	+	396				
trnF(ttc)	tRNA	1165	1233	+	69			gaa	0
rrnS	rRNA	1234	2199	+	966				0
trnV(gta)	tRNA	2200	2267	+	68			tac	-1
rrnL	rRNA	2267	3848	+	1582				+1
trnL2(tta)	tRNA	3850	3924	+	75			taa	+1
nad1	PCG	3926	4882	+	957	ATG	TAA		-1
trnI(atc)	tRNA	4882	4950	+	69			gat	-3
trnQ(caa)	tRNA	4948	5020	-	73			ttg	+1
trnM(atg)	tRNA	5022	5090	+	69			cat	0
nad2	PCG	5091	6134	+	1044	ATA	TAG		-2
trnW(tga)	tRNA	6133	6200	+	68			tca	+7
trnA(gca	tRNA	6208	6276	-	69			tgc	0
trnN(aac)	tRNA	6277	6349	-	73			gtt	+2
OL		6352	6383	+	32				-1
trnC(tgc)	tRNA	6383	6449	-	67			gca	0
trnY(tac)	tRNA	6450	6516	-	67			gta	+2
cox1	PCG	6518	8062	+	1545	ATG	TAA		-2
trnS2(tca)	tRNA	8060	8128	-	69			tga	+6
trnD(gac)	tRNA	8135	8201	+	67			gtc	0
cox2	PCG	8202	8885	+	684	ATG	TAA		+3
trnK(aaa)	tRNA	8889	8955	+	67			ttt	+2
atp8	PCG	8958	9161	+	204	ATG	TAA		-43
atp6	PCG	9119	9799	+	681	ATG	TAA		-1
cox3	PCG	9799	10583	+	785	ATG	TA-		-1
trnG(gga)	tRNA	10583	10651	+	69			tcc	0
nad3	PCG	10652	10999	+	348	ATA	TA-		-1
trnR(cga)	tRNA	10999	11067	+	69			tcg	0
nad4l	PCG	11068	11364	+	297	ATG	TAA		-7
nad4	PCG	11358	12735	+	1378	ATG	T--		0
trnH(cac)	tRNA	12736	12804	+	69			gtg	0
trnS1(agc)	tRNA	12805	12863	+	59			gct	0
trnL1(cta)	tRNA	12864	12933	+	70			tag	0
nad5	PCG	12934	14763	+	1830	ATA	TAA		-26
nad6	PCG	14738	15265	-	528	ATG	TAA		0
trnE(gaa)	tRNA	15266	15334	-	69			ttc	+4
cob	PCG	15339	16478	+	1140	ATG	AGA		0
trnT(aca)	tRNA	16479	16550	+	72			tgt	-1
trnP(cca)	tRNA	16550	16615	-	66			tgg	

Table S4. Mitochondrial genome of panda bear *Ailuropoda melanoleuca* assembled from scat sample SRR6109208. Features and annotation.

Name	Type	Start	Stop	Strand	Length (bp)	Start	Stop	Anticodon	Continuity
CR		1	1335	+	1335				
OH		695	1004	+	310				
trnF	tRNA	1336	1404	+	69				0
rrnS	rRNA	1405	2370	+	966				0
trnV	tRNA	2371	2438	+	68				+1
rrnL	rRNA	2440	4019	+	1580				+1
trnL2	tRNA	4021	4095	+	75				+1
nad1	pcg	4097	5053	+	957	ATG	TAA		-1
trnI	tRNA	5053	5121	+	69				-3
trnQ	tRNA	5119	5191	-	73				+1
trnM	tRNA	5193	5261	+	69				0
nad2	pcg	5262	6305	+	1044	ATA	TAG		-2
trnW	tRNA	6304	6371	+	68				+7
trnA	tRNA	6379	6447	-	69				0
trnN	tRNA	6448	6520	-	73				2
OL		6523	6554	+	32				-1
trnC	tRNA	6554	6620	-	67				0
trnY	tRNA	6621	6687	-	67				+1
cox1	pcg	6689	8233	+	1545	ATG	TAA		-3
trnS2	tRNA	8231	8299	-	69				+6
trnD	tRNA	8306	8372	+	67				0
cox2	pcg	8373	9056	+	684	ATG	TAA		+3
trnK	tRNA	9060	9126	+	67				+2
atp8	pcg	9129	9332	+	204	ATG	TAA		-43
atp6	pcg	9290	9970	+	681	ATG	TAA		-1
cox3	pcg	9970	10753	+	784	ATG	T--		0
trnG	tRNA	10754	10822	+	69				0
nad3	pcg	10823	11169	+	347	ATA	TA-		0
trnR	tRNA	11170	11238	+	69				0
nad4l	pcg	11239	11535	+	297	ATG	TAA		-7
nad4	pcg	11529	12906	+	1378	ATG	T--		0
trnH	tRNA	12907	12975	+	69				0
trnS1	tRNA	12976	13034	+	59				0
trnL1	tRNA	13035	13104	+	70				0
nad5	pcg	13105	14934	+	1830	ATT	TAA		-26
nad6	pcg	14909	15436	-	528	ATG	TAA		0
trnE	tRNA	15437	15505	-	69				+4
cob	pcg	15510	16649	+	1140	ATG	AGA		0
trnT	tRNA	16650	16721	+	72				-1
trnP	tRNA	16721	16786	-	66				

Table S5. Mitochondrial genome of panda bear *Ailuropoda melanoleuca* assembled from scat sample SRR6109209. Features and annotation.

Name	Type	Start	Stop	Strand	Length (bp)	Start	Stop	Anticodon	Continuity
CR		1	1226	+	1226				0
trnF	tRNA	1227	1295	+	69				0
rrnS	rRNA	1296	2261	+	966				0
trnV(gta)	tRNA	2262	2329	+	68				-1
rrnL	rRNA	2329	3910	+	1581				+1
trnL2 (tta)	tRNA	3912	3986	+	75				+7
nad1	PCG	3994	4944	+	951	ATA	TAA		0
trnI (atc)	tRNA	4944	5012	+	69				-3
trnQ (caa)	tRNA	5010	5082	+	73				+1
trnM (atg)	tRNA	5084	5151	+	68				+1
nad2	PCG	5153	6196	+	1044	ATA	TAG		-2
trnW (tga)	tRNA	6195	6262	+	67				+7
trnA (gca)	tRNA	6270	6338	-	69				0
trnN (aac)	tRNA	6339	6411	-	73				+3
trnC (tgc)	tRNA	6445	6511	-	67				0
trnY (tac)	tRNA	6512	6578	-	67				+1
cox1	PCG	6580	8124	+	1545	ATG	TAA		-3
trnS2(tca)	tRNA	8122	8190	-	69				
trnD (gac)	tRNA	8197	8263	+	67				0
cox2	PCG	8264	8947	+	684	ATG	TAA		+3
trnK (aaa)	tRNA	8951	9017	+	67				+2
atp8	PCG	9020	9223	+	204	ATG	TAA		-43
atp6	PCG	9181	9861	+	681	ATG	TAA		+1
cox3	PCG	9861	10644	+	784	ATG	T- -		0
trnG (gga)	tRNA	10645	10713	+	69				0
nad3	PCG	10714	11060	+	347	ATA	TA-		0
trnR (cga)	tRNA	11061	11129	+	69				
nad4l	PCG	11130	11426	+	297	ATG	TAA		-6
nad4	PCG	11420	12797	+	1378	ATG	T- -		0
trnH (cac)	tRNA	12798	12866	+	59				0
trnS1 (agc)	tRNA	12867	12925	+	59				0
trnL1 (cta)	tRNA	12996	12995	+	70				0
nad5	PCG	12996	14825	+	23	ATT	TAA		-25
nad6	PCG	14800	15435	-	528	ATG	TAA		0
trnE (gaa)	tRNA	15436	15555	-	120				0
cob	PCG	15401	16540	+	1140	ATG	AGA		+9
trnT (aca)	tRNA	16541	16612	+	71				0
trnP (cca)	tRNA	16612	16677	-	66				

Table S6. Mitochondrial genome of panda bear *Ailuropoda melanoleuca* assembled from scat sample SRR6109210. Features and annotation.

Name	Type	Start	Stop	Strand	Length (bp)	Start	Stop	Anticodon	Continuity
CR		1	1452	+	1452				
OH		696	1070	+	375				
trnF (gaa)	tRNA	1402	1470	+	69				0
rrnS	rRNA	1471	2436	+	966				0
trnV (tac)	tRNA	2437	2504	+	68				+1
rrnL	rRNA	2506	4085	+	1580				+1
trnL2 (taa)	tRNA	4087	4161	+	75				+1
nad1	PCG	4163	5119	+	957	ATG	TAA		-1
trnI (gat)	tRNA	5119	5187	+	69				-3
trnQ (ttg)	tRNA	5185	5257	-	73				+1
trnM (cat)	tRNA	5259	5327	+	69				0
nad2	PCG	5328	6371	+	1044	ATA	TAG		-2
trnW (tca)	tRNA	6370	6437	+	68				+7
trnA (tgc)	tRNA	6445	6513	-	69				0
trnN (gtt)	tRNA	6514	6586	-	73				+2
OL		6589	6620	+	67				-1
trnC (gca)	tRNA	6620	6686	-	67				0
trnY (gta)	tRNA	6687	6753	-	67				+1
cox1	PCG	6755	8299	+	1545	ATG	TAA		-3
trnS2 (tga)	tRNA	8297	8365	-	69				+6
trnD (gtc)	tRNA	8372	8438	+	67				0
cox2	PCG	8439	9122	+	684	ATG	TAA		+3
trnK(ttt)	tRNA	9126	9192	+	67				+2
atp8	PCG	9195	9398	+	204	ATG	TAA		-43
atp6	PCG	9356	10036	+	681	ATG	TAA		-1
cox3	PCG	10036	10819	+	784	ATG	T - -		0
trnG (tcc)	tRNA	10820	10888	+	69				0
nad3	PCG	10889	11235	+	347	ATA	TA-		0
trnR (tcg)	tRNA	11236	11304	+	69				0
nad4l	PCG	11305	11601	+	297	ATG	TAA		-7
nad4	PCG	11595	12972	+	1378	ATG	T - -		0
trnH (gtg)	tRNA	12973	13041	+	69				0
trnS1 (gct)	tRNA	13042	13100	+	59				0
trnL1 (tag)	tRNA	13101	13170	+	70				0
nad5	PCG	13171	15000	+	1830	ATT	TAA		-26
nad6	PCG	14975	15502	-	528	ATG	TAA		0
trnE (ttc)	tRNA	15503	15571	-	69				+4
cob	PCG	15576	16715	+	1140	ATG	AGA		0
trnT (tgt)	tRNA	16716	16787	+	72				-1
trnP (tgg)	tRNA	16787	16852	-	66				

Table S7. Mitochondrial genome of panda bear *Ailuropoda melanoleuca* assembled from scat sample SRR6109211. Features and annotation.

Name	Type	Start	Stop	Strand	Length (bp)	Start Codon	Stop Codon	Anticodon	Continuity
CR		1	1164	+	1165				
OH		271	666	+	396				
trnF(ttc)	tRNA	1165	1233	+	69			gaa	0
rrnS	rRNA	1234	2199	+	966				0
trnV(gta)	tRNA	2200	2267	+	68			tac	-1
rrnL	rRNA	2267	3848	+	1582				+1
trnL2(tta)	tRNA	3850	3924	+	75			taa	+1
nad1	PCG	3926	4882	+	957	ATG	TAA		-1
trnI(atc)	tRNA	4882	4950	+	69			gat	-3
trnQ(caa)	tRNA	4948	5020	-	73			ttg	+1
trnM(atg)	tRNA	5022	5090	+	69			cat	0
nad2	PCG	5091	6134	+	1044	ATA	TAG		-2
trnW(tga)	tRNA	6133	6200	+	68			tca	+7
trnA(gca	tRNA	6208	6276	-	69			tgc	0
trnN(aac)	tRNA	6277	6349	-	73			gtt	+2
OL		6352	6383	+	32				-1
trnC(tgc)	tRNA	6383	6449	-	67			gca	0
trnY(tac)	tRNA	6450	6516	-	67			gta	+2
cox1	PCG	6518	8062	+	1545	ATG	TAA		-2
trnS2(tca)	tRNA	8060	8128	-	69			tga	+6
trnD(gac)	tRNA	8135	8201	+	67			gtc	0
cox2	PCG	8202	8885	+	684	ATG	TAA		+3
trnK(aaa)	tRNA	8889	8955	+	67			ttt	+2
atp8	PCG	8958	9161	+	204	ATG	TAA		-43
atp6	PCG	9119	9799	+	681	ATG	TAA		-1
cox3	PCG	9799	10583	+	785	ATG	TA-		-1
trnG(gga)	tRNA	10583	10651	+	69			tcc	0
nad3	PCG	10652	10999	+	348	ATA	TA-		-1
trnR(cga)	tRNA	10999	11067	+	69			tcg	0
nad4l	PCG	11068	11364	+	297	ATG	TAA		-7
nad4	PCG	11358	12735	+	1378	ATG	T--		0
trnH(cac)	tRNA	12736	12804	+	69			gtg	0
trnS1(agc)	tRNA	12805	12863	+	59			gct	0
trnL1(cta)	tRNA	12864	12933	+	70			tag	0
nad5	PCG	12934	14763	+	1830	ATA	TAA		-26
nad6	PCG	14738	15265	-	528	ATG	TAA		0
trnE(gaa)	tRNA	15266	15334	-	69			ttc	+4
cob	PCG	15339	16478	+	1140	ATG	AGA		0
trnT(aca)	tRNA	16479	16550	+	72			tgt	-1
trnP(cca)	tRNA	16550	16615	-	66			tgg	

Table S8. Mitochondrial genome of panda bear *Ailuropoda melanoleuca* assembled from scat sample SRR6109216. Features and annotation.

Name	Type	Start	Stop	Strand	Length (bp)	Start	Stop	Anticodon	Continuity
Control		1	1316						0
OH		695	988	+	294				0
trnF(gaa)	tRNA	1317	1385	+	69				0
rrnS	rRNA	1386	2351	+	966				0
trnV(tac)	tRNA	2352	2419	+	68				+1
rrnL	rRNA	2421	4000	+	1580				+1
trnL2(taa)	tRNA	4002	4076	+	75				+1
nad1	PCG	4078	5034	+	957	ATG	TAA		-1
trnI(gat)	tRNA	5034	5102	+	69				-3
trnQ(ttq)	tRNA	5100	5172	-	73				+1
trnM(cat)	tRNA	5174	5242	+	69				0
nad2	PCG	5243	6286	+	1044	ATA	TAG		-2
trnW(tca)	tRNA	6285	6352	+	68				+7
trnA(tgc)	tRNA	6360	6428	-	69				0
trnN(gtt)	tRNA	6429	6501	-	73				+2
OL		6504	6535	+	32				-1
trnC(gca)	tRNA	6535	6601	-	67				0
trnY(gta)	tRNA	6602	6668	-	67				+1
cox1	PCG	6670	8214	+	1545	ATG	TAA		-3
trnS2(tga)	tRNA	8212	8280	-	69				+6
trnD(gtc)	tRNA	8287	8353	+	67				0
cox2	PCG	8354	9037	+	684	ATG	TAA		+3
trnK(ttt)	tRNA	9041	9107	+	67				+2
atp8	PCG	9110	9313	+	204	ATG	TAA		-43
atp6	PCG	9271	9951	+	681	ATG	TAA		-1
cox3	PCG	9951	10734	+	784	ATG	T-		0
trnG(tcc)	tRNA	10735	10803	+	69				0
nad3	PCG	10804	11150	+	347	ATA	TA-		0
trnR(tcg)	tRNA	11151	11219	+	69				0
nad4l	PCG	11220	11516	+	297	ATG	TAA		-7
nad4	PCG	11510	12887	+	1378	ATG	T–		0
trnH(gtg)	tRNA	12888	12956	+	69				0
trnS1(gct)	tRNA	12957	13015	+	59				0
trnL1(tag)	tRNA	13016	13085	+	70				-9
nad5	PCG	13086	14915	+	1830	ATT	TAA		-26
nad6	PCG	14890	15417	-	528	ATG	TAA		0
trnE(ttc)	tRNA	15418	15486	-	69				+4
cob	PCG	15491	16630	+	1140	ATG	AGA		0
trnT(tgt)	tRNA	16631	16702	+	72				-1
trnP(tgg)	tRNA	16702	16767	-	66				

Table S9. Mitochondrial genome of Java pangolin *Manis javanica* assembled from scat sample SRR7477311. Features and annotation.

Name	Type	Start	Stop	Strand	Length (bp)	Start	Stop	Anticodon	Continuity
trnF(gaa)	tRNA	1	68	+	68				0
rrnS	rRNA	69	1029	+	961				-1
trnV(tac)	tRNA	1029	1094	+	66				0
rrnL	rRNA	1095	2665	+	1571				0
trnL2(taa)	tRNA	2666	2739	+	74				3
**nad1**	**PCG**	**2743**	**3699**	**+**	**957**	**ATG**	**TAA**		**-1**
trnl(gat)	tRNA	3699	3767	+	69				-3
trnQ(ttg)	tRNA	3765	3837	-	73				1
trnM(cat)	tRNA	3839	3907	+	69				0
**nad2**	**PCG**	**3908**	**4948**	**+**	**1041**	**ATA**	**TAG**		**-2**
trnW(tca)	tRNA	4947	5013	+	67				3
trnA(tgc)	tRNA	5017	5085	-	69				1
trnN(gtt)	tRNA	5087	5159	-	73				2
OL		5162	5193	+	32				-1
trnC(gca)	tRNA	5193	5257	-	65				0
trnY(gta)	tRNA	5258	5324	-	67				1
**cox1**	**PCG**	**5326**	**6876**	**+**	**1551**	**ATG**	**AGA**		**-5**
trnS2(tga)	tRNA	6872	6940	-	69				7
trnD(gtc)	tRNA	6948	7014	+	67				0
**cox2**	**PCG**	**7015**	**7698**	**+**	**684**	**ATG**	**TAA**		**2**
trnK(aaa)	tRNA	7701	7764	+	64				1
**atp8**	**PCG**	**7766**	**7966**	**+**	**201**	**ATG**	**TAA**		**-40**
**atp6**	**PCG**	**7927**	**8607**	**+**	**681**	**ATG**	**TAA**		**-1**
**cox3**	**PCG**	**8607**	**9391**	**+**	**785**	**ATG**	**TA-**		**-1**
trnG(tcc)	tRNA	9391	9459	+	69				0
nad3	PCG	9460	9806	+	347	ATA	TA-		0
trnR(tcg)	tRNA	9807	9873	+	67				0
**nad4l**	**PCG**	**9874**	**10,170**	**+**	**297**	**ATG**	**TAA**		**-7**
**nad4**	**PCG**	**10,164**	**11,541**	**+**	**1378**	**ATG**	**T--**		**0**
trnH(gtg)	tRNA	11,542	11,609	+	68				0
trnS1(gct)	tRNA	11,610	11,668	+	59				1
trnL1(tag)	tRNA	11,670	11,740	+	71				0
**nad5**	**PCG**	**11,741**	**13,561**	**+**	**1821**	**ATT**	**TAA**		**-17**
**nad6**	**PCG**	**13,545**	**14,069**	**-**	**525**	**ATG**	**AGA**		**0**
trnE(ttc)	tRNA	14,070	14,138	-	69				3
**cob**	**PCG**	**14,142**	**15,281**	**+**	**1140**	**ATG**	**AGA**		**0**
trnT(tgt)	tRNA	15,282	15,348	+	67				-1
trnP(tgg)	tRNA	15,348	15,413	-	66				161
OH		15,575	16,012	+	438				0
CR		16,013	16,576		564				

Table S10. Mitochondrial genome of Java pangolin *Manis javanica* assembled from scat sample SRR7498027. Features and annotation.

Name	Type	Start	Stop	Strand	Length (bp)	Start	Stop	Anticodon	Continuity
trnF(ttc)	tRNA	1	68	+	68				0
rrnS	rRNA	69	1029	+	961				0
trnV(gta)	tRNA	1029	1094	+	66				-1
rrnL	rRNA	1093	2665	+	1573				-2
trnL2(tta)	tRNA	2666	2739	+	74				0
nad1	PCG	2743	3700	+	958	ATG	TAA		+3
trnI(atc)	tRNA	3699	3767	+	69				-2
trnQ(caa)	tRNA	3765	3837	-	73				-3
trnM(atg)	tRNA	3839	3907	+	69				+1
nad2	PCG	3908	4948	+	1041	ATA	TAG		0
trnW(tga)	tRNA	4947	5013	+	67				-2
trnA(gca)	tRNA	5017	5085	-	69				+3
trnN(aac)	tRNA	5087	5159	-	73				+1
trnC(tgc)	tRNA	5193	5257	-	65				+33
trnY(tac)	tRNA	5258	5324	-	67				0
cox1	PCG	5326	6876	+	1551	ATG	AGA		+1
trnS2(tca)	tRNA	6872	6940	-	69				-3
trnD(gac)	tRNA	6948	7014	+	67				+7
cox2	PCG	7015	7698	+	684	ATG	TAA		0
trnK(aaa)	tRNA	7701	7764	+	64				+2
atp8	PCG	7766	7969	+	203	ATG	TAA		+1
atp6	PCG	7927	8607	+	681	ATG	TAA		-43
cox3	PCG	8607	9391	+	785	ATG	TA-		-1
trnG(gga)	tRNA	9391	9459	+	69				-1
nad3	PCG	9460	9806	+	347	ATA	TA-		0
trnR(cga)	tRNA	9807	9873	+	67				0
nad4l	PCG	9874	10170	+	297	ATG	TAA		0
nad4	PCG	10164	11541	+	1378	ATG	T--		-3
trnH(cac)	tRNA	11542	11609	+	68				0
trnS1(agc)	tRNA	11610	11668	+	59				0
trnL1(cta)	tRNA	11670	11740	+	71				+1
nad5	PCG	11741	13561	+	1821	ATT	TAA		0
nad6	PCG	13545	14069	-	525	ATG	AGA		-17
trnE(gaa)	tRNA	14070	14138	-	69				0
cob	PCG	14142	15281	+	1140	ATG	AGA		+3
trnT(aca)	tRNA	15282	15348	+	67				0
trnP(cca)	tRNA	15348	15413	-	66				-1
OH		15575	16012	+	438				

Table S11. Mitochondrial genome of Java pangolin *Manis javanica* assembled from scat sample SRR7507293. Features and annotation.

Name	Type	Start	Stop	Strand	Length (bp)	Start	Stop	Anticodon	Continuity
trnF(gaa)	tRNA	1	68	+	68				0
rrnS	rRNA	69	1029	+	961				-1
trnV(tac)	tRNA	1029	1094	+	66				0
rrnL	rRNA	1095	2665	+	1571				0
trnL2(taa)	tRNA	2666	2739	+	74				+3
nad1	PCG	2743	3699	+	957	ATG	TAA		-1
trnI(gat)	tRNA	3699	3767	+	69				-3
trnQ(ttg)	tRNA	3765	3837	-	73				+1
trnM(cat)	tRNA	3839	3907	+	69				0
nad2	PCG	3908	4948	+	1041	ATA	TAG		-2
trnW(tca)	tRNA	4947	5013	+	67				+3
trnA(tgc)	tRNA	5017	5085	-	69				+1
trnN(gtt)	tRNA	5087	5159	-	73				+2
OL		5162	5193	+	32				-1
trnC(gca)	tRNA	5193	5257	-	65				0
trnY(gta)	tRNA	5258	5324	-	67				+1
cox1	PCG	5326	6876	+	1551	ATG	AGA		-5
trnS2(tga)	tRNA	6872	6940	-	69				+7
trnD(gtc)	tRNA	6948	7014	+	67				0
cox2	PCG	7015	7698	+	684	ATG	TAA		+2
trnK(ttt)	tRNA	7701	7764	+	64				+1
atp8	PCG	7766	7969	+	204	ATG	TAA		-43
atp6	PCG	7927	8607	+	681	ATG	TAA		-1
cox3	PCG	8607	9391	+	785	ATG	TA -		-1
trnG(tcc)	tRNA	9391	9459	+	69				0
nad3	PCG	9460	9806	+	347	ATA	TA -		0
trnR(tcg)	tRNA	9807	9873	+	67				0
nad4l	PCG	9874	10170	+	297	ATG	TAA		-7
nad4	PCG	10164	11541	+	1378	ATG	T - -		0
trnH(gtg)	tRNA	11542	11609	+	68				0
trnS1(gct)	tRNA	11610	11668	+	59				+1
trnL1(tag)	tRNA	11670	11740	+	71				0
nad5	PCG	11741	13561	+	1821	ATT	TAA		-17
nad6	PCG	13545	14069	-	525	ATG	AGA		0
trnE(ttc)	tRNA	14070	14138	-	69				+3
cob	PCG	14142	15281	+	1140	ATG	AGA		0
trnT(tgt)	tRNA	15282	15348	+	67				-1
trnP(tgg)	tRNA	15348	15413	-	66				+161
OH		15575	16012	+	438				-599
CR		15414	16576		1163				0

Table S12. Mitochondrial genome of Java pangolin *Manis javanica* assembled from scat sample SRR7524043. Features and annotation.

Name	Type	Start	Stop	Strand	Length (bp)	Start Codon	Stop Codon	Anti-codon	Continuity
trnF(ttc)	trna	1	68	+	68				0
rrnS	rrna	69	1029	+	961				-1
trnV(gta)	trna	1029	1094	+	66				0
rrnL	rrna	1095	2665	+	1571				0
trnL2(tta)	trna	2666	2739	+	74				3
nad1	PCG	2743	3699	+	957	ATG	TAA		-1
trnI(atc)	trna	3699	3767	+	69				-3
trnQ(caa)	trna	3765	3837	-	73				1
trnM(atg)	trna	3839	3907	+	69				3
nad2	PCG	3908	4948	+	1041	ATA	TAG		-2
rnW(tga)	rrna	4947	5013	+	67				3
trnA(gca)	trna	5017	5085	-	69				1
trnN(aac)	trna	5087	5159	-	73				2
OL		5162	5193		31				-1
trnC(tgc)	trna	5193	5257	-	65				0
trnY(tac)	trna	5258	5324	-	67				1
cox1	PCG	5326	6876	+	1551	ATG	AGA		-5
trnS2(tca)	trna	6872	6940	-	69				7
trnD(gac)	trna	6948	7014	+	67				0
cox2	PCG	7015	7698	+	684	ATG	TAA		2
trnK(aaa)	trna	7701	7764	+	64				1

atp8	PCG	7766	7969	+	204	ATG	TAA		-43
atp6	PCG	7927	8607	+	681	ATG	TAA		-1
cox3	PCG	8607	9391	+	784	ATG	TA-		-1
trnG(gga)	trna	9391	9459	+	69				-3
nad3	PCG	9460	9805	+	345	ATA	T-		1
trnR(cga	trna	9807	9873	+	67				0
nad4l	PCG	9874	10170	+	297	ATG	TAA		-7
nad4	PCG	10164	11541	+	1378	ATG	T-		0
trnH(cac)	trna	11542	11609	+	68				0
trnS1(agc)	trna	11610	11668	+	58				1
trnL1(cta)	trna	11670	11740	+	71				0
nad5	PCG	11741	13561	+	1821	ATA	TAA		-17
nad6	PCG	13545	14069	-	525	ATG	AGA		0
trnE(gaa)	trna	14070	14138	-	69				3
cob	PCG	14142	15281	+	1139	ATG	AGA		0
trnT(aca	trna	15282	15348	+	67				-1
trnP(cca)	trna	15348	15413	-	66				
OH		15575	16012	+	438				
CR		15414	16576		1163				

Table S13. Mitochondrial genome of *Eubalaena glacialis* assembled from scat sample SRR4056912. Features and annotation.

Name	Type	Start	Stop	Strand	Length (bp)	Start	Stop	Anticodon	Continuity
trnF(ttc)	tRNA	1	73	+	73				1
rrnS	rRNA	75	1048	+	974				0
trnV (gta)	tRNA	1049	1115	+	67				0
rrnL	rRNA	1116	2689	+	1573				1
trnL2 (tta)	tRNA	2691	2765	+	75				-1
nad 1	PCG	2768	3724	+	957	ATG	TAA		-1
trnI (atc)	tRNA	3724	3792	+	69				-3
trnQ (caa)	tRNA	3790	3862	-	73				1
trnM (atg)	tRNA	3864	3932	+	69				0
nad 2	PCG	3933	4976	+	1044	ATA	TAG		-2
trnW (tga)	tRNA	4975	5043	+	69				5
trnA (gca)	tRNA	5049	5117	-	69				1
trnN (aac)	tRNA	5119	5192	-	74				2
OL		5195	5225	+	31				-1
trnC (tgc)	tRNA	5225	5292	-	68				0
trnY (tac)	tRNA	5293	5359	-	67				1
cox 1	PCG	5361	6911	+	1551	ATG	AGA		-5
trnS2 (tca)	tRNA	6907	6975	-	69				7
trnD (gac)	tRNA	6983	7050	+	68				0
cox 2	PCG	7051	7734	+	684	ATG	TAA		3
trnK (aaa)	tRNA	7738	7804	+	67				1
atp8	PCG	7806	7997	+	192	ATG	TAA		-4
atp6	PCG	7994	8647	+	654	ATA	TAA		-1
cox3	PCG	8647	9432	+	786	ATG	TAG		-1

trnG (gga)	tRNA	9432	9500	+	69			0
nad3	PCG	9501	9847	+	347	ATA	TA-	0
trnR (cga)	tRNA	9848	9916	+	69			0
nad4l	PCG	9917	10213	+	297	GTG	TAA	-7
nad4	PCG	10207	11584	+	1378	ATG	T--	0
trnH (cac)	tRNA	11585	11653	+	69			0
trnS1 (agc)	tRNA	11654	11714	+	61			1
trnL1 (cta)	tRNA	11716	11785	+	70			0
nad5	PCG	11786	13606	+	1821	ATA	TAA	-17
nad6	PCG	13590	14117	-	528	ATG	TAA	0
trnE (gaa)	tRNA	14118	14186	-	69			4
cob	PCG	14191	15330	+	1140	ATG	AGA	0
trnT (aca)	tRNA	15331	15402	+	72			-1
trnP (cca)	tRNA	15402	15468	-	67			229
OH		15698	16107	+	410			0
Control Region		15468	16387		919			

Table S14. Mitochondrial genome of *Eubalaena glacialis* assembled from scat sample SRR4056914. Features and annotation.

Name	Type	Start	Stop	Strand	Length (bp)	Start	Stop	Anticodon	Continuity
trnF(ttc)	tRNA	1	73	+	73				+1
rrnS	rRNA	75	1048	+	974				0
trnV(gta)	tRNA	1049	1115	+	67				-2
**rrnL**	rRNA	1114	2689	+	1576				+1
trnL2(tta)	tRNA	2691	2765	+	75				+2
nad1	PCG	2768	3724	+	957	ATG	TAA		-1
trnI(atc)	tRNA	3724	3792	+	69				-3
trnQ(caa)	tRNA	3790	3862	-	73				+1
trnM(atg)	tRNA	3864	3932	+	69				0
nad2	PCG	3933	4976	+	1044	ATA	TAG		-2
trnW(tga)	tRNA	4975	5043	+	69				+5
trnA(gca)	tRNA	5049	5117	-	69				+1
trnN(aac)	tRNA	5119	5192	-	74				+32
trnC(tgc)	tRNA	5225	5292	-	68				0
trnY(tac)	tRNA	5293	5359	-	67				+1
cox1	PCG	5361	6911	+	1551	ATG	AGA		-5
trnS2(tca)	tRNA	6907	6975	-	69				+7
trnD(gac)	tRNA	6983	7050	+	68				0
cox2	PCG	7051	7734	+	684	ATG	TAA		+3
trnK(aaa)	tRNA	7738	7804	+	67				+1
atp8	PCG	7806	7997	+	192	ATG	TAA		-4
atp6	PCG	7994	8647	+	681	ATG	TAA		-1
cox3	PCG	8647	9432	+	786	ATG	TAG		-1
trnG(gga)	tRNA	9432	9500	+	69				0
nad3	PCG	9501	9847	+	347	ATA	TA-		0
trnR(cga)	tRNA	9848	9916	+	69				0
nad4l	PCG	9917	10213	+	282	GTG	TAA		-7
nad4	PCG	10207	11584	+	1395	ATG	T–		0
trnH(cac)	tRNA	11585	11653	+	69				0
trnS1(agc)	tRNA	11654	11714	+	61				+1
trnL1(cta)	tRNA	11716	11785	+	70				0
nad5	PCG	11786	13606	+	1821	ATA	TAA		-27
nad6	PCG	13590	14117	-	525	ATG	TAA		0
trnE(gaa)	tRNA	14118	14186	-	69				+4
cob	PCG	14191	15330	+	1140	ATG	AGA		0
trnT(aca)	tRNA	15331	15402	+	72				-1
trnP(cca)	tRNA	15402	15468	-	67				
OH		15698	16107	+	410				
Control Region		15469	16387		919				

Assemblies

>SRR6109207 (circular) ReOrganized
ATACTATAAATCCACCTCTCATTTTATTCACTTCATACATGCTATTACACACTCTGTGCCATC
ATAGTATGTTTTCATACATCCTCCCTTCTTTCACACCCTATGTATATCGTACATTAATGGTGTA
CCCCCCCTCCCCCTATGTATATCGTGCATTAATGGCGTGCCCCATGCATATAAGCATGTACAT
ACTGTGCTTGGCTTTACATGAGGATACTCATTACAAGAACTTATTTCAAGCGATAGTCTATG
AGCATGTATTTCACTTAGTCCAAGAGCTTGATCACCAAGCCTCGAGAAACCAGCAATCCTTG
CGAGTACGTGTACCTCTTCTCGCTCCGGGCCCATAATTTGTGGGGGTTTCTATACTGAAACTA
TACCTGGCATCTGGTTCTTACCTCAGGGCCATGTTAGCGTCAACTCAATCCTACTAACCCTTC
AAATGGGACATCTCGATGGACTAATGACTAATCAGCCCATGATCACACATAACTGTGGTGTC
ATGCATTTGGTATTTTTTAATTTTAGGGGGGGAACTTGCTATGACTCAGCTATGACCGTAAAG
GTCTCGTCGCAGTCAAATCAGCTGTAGCTGGGCTTATTCATCTTTCGAGGCTCCTCATGGACA
CCCATAAGGTGCAATTCAGTCAATGGTCACAGGACATAACACTATAGATCACCCGGACTGGC
GTTACGTGTACGTACGTGTACGTACGTGTACGCACGTGTACGTACGTGTACGCACGTGTACG
TACGTGTACGTACGTGTACGCACGTGTACGCACGTGTACGCACGTGTACGCACGTGTACGCA
CGTGTACGTACGTGTACGCGTACGTATTTTAGATACTAAGTTAGCTTAGACAAACCCCCCTT
ACCCCCCGTAACTTCAAGAAGCTTACATATACTTATGGATGTCCTGCCAAACCCCAAAAACA
AGACTAAATATATGCGCAAACATGAAGTCACTTACACCTAAACCCATATAATTAAGCTAACC
CCCCAGCCAATGTTGCAACAACTACGGACATGGGACTCTAAATTTTAATTTATCTATAGATA
TTTTTCTTTTACTGTGTCTCCCCAGCATTGATTTTTTAATTATCATTATTCCACACCACCAATT
TCCATTGAGCTATTTCACATGAGTTCCAAATCAATTATGTTCATGTAGCTTAACGAATAAAGC
AAGGTACTGAAAATGCCTAGATGGGTCACGCTACCCCATAGACATAAAGGTTTGGTCCTAGC
CTTCCTATTAGCCATTAACAAGATTACACATGTAAGTCTCCACGCTCCAGTGAAAATGCCCC
TTAAGTCCTCTTAGACGACCTAAAGGAGCGGGTATCAAGCACACCTTATGGTAGCTCACAAC
GCCTTGCTTAGCCACACCCCCACGGGAAACAGCAGTGATAAAAATTAAGCTATGAACGAAA
GTTCGACTAAGCTATGTTAATACTAGGGTTGGTAAATCTCGTGCCAGCCACCGCGGTCATAC
GATTAACTCGAGTTAATAGGCCTACGGCGTAAAGCGTGTAAAAGAAAAAATCTCCTCTACTA
AAGTTAAAGTATGATTAAGCTGTAAAAAGCTACCATTAATACTAAAATAAACTACGAAAGT
GACTTTAAAATTTCTGATTACACGATAGCTAGGGCCCAAACTGGGATTAGATACCCCACTAT
GCCTAGCTCTAAACATAGATATTTTACTAAACAAAACTATTCGCCAGAGAACTACTAGCAAC
AGCTTAAAACTCAAAGGACTTGGCGGTGCTTTATATCCCCCTAGAGGAGCCTGTTCTGTAAT
CGATAAACCCCGATAGACCTCACCATCCCTTGCTAATTCAGTTTATATACCGCCATCTTCAGC
AAACCCTTAAAAGGAAAAAAAGTAAGCATAACTACCCTACATAAAAAAGTTAGGTCAAGGT
GTAACCTATGGGCTGGGAAGAAATGGGCTACATTTTCTATTCAAGAACAACTTCTACGAAAA
CTTTTATGAAACTAAAAGCTAAAGGCGGATTTAGTAGTAAATTAAGAATAGAGAGCTTAATT
GAACAGGGCAATGAAGCACGCACACACCGCCCGTCACCCTCCTCGAGTGATATAATTTAATT
ATAACCTATTTAAACTAAGCAAAGCATAAGAGGAGACAAGTCGTAACAAGGTAAGCATACT
GGAAAGTGTGCTTGGATGAGCCAAAGTGTAGCTTAAACAAAGCGTCTGGCTTACATCCAGA
AGATTTCATTAATATATGACTACTTTGAACCCAAAGCTAGCCCAAGCAACAATGACTAGTAA
AACCATTATGAAACATTCAAACAAAACATTTAGTAGCATGACTAGAGTATAGGAGATAGAA
ATTTTTAACTGGAGCTATAGAGAGAGTACCGCAAGGGAATGATGAAAGATTACCTAAAGTG
ATAAACAGCAAAGATTGCCCCTTCTACCTTTTGTATAATGAGTTAGCTAGAAATAACTTAAC
AAAGAGAACTTAAGCTAAGTCCCCCGAAACCAGACGAGCTACCTATGAACAATCCACTGGG
ATGAACTCATCTATGTTGCAAAATAGTGAGAAGATCCATAGGTAGAGGTGAAAGGCCTAAC
GAGCCTGGTGATAGCTGGTTGCCCAGAATAGAATTTTAGTTCGACTTTAAACCTGCCTACAA
AACTAATAATTCTAATGCAGATTTAAAATATATTCTAAAAAGGTACAGCTTTTTAGAGTTAA
GGATACAGCCTTACTTAGAGAGTAAATATTTATATAAGCCATAGTAGGCCTAGAGGCAGCCA
TCAATTAAGAAAGCGTTAAAGCTCAACATCTCTATTAACTTAATACCAAGAATATTTAATCA
ACTCCTAATGTATTACTGGGTCAATCTATTTAAATATAGAAGTGATAATGCTAATATGAGTA

ACAAGAAATATTTCTCCCATGCATAAGCTTATAACAGCAACGGATAACCACTGATAGTTAAC
AACAACATAGAAATAACCTAATGATAAAACACCTATTAAATCAATTGTTAGTCCAACACAG
GCATGCAATCAGGGAAAGATTAAAAGAAGTGAAAGGAACTCGGCAAATACAAACCCCGCCT
GTTTACCAAAAACATCACCTCCAGCATTTCCAGTATTGGAGGCACTGCCTGCCCGGTGACAT
CAGTTAAACGGCCGCGGTATTCTGACCGTGCAAAGGTAGCATAATCATTTGTTCTCTAAATA
AGGACTTGTATGAATGGCCACACGAGGGTTTAACTGTCTCTTACTTCCAATCAGTGAAATTG
ACCTCCCCGTGAAGAGGCGGGGATAAGACAATAAGACGAGAAGACCCTATGGAGCTTTAAT
TAACTAATTCAAAAAGAAACTACTAACGACCCAACAGGAATAATATATCTCTTTTATGAATT
AGCAATTTAGGTTGGGGCGACCTCGGAGGACAAAATAGCCTCCGAGTGATTATAAATCTAG
ACTTACCAGTCAAAATGCTTAATCACTTATTGATCCAAAAATTCTTTTGATCAACGGAACAA
GTTACCCTAGGGATAACAGCGCAATCCTATCCGAGAGTCCATATCGACAATAGGGTTTACGA
CCTCGATGTTGGATCAGGACATCCTAATGGTGCAGCAGCTATTAAAGGTTCGTTTGTTCAAC
GATTAAAGTCCTACGTGATCTGAGTTCAGACCGGAGCAATCCAGGTCGGTTTCTATCTATTC
AAATAATTTCTCCCAGTACGAAAGGACAAGAGAAATAAGGCCTACTTCTCTGAAGCGCCTTA
AGACCAATAGATGAATTTATCTAAATCTAGTAAATCTAACTCCAATATTGCCCAAGAGACAG
GGCTTTGTTAGGGTGGCAGAGCCCGGTAATTGTGCAAAACTTAAACTCTTGTGTCCAGAGGT
TCAATTCCTCTCCCTAGCATATGTTTATAATTAACATCTTCTCACTAATTGTACCTATTCTTCT
TGCTGTAGCCTTTCTGACTCTAGTAGAACGAAAAGTACTAGGCTACATACAGCTTCGAAAAG
GCCCAAATATTGTAGGTCCATATGGTCTCCTACAACCAATCGCAGATGCCGTAAAACTCTTT
ACTAAAGAACCTTTACGACCACTTACGTCATCCATAACTATATTTATTATAGCCCCTATTCTA
GCCTTAACACTAGCTCTGACCATGTGAGTCCCCTTACCAATACCGCACCCCCTTATTAACATA
AACCTAGGAGTACTATTCATACTAGCTATATCAAGTCTAGCTGTATACTCTATTCTCTGATCT
GGATGAGCCTCAAACTCAAAATATGCCCTAATTGGAGCCCTACGAGCCGTAGCTCAAACGAT
CTCATATGAAGTCACACTAGCCATTATCCTTTTATCCGTTCTACTAATAAATGGCTCATTTAC
GCTATCCACGCTAATCATTACCCAAGAACATCTTTGATTAATCTTCCCTGCATGACCACTAGC
TATAATATGATTTATTTCTACCCTAGCAGAAACAAATCGAGCCCCCTTTGACCTAACAGAAG
GAGAATCAGAACTAGTCTCTGGGTTTAATGTCGAATATGCAGCAGGTCCATTTGCCCTGTTC
TTCCTAGCAGAATATGCCAACATCATCATAATAAATGCTCTTACAACTATTCTATTCTTCGGA
GCATTTCACAGCCCCTACATACCAGAATTATACATTGTTAATTTTACCGTAAAAACTTTACTC
CTCACAATCTCTTTTCTATGAATCCGAGCATCATATCCTCGATTCCGATACGACCAACTAATA
CACCTACTATGAAAAAACTTTCTACCTCTCACACTAGCTCTATGTATATGACATGTAACTCTA
CCTATTATTACAGCAAGCATTCCACCTCAAACGTAAGAAATATGTCTGACAAAAGAATTACT
TTGATAGAGTAAGCCATAGGGGTTTAAGTCCCCTTATTTCTAGAATCATAGGAATCGAACCT
AATCTTAAGAACTCAAAAATCTTCGTGCTACCTAACTACACCATATCCTAAAGTAAGGTCAG
CTAAATAAGCTATCGGGCCCATACCCCGAAAATGTTGGTTTATCCCCTTCCCATACTAATAA
AACCAGCCATCCTTATTACCATTACATCCACCGTTGTCTTAGGAACTATAATTGTTCTATTTA
GCTCCCACTGATTCATAATCTGAGTTGGCTTTGAAATAAATATACTAGCCATTATCCCAATTC
TAATAAAAAAATATAACCCACGAGCCATAGAAGCTTCCACAAAATATTTTCTAACACAAGCT
ACAGCATCCATGCTTCTAATGCTAAGCATCATCATCAATCTACTATGCTCAGGACACTGGAC
AGTTTCAACAATTCCCAATCCAGTAGCATCTACCATAATTACTATTGCTCTAACAATAAAACT
TGGCCTATCACCATTTCACTTTTGAGTCCCAGAAGTTACACAAGGCATTTCCTTATCTTCAGG
AATAATCCTATTAACATGACAAAAAATTGCACCTTTATCCATTCTATACCAAATTTCACCATC
CGTTAACTCAAATCTCTTACTAATAATAGCTATCACATCCATACTGGTAGGAGGCTGAGGAG
GATTAAATCAAACCCAACTCCGAAAAATCTTGGCATACTCCTCAATTACCCATATAGGCTGA
ATGGCTGCTATTATAGTATATAATCCTACCCTAGCAATTCTTAACCTAACAATTTACATCATA
ATAACTCTAGGAACATTTATACTATTCATACATAGCTCATCTACAACAACACTATCACTATCT
TATACATGAAATAAATTCCCATTAATAGCCCCACTAATCTTAATACTTATACTATCACTAGGA
GGCCTCCCTCCTCTCTCAGGCTTTATCCCCAAATGAATAATTATTCAAGAATTAACAAAAAA
CGACATAATTATTATACCTACACTTATAGCCATTACAGCCCTACTAAACCTATATTTTTACAC
ACGCCTAACATATACTACAGCACTAACTATATTTCCCGCAACAAATAATATAAAAATAAAAT
GACAATTCGAAACCACAAAAAAGACAACTCTCCTAGCCCCTCTAATCGTGACATCAACTATA

CTACTACCACTAACCCCAATACTAGCAGCTCTTGATTAGAGATTTAGGCTAAACAGACCAAG
GGCCTTCAAAGCCTCAAGTAAGTGAAAATCCACTTAATCTCTGCAATATTTAAGGACTGCAA
GAATCTACCTCACATCAACTGAACGCAAAACAATCGCTTTAATTAAGCTAAGCCCTTCTAGA
TCGGTGGGCTTTTACCCCACAAAACTTTAGTTAACAGCTAAAAACCCTAGACAACTGGCTTC
AATCTACTTCTCCCGCCGCGTAGGAAAAAAAGGCGGGAGAAGCCCCGGCAGAATTGAAGCT
GCTTCTTTGAATTTGCAATTCAATATGATTACTCACCACAGAGCCTGGTAAAAAGAGGACTT
AACCCCCATTCTTAGATTTACAGTCTAGTGCTTTTATCAGCCATTTTACCTATGTTCATTAACC
GATGACTGTTTTCCACCAACCACAAAGATATTGGCACTCTTTATCTTCTGTTTGGCGCATGAG
CTGGTATAACGGGCACTGCTCTTAGCCTTCTAATCCGTGCTGAATTAGGTCAGCCTGGAGCT
CTGTTAGGAGATGACCAAATTTACAATGTAGTTGTAACTGCCCACGCATTCGTAATAATCTT
CTTTATAGTTATGCCTATTATGATCGGAGGCTTTGGAAATTGACTAGTACCTTTGATAATTGG
TGCGCCTGATATAGCATTTCCCCGAATAAACAATATGAGTTTCTGATTACTACCTCCATCCTT
TCTTCTCCTTCTGGCTTCTTCCATAGTAGAAGCAGGCGCAGGGACTGGATGAACTGTATACC
CTCCTCTAGCAGGTAACTTAGCCCATGCAGGAGCATCAGTAGACCTAACAATCTTTTCTCTA
CATCTGGCAGGGGTTTCTTCTATTTTAGGGGCTATTAACTTTATTACCACTATCATCAACATG
AAACCCCCTGCAATATCTCAATATCAAACTCCCCTATTCGTGTGATCAGTACTTATCACAGCA
GTACTTCTCCTTCTATCTTTACCAGTCTTAGCAGCTGGAATTACTATACTACTTACAGATCGA
AATCTCAATACTACTTTCTTCGACCCGGCCGGAGGAGGGGATCCCATTCTATATCAACATCT
ATTTTGATTCTTTGGCCATCCTGAAGTCTATATTCTAATCCTCCCTGGGTTTGGAATAATTTCA
CACATTGTCACATACTATTCAGGAAAGAAAGAGCCCTTTGGTTATATGGGAATAGTGTGGGC
AATAATGTCAATTGGATTCTTAGGATTTATTGTATGGGCCCATCATATATTTACTGTAGGCAT
AGATGTTGATACACGAGCCTATTTTACCTCGGCCACTATAATTATTGCAATCCCGACAGGAG
TTAAAGTATTCAGCTGACTAGCTACCCTGCATGGAGGAAATATTAAGTGATCCCCTGCCATA
ATGTGAGCCCTAGGTTTCATTTTTCTATTTACAGTAGGGGGTCTTACAGGCATTGTTCTAGCT
AACTCATCTCTGGACATTGTCCTCCATGACACATACTACGTAGTAGCACACTTCCACTATGTA
CTATCAATAGGGGCCGTGTTCGCTATTATAGGAGGCTTCGTCCATTGATTCCCATTATTTTCG
GGCTATACACTTAATAATACATGAGCAAAAATTCACTTCGTAATTATATTTGTTGGAGTCAA
TATAACATTCTTCCCCCAGCACTTCCTAGGTCTATCAGGTATGCCTCGACGATATTCTGATTA
TCCAGACGCCTATACAGCATGAAACACAGTGTCTTCAATGGGTTCATTCATTTCACTAACAG
CAGTTATGTTAATAATTTTCATAATTTGAGAAGCCTTCGCATCTAAACGAGAAGTGGCAGTG
ATTGAGCTTACTACAACTAATATTGAATGGCTCCACGGATGTCCTCCCCCATACCATACATTC
GAAGAGCCCGCCCACGTCATACTGAAATAAGAAAGGAAGGAATCGAACCCTCTAGAATTGG
TTTCAAGCCAGCATCATAACCACTATGTCTCTCTCAATAAAGAGGTATTAGTAAAAATTACA
TAACTTTGTCAAAGTTGAATTATAGGTGAAAATCCTTTATATCTCTATGGCGCATCCCTTTCA
AACAGGCCTTCAAGATGCAACTTCCCCCATCATGGAAGAACTCCTACACTTCCATGATCATA
CACTAATAATTGTATTCTTAATTAGTTCCTTAGTTCTCTATATCATTTCAATTATATTAACTAC
TAAATTAACACATACAAATACAATAGACGCACAAGAAGTGGAGACAGTATGAACTATTCTA
CCAGCTATCATCTTAATTATAATTGCACTTCCATCACTACGAATTCTTTATATGATAGATGAA
ATTAATAACCCATCATTAACTGTAAAGACTATGGGGCATCAATGATACTGAAGCTATGAGTA
TACAGATTATGAAGACTTGAGCTTCGATTCTTATATAACCCCAACACAAGAATTAAAGCCCG
GAGAGCTGCGATTACTAGAGGTAGATAATCGAGTAGTACTACCCATAGAAATAACAATTCG
CATATTAATTTCCTCAGAAGATGTGCTACATTCGTGAGCTGTCCCATCTCTAGGACTAAAAA
CTGATGCAATCCCGGGGCGATTAAATCAAACAACTCTTATGGCCATGCGACCAGGACTATAT
TATGGCCAATGCTCAGAGATTTGCGGTTCTAATCATAGCTTTATACCCATTGTACTTGAAATA
GTTCCGCTGTCTTACTTTGAAAAATGATCCGCTTCAATACTATAAAATCATTAAGAAGCTTTA
TAGCGTTAACCTTTTAAGTTAAAGACTGAGAGTGCAAGTCTCTCCTTAATGGAAATGCCACA
ATTAGACACATCCACATGATTTATTACAATCCTATCCATGATCTTAACACTATTTATTATATT
TCAGTTAAAAGTCTCAAAACACAACTATCCTGAGTTTCCTGAATTAAAAACAACCCCACTAT
CAAAAAAACCTATGCCTTGAGAAGAAAAATGAACGAAAATCTGTTCGCCTCTTTTACTACCC
CAATGATAATAGGAGTACCAATTGTGGTTCTGATTATTATTTTCCCAAGTATCTTATTTCCCT
CACCTAATCGATTAATCAATAATCGCCTAGCTACTGTCCAACAATGATTAATTCAATTAGTAT

CTAAGCATATACTGTCTATTCACAATTATAAAGGACAAACCTGAGCGCTAATACTAATGTCA
TTAATTTTATTTATTGGCTCAACTAACCTTTTAGGCCTATTACCACATTCATTCACACCTACTA
CACAACTATCAATAAATTTAGGAATGGCCATTCCTCTGTGAGCAGGTACAGTAGTTATAGGA
TTTCGACACAAAACTAAGGCATCTCTGGCCCACTTTCTACCCCAAGGAACACCTCTTCCTCTG
ATCCCAATGTTAGTAATTATCGAGACAATTAGTCTGTTTATTCAACCTGTGGCCCTAGCTGTA
CGACTGACCGCTAATATTACTGCAGGCCATCTACTTATTCATTTAATCGGAGGGGCTACCCT
AGTTTTAATAAACATTAACCCTGTAACAGCCCTAATTACCTTTATTATTCTTATTCTACTTACT
ATTCTCGAATTAGCTGTAGCTCTTATCCAAGCCTATGTTTTCACCCTACTAGTTAGTCTATAC
TTACATGATAATACCTAATGATCCACCAAACACATGCATATCATATAGTTAACCCAAGCCCA
TGGCCACTTACAGGAGCTCTCTCGGCTCTTCTTATAACATCAGGACTAATTATATGATTTCAC
TTTAACTCGGTATACTTACTAATACTAGGCCTCACCACCAACATACTTACCATGTACCAATGA
TGACGAGATATCGTCCGGGAAAGTACCTTCCAAGGCCATCACACCCCAATCGTCCAAAAAG
GACTACGATATGGAATAGTCTTATTTATTGTATCAGAAGTATTTTTCTTCGCGGGATTCTTTT
GGGCCTTCTACCACTCAAGCCTAGCACCTACTCCTGAATTAGGAGCATGTTGACCACCTACA
GGTATCATCCCCTTAAATCCGCTAGAAGTTCCACTACTTAACACCTCTGTACTTCTAGCATCT
GGGGTATCTATCACTTGAGCTCATCATAGTCTAATAGAAGGAGACCGCAAACATATGCTCCA
AGCCCTATTTATTACTATTTCACTAGGAGTTTATTTTACACTTCTACAAGCCTCAGAATACTA
TGAGACATCCTTTACTATTTCAGATGGAGTGTATGGTTCAACCTTCTTTATAGCAACTGGATT
TCATGGGCTTCATGTAATTATTGGTTCAACTTTTCTTACTGTGTGTTTTCTACGACAACTGCAC
TTTCACTTTACATCCAACCATCACTTTGGCTTTGAAGCGGCAGCATGATACTGACACTTCGTA
GACGTTGTATGGCTTTTCCTTTATGTTTCTATTTACTGATGAGGATCCTACTTCTTTAGTATTA
ACTAGTACGATTGACCTCCAATCAATAGGCTCTGGTACAAGCCAGAAAGAAGTAATAAACC
TAATTCTAGCACTTCTAACCAACGTATTATTAGCCTCTCTGCTCGTTCTTATTGCATTCTGACT
GCCACAACTAAATATCTATGCAGAAAAAGTAAGTCCTTATGAATGTGGATTTGATCCTATAG
GATCAGCACGCCTTCCTTTCTCTATAAAATTTTTCTAGTAGCTATTACATTCCTTTTATTCGA
CTTAGAAATTGCTCTACTCCTCCCGCTTCCTTGAGCATTACAGACTAACAACTTAATAACTAT
ACTTATTATAGCTCTATTACTCATTTCCCTATTAGCCGCAAGCCTAGCTTATGAGTGAACCGA
AAAAGGGCTAGAATGGGCCGAATATGATAATTAGTTTAAACTAAAATAAGTGATTTCGACTC
ACTAGATTATGACTAACATCATAATTATCAAATGTCTGTGGTCTATATTAATCTCTTTCTAGC
CTTTACTATATCCTTAATAGGGCTACTCATCTATCGATCCCATTTAATATCCTCCCTATTATGC
TTAGAAGGCATGATGCTGTCACTATTTGTTATACTAGCAGTAACAATCCTAAGTAACCACTT
CACACTAGCCAGTATGACTCCAATCATTCTATTAGTATTCGCTGCCTGTGAAGCAGCGTTGG
GGCTTTCTCTGCTAGTAATAGTCTCTAATACCTATGGAACTGACTACGTACAAAATCTTAACC
TCCTACAATGCTAAAAATTATCCTCCCTACCATAATACTAGTACCCCTTACATGAATATCAAA
GCCTAACATAATCTGAATTAATACAACAACCTATAGTCTACTCATCAGCCTTATTAGTCTGAC
ATATCTCAATCAACTCAGTGATAATAGTCTAAACTTCTCATTAACATTCTTTACCGACTCCCT
ATCAGCCCCCCTGTTAGTTCTCACAACATGACTACTGCCCTTAATACTTATAGCTAGTCAATT
TCACCTGTCAAAAGAGACCCTGACCCGGAAAAAATTATATATCACAATACTAATTCTCCTGC
AACTATTCTTAATTATAACATTTTCTGCTACTGAGTTAATCATATTCTATATTTTATTTGAAGC
TACCCTGGTACCGACTTTAGTTATCATTACTCGATGAGGAAACCAAACAGAACGACTAAACG
CGGGCCTCTATTTTTTATTCTACACCTTAGTAGGATCCTTGCCCTTACTAGTTGCACTGTTATA
TGCCCAAAACAGCTTAGGATCACTAAATTTTCTCATAGTACAGTACTGAACTCAGCCCTTGC
CAAATTCCTGATCTAACATTTTCATGTGGCTGGCATGTATAATAGCATTCATAGTAAAAATA
CCGCTATATGGCTTACACCTATGACTACCTAAAGCACATGTAGAAGCTCCTATTGCAGGATC
CATAGTACTTGCTGCAGTACTTCTAAAACTAGGGGGATACGGTATAATGCGGATTACAACAA
TACTCAACCCTCTAACAAATTTTATAGCCTACCCCTTCATAATGCTCTCACTATGAGGAATAA
TCATAACAAGTTCTATTTGTTTACGCCAGACAGACCTAAAGTCCCTAATTGCATACTCCTCTG
TTAGTCACATAGCCCTAGTCATTGTAGCAATTCTAATCCAATCCCCATGAAGCTACATAGGA
GCTACTGCTCTAATAATCGCCCATGGGTTAACATCTTCTATGCTATTCTGTCTTGCAAACTCT
AACTACGAGCGAACCCATAGCCGAACTATAATTCTCGCACGGGGCTTGCAAACGCTTCTTCC
ACTTATAGCAGCTTGATGACTATTAGCAGGCCTAACTAACTTAGCTCTTCCTCCTACAATTAA

TCTGATTGGGGAACTGTTTGTAGTGATAGCCACATTTTCATGATCCAATATTACCATTCTCTT
AATAGGAGTTAACATTACCATTACTGCTCTCTACTCTCTATATATACTAATTTCTACACAGCG
AGGCAAGTATACAGACCACATCAAAAATATTAAGCCATCTTATACACGAGAAAATGCCTTA
ATAGCCTTACATTTACTACCCCTTTTACTTCTATCTCTTAACCCTAAAGTAGTTCTAGGCTTTA
TTTACTGTAAATATAGTTTAACAAAAACATTAGATTGTGAATCTAGTAATAAAAGCTTAAAT
CTTTTTATTTACCGAAAAAGCATGCAAGAACTGCTAATTCATGCACCCATGCATAAAATCAT
GGCTTTTTCAACTTTTAGGGGATAGAAGTAATCCATTGGTCTTAGGAGCCAAAAAATTGGTG
CAACTCCAAATAAAAGTAATTAACCTACTTACCTCCTTCACCATTACAACCCTATTTATATTA
ATCATGCCCATTATCCTAACCTGTACCCCAGCATATGAAAATAAACTTTACCCGCAATATGT
AAAAACCACAGTCTCTTATGCCTTCACAATTAGCATAATTCCCATAGTAATATTCATCCACTC
CGGACAAGAAGCAATCATCTCAAACTGACACTGAATAACCATCCAAACCCTAAAACTCAGT
CTAAGCTTCAAATTAGATTACTTTTCAATAATCTTCGTACCAGTGGCCCTATTCGTCACATGG
TCAATTATAGAATTCTCTATATGATATATACACGCAGATCCTAACATTAACCGATTCTTTAAA
TATCTACTTATATTCCTCGTCACGATAATAATTCTAGTAACCGCAAATAATCTATTTCAGCTA
TTTATCGGCTGAGAGGGAGTAGGAATCATATCATTTCTCCTCATTGGATGATGATATGGACG
AACAGATGCTAATACGGCTGCCCTACAAGCAGTACTATACAACCGCATTGGAGATGTTGGTT
TTATCATGGCCATAGCTTGATTCTTACTCAATACAAATGCATGGGACTTCCAACAAATCTTTA
TAATCGAGCATAAAAACCTCAATATTCCACTGGCAGGACTACTTCTAGCAGCTACCGGAAAG
TCTGCCCAATTTGGACTTCACCCATGACTCCCATCAGCCATAGAAGGCCCCACTCCTGTCTCA
GCACTACTTCACTCTAGCACAATAGTAGTAGCAGGCGTATTTCTTCTAGTCCGATTTCACCCC
TTAATAGAACATAACAAGACAATTCAAACGGTTACCCTATGCTTAGGGGCAATAACAACCTT
GTTTACAGCAATCTGCGCCCTTACCCAAAATGATATCAAAAAAATTATTGCCTTCTCCACCTC
GAGCCAACTAGGACTAATAATTGTAACAATTGGTATTAATCAGCCTCATCTGGCCTTTCTCC
ATATCTGCACGCACGCATTCTTCAAGGCCATGCTATTTCTATGCTCCGGATCTATTATTCATA
GTCTAAACAACGAACAAGATATTCGAAAGATAGGAGGGCTATTTAAAAGTCTACCATTTACC
ACTACTGCTCTAATCATCGGAACTCTAGCACTTACAGGAATGCCCTTCTTAACAGGATTTTAC
TCCAAAGACTTAATTATTGAAACCGCCAATACGTCATACACCAACGCCTGAGCCTTACTAAC
AACCCTTATCGCCACATCCATAACTGCCGCCTACAGTACTCGAATTGTATTTTTTACACTTCT
AGGACAACCTCGCTATAGTCCCATTATCACCATTAACGAGAATAACCAGTTCCTGACTAACT
CTGTCAAACGATTGCTACTAGGAAGTATCTTTGCGGGATTCCTAATCTCTCATAACATTACAC
CCACCACTGTCCCACAAATAACCATGCCCTGTCACCTAAAAATGATAGCCCTTACAGTAACA
ATCCTAGGCTTTATTCTAGCACTAGAACTTAACCTAGCCATACAAAATCTCAAATTTGACTAC
CCTTTAAGCACGTTCAAATTTTCAAATCAACTAGGATATTTCCCTACTACTCTGCATCGTCTC
GCACCAATAATAGGTCTATCAATAAGCCAAAAAACAGCATCCATACTGCTAGATGCAATCTG
ACTAGAGAATGTTCTACCAAAGTCCATCTCCTACTTTCAAATAAAATCCTCAATCACAGTCTC
CAACCAAAAGGGACTAATTAAACTATACTTTCTCTCCTTTATAATTACACTAATCTTAAGTCT
CTTTATACTTAATTTCCACGAGTGACTTCCATAATTACCAATACACCAATAAGGAGAGATCA
TCCAGTAACAATAACTAATCAAGTACCATAACTATATAAAGCTGCAATTCCCATAGCCTCTT
CACTAAAAAATCCTGAATCCCCTGTGTCATAAATCACCCAATCTCCTATTCCATTAAACTTAA
ATACTACCTCGGCTTCATCATCCTTTAAAATATAACAAACAGCCAGTAACTCAGACAACAGA
CCTACAATAAATGCACCTAGTACAGCCTTATTAGAAAGTCAAACCTCAGGATATTGCTCAGT
GGCTATGGCCGTAGTGTACCCGAACACTACAAGTATACCCCCTAAATAAATTAAAAAGACC
ATTAAACCCAAAAAAGATCCTCCAAAGCTTAACACAATACCACACCCTACACCCCCACTAAC
AATCAAGACTAAACCTCCGTAGATAGGAGAGGGCTTAGAAGAAAACCCCGCAAAACTAACT
ACAAAAACGATACTTAAAATAGATACAATGTATGTTATCATCATTCTTACATGGAATCTAAC
CATGACCAATGACATGAAAAATCATCGTTGTATTTCAACTATAAGAACCCCAATGATCAACA
TCCGAAAAACTCATCCATTAGTTAAAATTATCAACAACTCATTCATTGACCTTCCAACACCAT
CAAACATTTCAACATGATGGAACTTTGGGTCCCTGTTAGGAGTGTGTCTGATCTTGCAAATCT
TAACAGGCTTATTTCTAGCCATACACTATACATCAGATACAGCTACAGCCTTTTCATCAGTCG
CACACATTTGTCGAGACGTCAACTATGGTTGATTTATCCGATATATACATGCCAATGGGGCC
TCTATATTTTTATCTGCCTATTTATACACGTAGGGCGAGGCTTATACTATGGATCATACCTA

TTTCCAGAGACATGGAATATCGGAATTATTCTCCTACTTACAGTTATAGCCACAGCATTCATA
GGGTATGTACTACCTTGAGGACAAATATCCTTCTGAGGAGCAACCGTCATTACTAACCTACT
ATCAGCAATTCCTTACATTGGCACTAATCTAGTGGAGTGAGTCTGAGGGGGTTTCTCCGTAG
ATAAAGCAACACTAACCCGATTTTTTGCTTTTCACTTTATCCTTCCATTTATCATCTCAGCACT
AGCAATAGTCCATCTATTATTCCTTCACGAAACAGGATCTAATAACCCCTCCGGAATTCCAT
CTGACCCAGACAAAATCCCATTTTACCCCTATCATACAATTAAAGACATCCTAGGCGTCCTA
TTTCTTGTCCTCGCCTTAATAACCCTGGCTTTATTCTCACCAGACCTGTTAGGAGACCCTGAT
AACTATACCCCTGCAAATCCACTAAGTACCCCGCCACATATTAAGCCTGAATGGTACTTTCT
ATTTGCCTACGCTATCCTGCGATCTATTCCTAATAAACTAGGAGGGGTGCTAGCTCTAATCTT
CTCTATTCTAATTCTAACTATTATTCCACTATTACATACATCCAAACAACGAAGCATGATATT
CCGACCTCTAAGTCAATGCTTATTCTGACTCCTAGTAGCAGACCTACTCACACTAACATGAA
TTGGAGGACAGCCAGTAGAACACCCCTTCATTATTATTGGGCAATTGGCCTCTATTCTCTACT
TTACAATTCTTCTAGTACTTATACCTATCACTAGCATTATTGAGAATAGCCTCTCAAAATGAA
GAGTCTTTGTAGTATATTAATTACTTTGGTCTTGTAAACCAAAAACGGAGATTCCTCTACTTC
TCCCTAAGACTCAAGGAAGGAGCAACAACCCCACTACCAGCACCCAAAGCTGGTGTTCTACT
TAAACTATCCCCTG
>SRR6109208 (circular) ReOrganized
ATACTATAAATCCACCTCTCATTTTATTCACTTCATACATGTTATTACACACTCTGTGCCATC
ATAGTATGTTTTCATACATCCTCCCTTCTTTCACACCCTATGTATATCGTACATTAATGGTGTA
CCCCCCCTCCCCCCTATGTATATCGTGCATTAATGGCGTGCCCCATGCATATAAGCATGTACA
TACTGTGCTTGGCTTTACATGAGGATACTCATTACAAGAACTTATTTCAAGCGATAGTCTATG
AGCATGTATTTCACTTAGTCCAAGAGCTTGATCACCAAGCCTCGAGAAACCAGCAATCCTTG
CGAGTACGTGTACCTCTTCTCGCTCCGGGCCCATAATTTGTGGGGGTTTCTATACTGAAACTA
TACCTGGCATCTGGTTCTTACCTCAGGGCCATGTTAGCGTCAACTCAATCCTACTAACCCTTC
AAATGGGACATCTCGATGGACTAATGACTAATCAGCCCATGATCACACATAACTGTGGTGTC
ATGCATTTGGTATTTTTTAATTTTAGGGGGGGAACTTGCTATGACTCAGCTATGACCGTAAAG
GTCTCGTCGCAGTCAAATCAGCTGTAGCTGGGCTTATTCATCTTTCGAGGCTCCTCATAGACA
CCCATAAGGTGCAATTCAGTCAATGGTCACAGGACATAACACTATAGATCACCCGGACTGGC
GTTACGTGTACGCACGTGTACGCACGTGTACGTACGTGTACGCACGTGTACGTACGTGTACG
CACGTGTACGTACGTGTACGCACGTGTACGTACGTGTACGCACGTGTACGTACGTGTACGCA
CGTGTACGCACGTGTACGCACGTGTACGTACGTGTACGCACGTGTACGTACGTGTACGCATG
TGTACGTACGTGTACGCACGTGTACGTACGTGTACGTACGTGTACGCACGTGTACGTACGTG
TACGCACGTGTACGCACGTGTACGTACGTGTACGCACGTGTACGCACGTGTACGCACGTGTA
CGCGTACGTATTTTAGATACTAAGTTAGCTTAGACAAACCCCCCTTACCCCCCGTAACTTCAA
GAAGCTTACATATACTTATGGATGTCCTGCCAAACCCCAAAAACAAGACTAAATATATGCGC
AAACATGAAGTCACTTACACCTAAACCCATATAATTAAGCTAACCCCCCAGCCAATGTTGCA
ACAACTACGGACATGGGACTCTAAATTTTAATTTATCTATAGATATTTTTCTTTTACTGTGTC
TCCCCAGCATTGATTTTTTAACTATCATTATTCCACACCACCAATTTCCATTGAGCTATTTCAC
ATGAGTTCCAAATCAATTATGTTCATGTAGCTTAACGAATAAAGCAAGGTACTGAAAATGCC
TAGATGGGTCACGCTACCCCATAGACATAAAGGTTTGGTCCTAGCCTTCCTATTAGCCATTA
ACAAGATTACACATGTAAGTCTCCACGCTCCAGTGAAAATGCCCCTTAAGTCCTCTTAGACG
ACCTAAAGGAGCGGGTATCAAGCACACCTTATGGTAGCTCACAACGCCTTGCTTAGCCACAC
CCCCACGGGAAACAGCAGTGATAAAAATTAAGCTATGAACGAAAGTTCGACTAAGCTATGT
TAATACTAGGGTTGGTAAATCTCGTGCCAGCCACCGCGGTCATACGATTAACTCGAGTTAAT
AGGCCTACGGCGTAAAGCGTGTAAAAGAAAAAATCTCCTCTACTAAAGTTAAAGTATGATT
AAGCTGTAAAAAGCTACCATTAATACTAAAATAAACTACGAAAGTGACTTTAAAATTTCTGA
TTACACGATAGCTAGGGCCCAAACTGGGATTAGATACCCCACTATGCCTAGCTCTAAACATA
GATATTTTACTAAACAAAACTATTCGCCAGAGAACTACTAGCAACAGCTTAAAACTCAAAGG
ACTTGGCGGTGCTTTATATCCCCCTAGAGGAGCCTGTTCTGTAATCGATAAACCCCGATAGA
CCTCACCATCCCTTGCTAATTCAGTTTATATACCGCCATCTTCAGCAAACCCTTAAAAGGAAA
AAAAGTAAGCATAACTACCCTACATAAAAAAGTTAGGTCAAGGTGTAACCTATGGGCTGGG

AAGAAATGGGCTACATTTTCTATTCAAGAACAACTTCTACGAAAACTTTTATGAAACTAAAA
GCTAAAGGCGGATTTAGTAGTAAATTAAGAATAGAGAGCTTAATTGAACAGGGCAATGAAG
CACGCACACACCGCCCGTCACCCTCCTCGAGTGATATAATTTAATTATAACCTATTTAAACTA
AGCAAAGCATAAGAGGAGACAAGTCGTAACAAGGTAAGCATACTGGAAAGTGTGCTTGGAT
GAGCCAAAGTGTAGCTTAAACAAAGCGTCTGGCTTACATCCAGAAGATTTCATTAATATATG
ACTACTTTGAACCCAAAGCTAGCCCAAGCAACAATGACTAGTAAAACCATTATGAAACATTC
AAACAAAACATTTAGTAGCATGACTAGAGTATAGGAGATAGAAATTTTTAACTGGAGCTAT
AGAGAGAGTACCGCAAGGGAATGATGAAAGATTACCTAAAGTGATAAACAGCAAAGATTGC
CCCTTCTACCTTTTGTATAATGAGTTAGCTAGAAATAACTTAACAAAGAGAACTTAAGCTAA
GTCCCCCGAAACCAGACGAGCTACCTATGAACAATCCACTGGGATGAACTCATCTATGTTGC
AAAATAGTGAGAAGATCCATAGGTAGAGGTGAAAGGCCTAACGAGCCTGGTGATAGCTGGT
TGCCCAGAATAGAATTTTAGTTCGACTTTAAACCTGCCTACAAAACTAATAATTCTAATGCA
GATTTAAAATATATTCTAAAAAGGTACAGCTTTTTAGAGTTAAGGATACAGCCTTACTTAGA
GAGTAAATATTTATATAAGCCATAGTAGGCCTAGAGGCAGCCATCAATTAAGAAAGCGTTA
AAGCTCAACATCTCTATTAACTTAATACCAAGAATATTTAATCAACTCCTAATGTATTACTGG
GTCAATCTATTTAAATATAGAAGTGATAATGCTAATATGAGTAACAAGAAATATTTCTCCCA
TGCATAAGCTTATAACAGCAACGGATAACCACTGATAGTTAACAACAACATAGAAATAACC
TAATGATAAAACACCTATTAAATCAATTGTTAGTCCAACACAGGCATGCAATCAGGGAAAG
ATTAAAAGAAGTGAAAGGAACTCGGCAAACACAAACCCCGCCTGTTTACCAAAAACATCAC
CTCCAGCATTTCCAGTATTGGAGGCACTGCCTGCCCGGTGACATCAGTTAAACGGCCGCGGT
ATTCTGACCGTGCAAAGGTAGCATAATCATTTGTTCTCTAAATAAGGACTTGTATGAACGGC
CACACGAGGGTTTAACTGTCTCTTACTTCCAATCAGTGAAATTGACCTCCCCGTGAAGAGGC
GGGGATAAGACAATAAGACGAGAAGACCCTATGGAGCTTTAATTAACTAATTCAAAAAGAA
ACTACTAACGACCCAACAGGAATAATATATCTCTTTTATGAATTAGCAATTTAGGTTGGGGC
GACCTCGGAGGACAAAATAGCCTCCGAGTGATTATAAATCTAGACTTACCAGTCAAAATGCT
TAATCACTTATTGATCCAAAAATTCTTTTGATCAACGGAACAAGTTACCCTAGGGATAACAG
CGCAATCCTATCCGAGAGTCCATATCGACAATAGGGTTTACGACCTCGATGTTGGATCAGGA
CATCCTAATGGTGCAGCAGCTATTAAAGGTTCGTTTGTTCAACGATTAAAGTCCTACGTGAT
CTGAGTTCAGACCGGAGCAATCCAGGTCGGTTTCTATCTATTCAAATAATTTCTCCCAGTACG
AAAGGACAAGAGAAATAAGGCCTACTTCTCTGAAGCGCCTTAAGACCAATAGATGAATTTA
TCTAAATCTAGTAAATCTAACTCCAATATTGCCCAAGAGACAGGGCTTTGTTAGGGTGGCAG
AGCCCGGTAATTGTGCAAAACTTAAACTCTTGTGTCCAGAGGTTCAATTCCTCTCCCTAGCAT
ATGTTTATAATTAACATCTTCTCACTAATTGTACCTATTCTTCTTGCTGTAGCCTTTCTGACTC
TAGTAGAACGAAAAGTACTAGGCTACATACAGCTTCGAAAAGGCCCAAATATTGTAGGTCC
ATATGGTCTCCTACAACCAATCGCAGATGCCGTAAAACTCTTTACTAAAGAACCTTTACGAC
CACTTACGTCATCCATAACTATATTTATTATAGCCCCTATTCTAGCCTTAACACTAGCTCTGA
CCATGTGAGTCCCCTTACCAATACCGCACCCCCTTATTAACATAAACCTAGGAGTACTATTC
ATACTAGCTATATCAAGTCTAGCTGTATACTCTATTCTCTGATCTGGATGAGCCTCAAACTCA
AAATATGCCCTAATTGGAGCCCTACGAGCCGTAGCTCAAACGATCTCATATGAAGTCACACT
AGCCATTATCCTTTTATCCGTTCTACTAATAAATGGCTCATTTACGCTATCCACGCTAATCAT
TACCCAAGAACATCTTTGATTAATCTTCCCTGCATGACCACTAGCTATAATATGATTTATTTC
TACCCTAGCAGAAACAAATCGAGCCCCCTTTGACCTAACAGAAGGAGAATCAGAACTAGTC
TCTGGGTTTAATGTCGAATATGCAGCAGGTCCATTTGCCCTGTTCTTCCTAGCAGAATATGCC
AACATCATCATAATAAATGCTCTTACAACTATTCTATTCTTCGGAGCATTTCACAGCCCCTAC
ATACCAGAATTATACATTGTTAATTTTACCGTAAAAACTTTACTCCTCACAATCTCTTTTCTA
TGAATCCGAGCATCATATCCTCGATTCCGATACGACCAACTAATACACCTACTATGAAAAAA
CTTTCTACCTCTCACACTAGCTCTATGTATATGACATGTAACTCTACCTATTATTACAGCAAG
CATTCCACCTCAAACGTAAGAAATATGTCTGACAAAAGAATTACTTTGATAGAGTAAGCCAT
AGGGGTTTAAGTCCCCTTATTTCTAGAATCATAGGAATCGAACCTAATCTTAAGAACTCAAA
AATCTTCGTGCTACCTAACTACACCATATCCTAAAGTAAGGTCAGCTAAATAAGCTATCGGG
CCCATACCCCGAAAATGTTGGTTTATCCCCTTCCCATACTAATAAAACCAGCCATCCTTATTA

CCATTATATCCACCGTTGTCTTAGGAACTATAATTGTTCTATTTAGCTCCCACTGATTCATAA
TCTGAGTTGGCTTTGAAATAAATATACTAGCCATTATCCCAATTCTAATAAAAAAATATAAC
CCACGAGCCATAGAAGCTTCCACAAAATATTTCTAACACAAGCTACAGCATCCATGCTTCT
AATGCTAAGCATCATCATCAATCTACTATGCTCAGGACACTGGACAGTTTCAACAATTCCCA
ATCCAGTAGCATCTACCATAATTACTATTGCTCTAACAATAAAACTTGGCCTATCACCATTTC
ACTTTTGAGTCCCAGAAGTTACACAAGGCATTTCCTTATCTTCAGGAATAATCCTATTAACAT
GACAAAAAATTGCACCTTTATCCATTCTATACCAAATTTCACCATCCGTTAACTCAAATCTCT
TACTAATAATAGCTATCACATCCATACTGGTAGGAGGCTGAGGAGGATTAAATCAAACCCA
ACTCCGAAAAATCTTGGCATACTCCTCAATTACCCATATAGGCTGAATGGCTGCTATTATAG
TATATAATCCTACCCTAGCAATTCTTAACCTAACAATTTACATCATAATAACTCTAGGAACAT
TTATACTATTCATACATAGCTCATCTACAACAACACTATCACTATCTTATACATGAAATAAAT
TCCCATTAATAGCCCCACTAATCTTAATACTTATACTATCACTAGGAGGCCTCCCTCCTCTCT
CAGGCTTTATCCCCAAATGAATAATTATTCAAGAATTAACAAAAAACGACATAATTATTATA
CCTACACTTATAGCCATTACAGCCCTACTAAACCTATATTTTTACACACGCCTAACATATACT
ACAGCACTAACTATATTTCCCGCAACAAATAATATAAAAATAAAATGACAATTCAAAACCA
CAAAAAAGACAACTCTCCTAGCCCCTCTAATCGTGACATCAACTATACTACTACCACTAACC
CCAATACTAGCAGCTCTTGATTAGAGATTTAGGCTAAACAGACCAAGGGCCTTCAAAGCCTC
AAGTAAGTGAAAATCCACTTAATCTCTGCAATATTTAAGGACTGCAAGAATCTATCTCACAT
CAACTGAACGCAAAACAATCGCTTTAATTAAGCTAAGCCCTTCTAGATCGGTGGGCTTTTAC
CCCACAAAACTTTAGTTAACAGCTAAAAACCCTAGACAACTGGCTTCAATCTACTTCTCCCG
CCGCGTAGGAAAAAAAGGCGGGAGAAGCCCCGGCAGAATTGAAGCTGCTTCTTTGAATTTG
CAATTCAATATGATTACTCACCACAGAGCCTGGTAAAAAGAGGACTTAACCCCCATTCTTAG
ATTTACAGTCTAGTGCTTTTATCAGCCATTTTACCTATGTTCATTAACCGATGACTGTTTTCCA
CCAACCACAAGGATATTGGCACTCTTTATCTTCTGTTTGGCGCATGAGCTGGTATAACGGGC
ACTGCTCTTAGCCTTCTAATCCGTGCTGAATTAGGTCAGCCTGGAGCTCTGTTAGGAGATGA
CCAAATTTACAATGTAGTTGTAACTGCCCACGCATTCGTAATAATCTTCTTTATAGTTATGCC
TATTATGATCGGAGGCTTTGGAAATTGACTAGTACCTTTGATAATTGGTGCGCCTGATATAG
CATTTCCCCGAATAAACAATATGAGTTTCTGATTACTACCTCCATCCTTTCTTCTCCTTCTGGC
TTCTTCCATAGTAGAAGCAGGCGCAGGGACTGGATGAACTGTATACCCCCCTCTAGCAGGTA
ACTTAGCCCATGCAGGAGCATCAGTAGACCTAACAATCTTTTCTCTACATCTGGCAGGGGTT
TCTTCTATTTTAGGGGCTATTAACTTTATTACCACTATCATCAACATGAAACCCCCTGCAATA
TCTCAATATCAAACTCCCCTATTCGTGTGATCAGTACTTATCACAGCAGTACTTCTCCTTCTA
TCTTTACCAGTCTTAGCAGCCGGAATTACTATACTACTTACAGATCGAAATCTCAATACTACT
TTCTTCGACCCGGCCGGAGGAGGGGATCCCATTCTATATCAACATCTATTTTGATTCTTTGGC
CATCCTGAAGTCTATATTCTAATCCTCCCTGGGTTTGGAATAATTTCACACATTGTCACATAC
TATTCAGGAAAGAAAGAGCCCTTTGGTTATATGGGAATAGTGTGGGCAATAATGTCAATTGG
GTTCTTAGGATTTATTGTATGGGCCCATCATATATTTACTGTAGGCATAGATGTTGATACACG
AGCCTATTTTACCTCGGCCACTATAATTATTGCAATCCCGACAGGAGTTAAAGTATTCAGCT
GACTAGCTACCCTGCATGGAGGAAATATTAAGTGATCCCCTGCCATAATGTGAGCCCTAGGT
TTCATTTTTCTATTTACAGTAGGGGGTCTTACAGGCATTGTTCTAGCTAACTCATCTCTGGAC
ATTGTCCTCCATGACACATACTACGTAGTAGCACACTTCCACTATGTACTATCAATAGGGGC
CGTGTTCGCTATTATAGGAGGCTTCGTCCATTGATTCCCATTATTTTCGGGCTATACACTTAA
TAATACATGAGCAAAAATTCACTTCGTAATTATATTTGTTGGAGTCAATATAACATTCTTCCC
CCAGCACTTCCTAGGTCTATCAGGTATGCCTCGACGATATTCTGATTATCCAGACGCCTATAC
AGCATGAAACACAGTGTCTTCAATGGGTTCATTCATTTCACTAACAGCAGTTATGTTAATAA
TTTTCATAATTTGAGAAGCCTTCGCATCTAAACGAGAAGTGGCAGTGATTGAGCTTACTACA
ACTAATATTGAATGGCTCCACGGATGTCCTCCCCCATACCATACATTCGAAGAGCCCGCCTA
CGTCATACTGAAATAAGAAAGGAAGGAATCGAACCCTCTAGAATTGGTTTCAAGCCAACAT
CATAACCACTATGTCTCTCTCAATAAAGAGGTATTAGTAAAAATTACATAACTTTGTCAAAG
TTGAATTATAGGTGAAAATCCTTTATATCTCTATGGCGCATCCCTTTCAAACAGGCCTTCAAG
ATGCAACTTCCCCCATCATGGAAGAACTCCTACACTTCCATGATCATACACTAATAATTGTAT

TCTTAATTAGTTCCTTAGTTCTCTATATCATTTCAATTATATTAACTACTAAATTAACACATAC
AAATACAATAGACGCACAAGAAGTGGAGACAGTATGAACTATTCTACCAGCTATCATCTTA
ATTATAATTGCACTTCCATCACTACGAATTCTTTATATGATAGATGAAATTAATAACCCATCA
TTAACTGTAAAGACTATGGGGCATCAATGATACTGAAGCTATGAGTATACAGATTATGAAGA
CTTGAGCTTCGATTCTTATATAACCCCAACACAAGAATTAAAGCCCGGAGAGCTGCGATTAC
TAGAGGTAGATAATCGAGTAGTACTACCCATAGAAATAACAATTCGCATATTAATTTCCTCA
GAAGATGTGCTACATTCGTGAGCTGTCCCATCTCTAGGACTAAAAACTGATGCAATCCCGGG
GCGATTAAATCAAACAACTCTTATGGCCATGCGACCAGGACTATATTATGGCCAATGCTCAG
AGATTTGCGGTTCTAATCATAGCTTTATACCCATTGTACTTGAAATAGTTCCGCTGTCTTACT
TTGAAAAATGATCCGCTTCAATACTATAAAATCATTAAGAAGCTTTATAGCGTTAACCTTTTA
AGTTAAAGACTGAGAGTGCAAGTCTCTCCTTAATGGAAATGCCACAATTAGACACATCCACA
TGATTTATTACAATCCTATCCATGATCTTAACACTATTTATTATATTTCAGTTAAAAGTCTCA
AAACACAACTATCCTGAGTTTCCTGAATTAAAAACAACCCCACTATCAAAAAAACCTATGCC
TTGAGAAGAAAAATGAACGAAAATCTGTTCGCCTCTTTTACTACCCCAATGATAATAGGAGT
ACCAATTGTGGTTCTGATTATTATTTTCCCAAGTATCTTATTTCCCTCACCTAATCGATTAATC
AATAACCGCCTAGCTACTGTCCAACAATGATTAATTCAATTAGTATCTAAGCATATACTGTCT
ATTCACAATTATAAAGGACAAACCTGAGCGCTAATACTAATGTCATTAATTTTATTTATTGGC
TCAACTAACCTTTTAGGCCTATTACCACATTCATTCACACCTACTACACAACTATCAATAAAT
TTAGGAATGGCCATTCCTCTGTGAGCAGGTACAGTAGTTATAGGATTTCGACACAAAACTAA
GGCATCTCTGGCCCACTTTCTACCTCAAGGAACACCTCTTCCTCTGATCCCAGTGTTAGTAAT
TATCGAGACAATTAGTCTGTTTATTCAACCTGTGGCCCTAGCTGTACGACTGACCGCTAATAT
TACTGCAGGCCATCTACTTATTCATTTAATCGGAGGGGCTACCCTAGTTTTAATAAACATTAA
CCCTGTAACAGCCCTAATTACCTTTATTATTCTTATTCTACTTACTATTCTCGAATTAGCTGTA
GCTCTTATCCAAGCCTATGTTTTCACCCTACTAGTTAGTCTATACTTACATGATAATACCTAA
TGATCCACCAAACACATGCATATCATATAGTTAACCCAAGCCCATGGCCACTTACAGGAGCT
CTCTCGGCTCTTCTTATAACATCAGGACTAATTATATGATTTCACTTTAACTCGGTATACTTA
CTAATACTAGGCCTCACCACCAACATACTTACCATGTACCAATGATGACGAGATATCGTCCG
GGAAAGTACCTTCCAAGGCCATCACACCCCAATCGTCCAAAAAGGACTACGATATGGAATA
GTCTTATTTATTGTATCAGAAGTATTTTTCTTCGCGGGATTCTTTTGGGCCTTCTACCACTCAA
GCCTAGCACCTACTCCTGAATTAGGAGCATGTTGACCACCTACAGGTATCATCCCCTTAAAT
CCGCTAGAAGTTCCACTACTTAACACCTCTGTACTTTTAGCATCTGGGGTATCTATCACTTGA
GCCCATCATAGTCTAATAGAAGGAGACCGTAAACATATGCTCCAAGCCCTATTTATTACTAT
TTCACTAGGAGTTTATTTTACACTTCTACAAGCCTCAGAATACTATGAGACATCCTTTACTAT
TTCAGATGGAGTGTATGGTTCAACCTTCTTTATAGCAACTGGATTTCATGGGCTTCATGTAAT
TATTGGTTCAACTTTTCTTACTGTGTGTTTTCTACGACAACTGCACTTTCACTTTACATCCAAC
CATCACTTTGGCTTTGAAGCGGCAGCATGATACTGACACTTCGTAGACGTTGTATGGCTTTTC
CTTTATGTTTCTATTTACTGATGAGGATCCTACTTCTTTAGTATTAACTAGTACGATTGACTTC
CAATCAATAGGCTCTGGTACAAGCCAGAAAGAAGTAATAAACCTAATTCTAGCACTTCTAAC
CAACGTATTATTAGCCTCTCTGCTCGTTCTTATTGCATTCTGACTGCCACAACTAAATATCTA
TGCAGAAAAAGTAAGTCCTTATGAATGTGGATTTGATCCTGTAGGATCAGCACGCCTTCCTT
TCTCTATAAAATTTTTTCTAGTAGCTATTACATTCCTTTTATTCGACTTAGAAATTGCTCTACT
CCTCCCGCTTCCTTGAGCATTACAGACTAACAACTTAATAACTATACTTATTATAGCTCTATT
ACTCATTTCCCTATTAGCCGCAAGCCTAGCTTATGAGTGAACCGAAAAGGGGCTAGAATGGG
CCGAATATGATAATTAGTTTAAACTAAAATAAGTGATTTCGACTCACTAGATTATGACTAAC
ATCATAATTATCAAATGTCTGTGGTCTATATTAATCTCTTTCTAGCCTTTACTATATCCTTAAT
AGGGCTACTCATCTACCGATCCCATTTAATATCCTCCCTATTATGCTTAGAAGGCATGATGCT
GTCACTATTTGTTATACTAACAGTAACAATCCTAAGTAACCACTTCACACTAGCCAGTATGA
CTCCAATCATTCTATTAGTATTCGCTGCCTGTGAAGCAGCGTTGGGGCTTTCTCTGCTAGTAA
TAGTCTCTAATACCTATGGAACTGACTACGTACAAAATCTTAACCTCCTACAATGCTAAAAA
TTATCCTCCCTACCATAATACTAGTACCCCTTACATGAATATCAAAGCCTAACATAATCTGAA
TTAATACAACAACCTATAGTCTACTCATCAGCCTTATTAGTCTGACATATCTCAATCAACTCA

GTGATAATAGTCTAAACTTCTCATTAACATTCTTTACCGACTCCCTATCAGCCCCCCTGTTAG
TTCTCACAACATGACTACTGCCCTTAATACTTATAGCTAGTCAATTTCACCTGTCAAAAGAGA
CCCTGACCCGGAAAAAATTATATATCACAATACTAATTCTCCTGCAACTATTCTTAATTATAA
CATTTTCTGCTACTGAGTTAATCATATTCTATATTTATTTGAAGCTACCCTGGTACCGACTTT
AGTTATCATTACTCGATGAGGAAACCAAACAGAACGACTAAACGCGGGCCTCTATTTTTTAT
TCTACACCTTAGTAGGATCCTTGCCCTTACTAGTTGCACTGTTATATGCCCAAAACAGCTTAG
GATCACTAAATTTTCTCATAGTACAGTACTGAACTCAGCCCTTGCCAAATTCCTGATCTAACA
TTTTCATGTGGCTGGCATGTATAATAGCATTCATAGTAAAAATACCGCTATATGGCTTACACC
TATGACTACCCAAAGCACATGTAGAAGCTCCTATTGCAGGATCCATAGTACTTGCTGCAGTA
CTTCTAAAACTAGGGGGATACGGTATAATGCGGATTACAACAATACTCAACCCTCTAACAAA
TTTTATAGCCTACCCCTTCATAATGCTCTCACTATGAGGAATAATCATAACAAGTTCTATTTG
TTTACGCCAGACAGACCTAAAGTCCCTAATTGCATACTCCTCTGTTAGTCACATAGCCCTAGT
CATTGTAGCAATTCTAATCCAATCCCCATGAAGCTACATAGGAGCTACTGCTCTAATAATCG
CCCATGGGTTAACATCTTCTATGCTATTCTGTCTTGCAAACTCTAACTACGAGCGAACCCATA
GCCGAACTATAATTCTCGCACGGGGCTTGCAAACGCTTCTTCCACTTATAGCAGCTTGATGA
CTATTAGCAGGCCTAACTAACTTAGCTCTTCCTCCTACAATTAATCTGATTGGGGAACTGTTT
GTAGTGATAGCCACATTTTCATGATCCAATATTACCATTCTCTTAATAGGAGTTAACATTACC
ATTACTGCTCTCTACTCTCTATATATACTAATTTCTACACAGCGAGGCAAGTATACAGACCAC
ATCAAAAATATTAAGCCATCTTATACACGAGAAAATGCCTTAATAGCCTTACATTTACTACC
CCTTTTACTTCTATCTCTTAACCCTAAAGTAGTTCTAGGCTTTATTTACTGTAAATATAGTTTA
ACAAAAACATTAGATTGTGAATCTAGTAATAAAAGCTTAAATCTTTTTATTTACCGAAAAAG
CATGCAAGAACTGCTAATTCATGCACCCATGCATAAAATCATGGCTTTTTCAACTTTTAGGG
GATAGAAGTAATCCATTGGTCTTAGGAGCCAAAAAATTGGTGCAACTCCAAATAAAAGTAA
TTAACCTACTTACCTCCTTCACCATTACAACTCTATTTATATTAATCATGCCCATTATCCTAAC
CTGTACCCCAGCATATGAAAATAAACTTTACCCGCAATATGTAAAAACCACAGTCTCTTATG
CCTTCACAATTAGCATAATTCCCATAGTAATATTCATCCACTCCGGACAAGAAGCAATCATC
TCAAACTGACACTGAATAACCATCCAAACCCTAAAACTCAGTCTAAGCTTCAAATTAGATTA
CTTTTCAATAATCTTCGTACCAGTGGCCCTATTCGTCACATGGTCAATTATAGAATTCTCTAT
ATGATATATACACGCAGATCCTAACATTAACCGATTCTTTAAATATCTACTTATATTCCTCGT
CACGATAATAATTCTAGTAACCGCAAATAATCTATTTCAGCTATTCATCGGCTGAGAGGGAG
TAGGAATCATATCATTTCTCCTCATTGGATGATGATATGGACGAACAGATGCTAATACGGCT
GCCCTACAAGCAGTACTATACAACCGCATTGGAGATGTTGGTTTTATCATGGCCATAGCTTG
ATTCTTACTCAATACTAATGCATGGGACTTCCAACAAATCTTTATAATCGAACATAAAAACC
TCAATATTCCACTGGCAGGACTACTTCTAGCAGCTACCGGAAAGTCTGCCCAATTTGGACTT
CATCCATGACTCCCATCAGCCATAGAAGGCCCCACTCCTGTCTCAGCACTACTTCACTCTAGC
ACAATAGTAGTAGCAGGCGTATTTCTTCTAGTCCGGTTTCACCCCTTAATAGAACATAACAA
GACAATTCAAACGGTTACCCTATGCTTAGGGGCAATAACAACCTTGTTTACAGCAATCTGCG
CCCTTACCCAAAATGATATCAAAAAAATTATTGCCTTCTCCACCTCGAGCCAACTAGGACTA
ATAATTGTAACAATTGGTATTAATCAGCCTCATCTGGCCTTTCTCCATATCTGCACGCACGCA
TTCTTCAAGGCCATGCTATTTCTATGCTCCGGATCTATTATTCATAGTCTAAACAACGAACAA
GATATTCGAAAGATAGGAGGGCTATTTAAAAGTCTACCATTTACCACTACTGCTCTAATCAT
CGGAACTCTAGCACTTACAGGAATGCCCTTCTTAACAGGATTTTACTCCAAAGACTTAATTA
TTGAAACCGCCAATACGTCATACACCAACGCCTGAGCCTTACTAACAACCCTTATCGCCACA
TCCATAACTGCCGCCTACAGTACTCGAATTGTATTTTTTACACTTCTAGGACAACCTCGCTAT
AGTCCCATTATCACCATTAACGAGAATAACCAGTTCCTGACTAACTCTGTCAAACGATTGCT
ACTAGGAAGTATCTTTGCGGGATTCTTAATCTCTCATAACATTACACCCACCACTGTCCCACA
AATAACCATGCCCTGTCACCTAAAAATGATAGCCCTTACAGTAACAATCCTAGGCTTTATTC
TAGCACTAGAACTTAACCTAGCCATACAAAATCTCAAATTTGACTACCCGTTAAGCACGTTC
AAATTTTCAAATCAACTAGGATATTTCCCTACTACTCTGCATCGTCTCGCACCAATAATAGGT
CTATCAATAAGCCAAAAAACAGCATCCATACTGCTAGATGCAATCTGACTAGAGAATGTTCT
ACCAAAGTCCATCTCCTACTTTCAAATAAAATCCTCAATCACAGTCTCCAACCAAAAGGGAC

TAATTAAACTATACTTTCTCTCCTTTATAATTACACTAATCTTAAGTCTCTTTATACTTAATTT
CCACGAGTGACTTCCATAATTACCAATACACCAATAAGGAGAGATCATCCAGTAACAATAA
CTAATCAAGTACCATAACTATATAAAGCTGCAATTCCCATAGCCTCTTCACTAAAAAATCCT
GAATCCCCTGTATCATAAATCACCCAATCTCCTATTCCATTAAACTTAAATACTACCTCGGCT
TCATCATCCTTTAAAATATAACAAACAGCCAGTAACTCAGACAACAGACCTACAATAAATGC
ACCTAGTACAGCCTTATTAGAAAGTCAAACCTCAGGATATTGCTCAGTGGCTATGGCCGTAG
TGTACCCGAACACTACAAGTATACCCCCTAAATAAATTAAAAAGACCATTAAACCCAAAAA
AGATCCTCCAAAGCTTAACACAATACCACACCCTACACCCCCACTAACAATCAAGACTAAAC
CTCCGTAGATAGGAGAGGGCTTAGAAGAAAACCCCGCAAAACTAACTACAAAAACGATACT
TAAAATAGATACAATGTATGTTATCATCATTCTTACATGGAATCTAACCATGACCAATGACA
TGAAAAATCATCGTTGTATTTCAACTATAAGAACCCCAATGATCAACATCCGAAAAACTCAT
CCATTAGTTAAAATTATCAACAACTCATTCATTGACCTTCCAACACCATCAAACATTTCAACA
TGATGGAACTTTGGGTCCCTGTTAGGAGTGTGTCTGATCTTGCAAATCTTAACAGGCTTATTT
CTAGCCATACACTATACATCAGATACAGCTACAGCCTTTTCATCAGTCGCACACATTTGTCG
AGACGTCAACTATGGTTGATTTATCCGATATATACATGCCAATGGGGCCTCTATATTTTTTAT
CTGCCTATTTATACACGTAGGGCGAGGCTTATACTATGGATCATACCTATTTCCAGAGACAT
GGAATATCGGAATTATTCTCCTACTTACAGTTATAGCCACAGCATTCATAGGATATGTACTA
CCTTGAGGACAAATATCCTTCTGAGGAGCAACCGTCATTACTAACCTACTATCAGCAATTCC
TTACATTGGCACTAATCTAGTGGAGTGAATCTGAGGGGGTTTCTCCGTAGATAAAGCAACAC
TAACCCGATTTTTTGCTTTTCACTTTATCCTTCCATTTATCATCTCAGCACTAGCAATAGTCCA
TCTATTATTCCTTCACGAAACAGGATCTAATAACCCCTCCGGAATTCCATCTGACCCAGACA
AAATCCCATTTTACCCCTATCATACAATTAAAGACATCCTAGGCGTCCTATTTCTTGTCCTCG
CCTTAATAACCCTGGCTTTATTCTCACCAGACCTGTTAGGAGACCCTGATAACTATACCCCTG
CAAATCCACTAAGTACCCCGCCACATATTAAGCCTGAATGGTACTTTCTATTTGCCTACGCTA
TCCTGCGATCTATCCCTAATAAACTAGGAGGGGTGCTAGCTCTAATCTTCTCTATTCTAATTC
TAACTATTATTCCACTATTACATACATCCAAACAACGAAGCATGATATTCCGACCTCTAAGT
CAATGCTTATTCTGACTCCTAGTAGCAGACCTACTCACACTAACATGAATTGGAGGACAGCC
AGTAGAACACCCCTTCATTATTATTGGGCAATTGGCCTCTATTCTCTACTTTACAATTCTTCT
AGTACTTATACCTATCACTAGCATTATTGAGAATAGCCTCTCAAAATGAAGAGTCTTTGTAG
TATATTAATTACTTTGGTCTTGTAAACCAAAAACGGAGATTCCTCTACTTCTCCCTAAGACTC
AAGGAAGGAGCAACAACCCCACTACCAGCACCCAAAGCTGGTGTTCTACTTAAACTATCCCC
TG
>SRR6109209 (circular) ReOrganized
ATACTATAAATCCACCTCTCATTTTATTCACTTCATACATGTTATTACACACTCTGTGCCATC
ATAGTATGTTTTCATACATCCTCCCTTCTTTCACACCCTATGTATATCGTACATTAATGGTGTA
CCCCCCCCTCCCCCCTATGTATATCGTGCATTAATGGCGTGCCCCATGCATATAAGCATGTAC
ATACTGTGCTTGGCTTTACATGAGGATACTCATTACAAGAACTTATTTCAAGCGATAGTCTAT
GAGCATGTATTTCACTTAGTCCAAGAGCTTGATCACCAAGCCTCGAGAAACCAGCAATCCTT
GCGAGTACGTGTACCTCTTCTCGCTCCGGGCCCATAATTTGTGGGGGTTTCTATACTGAAACT
ATACCTGGCATCTGGTTCTTACCTCAGGGCCATGTTAGCGTCAACTCAATCCTACTAACCCTT
CAAATGGGACATCTCGATGGACTAATGACTAATCAGCCCATGATCACACATAACTGTGGTGT
CATGCATTTGGTATTTTTTAATTTTAGGGGGGGAACTTGCTATGACTCAGCTATGACCGTAAA
GGTCTCGTCGCAGTCAAATCAGCTGTAGCTGGGCTTATTCATCTTTCGAGGCTCCTCATAGAC
ACCCATAAGGTGCAATTCAGTCAATGGTCACAGGACATAACACTATAGATCACCCGGACTG
GCGTTACGTGTACGCACGTGTACGCACGTGTACGTACGTGTACGCACGTGTACGTACGTGTA
CGCATGTGTACGTACGTGTACGCACGTGTACGTACGTGTACGTACGTGTACGCACGTGTACG
CACGTGTACGCACGTGTACGCACGTGTACGCACGTGTACGTACGTGTACGCACGTGTACGCA
CGTGTACGCACGTGTACGCGTACGTATTTTAGATACTAAGTTAGCTTAGACAAACCCCCCTT
ACCCCCCGTAACTTCAAGAAGCTTACATATACTTATGGATGTCCTGCCAAACCCCAAAAACA
AGACTAAATATATGCGCAAACATGAAGTCACTTACACCTAAACCCATATAATTAAGCTAACC
CCCCAGCCAATGTTGCAACAACTACGGACATGGGACTCTAAATTTTAATTTATCTATAGATA

TTTTTCTTTTACTGTGTCTCCCCAGCATTGATTTTTTAACTATCATTATTCCACACCACCAATT
TCCATTGAGCTATTTCACATGAGTTCCAAATCAATTATGTTCATGTAGCTTAACGAATAAAGC
AAGGTACTGAAAATGCCTAGATGGGTCACGCTACCCCATAGACATAAAGGTTTGGTCCTAGC
CTTCCTATTAGCCATTAACAAGATTACACATGTAAGTCTCCACGCTCCAGTGAAAATGCCCC
TTAAGTCCTCTTAGACGACCTAAAGGAGCGGGTATCAAGCACACCTTATGGTAGCTCACAAC
GCCTTGCTTAGCCACACCCCCACGGGAAACAGCAGTGATAAAAATTAAGCTATGAACGAAA
GTTCGACTAAGCTATGTTAATACTAGGGTTGGTAAATCTCGTGCCAGCCACCGCGGTCATAC
GATTAACTCGAGTTAATAGGCCTACGGCGTAAAGCGTGTAAAAGAAAAAATCTCCTCTACTA
AAGTTAAAGTATGATTAAGCTGTAAAAAGCTACCATTAATACTAAAATAAACTACGAAAGT
GACTTTAAAATTTCTGATTACACGATAGCTAGGGCCCAAACTGGGATTAGATACCCCACTAT
GCCTAGCTCTAAACATAGATATTTTACTAAACAAAACTATTCGCCAGAGAACTACTAGCAAC
AGCTTAAAACTCAAAGGACTTGGCGGTGCTTTATATCCCCCTAGAGGAGCCTGTTCTGTAAT
CGATAAACCCCGATAGACCTCACCATCCCTTGCTAATTCAGTTTATATACCGCCATCTTCAGC
AAACCCTTAAAAGGAAAAAAAGTAAGCATAACTACCCTACATAAAAAAGTTAGGTCAAGGT
GTAACCTATGGGCTGGGAAGAAATGGGCTACATTTTCTATTCAAGAACAACTTCTACGAAAA
CTTTTATGAAACTAAAAGCTAAAGGCGGATTTAGTAGTAAATTAAGAATAGAGAGCTTAATT
GAACAGGGCAATGAAGCACGCACACACCGCCCGTCACCCTCCTCGAGTGATATAATTTAATT
ATAACCTATTTAAACTAAGCAAAGCATAAGAGGAGACAAGTCGTAACAAGGTAAGCATACT
GGAAAGTGTGCTTGGATGAGCCAAAGTGTAGCTTAAACAAAGCGTCTGGCTTACATCCAGA
AGATTTCATTAATATATGACTACTTTGAACCCAAAGCTAGCCCAAGCAACAATGACTAGTAA
AACCATTATGAAACATTCAAACAAAACATTTAGTAGCATGACTAGAGTATAGGAGATAGAA
ATTTTTAACTGGAGCTATAGAGAGAGTACCGCAAGGGAATGATGAAAGATTACCTAAAGTG
ATAAACAGCAAAGATTGCCCCTTCTACCTTTTGTATAATGAGTTAGCTAGAAATAACTTAAC
AAAGAGAACTTAAGCTAAGTCCCCCGAAACCAGACGAGCTACCTATGAACAATCCACTGGG
ATGAACTCATCTATGTTGCAAAATAGTGAGAAGATCCATAGGTAGAGGTGAAAGGCCTAAC
GAGCCTGGTGATAGCTGGTTGCCCAGAATAGAATTTTAGTTCGACTTTAAACCTGCCTACAA
AACTAATAATTCTAATGCAGATTTAAAATATATTCTAAAAAGGTACAGCTTTTTAGAGTTAA
GGATACAGCCTTACTTAGAGAGTAAATATTTATATAAGCCATAGTAGGCCTAGAGGCAGCCA
TCAATTAAGAAAGCGTTAAAGCTCAACATCTCTATTAACTTAATACCAAGAATATTTAATCA
ACTCCTAATGTATTACTGGGTCAATCTATTTAAATATAGAAGTGATAATGCTAATATGAGTA
ACAAGAAATATTTCTCCCATGCATAAGCTTATAACAGCAACGGATAACCACTGATAGTTAAC
AACAACATAGAAATAACCTAATGATAAAACACCTATTAAATCAATTGTTAGTCCAACACAG
GCATGCAATCAGGGAAAGATTAAAAGAAGTGAAAGGAACTCGGCAAACACAAACCCCGCCT
GTTTACCAAAAACATCACCTCCAGCATTTCCAGTATTGGAGGCACTGCCTGCCCGGTGACAT
CAGTTAAACGGCCGCGGTATTCTGACCGTGCAAAGGTAGCATAATCATTTGTTCTCTAAATA
AGGACTTGTATGAACGGCCACACGAGGGTTTAACTGTCTCTTACTTCCAATCAGTGAAATTG
ACCTCCCCGTGAAGAGGCGGGGATAAGACAATAAGACGAGAAGACCCTATGGAGCTTTAAT
TAACTAATTCAAAAAGAAACTACTAACGACCCAACAGGAATAATATATCTCTTTTATGAATT
AGCAATTTAGGTTGGGGCGACCTCGGAGGACAAAATAGCCTCCGAGTGATTATAAATCTAG
ACTTACCAGTCAAAATGCTTAATCACTTATTGATCCAAAAATTCTTTTGATCAACGGAACAA
GTTACCCTAGGGATAACAGCGCAATCCTATCCGAGAGTCCATATCGACAATAGGGTTTACGA
CCTCGATGTTGGATCAGGACATCCTAATGGTGCAGCAGCTATTAAAGGTTCGTTTGTTCAAC
GATTAAAGTCCTACGTGATCTGAGTTCAGACCGGAGCAATCCAGGTCGGTTTCTATCTATTC
AAATAATTTCTCCCAGTACGAAAGGACAAGAGAAATAAGGCCTACTTCTCTGAAGCGCCTTA
AGACCAATAGATGAATTTATCTAAATCTAGTAAATCTAACTCCAATATTGCCCAAGAGACAG
GGCTTTGTTAGGGTGGCAGAGCCCGGTAATTGTGCAAAACTTAAACTCTTGTGTCCAGAGGT
TCAATTCCTCTCCCTAGCATATGTTTATAATTAACATCTTCTCACTAATTGTACCTATTCTTCT
TGCTGTAGCCTTTCTGACTCTAGTAGAACGAAAAGTACTAGGCTACATACAGCTTCGAAAAG
GCCCAAATATTGTAGGTCCATATGGTCTCCTACAACCAATCGCAGATGCCGTAAAACTCTTT
ACTAAAGAACCTTTACGACCACTTACGTCATCCATAACTATATTTATTATAGCCCCTATTCTA
GCCTTAACACTAGCTCTGACCATGTGAGTCCCCTTACCAATACCGCACCCCCTTATTAACATA

AACCTAGGAGTACTATTCATACTAGCTATATCAAGTCTAGCTGTATACTCTATTCTCTGATCT
GGATGAGCCTCAAACTCAAAATATGCCCTAATTGGAGCCCTACGAGCCGTAGCTCAAACGAT
CTCATATGAAGTCACACTAGCCATTATCCTTTTATCCGTTCTACTAATAAATGGCTCATTTAC
GCTATCCACGCTAATCATTACCCAAGAACATCTTTGATTAATCTTCCCTGCATGACCACTAGC
TATAATATGATTTATTTCTACCCTAGCAGAAACAAATCGAGCCCCCTTTGACCTAACAGAAG
GAGAATCAGAACTAGTCTCTGGGTTTAATGTCGAATATGCAGCAGGTCCATTTGCCCTGTTC
TTCCTAGCAGAATATGCCAACATCATCATAATAAATGCTCTTACAACTATTCTATTCTTCGGA
GCATTTCACAGCCCCTACATACCAGAATTATACATTGTTAATTTTACCGTAAAAACTTTACTC
CTCACAATCTCTTTTCTATGAATCCGAGCATCATATCCTCGATTCCGATACGACCAACTAATA
CACCTACTATGAAAAAACTTTCTACCTCTCACACTAGCTCTATGTATATGACATGTAACTCTA
CCTATTATTACAGCAAGCATTCCACCTCAAACGTAAGAAATATGTCTGACAAAAGAATTACT
TTGATAGAGTAAGCCATAGGGGTTTAAGTCCCCTTATTTCTAGAATCATAGGAATCGAACCT
AATCTTAAGAACTCAAAAATCTTCGTGCTACCTAACTACACCATATCCTAAAGTAAGGTCAG
CTAAATAAGCTATCGGGCCCATACCCCGAAAATGTTGGTTTATCCCCTTCCCATACTAATAA
AACCAGCCATCCTTATTACCATTATATCCACCGTTGTCTTAGGAACTATAATTGTTCTATTTA
GCTCCCACTGATTCATAATCTGAGTTGGCTTTGAAATAAATATACTAGCCATTATCCCAATTC
TAATAAAAAAATATAACCCACGAGCCATAGAAGCTTCCACAAAATATTTTCTAACACAAGCT
ACAGCATCCATGCTTCTAATGCTAAGCATCATCATCAATCTACTATGCTCAGGACACTGGAC
AGTTTCAACAATTCCCAATCCAGTAGCATCTACCATAATTACTATTGCTCTAACAATAAAACT
TGGCCTATCACCATTTCACTTTTGAGTCCCAGAAGTTACACAAGGCATTTCCTTATCTTCAGG
AATAATCCTATTAACATGACAAAAAATTGCACCTTTATCCATTCTATACCAAATTTCACCATC
CGTTAACTCAAATCTCTTACTAATAATAGCTATCACATCCATACTGGTAGGAGGCTGAGGAG
GATTAAATCAAACCCAACTCCGAAAAATCTTGGCATACTCCTCAATTACCCATATAGGCTGA
ATGGCTGCTATTATAGTATATAATCCTACCCTAGCAATTCTTAACCTAACAATTTACATCATA
ATAACTCTAGGAACATTTATACTATTCATACATAGCTCATCTACAACAACACTATCACTATCT
TATACATGAAATAAATTCCCATTAATAGCCCCACTAATCTTAATACTTATACTATCACTAGGA
GGCCTCCCTCCTCTCTCAGGCTTTATCCCCAAATGAATAATTATTCAAGAATTAACAAAAAA
CGACATAATTATTATACCTACACTTATAGCCATTACAGCCCTACTAAACCTATATTTTTACAC
ACGCCTAACATATACTACAGCACTAACTATATTTCCCGCAACAAATAATATAAAAATAAAAT
GACAATTCAAAACCACAAAAAAGACAACTCTCCTAGCCCCTCTAATCGTGACATCAACTATA
CTACTACCACTAACCCCAATACTAGCAGCTCTTGATTAGAGATTTAGGCTAAACAGACCAAG
GGCCTTCAAAGCCTCAAGTAAGTGAAAATCCACTTAATCTCTGCAATATTTAAGGACTGCAA
GAATCTATCTCACATCAACTGAACGCAAAACAATCGCTTTAATTAAGCTAAGCCCTTCTAGA
TCGGTGGGCTTTTACCCCACAAAACTTTAGTTAACAGCTAAAAACCCTAGACAACTGGCTTC
AATCTACTTCTCCCGCCGCGTAGGAAAAAAAGGCGGGAGAAGCCCCGGCAGAATTGAAGCT
GCTTCTTTGAATTTGCAATTCAATATGATTACTCACCACAGAGCCTGGTAAAAAGAGGACTT
AACCCCCATTCTTAGATTTACAGTCTAGTGCTTTTATCAGCCATTTTACCTATGTTCATTAACC
GATGACTGTTTTCCACCAACCACAAGGATATTGGCACTCTTTATCTTCTGTTTGGCGCATGAG
CTGGTATAACGGGCACTGCTCTTAGCCTTCTAATCCGTGCTGAATTAGGTCAGCCTGGAGCT
CTGTTAGGAGATGACCAAATTTACAATGTAGTTGTAACTGCCCACGCATTCGTAATAATCTT
CTTTATAGTTATGCCTATTATGATCGGAGGCTTTGGAAATTGACTAGTACCTTTGATAATTGG
TGCGCCTGATATAGCATTTCCCCGAATAAACAATATGAGTTTCTGATTACTACCTCCATCCTT
TCTTCTCCTTCTGGCTTCTTCCATAGTAGAAGCAGGCGCAGGGACTGGATGAACTGTATACC
CCCCTCTAGCAGGTAACTTAGCCCATGCAGGAGCATCAGTAGACCTAACAATCTTTTCTCTA
CATCTGGCAGGGGTTTCTTCTATTTTAGGGGCTATTAACTTTATTACCACTATCATCAACATG
AAACCCCCTGCAATATCTCAATATCAAACTCCCCTATTCGTGTGATCAGTACTTATCACAGCA
GTACTTCTCCTTCTATCTTTACCAGTCTTAGCAGCCGGAATTACTATACTACTTACAGATCGA
AATCTCAATACTACTTTCTTCGACCCGGCCGGAGGAGGGGATCCCATTCTATATCAACATCT
ATTTTGATTCTTTGGCCATCCTGAAGTCTATATTCTAATCCTCCCTGGGTTTGGAATAATTTCA
CACATTGTCACATACTATTCAGGAAAGAAAGAGCCCTTTGGTTATATGGGAATAGTGTGGGC
AATAATGTCAATTGGGTTCTTAGGATTTATTGTATGGGCCCATCATATATTTACTGTAGGCAT

AGATGTTGATACACGAGCCTATTTTACCTCGGCCACTATAATTATTGCAATCCCGACAGGAG
TTAAAGTATTCAGCTGACTAGCTACCCTGCATGGAGGAAATATTAAGTGATCCCCTGCCATA
ATGTGAGCCCTAGGTTTCATTTTTCTATTTACAGTAGGGGGTCTTACAGGCATTGTTCTAGCT
AACTCATCTCTGGACATTGTCCTCCATGACACATACTACGTAGTAGCACACTTCCACTATGTA
CTATCAATAGGGGCCGTGTTCGCTATTATAGGAGGCTTCGTCCATTGATTCCCATTATTTTCG
GGCTATACACTTAATAATACATGAGCAAAAATTCACTTCGTAATTATATTTGTTGGAGTCAA
TATAACATTCTTCCCCCAGCACTTCCTAGGTCTATCAGGTATGCCTCGACGATATTCTGATTA
TCCAGACGCCTATACAGCATGAAACACAGTGTCTTCAATGGGTTCATTCATTTCACTAACAG
CAGTTATGTTAATAATTTTCATAATTTGAGAAGCCTTCGCATCTAAACGAGAAGTGGCAGTG
ATTGAGCTTACTACAACTAATATTGAATGGCTCCACGGATGTCCTCCCCCATACCATACATTC
GAAGAGCCCGCCTACGTCATACTGAAATAAGAAAGGAAGGAATCGAACCCTCTAGAATTGG
TTTCAAGCCAACATCATAACCACTATGTCTCTCTCAATAAAGAGGTATTAGTAAAAATTACA
TAACTTTGTCAAAGTTGAATTATAGGTGAAAATCCTTTATATCTCTATGGCGCATCCCTTTCA
AACAGGCCTTCAAGATGCAACTTCCCCCATCATGGAAGAACTCCTACACTTCCATGATCATA
CACTAATAATTGTATTCTTAATTAGTTCCTTAGTTCTCTATATCATTTCAATTATATTAACTAC
TAAATTAACACATACAAATACAATAGACGCACAAGAAGTGGAGACAGTATGAACTATTCTA
CCAGCTATCATCTTAATTATAATTGCACTTCCATCACTACGAATTCTTTATATGATAGATGAA
ATTAATAACCCATCATTAACTGTAAAGACTATGGGGCATCAATGATACTGAAGCTATGAGTA
TACAGATTATGAAGACTTGAGCTTCGATTCTTATATAACCCCAACACAAGAATTAAAGCCCG
GAGAGCTGCGATTACTAGAGGTAGATAATCGAGTAGTACTACCCATAGAAATAACAATTCG
CATATTAATTTCCTCAGAAGATGTGCTACATTCGTGAGCTGTCCCATCTCTAGGACTAAAAA
CTGATGCAATCCCGGGGCGATTAAATCAAACAACTCTTATGGCCATGCGACCAGGACTATAT
TATGGCCAATGCTCAGAGATTTGCGGTTCTAATCATAGCTTTATACCCATTGTACTTGAAATA
GTTCCGCTGTCTTACTTTGAAAAATGATCCGCTTCAATACTATAAAATCATTAAGAAGCTTTA
TAGCGTTAACCTTTTAAGTTAAAGACTGAGAGTGCAAGTCTCTCCTTAATGGAAATGCCACA
ATTAGACACATCCACATGATTTATTACAATCCTATCCATGATCTTAACACTATTTATTATATT
TCAGTTAAAAGTCTCAAAACACAACTATCCTGAGTTTCCTGAATTAAAAACAACCCCACTAT
CAAAAAAACCTATGCCTTGAGAAGAAAAATGAACGAAAATCTGTTCGCCTCTTTTACTACCC
CAATGATAATAGGAGTACCAATTGTGGTTCTGATTATTATTTTCCCAAGTATCTTATTTCCCT
CACCTAATCGATTAATCAATAACCGCCTAGCTACTGTCCAACAATGATTAATTCAATTAGTA
TCTAAGCATATACTGTCTATTCACAATTATAAAGGACAAACCTGAGCGCTAATACTAATGTC
ATTAATTTTATTTATTGGCTCAACTAACCTTTTAGGCCTATTACCACATTCATTCACACCTACT
ACACAACTATCAATAAATTTAGGAATGGCCATTCCTCTGTGAGCAGGTACAGTAGTTATAGG
ATTTCGACACAAAACTAAGGCATCTCTGGCCCACTTTCTACCTCAAGGAACACCTCTTCCTCT
GATCCCAGTGTTAGTAATTATCGAGACAATTAGTCTGTTTATTCAACCTGTGGCCCTAGCTGT
ACGACTGACCGCTAATATTACTGCAGGCCATCTACTTATTCATTTAATCGGAGGGGCTACCC
TAGTTTTAATAAACATTAACCCTGTAACAGCCCTAATTACCTTTATTATTCTTATTCTACTTAC
TATTCTCGAATTAGCTGTAGCTCTTATCCAAGCCTATGTTTTCACCCTACTAGTTAGTCTATA
CTTACATGATAATACCTAATGATCCACCAAACACATGCATATCATATAGTTAACCCAAGCCC
ATGGCCACTTACAGGAGCTCTCTCGGCTCTTCTTATAACATCAGGACTAATTATATGATTTCA
CTTTAACTCGGTATACTTACTAATACTAGGCCTCACCACCAACATACTTACCATGTACCAATG
ATGACGAGATATCGTCCGGGAAAGTACCTTCCAAGGCCATCACACCCCAATCGTCCAAAAA
GGACTACGATATGGAATAGTCTTATTTATTGTATCAGAAGTATTTTTCTTCGCGGGATTCTTT
TGGGCCTTCTACCACTCAAGCCTAGCACCTACTCCTGAATTAGGAGCATGTTGACCACCTAC
AGGTATCATCCCCTTAAATCCGCTAGAAGTTCCACTACTTAACACCTCTGTACTTTTAGCATC
TGGGGTATCTATCACTTGAGCCCATCATAGTCTAATAGAAGGAGACCGTAAACATATGCTCC
AAGCCCTATTTATTACTATTTCACTAGGAGTTTATTTTACACTTCTACAAGCCTCAGAATACT
ATGAGACATCCTTTACTATTTCAGATGGAGTGTATGGTTCAACCTTCTTTATAGCAACTGGAT
TTCATGGGCTTCATGTAATTATTGGTTCAACTTTTCTTACTGTGTGTTTTCTACGACAACTGCA
CTTTCACTTTACATCCAACCATCACTTTGGCTTTGAAGCGGCAGCATGATACTGACACTTCGT
AGACGTTGTATGGCTTTTCCTTTATGTTTCTATTTACTGATGAGGATCCTACTTCTTTAGTATT

AACTAGTACGATTGACTTCCAATCAATAGGCTCTGGTACAAGCCAGAAAGAAGTAATAAAC
CTAATTCTAGCACTTCTAACCAACGTATTATTAGCCTCTCTGCTCGTTCTTATTGCATTCTGAC
TGCCACAACTAAATATCTATGCAGAAAAAGTAAGTCCTTATGAATGTGGATTTGATCCTGTA
GGATCAGCACGCCTTCCTTTCTCTATAAAATTTTTTCTAGTAGCTATTACATTCCTTTTATTCG
ACTTAGAAATTGCTCTACTCCTCCCGCTTCCTTGAGCATTACAGACTAACAACTTAATAACTA
TACTTATTATAGCTCTATTACTCATTTCCCTATTAGCCGCAAGCCTAGCTTATGAGTGAACCG
AAAAGGGGCTAGAATGGGCCGAATATGATAATTAGTTTAAACTAAAATAAGTGATTTCGAC
TCACTAGATTATGACTAACATCATAATTATCAAATGTCTGTGGTCTATATTAATCTCTTTCTA
GCCTTTACTATATCCTTAATAGGGCTACTCATCTACCGATCCCATTTAATATCCTCCCTATTAT
GCTTAGAAGGCATGATGCTGTCACTATTTGTTATACTAACAGTAACAATCCTAAGTAACCAC
TTCACACTAGCCAGTATGACTCCAATCATTCTATTAGTATTCGCTGCCTGTGAAGCAGCGTTG
GGGCTTTCTCTGCTAGTAATAGTCTCTAATACCTATGGAACTGACTACGTACAAAATCTTAAC
CTCCTACAATGCTAAAAATTATCCTCCCTACCATAATACTAGTACCCCTTACATGAATATCAA
AGCCTAACATAATCTGAATTAATACAACAACCTATAGTCTACTCATCAGCCTTATTAGTCTG
ACATATCTCAATCAACTCAGTGATAATAGTCTAAACTTCTCATTAACATTCTTTACCGACTCC
CTATCAGCCCCCCTGTTAGTTCTCACAACATGACTACTGCCCTTAATACTTATAGCTAGTCAA
TTTCACCTGTCAAAAGAGACCCTGACCCGGAAAAAATTATATATCACAATACTAATTCTCCT
GCAACTATTCTTAATTATAACATTTTCTGCTACTGAGTTAATCATATTCTATATTTTATTTGAA
GCTACCCTGGTACCGACTTTAGTTATCATTACTCGATGAGGAAACCAAACAGAACGACTAAA
CGCGGGCCTCTATTTTTTATTCTACACCTTAGTAGGATCCTTGCCCTTACTAGTTGCACTGTTA
TATGCCCAAAACAGCTTAGGATCACTAAATTTCTCATAGTACAGTACTGAACTCAGCCCTT
GCCAAATTCCTGATCTAACATTTTCATGTGGCTGGCATGTATAATAGCATTCATAGTAAAAA
TACCGCTATATGGCTTACACCTATGACTACCCAAAGCACATGTAGAAGCTCCTATTGCAGGA
TCCATAGTACTTGCTGCAGTACTTCTAAAACTAGGGGGATACGGTATAATGCGGATTACAAC
AATACTCAACCCTCTAACAAATTTTATAGCCTACCCCTTCATAATGCTCTCACTATGAGGAAT
AATCATAACAAGTTCTATTTGTTTACGCCAGACAGACCTAAAGTCCCTAATTGCATACTCCTC
TGTTAGTCACATAGCCCTAGTCATTGTAGCAATTCTAATCCAATCCCCATGAAGCTACATAG
GAGCTACTGCTCTAATAATCGCCCATGGGTTAACATCTTCTATGCTATTCTGTCTTGCAAACT
CTAACTACGAGCGAACCCATAGCCGAACTATAATTCTCGCACGGGGCTTGCAAACGCTTCTT
CCACTTATAGCAGCTTGATGACTATTAGCAGGCCTAACTAACTTAGCTCTTCCTCCTACAATT
AATCTGATTGGGGAACTGTTTGTAGTGATAGCCACATTTTCATGATCCAATATTACCATTCTC
TTAATAGGAGTTAACATTACCATTACTGCTCTCTACTCTCTATATATACTAATTTCTACACAG
CGAGGCAAGTATACAGACCACATCAAAAATATTAAGCCATCTTATACACGAGAAAATGCCT
TAATAGCCTTACATTTACTACCCCTTTTACTTCTATCTCTTAACCCTAAAGTAGTTCTAGGCTT
TATTTACTGTAAATATAGTTTAACAAAAACATTAGATTGTGAATCTAGTAATAAAAGCTTAA
ATCTTTTTATTTACCGAAAAAGCATGCAAGAACTGCTAATTCATGCACCCATGCATAAAATC
ATGGCTTTTTCAACTTTTAGGGGATAGAAGTAATCCATTGGTCTTAGGAGCCAAAAAATTGG
TGCAACTCCAAATAAAAGTAATTAACCTACTTACCTCCTTCACCATTACAACTCTATTTATAT
TAATCATGCCCATTATCCTAACCTGTACCCCAGCATATGAAAATAAACTTTACCCGCAATAT
GTAAAAACCACAGTCTCTTATGCCTTCACAATTAGCATAATTCCCATAGTAATATTCATCCAC
TCCGGACAAGAAGCAATCATCTCAAACTGACACTGAATAACCATCCAAACCCTAAAACTCA
GTCTAAGCTTCAAATTAGATTACTTTTCAATAATCTTCGTACCAGTGGCCCTATTCGTCACAT
GGTCAATTATAGAATTCTCTATATGATATATACACGCAGATCCTAACATTAACCGATTCTTTA
AATATCTACTTATATTCCTCGTCACGATAATAATTCTAGTAACCGCAAATAATCTATTTCAGC
TATTCATCGGCTGAGAGGGAGTAGGAATCATATCATTTCTCCTCATTGGATGATGATATGGA
CGAACAGATGCTAATACGGCTGCCCTACAAGCAGTACTATACAACCGCATTGGAGATGTTGG
TTTTATCATGGCCATAGCTTGATTCTTACTCAATACTAATGCATGGGACTTCCAACAAATCTT
TATAATCGAACATAAAAACCTCAATATTCCACTGGCAGGACTACTTCTAGCAGCTACCGGAA
AGTCTGCCCAATTTGGACTTCATCCATGACTCCCATCAGCCATAGAAGGCCCCACTCCTGTCT
CAGCACTACTTCACTCTAGCACAATAGTAGTAGCAGGCGTATTTCTTCTAGTCCGGTTTCACC
CCTTAATAGAACATAACAAGACAATTCAAACGGTTACCCTATGCTTAGGGGCAATAACAACC

TTGTTTACAGCAATCTGCGCCCTTACCCAAAATGATATCAAAAAAATTATTGCCTTCTCCACC
TCGAGCCAACTAGGACTAATAATTGTAACAATTGGTATTAATCAGCCTCATCTGGCCTTTCTC
CATATCTGCACGCACGCATTCTTCAAGGCCATGCTATTTCTATGCTCCGGATCTATTATTCAT
AGTCTAAACAACGAACAAGATATTCGAAAGATAGGAGGGCTATTTAAAAGTCTACCATTTA
CCACTACTGCTCTAATCATCGGAACTCTAGCACTTACAGGAATGCCCTTCTTAACAGGATTTT
ACTCCAAAGACTTAATTATTGAAACCGCCAATACGTCATACACCAACGCCTGAGCCTTACTA
ACAACCCTTATCGCCACATCCATAACTGCCGCCTACAGTACTCGAATTGTATTTTTTACACTT
CTAGGACAACCTCGCTATAGTCCCATTATCACCATTAACGAGAATAACCAGTTCCTGACTAA
CTCTGTCAAACGATTGCTACTAGGAAGTATCTTTGCGGGATTCTTAATCTCTCATAACATTAC
ACCCACCACTGTCCCACAAATAACCATGCCCTGTCACCTAAAAATGATAGCCCTTACAGTAA
CAATCCTAGGCTTTATTCTAGCACTAGAACTTAACCTAGCCATACAAAATCTCAAATTTGACT
ACCCGTTAAGCACGTTCAAATTTTCAAATCAACTAGGATATTTCCCTACTACTCTGCATCGTC
TCGCACCAATAATAGGTCTATCAATAAGCCAAAAAACAGCATCCATACTGCTAGATGCAATC
TGACTAGAGAATGTTCTACCAAAGTCCATCTCCTACTTTCAAATAAAATCCTCAATCACAGT
CTCCAACCAAAAGGGACTAATTAAACTATACTTTCTCTCCTTTATAATTACACTAATCTTAAG
TCTCTTTATACTTAATTTCCACGAGTGACTTCCATAATTACCAATACACCAATAAGGAGAGAT
CATCCAGTAACAATAACTAATCAAGTACCATAACTATATAAAGCTGCAATTCCCATAGCCTC
TTCACTAAAAAATCCTGAATCCCCTGTATCATAAATCACCCAATCTCCTATTCCATTAAACTT
AAATACTACCTCGGCTTCATCATCCTTTAAAATATAACAAACAGCCAGTAACTCAGACAACA
GACCTACAATAAATGCACCTAGTACAGCCTTATTAGAAAGTCAAACCTCAGGATATTGCTCA
GTGGCTATGGCCGTAGTGTACCCGAACACTACAAGTATACCCCCTAAATAAATTAAAAAGAC
CATTAAACCCAAAAAAGATCCTCCAAAGCTTAACACAATACCACACCCTACACCCCCACTAA
CAATCAAGACTAAACCTCCGTAGATAGGAGAGGGCTTAGAAGAAAACCCCGCAAAACTAAC
TACAAAAACGATACTTAAAATAGATACAATGTATGTTATCATCATTCTTACATGGAATCTAA
CCATGACCAATGACATGAAAAATCATCGTTGTATTTCAACTATAAGAACCCCAATGATCAAC
ATCCGAAAAACTCATCCATTAGTTAAAATTATCAACAACTCATTCATTGACCTTCCAACACC
ATCAAACATTTCAACATGATGGAACTTTGGGTCCCTGTTAGGAGTGTGTCTGATCTTGCAAA
TCTTAACAGGCTTATTTCTAGCCATACACTATACATCAGATACAGCTACAGCCTTTTCATCAG
TCGCACACATTTGTCGAGACGTCAACTATGGTTGATTTATCCGATATATACATGCCAATGGG
GCCTCTATATTTTTTATCTGCCTATTTATACACGTAGGGCGAGGCTTATACTATGGATCATAC
CTATTTCCAGAGACATGGAATATCGGAATTATTCTCCTACTTACAGTTATAGCCACAGCATTC
ATAGGATATGTACTACCTTGAGGACAAATATCCTTCTGAGGAGCAACCGTCATTACTAACCT
ACTATCAGCAATTCCTTACATTGGCACTAATCTAGTGGAGTGAATCTGAGGGGGTTTCTCCG
TAGATAAAGCAACACTAACCCGATTTTTTGCTTTTCACTTTATCCTTCCATTTATCATCTCAGC
ACTAGCAATAGTCCATCTATTATTCCTTCACGAAACAGGATCTAATAACCCCTCCGGAATTC
CATCTGACCCAGACAAAATCCCATTTTACCCCTATCATACAATTAAAGACATCCTAGGCGTC
CTATTTCTTGTCCTCGCCTTAATAACCCTGGCTTTATTCTCACCAGACCTGTTAGGAGACCCT
GATAACTATACCCCTGCAAATCCACTAAGTACCCCGCCACATATTAAGCCTGAATGGTACTT
TCTATTTGCCTACGCTATCCTGCGATCTATCCCTAATAAACTAGGAGGGGTGCTAGCTCTAAT
CTTCTCTATTCTAATTCTAACTATTATTCCACTATTACATACATCCAAACAACGAAGCATGAT
ATTCCGACCTCTAAGTCAATGCTTATTCTGACTCCTAGTAGCAGACCTACTCACACTAACATG
AATTGGAGGACAGCCAGTAGAACACCCCTTCATTATTATTGGGCAATTGGCCTCTATTCTCT
ACTTTACAATTCTTCTAGTACTTATACCTATCACTAGCATTATTGAGAATAGCCTCTCAAAAT
GAAGAGTCTTTGTAGTATATTAATTACTTTGGTCTTGTAAACCAAAAACGGAGATTCCTCTAC
TTCTCCCTAAGACTCAAGGAAGGAGCAACAACCCCACTACCAGCACCCAAAGCTGGTGTTCT
ACTTAAACTATCCCCTG
>SRR6109210 AssemblyGraph Reorganized
ATACTATAAATCCACCTCTCATTTTATTCACTTCATACATGCTATTACACACTCTGTGCCATC
ATAGTATGTTTTCATACATCCTCCCTTCTTTCACACCCTATGTATATCGTACATTAATGGTGTA
CCCCCCCCTCCCCCCTATGTATATCGTGCATTAATGGCGTGCCCCATGCATATAAGCATGTAC
ATACTGTGCTTGGCTTTACATGAGGATACTCATTACAAGAACTTATTTCAAGCGATAGTCTAT

GAGCATGTATTTCACTTAGTCCAAGAGCTTGATCACCAAGCCTCGAGAAACCAGCAATCCTT
GCGAGTACGTGTACCTCTTCTCGCTCCGGGCCCATAATTTGTGGGGGTTTCTATACTGAAACT
ATACCTGGCATCTGGTTCTTACCTCAGGGCCATGTTAGCGTCAACTCAATCCTACTAACCCTT
CAAATGGGACATCTCGATGGACTAATGACTAATCAGCCCATGATCACACATAACTGTGGTGT
CATGCATTTGGTATTTTTTAATTTTAGGGGGGGAACTTGCTATGACTCAGCTATGACCGTAAA
GGTCTCGTCGCAGTCAAATCAGCTGTAGCTGGGCTTATTCATCTTTCGAGGCTCCTCATGGAT
ACCCATAAGGTGCAATTCAGTCAATGGTCACAGGACATAACACTATAGATCACCCGGACTG
GCGTTACGTGTACGTACGTGTACGTACGTGTACGTACGTGTACGCACGTGTACGTACGTGTA
CGTACGTGTACGTACGTGTACGCACGTGTACGTACGTGTACGCACCCGGACTGGCGTTACGT
GTACGTACGTGTACGTACGTGTACGTACGTGTACGCACGTGTACGTACGTGTACGTACGTGT
ACGTACGTGTACGCACGTGTACGTACGTGTACGCACGTGTACGTACGTGTACGCACGTGTAC
GCACGTGTACGCACGTGTACGTACGTGTACGTACGTGTACGTACGTGTACGTACGTGTACGC
ACGTGTACGCACGTGTACGCACGTGTACGCACGTGTACGCACGTGTACGCACGTGTACGTAC
GTGTACGCGTACGTATTTTAGATACTAAGTTAGCTTAGACAAACCCCCCTTACCCCCCGTAA
CTTCAAGAAGCTTACATATACTTATGGATGTCCTGCCAAACCCCAAAAACAAGACTAAATAT
ATGCGCAAACATGAAGTCACTTACACCTAAACCCATATAATTAAGCTAACCCCCCAGCCAAT
GTTGCAACAACTACGGACATGGGACTCTAAATTTTAATTTATCTATAGATATTTTTCTTTTAC
TGTGTCTCCCCAGCATTGATTTTTTAATTATCATTATTCCACACCACCAATTTCCATTGAGCTA
TTTCACATGAGTTCCAAATCAATTATGTTCATGTAGCTTAACGAATAAAGCAAGGTACTGAA
AATGCCTAGATGGGTCACGCTACCCCATAGACATAAAGGTTTGGTCCTAGCCTTCCTATTAG
CCATTAACAAGATTACACATGTAAGTCTCCACGCTCCAGTGAAAATGCCCCTTAAGTCCTCT
TAGACGACCTAAAGGAGCGGGTATCAAGCACACCTTATGGTAGCTCACAACGCCTTGCTTAG
CCACACCCCCACGGGAAACAGCAGTGATAAAAATTAAGCTATGAACGAAAGTTCGACTAAG
CTATGTTAATACTAGGGTTGGTAAATCTCGTGCCAGCCACCGCGGTCATACGATTAACTCGA
GTTAATAGGCCTACGGCGTAAAGCGTGTAAAAGAAAAAATCTCCTCTACTAAAGTTAAAGT
ATGATTAAGCTGTAAAAAGCTACCATTAATACTAAAATAAACTACGAAAGTGACTTTAAAAT
TTCTGATTACACGATAGCTAGGGCCCAAACTGGGATTAGATACCCCACTATGCCTAGCTCTA
AACATAGATATTTTACTAAACAAAACTATTCGCCAGAGAACTACTAGCAACAGCTTAAAACT
CAAAGGACTTGGCGGTGCTTTATATCCCCCTAGAGGAGCCTGTTCTGTAATCGATAAACCCC
GATAGACCTCACCATCCCTTGCTAATTCAGTTTATATACCGCCATCTTCAGCAAACCCTTAAA
AGGAAAAAAAGTAAGCATAACTACCCTACATAAAAAAGTTAGGTCAAGGTGTAACCTATGG
GCTGGGAAGAAATGGGCTACATTTTCTATTCAAGAACAACTTCTACGAAAACTTTTATGAAA
CTAAAAGCTAAAGGCGGATTTAGTAGTAAATTAAGAATAGAGAGCTTAATTGAACAGGGCA
ATGAAGCACGCACACACCGCCCGTCACCCTCCTCGAGTGATATAATTTAATTATAACCTATT
TAAACTAAGCAAAGCATAAGAGGAGACAAGTCGTAACAAGGTAAGCATACTGGAAAGTGTG
CTTGGATGAGCCAAAGTGTAGCTTAAACAAAGCGTCTGGCTTACATCCAGAAGATTTCATTA
ATATATGACTACTTTGAACCCAAAGCTAGCCCAAGCAACAATGACTAGTAAAACCATTATGA
AACATTCAAACAAAACATTTAGTAGCATGACTAGAGTATAGGAGATAGAAATTTTTAACTGG
AGCTATAGAGAGAGTACCGCAAGGGAATGATGAAAGATTACCTAAAGTGATAAACAGCAAA
GATTGCCCCTTCTACCTTTTGTATAATGAGTTAGCTAGAAATAACTTAACAAAGAGAACTTA
AGCTAAGTCCCCCGAAACCAGACGAGCTACCTATGAACAATCCACTGGGATGAACTCATCTA
TGTTGCAAAATAGTGAGAAGATCCATAGGTAGAGGTGAAAGGCCTAACGAGCCTGGTGATA
GCTGGTTGCCCAGAATAGAATTTTAGTTCGACTTTAAACCTGCCTACAAAACTAATAATTCT
AATGCAGATTTAAAATATATTCTAAAAAGGTACAGCTTTTTAGAGTTAAGGATACAGCCTTA
CTTAGAGAGTAAATATTTATATAAGCCATAGTAGGCCTAGAGGCAGCCATCAATTAAGAAA
GCGTTAAAGCTCAACATCTCTATTAACTTAATACCAAGAATATTTAATCAACTCCTAATGTAT
TACTGGGTCAATCTATTTAAATATAGAAGTGATAATGCTAATATGAGTAACAAGAAATATTT
CTCCCATGCATAAGCTTATAACAGCAACGGATAACCACTGATAGTTAACAACAACATAGAA
ATAACCTAATGATAAAACACCTATTAAATCAATTGTTAGTCCAACACAGGCATGCAATCAGG
GAAAGATTAAAAGAAGTGAAAGGAACTCGGCAAATACAAACCCCGCCTGTTTACCAAAAAC
ATCACCTCCAGCATTTCCAGTATTGGAGGCACTGCCTGCCCGGTGACATCAGTTAAACGGCC

GCGGTATTCTGACCGTGCAAAGGTAGCATAATCATTTGTTCTCTAAATAAGGACTTGTATGA
ATGGCCACACGAGGGTTTAACTGTCTCTTACTTCCAATCAGTGAAATTGACCTCCCCGTGAA
GAGGCGGGGATAAGACAATAAGACGAGAAGACCCTATGGAGCTTTAATTAACTAATTCAAA
AAGAAACTACTAACGACCCAACAGGAATAATATATCTCTTTTATGAATTAGCAATTTAGGTT
GGGGCGACCTCGGAGGACAAAATAGCCTCCGAGTGATTATAAATCTAGACTTACCAGTCAA
AATGCTTAATCACTTATTGATCCAAAAATTCTTTTGATCAACGGAACAAGTTACCCTAGGGA
TAACAGCGCAATCCTATCCGAGAGTCCATATCGACAATAGGGTTTACGACCTCGATGTTGGA
TCAGGACATCCTAATGGTGCAGCAGCTATTAAAGGTTCGTTTGTTCAACGATTAAAGTCCTA
CGTGATCTGAGTTCAGACCGGAGCAATCCAGGTCGGTTTCTATCTATTCAAATAATTTCTCCC
AGTACGAAAGGACAAGAGAAATAAGGCCTACTTCTCTGAAGCGCCTTAAGACCAATAGATG
AATTTATCTAAATCTAGTAAATCTAACTCCAATATTGCCCAAGAGACAGGGCTTTGTTAGGG
TGGCAGAGCCCGGTAATTGTGCAAAACTTAAACTCTTGTGTCCAGAGGTTCAATTCCTCTCC
CTAGCATATGTTTATAATTAACATCTTCTCACTAATTGTACCTATTCTTCTTGCTGTAGCCTTT
CTGACTCTAGTAGAACGAAAAGTACTAGGCTACATACAGCTTCGAAAAGGCCCAAATATTGT
AGGTCCATATGGTCTCCTACAACCAATCGCAGATGCCGTAAAACTCTTTACTAAAGAACCTT
TACGACCACTTACGTCATCCATAACTATATTTATTATAGCCCCTATTCTAGCCTTAACACTAG
CTCTGACCATGTGAGTCCCCTTACCAATACCGCACCCCCTTATTAACATAAACCTAGGAGTA
CTATTCATACTAGCTATATCAAGTCTAGCTGTATACTCTATTCTCTGATCTGGATGAGCCTCA
AACTCAAAATATGCCCTAATTGGAGCCCTACGAGCCGTAGCTCAAACGATCTCATATGAAGT
CACACTAGCCATTATCCTTTTATCCGTTCTACTAATAAATGGCTCATTTACGCTATCCACGCT
AATCATTACCCAAGAACATCTTTGATTAATCTTCCCTGCATGACCACTAGCTATAATATGATT
TATTTCTACCCTAGCAGAAACAAATCGAGCCCCCTTTGACCTAACAGAAGGAGAATCAGAAC
TAGTCTCTGGGTTTAATGTCGAATATGCAGCAGGTCCATTTGCCCTGTTCTTCCTAGCAGAAT
ATGCCAACATCATCATAATAAATGCTCTTACAACTATTCTATTCTTCGGAGCATTTCACAGCC
CCTACATACCAGAATTATACATTGTTAATTTTACCGTAAAAACTTTACTCCTCACAATCTCTT
TTCTATGAATCCGAGCATCATATCCTCGATTCCGATACGACCAACTAATACACCTACTATGA
AAAAACTTTCTACCTCTCACACTAGCTCTATGTATATGACATGTAACTCTACCTATTATTACA
GCAAGCATTCCACCTCAAACGTAAGAAATATGTCTGACAAAAGAATTACTTTGATAGAGTAA
GCCATAGGGGTTTAAGTCCCCTTATTTCTAGAATCATAGGAATCGAACCTAATCTTAAGAAC
TCAAAAATCTTCGTGCTACCTAACTACACCATATCCTAAAGTAAGGTCAGCTAAATAAGCTA
TCGGGCCCATACCCCGAAAATGTTGGTTTATCCCCTTCCCATACTAATAAAACCAGCCATCCT
TATTACCATTACATCCACCGTTGTCTTAGGAACTATAATTGTTCTATTTAGCTCCCACTGATT
CATAATCTGAGTTGGCTTTGAAATAAATATACTAGCCATTATCCCAATTCTAATAAAAAAAT
ATAACCCACGAGCCATAGAAGCTTCCACAAAATATTTTCTAACACAAGCTACAGCATCCATG
CTTCTAATGCTAAGCATCATCATCAATCTACTATGCTCAGGACACTGGACAGTTTCAACAATT
CCCAATCCAGTAGCATCTACCATAATTACTATTGCTCTAACAATAAAACTTGGCCTATCACC
ATTTCACTTTTGAGTCCCAGAAGTTACACAAGGCATTTCCTTATCTTCAGGAATAATCCTATT
AACATGACAAAAAATTGCACCTTTATCCATTCTATACCAAATTTCACCATCCGTTAACTCAA
ATCTCTTACTAATAATAGCTATCACATCCATACTGGTAGGAGGCTGAGGAGGATTAAATCAA
ACCCAACTCCGAAAAATCTTGGCATACTCCTCAATTACCCATATAGGCTGAATGGCTGCTAT
TATAGTATATAATCCTACCCTAGCAATTCTTAACCTAACAATTTACATCATAATAACTCTAGG
AACATTTATACTATTCATACATAGCTCATCTACAACAACACTATCACTATCTTATACATGAAA
TAAATTCCCATTAATAGCCCCACTAATCTTAATACTTATACTATCACTAGGAGGCCTCCCTCC
TCTCTCAGGCTTTATCCCCAAATGAATAATTATTCAAGAATTAACAAAAAACGACATAATTA
TTATACCTACACTTATAGCCATTACAGCCCTACTAAACCTATATTTTTACACACGCCTAACAT
ATACTACAGCACTAACTATATTTCCCGCAACAAATAATATAAAAATAAAATGACAATTCGAA
ACCACAAAAAAGACAACTCTCCTAGCCCCTCTAATCGTGACATCAACTATACTACTACCACT
AACCCCAATACTAGCAGCTCTTGATTAGAGATTTAGGCTAAACAGACCAAGGGCCTTCAAAG
CCTCAAGTAAGTGAAAATCCACTTAATCTCTGCAATATTTAAGGACTGCAAGAATCTACCTC
ACATCAACTGAACGCAAAACAATCGCTTTAATTAAGCTAAGCCCTTCTAGATCGGTGGGCTT
TTACCCCACAAAACTTTAGTTAACAGCTAAAAACCCTAGACAACTGGCTTCAATCTACTTCT

CCCGCCGCGTAGGAAAAAAAGGCGGGAGAAGCCCCGGCAGAATTGAAGCTGCTTCTTTGAA
TTTGCAATTCAATATGATTACTCACCACAGAGCCTGGTAAAAAGAGGACTTAACCCCCATTC
TTAGATTTACAGTCTAGTGCTTTTATCAGCCATTTTACCTATGTTCATTAACCGATGACTGTTT
TCCACCAACCACAAAGATATTGGCACTCTTTATCTTCTGTTTGGCGCATGAGCTGGTATAACG
GGCACTGCTCTTAGCCTTCTAATCCGTGCTGAATTAGGTCAGCCTGGAGCTCTGTTAGGAGA
TGACCAAATTTACAATGTAGTTGTAACTGCCCACGCATTCGTAATAATCTTCTTTATAGTTAT
GCCTATTATGATCGGAGGCTTTGGAAATTGACTAGTACCTTTGATAATTGGTGCGCCTGATAT
AGCATTTCCCCGAATAAACAATATGAGTTTCTGATTACTACCTCCATCCTTTCTTCTCCTTCTG
GCTTCTTCCATAGTAGAAGCAGGCGCAGGGACTGGATGAACTGTATACCCTCCTCTAGCAGG
TAACTTAGCCCATGCAGGAGCATCAGTAGACCTAACAATCTTTTCTCTACATCTGGCAGGGG
TTTCTTCTATTTTAGGGGCTATTAACTTTATTACCACTATCATCAACATGAAACCCCCTGCAA
TATCTCAATATCAAACTCCCCTATTCGTGTGATCAGTACTTATCACAGCAGTACTTCTCCTTC
TATCTTTACCAGTCTTAGCAGCTGGAATTACTATACTACTTACAGATCGAAATCTCAATACTA
CTTTCTTCGACCCGGCCGGAGGAGGGGATCCCATTCTATATCAACATCTATTTTGATTCTTTG
GCCATCCTGAAGTCTATATTCTAATCCTCCCTGGGTTTGGAATAATTTCACACATTGTCACAT
ACTATTCAGGAAAGAAAGAGCCCTTTGGTTATATGGGAATAGTGTGGGCAATAATGTCAATT
GGATTCTTAGGATTTATTGTATGGGCCCATCATATATTTACTGTAGGCATAGATGTTGATACA
CGAGCCTATTTTACCTCGGCCACTATAATTATTGCAATCCCGACAGGAGTTAAAGTATTCAG
CTGACTAGCTACCCTGCATGGAGGAAATATTAAGTGATCCCCTGCCATAATGTGAGCCCTAG
GTTTCATTTTTCTATTTACAGTAGGGGGTCTTACAGGCATTGTTCTAGCTAACTCATCTCTGG
ACATTGTCCTCCATGACACATACTACGTAGTAGCACACTTCCACTATGTACTATCAATAGGG
GCCGTGTTCGCTATTATAGGAGGCTTCGTCCATTGATTCCCATTATTTTCGGGCTATACACTT
AATAATACATGAGCAAAAATTCACTTCGTAATTATATTTGTTGGAGTCAATATAACATTCTTC
CCCCAGCACTTCCTAGGTCTATCAGGTATGCCTCGACGATATTCTGATTATCCAGACGCCTAT
ACAGCATGAAACACAGTGTCTTCAATGGGTTCATTCATTTCACTAACAGCAGTTATGTTAAT
AATTTTCATAATTTGAGAAGCCTTCGCATCTAAACGAGAAGTGGCAGTGATTGAGCTTACTA
CAACTAATATTGAATGGCTCCACGGATGCCCTCCCCCATACCATACATTCGAAGAGCCCGCC
CACGTCATACTGAAATAAGAAAGGAAGGAATCGAACCCTCTAGAATTGGTTTCAAGCCAGC
ATCATAACCACTATGTCTCTCTCAATAAAGAGGTATTAGTAAAAATTACATAACTTTGTCAA
AGTTGAATTATAGGTGAAAATCCTTTATATCTCTATGGCGCATCCCTTTCAAACAGGCCTTCA
AGATGCAACTTCCCCCATCATGGAAGAACTCCTACACTTCCATGATCATACACTAATAATTG
TATTCTTAATTAGTTCCTTAGTTCTCTATATCATTTCAATTATATTAACTACTAAATTAACACA
TACAAATACAATAGACGCACAAGAAGTGGAGACAGTATGAACTATTCTACCAGCTATCATCT
TAATTATAATTGCACTTCCATCACTACGAATTCTTTATATGATAGATGAAATTAATAACCCAT
CATTAACTGTAAAGACTATGGGGCATCAATGATACTGAAGCTATGAGTATACAGATTATGAA
GACTTGAGCTTCGATTCTTATATAACCCCAACACAAGAATTAAAGCCCGGAGAGCTGCGATT
ACTAGAGGTAGATAATCGAGTAGTACTACCCATAGAAATAACAATTCGCATATTAATTTCCT
CAGAAGATGTGCTACATTCGTGAGCTGTCCCATCTCTAGGACTAAAAACTGATGCAATCCCG
GGGCGATTAAATCAAACAACTCTTATGGCCATGCGACCAGGACTATATTATGGCCAATGCTC
AGAGATTTGCGGTTCTAATCATAGCTTTATACCCATTGTACTTGAAATAGTTCCGCTGTCTTA
CTTTGAAAAATGATCCGCTTCAATACTATAAAATCATTAAGAAGCTTTATAGCGTTAACCTTT
TAAGTTAAAGACTGAGAGTGCAAGTCTCTCCTTAATGGAAATGCCACAATTAGACACATCCA
CATGATTTATTACAATCCTATCCATGATCTTAACACTATTTATTATATTTCAGTTAAAAGTCT
CAAAACACAACTATCCTGAGTTTCCTGAATTAAAAACAACCCCACTATCAAAAAAACCTATG
CCTTGAGAAGAAAAATGAACGAAAATCTGTTCGCCTCTTTTACTACCCCAATGATAATAGGA
GTACCAATTGTGGTTCTGATTATTATTTTCCCAAGTATCTTATTTCCCTCACCTAATCGATTAA
TCAATAACCGCCTAGCTACTGTCCAACAATGATTAATTCAATTAGTATCTAAGCATATACTGT
CTATTCACAATTATAAAGGACAAACCTGAGCGCTAATACTAATGTCATTAATTTTATTTATTG
GCTCAACTAACCTTTTAGGCCTATTACCACATTCATTCACACCTACTACACAACTATCAATAA
ATTTAGGAATGGCCATTCCTCTGTGAGCAGGTACAGTAGTTATAGGATTTCGACACAAAACT
AAGGCATCTCTGGCCCACTTTCTACCCCAAGGAACACCTCTTCCTCTGATCCCAATGTTAGTA

ATTATCGAGACAATTAGTCTGTTTATTCAACCTGTGGCCCTAGCTGTACGACTGACCGCTAAT
ATTACTGCAGGCCATCTACTTATTCATTTAATCGGAGGGGCTACCCTAGTTTTAATAAACATT
AACCCTGTAACAGCCCTAATTACCTTTATTATTCTTATTCTACTTACTATTCTCGAATTAGCTG
TAGCTCTTATCCAAGCCTATGTTTTCACCCTACTAGTTAGTCTATACTTACATGATAATACCT
AATGATCCACCAAACACATGCATATCATATAGTTAATCCAAGCCCATGGCCACTTACAGGAG
CTCTCTCGGCTCTTCTTATAACATCAGGACTAATTATATGATTTCACTTTAACTCGGTATACTT
ACTAATACTAGGCCTCACCACCAACATACTTACCATGTACCAATGATGACGAGATATCGTCC
GGGAAAGTACCTTCCAAGGCCATCACACCCCAATCGTCCAAAAAGGACTACGATATGGAAT
AGTCTTATTTATTGTATCAGAAGTATTTTTCTTCGCGGGATTCTTTTGGGCCTTCTACCACTCA
AGCCTAGCACCTACTCCTGAATTAGGAGCATGTTGACCACCTACAGGTATCATCCCCTTAAA
TCCGCTAGAAGTTCCACTACTTAACACCTCTGTACTTCTAGCATCTGGGGTATCTATCACTTG
AGCTCATCATAGTCTAATAGAAGGAGACCGCAAACATATGCTCCAAGCCCTATTTATTACTA
TTTCACTAGGAGTTTATTTTACACTTCTACAAGCCTCAGAATACTATGAGACATCCTTTACTA
TTTCAGATGGAGTGTATGGTTCAACCTTCTTTATAGCAACTGGATTTCATGGGCTTCATGTAA
TTATTGGTTCAACTTTTCTTACTGTGTGTTTTCTACGACAACTGCACTTTCACTTTACATCCAA
CCATCACTTTGGCTTTGAAGCGGCAGCATGATACTGACACTTCGTAGACGTTGTATGGCTTTT
CCTTTATGTTTCTATTTACTGATGAGGATCCTACTTCTTTAGTATTAACTAGTACGATTGACCT
CCAATCAATAGGCTCTGGTACAAGCCAGAAAGAAGTAATAAACCTAATTCTAGCACTTCTAA
CCAACGTATTATTAGCCTCTCTGCTCGTTCTTATTGCATTCTGACTGCCACAACTAAATATCT
ATGCAGAAAAAGTAAGTCCTTATGAATGTGGATTTGATCCTATAGGATCAGCACGCCTTCCT
TTCTCTATAAAATTTTTCTAGTAGCTATTACATTCCTTTTATTCGACTTAGAAATTGCTCTAC
TCCTCCCGCTTCCTTGAGCATTACAGACTAACAACTTAATAACTATACTTATTATAGCTCTAT
TACTCATTTCCCTATTAGCCGCAAGCCTAGCTTATGAGTGAACCGAAAAGGGGCTAGAATGG
GCCGAATATGATAATTAGTTTAAACTAAAATAAGTGATTTCGACTCACTAGATTATGACTAA
CATCATAATTATCAAATGTCTGTGGTCTATATTAATCTCTTTCTAGCCTTTACTATATCCTTAA
TAGGGCTACTCATCTATCGATCCCATTTAATATCCTCCCTATTATGCTTAGAAGGCATGATGC
TGTCACTATTTGTTATACTAGCAGTAACAATCCTAAGTAACCACTTCACACTAGCCAGTATG
ACTCCAATCATTCTATTAGTATTCGCTGCCTGTGAAGCAGCGTTGGGGCTTTCTCTGCTAGTA
ATAGTCTCTAATACCTATGGAACTGACTACGTACAAAATCTTAACCTCCTACAATGCTAAAA
ATTATCCTCCCTACCATAATACTAGTACCCCTTACATGAATATCAAAGCCTAACATAATCTGA
ATTAATACAACAACCTATAGTCTACTCATCAGCCTTATTAGTCTGACATATCTCAATCAACTC
AGTGATAATAGTCTAAACTTCTCATTAACATTCTTTACCGACTCCCTATCAGCCCCCCTGTTA
GTTCTCACAACATGACTACTGCCCTTAATACTTATAGCTAGTCAATTTCACCTGTCAAAAGAG
ACCCTGACCCGGAAAAAATTATATATCACAATACTAATTCTCCTGCAACTATTCTTAATTATA
ACATTTTCTGCTACTGAGTTAATCATATTCTATATTTTATTTGAAGCTACCCTGGTACCGACTT
TAGTTATCATTACTCGATGAGGAAACCAAACAGAACGACTAAACGCGGGCCTCTATTTTTTA
TTCTACACCTTAGTAGGATCCTTGCCCTTACTAGTTGCACTGTTATATGCCCAAAACAGCTTA
GGATCACTAAATTTTCTCATAGTACAGTACTGAACTCAGCCCTTGCCAAATTCCTGATCTAAC
ATTTTCATGTGGCTGGCATGTATAATAGCATTCATAGTAAAAATACCGCTATATGGCTTACA
CCTATGACTACCTAAAGCACATGTAGAAGCTCCTATTGCAGGATCCATAGTACTTGCTGCAG
TACTTCTAAAACTAGGGGGATACGGTATAATGCGGATTACAACAATACTCAACCCTCTAACA
AATTTTATAGCCTACCCCTTCATAATGCTCTCACTATGAGGAATAATCATAACAAGTTCTATT
TGTTTACGCCAGACAGACCTAAAGTCCCTAATTGCATACTCCTCTGTTAGTCACATAGCCCTA
GTCATTGTAGCAATTCTAATCCAATCCCCATGAAGCTACATAGGAGCTACTGCTCTAATAAT
CGCCCATGGGTTAACATCTTCTATGCTATTCTGTCTTGCAAACTCTAACTACGAGCGAACCCA
TAGCCGAACTATAATTCTCGCACGGGGCTTGCAAACGCTTCTTCCACTTATAGCAGCTTGAT
GACTATTAGCAGGCCTAACTAACTTAGCTCTTCCTCCTACAATTAATCTGATTGGGGAACTGT
TTGTAGTGATAGCCACATTTTCATGATCCAATATTACCATTCTCTTAATAGGAGTTAACATTA
CCATTACTGCTCTCTACTCTCTATATATACTAATTTCTACACAGCGAGGCAAGTATACAGACC
ACATCAAAAATATTAAGCCATCTTATACACGAGAAAATGCCTTAATAGCCTTACATTTACTA
CCCCTTTTACTTCTATCTCTTAACCCTAAAGTAGTTCTAGGCTTTATTTACTGTAAATATAGTT

TAACAAAAACATTAGATTGTGAATCTAGTAATAAAAGCTTAAATCTTTTATTTACCGAAAA
AGCATGCAAGAACTGCTAATTCATGCACCCATGCATAAAATCATGGCTTTTTCAACTTTTAG
GGGATAGAAGTAATCCATTGGTCTTAGGAGCCAAAAAATTGGTGCAACTCCAAATAAAAGT
AATTAACCTACTTACCTCCTTCACCATTACAACCCTATTTATATTAATCATGCCCATTATCCT
AACCTGTACCCCAGCATATGAAAATAAACTTTACCCGCAATATGTAAAAACCACAGTCTCTT
ATGCCTTCACAATTAGCATAATTCCCATAGTAATATTCATCCACTCCGGACAAGAAGCAATC
ATCTCAAACTGACACTGAATAACCATCCAAACCCTAAAACTCAGTCTAAGCTTCAAATTAGA
TTACTTTTCAATAATCTTCGTACCAGTGGCCCTATTCGTCACATGGTCAATTATAGAATTCTC
TATATGATATATACACGCAGATCCTAACATTAACCGATTCTTTAAATATCTACTTATATTCCT
CGTCACGATAATAATTCTAGTAACCGCAAATAATCTATTTCAGCTATTTATCGGCTGAGAGG
GAGTAGGAATCATATCATTTCTCCTCATTGGATGATGATATGGACGAACAGATGCTAATACG
GCTGCCCTACAAGCAGTACTATACAACCGCATTGGAGATGTTGGTTTTATCATGGCCATAGC
TTGATTCTTACTCAATACAAATGCATGGGACTTCCAACAAATCTTTATAATCGAGCATAAAA
ACCTCAATATTCCACTGGCAGGACTACTTCTAGCAGCTACCGGAAAGTCTGCCCAATTTGGA
CTTCACCCATGACTCCCATCAGCCATAGAAGGCCCCACTCCTGTCTCAGCACTACTTCACTCT
AGCACAATAGTAGTAGCAGGCGTATTTCTTCTAGTCCGATTTCACCCCTTAATAGAACATAA
CAAGACAATTCAAACGGTTACCCTATGCTTAGGGGCAATAACAACCTTGTTTACAGCAATCT
GCGCCCTTACCCAAAATGATATCAAAAAAATTATTGCCTTCTCCACCTCGAGCCAACTAGGA
CTAATAATTGTAACAATTGGTATTAATCAGCCTCATCTGGCCTTTCTCCATATCTGCACGCAC
GCATTCTTCAAGGCCGTGCTATTTCTATGCTCCGGATCTATTATTCATAGTCTAAACAACGAA
CAAGATATTCGAAAGATAGGAGGGCTATTTAAAAGTCTACCATTTACCACTACTGCTCTAAT
CATCGGAACTCTAGCACTTACAGGAATGCCCTTCTTAACAGGATTTTACTCCAAAGACTTAA
TTATTGAAACCGCCAATACGTCATACACCAACGCCTGAGCCTTACTAACAACCCTTATCGCC
ACATCCATAACTGCCGCCTACAGTACTCGAATTGTATTTTTTACACTTCTAGGACAACCTCGC
TATAGTCCCATTATCACCATTAACGAGAATAACCAGTTCCTGACTAACTCTGTCAAACGATT
GCTACTAGGAAGTATCTTTGCGGGATTCCTAATCTCTCATAACATTACACCCACCACTGTCCC
ACAAATAACCATGCCCTGTCACCTAAAAATGATAGCCCTTACAGTAACAATCCTAGGCTTTA
TTCTAGCACTAGAACTTAACCTAGCCATACAAAATCTCAAATTTGACTACCCTTTAAGCACG
TTCAAATTTTCAAATCAACTAGGATATTTCCCTACTACTCTGCATCGTCTCGCACCAATAATA
GGTCTATCAATAAGCCAAAAAACAGCATCCATACTGCTAGATGCAATCTGACTAGAGAATGT
TCTACCAAAGTCCATCTCCTACTTTCAAATAAAATCCTCAATCACAGTCTCCAACCAAAAGG
GACTAATTAAACTATACTTTCTCTCCTTTATAATTACACTAGTCTTAAGTCTCTTTATACTTAA
TTTCCACGAGTGACTTCCATAATTACCAATACACCAATAAGGAGAGATCATCCAGTAACAAT
AACTAATCAAGTACCATAACTATATAAAGCTGCAATTCCCATAGCCTCTTCACTAAAAAATC
CTGAATCCCCTGTGTCATAAATCACCCAATCTCCTATTCCATTAAACTTAAATACTACCTCGG
CTTCATCATCCTTTAAAATATAACAAACAGCCAGTAACTCAGACAACAGACCTACAATAAAT
GCACCTAGTACAGCCTTATTAGAAAGTCAAACCTCAGGATATTGCTCAGTGGCTATGGCCGT
AGTGTACCCGAACACTACAAGTATACCCCCTAAATAAATTAAAAAGACCATTAAACCCAAA
AAAGATCCTCCAAAGCTTAACACAATACCACACCCTACACCCCCACTAACAATCAAGACTAA
ACCTCCGTAGATAGGAGAGGGCTTAGAAGAAAACCCCGCAAAACTAACTACAAAAACGATA
CTTAAAATAGATACAATGTATGTTATCATCATTCTTACATGGAATCTAACCATGACCAATGA
CATGAAAAATCATCGTTGTATTTCAACTATAAGAACCCCAATGATCAACATCCGAAAAACTC
ATCCATTAGTTAAAATTATCAACAACTCATTCATTGACCTTCCAACACCATCAAACATTTCAA
CATGATGAAACTTTGGGTCCCTGTTAGGAGTGTGTCTGATCTTGCAAATCTTAACAGGCTTAT
TTCTAGCCATACACTATACATCAGATACAGCTACAGCCTTTTCATCAGTCGCACACATTTGTC
GAGACGTCAACTATGGTTGATTTATCCGATATATACATGCCAATGGGGCCTCTATATTTTTTA
TCTGCCTATTTATACACGTAGGGCGAGGCTTATACTATGGATCATACCTATTTCCAGAGACAT
GGAATATCGGAATTATTCTCCTACTTACAGTTATAGCCACAGCATTCATAGGGTATGTACTA
CCTTGAGGACAAATATCCTTCTGAGGAGCAACCGTCATTACTAACCTACTATCAGCAATTCC
TTACATTGGCACTAATCTAGTGGAGTGAGTCTGAGGGGGTTTCTCCGTAGATAAAGCAACAC
TAACCCGATTTTTTGCTTTTCACTTTATCCTTCCATTTATCATCTCAGCACTAGCAATAGTCCA

TCTATTATTCCTTCACGAAACAGGATCTAATAACCCCTCCGGAATTCCATCTGACCCAGACA
AAATCCCATTTTACCCCTATCATACAATTAAAGACATCCTAGGCGTCCTATTTCTTGTCCTCG
CCTTAATAACCCTGGCTTTATTCTCACCAGACCTGTTAGGAGACCCTGATAACTATACCCCTG
CAAATCCACTAAGTACCCCGCCACATATTAAGCCTGAATGGTACTTTCTATTTGCCTACGCTA
TCCTGCGATCTATTCCTAATAAACTAGGAGGGGTGCTAGCTCTAATCTTCTCTATTCTAATTC
TAACTATTATTCCACTATTACATACATCCAAACAACGAAGCATGATATTCCGACCTCTAAGT
CAATGCTTATTCTGACTCCTAGTAGCAGACCTACTCACACTAACATGAATTGGAGGACAGCC
AGTAGAACACCCCTTCATTATTATTGGGCAATTGGCCTCTATTCTCTACTTTACAATTCTTCT
AGTACTTATACCTATCACTAGCATTATTGAGAATAGCCTCTCAAAATGAAGAGTCTTTGTAG
TATATTAATTACTTTGGTCTTGTAAACCAAAAACGGAGATTCCTCTACTTCTCCCTAAGACTC
AAGGAAGGAGCAACAACCCCACTACCAGCACCCAAAGCTGGTGTTCTACTTAAACTATCCCC
TG
>SRR6109211 (circular) ReOrganized
ATACTATAAATCCACCTCTCATTTTATTCACTTCATACATGCTATTACACACTCTGTGCCATC
ATAGTATGTTTTCATACATCCTCCCTTCTTTCACACCCTATGTATATCGTACATTAATGGTGTA
CCCCCCCCTCCCCCCTATGTATATCGTGCATTAATGGCGTGCCCCATGCATATAAGCATGTAC
ATACTGTGCTTGGCTTTACATGAGGATACTCATTACAAGAACTTATTTCAAGCGATAGTCTAT
GAGCATGTATTTCACTTAGTCCAAGAGCTTGATCACCAAGCCTCGAGAAACCAGCAATCCTT
GCGAGTACGTGTACCTCTTCTCGCTCCGGGCCCATAATTTGTGGGGGTTTCTATACTGAAACT
ATACCTGGCATCTGGTTCTTACCTCAGGGCCATGTTAGCGTCAACTCAATCCTACTAACCCTT
CAAATGGGACATCTCGATGGACTAATGACTAATCAGCCCATGATCACACATAACTGTGGTGT
CATGCATTTGGTATTTTTTAATTTTAGGGGGGGAACTTGCTATGACTCAGCTATGACCGTAAA
GGTCTCGTCGCAGTCAAATCAGCTGTAGCTGGGCTTATTCATCTTTCGAGGCTCCTCATGGAT
ACCCATAAGGTGCAATTCAGTCAATGGTCACAGGACATAACACTATAGATCACCCGGACTG
GCGTTACGTGTACGTACGTGTACGTACGTGTACGTACGTGTACGCACGTGTACGTACGTGTA
CGCACGTGTACGTACGTGTACGCACGTGTACGCACGTGTACGCACGTGTACGCACGTGTACG
CACGTGTACGTACGTGTACGCGTACGTATTTTAGATACTAAGTTAGCTTAGACAAACCCCCC
TTACCCCCCGTAACTTCAAGAAGCTTACATATACTTATGGATGTCCTGCCAAACCCCAAAAA
CAAGACTAAATATATGCGCAAACATGAAGTCACTTACACCTAAACCCATATAATTAAGCTAA
CCCCCCAGCCAATGTTGCAACAACTACGGACATGGGACTCTAAATTTTAATTTATCTATAGA
TATTTTTCTTTTACTGTGTCTCCCCAGCATTGATTTTTTAATTATCATTATTCCACACCACCAA
TTTCCATTGAGCTATTTCACATGAGTTCCAAATCAATTATGTTCATGTAGCTTAACGAATAAA
GCAAGGTACTGAAAATGCCTAGATGGGTCACGCTACCCCATAGACATAAAGGTTTGGTCCTA
GCCTTCCTATTAGCCATTAACAAGATTACACATGTAAGTCTCCACGCTCCAGTGAAAATGCC
CCTTAAGTCCTCTTAGACGACCTAAAGGAGCGGGTATCAAGCACACCTTATGGTAGCTCACA
ACGCCTTGCTTAGCCACACCCCCACGGGAAACAGCAGTGATAAAAATTAAGCTATGAACGA
AAGTTCGACTAAGCTATGTTAATACTAGGGTTGGTAAATCTCGTGCCAGCCACCGCGGTCAT
ACGATTAACTCGAGTTAATAGGCCTACGGCGTAAAGCGTGTAAAAGAAAAATCTCCTCTAC
TAAAGTTAAAGTATGATTAAGCTGTAAAAAGCTACCATTAATACTAAAATAAACTACGAAA
GTGACTTTAAAATTTCTGATTACACGATAGCTAGGGCCCAAACTGGGATTAGATACCCCACT
ATGCCTAGCTCTAAACATAGATATTTTACTAAACAAAACTATTCGCCAGAGAACTACTAGCA
ACAGCTTAAAACTCAAAGGACTTGGCGGTGCTTTATATCCCCCTAGAGGAGCCTGTTCTGTA
ATCGATAAACCCCGATAGACCTCACCATCCCTTGCTAATTCAGTTTATATACCGCCATCTTCA
GCAAACCCTTAAAAGGAAAAAAAGTAAGCATAACTACCCTACATAAAAAAGTTAGGTCAAG
GTGTAACCTATGGGCTGGGAAGAAATGGGCTACATTTTCTATTCAAGAACAACTTCTACGAA
AACTTTTATGAAACTAAAAGCTAAAGGCGGATTTAGTAGTAAATTAAGAATAGAGAGCTTA
ATTGAACAGGGCAATGAAGCACGCACACACCGCCCGTCACCCTCCTCGAGTGATATAATTTA
ATTATAACCTATTTAAACTAAGCAAAGCATAAGAGGAGACAAGTCGTAACAAGGTAAGCAT
ACTGGAAAGTGTGCTTGGATGAGCCAAAGTGTAGCTTAAACAAAGCGTCTGGCTTACATCCA
GAAGATTTCATTAATATATGACTACTTTGAACCCAAAGCTAGCCCAAGCAACAATGACTAGT
AAAACCATTATGAAACATTCAAACAAAACATTTAGTAGCATGACTAGAGTATAGGAGATAG

AAATTTTTAACTGGAGCTATAGAGAGAGTACCGCAAGGGAATGATGAAAGATTACCTAAAG
TGATAAACAGCAAAGATTGCCCCTTCTACCTTTTGTATAATGAGTTAGCTAGAAATAACTTA
ACAAAGAGAACTTAAGCTAAGTCCCCCGAAACCAGACGAGCTACCTATGAACAATCCACTG
GGATGAACTCATCTATGTTGCAAAATAGTGAGAAGATCCATAGGTAGAGGTGAAAGGCCTA
ACGAGCCTGGTGATAGCTGGTTGCCCAGAATAGAATTTTAGTTCGACTTTAAACCTGCCTAC
AAAACTAATAATTCTAATGCAGATTTAAAATATATTCTAAAAAGGTACAGCTTTTTAGAGTT
AAGGATACAGCCTTACTTAGAGAGTAAATATTTATATAAGCCATAGTAGGCCTAGAGGCAG
CCATCAATTAAGAAAGCGTTAAAGCTCAACATCTCTATTAACTTAATACCAAGAATATTTAA
TCAACTCCTAATGTATTACTGGGTCAATCTATTTAAATATAGAAGTGATAATGCTAATATGA
GTAACAAGAAATATTTCTCCCATGCATAAGCTTATAACAGCAACGGATAACCACTGATAGTT
AACAACAACATAGAAATAACCTAATGATAAAACACCTATTAAATCAATTGTTAGTCCAACAC
AGGCATGCAATCAGGGAAAGATTAAAAGAAGTGAAAGGAACTCGGCAAATACAAACCCCG
CCTGTTTACCAAAAACATCACCTCCAGCATTTCCAGTATTGGAGGCACTGCCTGCCCGGTGA
CATCAGTTAAACGGCCGCGGTATTCTGACCGTGCAAAGGTAGCATAATCATTTGTTCTCTAA
ATAAGGACTTGTATGAATGGCCACACGAGGGTTTAACTGTCTCTTACTTCCAATCAGTGAAA
TTGACCTCCCCGTGAAGAGGCGGGGATAAGACAATAAGACGAGAAGACCCTATGGAGCTTT
AATTAACTAATTCAAAAAGAAACTACTAACGACCCAACAGGAATAATATATCTCTTTTATGA
ATTAGCAATTTAGGTTGGGGCGACCTCGGAGGACAAAATAGCCTCCGAGTGATTATAAATCT
AGACTTACCAGTCAAAATGCTTAATCACTTATTGATCCAAAAATTCTTTTGATCAACGGAAC
AAGTTACCCTAGGGATAACAGCGCAATCCTATCCGAGAGTCCATATCGACAATAGGGTTTAC
GACCTCGATGTTGGATCAGGACATCCTAATGGTGCAGCAGCTATTAAAGGTTCGTTTGTTCA
ACGATTAAAGTCCTACGTGATCTGAGTTCAGACCGGAGCAATCCAGGTCGGTTTCTATCTAT
TCAAATAATTTCTCCCAGTACGAAAGGACAAGAGAAATAAGGCCTACTTCTCTGAAGCGCCT
TAAGACCAATAGATGAATTTATCTAAATCTAGTAAATCTAACTCCAATATTGCCCAAGAGAC
AGGGCTTTGTTAGGGTGGCAGAGCCCGGTAATTGTGCAAAACTTAAACTCTTGTGTCCAGAG
GTTCAATTCCTCTCCCTAGCATATGTTTATAATTAACATCTTCTCACTAATTGTACCTATTCTT
CTTGCTGTAGCCTTTCTGACTCTAGTAGAACGAAAAGTACTAGGCTACATACAGCTTCGAAA
AGGCCCAAATATTGTAGGTCCATATGGTCTCCTACAACCAATCGCAGATGCCGTAAAACTCT
TTACTAAAGAACCTTTACGACCACTTACGTCATCCATAACTATATTTATTATAGCCCCTATTC
TAGCCTTAACACTAGCTCTGACCATGTGAGTCCCCTTACCAATACCGCACCCCCTTATTAACA
TAAACCTAGGAGTACTATTCATACTAGCTATATCAAGTCTAGCTGTATACTCTATTCTCTGAT
CTGGATGAGCCTCAAACTCAAAATATGCCCTAATTGGAGCCCTACGAGCCGTAGCTCAAACG
ATCTCATATGAAGTCACACTAGCCATTATCCTTTTATCCGTTCTACTAATAAATGGCTCATTT
ACGCTATCCACGCTAATCATTACCCAAGAACATCTTTGATTAATCTTCCCTGCATGACCACTA
GCTATAATATGATTTATTTCTACCCTAGCAGAAACAAATCGAGCCCCCTTTGACCTAACAGA
AGGAGAATCAGAACTAGTCTCTGGGTTTAATGTCGAATATGCAGCAGGTCCATTTGCCCTGT
TCTTCCTAGCAGAATATGCCAACATCATCATAATAAATGCTCTTACAACTATTCTATTCTTCG
GAGCATTTCACAGCCCCTACATACCAGAATTATACATTGTTAATTTTACCGTAAAAACTTTAC
TCCTCACAATCTCTTTTCTATGAATCCGAGCATCATATCCTCGATTCCGATACGACCAACTAA
TACACCTACTATGAAAAAACTTTCTACCTCTCACACTAGCTCTATGTATATGACATGTAACTC
TACCTATTATTACAGCAAGCATTCCACCTCAAACGTAAGAAATATGTCTGACAAAAGAATTA
CTTTGATAGAGTAAGCCATAGGGGTTTAAGTCCCCTTATTTCTAGAATCATAGGAATCGAAC
CTAATCTTAAGAACTCAAAAATCTTCGTGCTACCTAACTACACCATATCCTAAAGTAAGGTC
AGCTAAATAAGCTATCGGGCCCATACCCCGAAAATGTTGGTTTATCCCCTTCCCATACTAAT
AAAACCAGCCATCCTTATTACCATTACATCCACCGTTGTCTTAGGAACTATAATTGTTCTATT
TAGCTCCCACTGATTCATAATCTGAGTTGGCTTTGAAATAAATATACTAGCCATTATCCCAAT
TCTAATAAAAAAATATAACCCACGAGCCATAGAAGCTTCCACAAAATATTTTCTAACACAAG
CTACAGCATCCATGCTTCTAATGCTAAGCATCATCATCAATCTACTATGCTCAGGACACTGG
ACAGTTTCAACAATTCCCAATCCAGTAGCATCTACCATAATTACTATTGCTCTAACAATAAA
ACTTGGCCTATCACCATTTCACTTTTGAGTCCCAGAAGTTACACAAGGCATTTCCTTATCTTC
AGGAATAATCCTATTAACATGACAAAAAATTGCACCTTTATCCATTCTATACCAAATTTCAC

CATCCGTTAACTCAAATCTCTTACTAATAATAGCTATCACATCCATACTGGTAGGAGGCTGA
GGAGGATTAAATCAAACCCAACTCCGAAAAATCTTGGCATACTCCTCAATTACCCATATAGG
CTGAATGGCTGCTATTATAGTATATAATCCTACCCTAGCAATTCTTAACCTAACAATTTACAT
CATAATAACTCTAGGAACATTTATACTATTCATACATAGCTCATCTACAACAACACTATCACT
ATCTTATACATGAAATAAATTCCCATTAATAGCCCCACTAATCTTAATACTTATACTATCACT
AGGAGGCCTCCCTCCTCTCTCAGGCTTTATCCCCAAATGAATAATTATTCAAGAATTAACAA
AAAACGACATAATTATTATACCTACACTTATAGCCATTACAGCCCTACTAAACCTATATTTTT
ACACACGCCTAACATATACTACAGCACTAACTATATTTCCCGCAACAAATAATATAAAAATA
AAATGACAATTCGAAACCACAAAAAAGACAACTCTCCTAGCCCCTCTAATCGTGACATCAAC
TATACTACTACCACTAACCCCAATACTAGCAGCTCTTGATTAGAGATTTAGGCTAAACAGAC
CAAGGGCCTTCAAAGCCTCAAGTAAGTGAAAATCCACTTAATCTCTGCAATATTTAAGGACT
GCAAGAATCTACCTCACATCAACTGAACGCAAAACAATCGCTTTAATTAAGCTAAGCCCTTC
TAGATCGGTGGGCTTTTACCCCACAAAACTTTAGTTAACAGCTAAAAACCCTAGACAACTGG
CTTCAATCTACTTCTCCCGCCGCGTAGGAAAAAAAGGCGGGAGAAGCCCCGGCAGAATTGA
AGCTGCTTCTTTGAATTTGCAATTCAATATGATTACTCACCACAGAGCCTGGTAAAAAGAGG
ACTTAACCCCCATTCTTAGATTTACAGTCTAGTGCTTTTATCAGCCATTTTACCTATGTTCATT
AACCGATGACTGTTTTCCACCAACCACAAAGATATTGGCACTCTTTATCTTCTGTTTGGCGCA
TGAGCTGGTATAACGGGCACTGCTCTTAGCCTTCTAATCCGTGCTGAATTAGGTCAGCCTGG
AGCTCTGTTAGGAGATGACCAAATTTACAATGTAGTTGTAACTGCCCACGCATTCGTAATAA
TCTTCTTTATAGTTATGCCTATTATGATCGGAGGCTTTGGAAATTGACTAGTACCTTTGATAA
TTGGTGCGCCTGATATAGCATTTCCCCGAATAAACAATATGAGTTTCTGATTACTACCTCCAT
CCTTTCTTCTCCTTCTGGCTTCTTCCATAGTAGAAGCAGGCGCAGGGACTGGATGAACTGTAT
ACCCTCCTCTAGCAGGTAACTTAGCCCATGCAGGAGCATCAGTAGACCTAACAATCTTTTCT
CTACATCTGGCAGGGGTTTCTTCTATTTTAGGGGCTATTAACTTTATTACCACTATCATCAAC
ATGAAACCCCCTGCAATATCTCAATATCAAACTCCCCTATTCGTGTGATCAGTACTTATCACA
GCAGTACTTCTCCTTCTATCTTTACCAGTCTTAGCAGCTGGAATTACTATACTACTTACAGAT
CGAAATCTCAATACTACTTTCTTCGACCCGGCCGGAGGAGGGGATCCCATTCTATATCAACA
TCTATTTTGATTCTTTGGCCATCCTGAAGTCTATATTCTAATCCTCCCTGGGTTTGGAATAATT
TCACACATTGTCACATACTATTCAGGAAAGAAAGAGCCCTTTGGTTATATGGGAATAGTGTG
GGCAATAATGTCAATTGGATTCTTAGGATTTATTGTATGGGCCCATCATATATTTACTGTAGG
CATAGATGTTGATACACGAGCCTATTTTACCTCGGCCACTATAATTATTGCAATCCCGACAG
GAGTTAAAGTATTCAGCTGACTAGCTACCCTGCATGGAGGAAATATTAAGTGATCCCCTGCC
ATAATGTGAGCCCTAGGTTTCATTTTTCTATTTACAGTAGGGGGTCTTACAGGCATTGTTCTA
GCTAACTCATCTCTGGACATTGTCCTCCATGACACATACTACGTAGTAGCACACTTCCACTAT
GTACTATCAATAGGGGCCGTGTTCGCTATTATAGGAGGCTTCGTCCATTGATTCCCATTATTT
TCGGGCTATACACTTAATAATACATGAGCAAAAATTCACTTCGTAATTATATTTGTTGGAGTC
AATATAACATTCTTCCCCCAGCACTTCCTAGGTCTATCAGGTATGCCTCGACGATATTCTGAT
TATCCAGACGCCTATACAGCATGAAACACAGTGTCTTCAATGGGTTCATTCATTTCACTAAC
AGCAGTTATGTTAATAATTTTCATAATTTGAGAAGCCTTCGCATCTAAACGAGAAGTGGCAG
TGATTGAGCTTACTACAACTAATATTGAATGGCTCCACGGATGCCCTCCCCCATACCATACA
TTCGAAGAGCCCGCCCACGTCATACTGAAATAAGAAAGGAAGGAATCGAACCCTCTAGAAT
TGGTTTCAAGCCAGCATCATAACCACTATGTCTCTCTCAATAAAGAGGTATTAGTAAAAATT
ACATAACTTTGTCAAAGTTGAATTATAGGTGAAAATCCTTTATATCTCTATGGCGCATCCCTT
TCAAACAGGCCTTCAAGATGCAACTTCCCCCATCATGGAAGAACTCCTACACTTCCATGATC
ATACACTAATAATTGTATTCTTAATTAGTTCCTTAGTTCTCTATATCATTTCAATTATATTAAC
TACTAAATTAACACATACAAATACAATAGACGCACAAGAAGTGGAGACAGTATGAACTATT
CTACCAGCTATCATCTTAATTATAATTGCACTTCCATCACTACGAATTCTTTATATGATAGAT
GAAATTAATAACCCATCATTAACTGTAAAGACTATGGGGCATCAATGATACTGAAGCTATGA
GTATACAGATTATGAAGACTTGAGCTTCGATTCTTATATAACCCCAACACAAGAATTAAAGC
CCGGAGAGCTGCGATTACTAGAGGTAGATAATCGAGTAGTACTACCCATAGAAATAACAAT
TCGCATATTAATTTCCTCAGAAGATGTGCTACATTCGTGAGCTGTCCCATCTCTAGGACTAAA

AACTGATGCAATCCCGGGGCGATTAAATCAAACAACTCTTATGGCCATGCGACCAGGACTAT
ATTATGGCCAATGCTCAGAGATTTGCGGTTCTAATCATAGCTTTATACCCATTGTACTTGAAA
TAGTTCCGCTGTCTTACTTTGAAAAATGATCCGCTTCAATACTATAAAATCATTAAGAAGCTT
TATAGCGTTAACCTTTTAAGTTAAAGACTGAGAGTGCAAGTCTCTCCTTAATGGAAATGCCA
CAATTAGACACATCCACATGATTTATTACAATCCTATCCATGATCTTAACACTATTTATTATA
TTTCAGTTAAAAGTCTCAAAACACAACTATCCTGAGTTTCCTGAATTAAAAACAACCCCACT
ATCAAAAAAACCTATGCCTTGAGAAGAAAAATGAACGAAAATCTGTTCGCCTCTTTTACTAC
CCCAATGATAATAGGAGTACCAATTGTGGTTCTGATTATTATTTTCCCAAGTATCTTATTTCC
CTCACCTAATCGATTAATCAATAACCGCCTAGCTACTGTCCAACAATGATTAATTCAATTAGT
ATCTAAGCATATACTGTCTATTCACAATTATAAAGGACAAACCTGAGCGCTAATACTAATGT
CATTAATTTTATTTATTGGCTCAACTAACCTTTTAGGCCTATTACCACATTCATTCACACCTAC
TACACAACTATCAATAAATTTAGGAATGGCCATTCCTCTGTGAGCAGGTACAGTAGTTATAG
GATTTCGACACAAAACTAAGGCATCTCTGGCCCACTTTCTACCCCAAGGAACACCTCTTCCT
CTGATCCCAATGTTAGTAATTATCGAGACAATTAGTCTGTTTATTCAACCTGTGGCCCTAGCT
GTACGACTGACCGCTAATATTACTGCAGGCCATCTACTTATTCATTTAATCGGAGGGGCTAC
CCTAGTTTTAATAAACATTAACCCTGTAACAGCCCTAATTACCTTTATTATTCTTATTCTACTT
ACTATTCTCGAATTAGCTGTAGCTCTTATCCAAGCCTATGTTTTCACCCTACTAGTTAGTCTA
TACTTACATGATAATACCTAATGATCCACCAAACACATGCATATCATATAGTTAATCCAAGC
CCATGGCCACTTACAGGAGCTCTCTCGGCTCTTCTTATAACATCAGGACTAATTATATGATTT
CACTTTAACTCGGTATACTTACTAATACTAGGCCTCACCACCAACATACTTACCATGTACCAA
TGATGACGAGATATCGTCCGGGAAAGTACCTTCCAAGGCCATCACACCCCAATCGTCCAAAA
AGGACTACGATATGGAATAGTCTTATTTATTGTATCAGAAGTATTTTTCTTCGCGGGATTCTT
TTGGGCCTTCTACCACTCAAGCCTAGCACCTACTCCTGAATTAGGAGCATGTTGACCACCTA
CAGGTATCATCCCCTTAAATCCGCTAGAAGTTCCACTACTTAACACCTCTGTACTTCTAGCAT
CTGGGGTATCTATCACTTGAGCTCATCATAGTCTAATAGAAGGAGACCGCAAACATATGCTC
CAAGCCCTATTTATTACTATTTCACTAGGAGTTTATTTTACACTTCTACAAGCCTCAGAATAC
TATGAGACATCCTTTACTATTTCAGATGGAGTGTATGGTTCAACCTTCTTTATAGCAACTGGA
TTTCATGGGCTTCATGTAATTATTGGTTCAACTTTTCTTACTGTGTGTTTTCTACGACAACTGC
ACTTTCACTTTACATCCAACCATCACTTTGGCTTTGAAGCGGCAGCATGATACTGACACTTCG
TAGACGTTGTATGGCTTTTCCTTTATGTTTCTATTTACTGATGAGGATCCTACTTCTTTAGTAT
TAACTAGTACGATTGACCTCCAATCAATAGGCTCTGGTACAAGCCAGAAAGAAGTAATAAA
CCTAATTCTAGCACTTCTAACCAACGTATTATTAGCCTCTCTGCTCGTTCTTATTGCATTCTGA
CTGCCACAACTAAATATCTATGCAGAAAAAGTAAGTCCTTATGAATGTGGATTTGATCCTAT
AGGATCAGCACGCCTTCCTTTCTCTATAAAATTTTTTCTAGTAGCTATTACATTCCTTTTATTC
GACTTAGAAATTGCTCTACTCCTCCCGCTTCCTTGAGCATTACAGACTAACAACTTAATAACT
ATACTTATTATAGCTCTATTACTCATTTCCCTATTAGCCGCAAGCCTAGCTTATGAGTGAACC
GAAAAGGGGCTAGAATGGGCCGAATATGATAATTAGTTTAAACTAAAATAAGTGATTTCGA
CTCACTAGATTATGACTAACATCATAATTATCAAATGTCTGTGGTCTATATTAATCTCTTTCT
AGCCTTTACTATATCCTTAATAGGGCTACTCATCTATCGATCCCATTTAATATCCTCCCTATT
ATGCTTAGAAGGCATGATGCTGTCACTATTTGTTATACTAGCAGTAACAATCCTAAGTAACC
ACTTCACACTAGCCAGTATGACTCCAATCATTCTATTAGTATTCGCTGCCTGTGAAGCAGCGT
TGGGGCTTTCTCTGCTAGTAATAGTCTCTAATACCTATGGAACTGACTACGTACAAAATCTTA
ACCTCCTACAATGCTAAAAATTATCCTCCCTACCATAATACTAGTACCCCTTACATGAATATC
AAAGCCTAACATAATCTGAATTAATACAACAACCTATAGTCTACTCATCAGCCTTATTAGTC
TGACATATCTCAATCAACTCAGTGATAATAGTCTAAACTTCTCATTAACATTCTTTACCGACT
CCCTATCAGCCCCCCTGTTAGTTCTCACAACATGACTACTGCCCTTAATACTTATAGCTAGTC
AATTTCACCTGTCAAAAGAGACCCTGACCCGGAAAAAATTATATATCACAATACTAATTCTC
CTGCAACTATTCTTAATTATAACATTTTCTGCTACTGAGTTAATCATATTCTATATTTTATTTG
AAGCTACCCTGGTACCGACTTTAGTTATCATTACTCGATGAGGAAACCAAACAGAACGACTA
AACGCGGGCCTCTATTTTTTATTCTACACCTTAGTAGGATCCTTGCCCTTACTAGTTGCACTG
TTATATGCCCAAAACAGCTTAGGATCACTAAATTTTCTCATAGTACAGTACTGAACTCAGCC

CTTGCCAAATTCCTGATCTAACATTTTCATGTGGCTGGCATGTATAATAGCATTCATAGTAAA
AATACCGCTATATGGCTTACACCTATGACTACCTAAAGCACATGTAGAAGCTCCTATTGCAG
GATCCATAGTACTTGCTGCAGTACTTCTAAAACTAGGGGGATACGGTATAATGCGGATTACA
ACAATACTCAACCCTCTAACAAATTTTATAGCCTACCCCTTCATAATGCTCTCACTATGAGGA
ATAATCATAACAAGTTCTATTTGTTTACGCCAGACAGACCTAAAGTCCCTAATTGCATACTCC
TCTGTTAGTCACATAGCCCTAGTCATTGTAGCAATTCTAATCCAATCCCCATGAAGCTACATA
GGAGCTACTGCTCTAATAATCGCCCATGGGTTAACATCTTCTATGCTATTCTGTCTTGCAAAC
TCTAACTACGAGCGAACCCATAGCCGAACTATAATTCTCGCACGGGGCTTGCAAACGCTTCT
TCCACTTATAGCAGCTTGATGACTATTAGCAGGCCTAACTAACTTAGCTCTTCCTCCTACAAT
TAATCTGATTGGGGAACTGTTTGTAGTGATAGCCACATTTTCATGATCCAATATTACCATTCT
CTTAATAGGAGTTAACATTACCATTACTGCTCTCTACTCTCTATATATACTAATTTCTACACA
GCGAGGCAAGTATACAGACCACATCAAAAATATTAAGCCATCTTATACACGAGAAAATGCC
TTAATAGCCTTACATTTACTACCCCTTTTACTTCTATCTCTTAACCCTAAAGTAGTTCTAGGCT
TTATTTACTGTAAATATAGTTTAACAAAAACATTAGATTGTGAATCTAGTAATAAAAGCTTA
AATCTTTTTATTTACCGAAAAAGCATGCAAGAACTGCTAATTCATGCACCCATGCATAAAAT
CATGGCTTTTTCAACTTTTAGGGGATAGAAGTAATCCATTGGTCTTAGGAGCCAAAAAATTG
GTGCAACTCCAAATAAAAGTAATTAACCTACTTACCTCCTTCACCATTACAACCCTATTTATA
TTAATCATGCCCATTATCCTAACCTGTACCCCAGCATATGAAAATAAACTTTACCCGCAATAT
GTAAAAACCACAGTCTCTTATGCCTTCACAATTAGCATAATTCCCATAGTAATATTCATCCAC
TCCGGACAAGAAGCAATCATCTCAAACTGACACTGAATAACCATCCAAACCCTAAAACTCA
GTCTAAGCTTCAAATTAGATTACTTTTCAATAATCTTCGTACCAGTGGCCCTATTCGTCACAT
GGTCAATTATAGAATTCTCTATATGATATATACACGCAGATCCTAACATTAACCGATTCTTTA
AATATCTACTTATATTCCTCGTCACGATAATAATTCTAGTAACCGCAAATAATCTATTTCAGC
TATTTATCGGCTGAGAGGGAGTAGGAATCATATCATTTCTCCTCATTGGATGATGATATGGA
CGAACAGATGCTAATACGGCTGCCCTACAAGCAGTACTATACAACCGCATTGGAGATGTTGG
TTTTATCATGGCCATAGCTTGATTCTTACTCAATACAAATGCATGGGACTTCCAACAAATCTT
TATAATCGAGCATAAAAACCTCAATATTCCACTGGCAGGACTACTTCTAGCAGCTACCGGAA
AGTCTGCCCAATTTGGACTTCACCCATGACTCCCATCAGCCATAGAAGGCCCCACTCCTGTCT
CAGCACTACTTCACTCTAGCACAATAGTAGTAGCAGGCGTATTTCTTCTAGTCCGATTTCACC
CCTTAATAGAACATAACAAGACAATTCAAACGGTTACCCTATGCTTAGGGGCAATAACAACC
TTGTTTACAGCAATCTGCGCCCTTACCCAAAATGATATCAAAAAAATTATTGCCTTCTCCACC
TCGAGCCAACTAGGACTAATAATTGTAACAATTGGTATTAATCAGCCTCATCTGGCCTTTCTC
CATATCTGCACGCACGCATTCTTCAAGGCCGTGCTATTTCTATGCTCCGGATCTATTATTCAT
AGTCTAAACAACGAACAAGATATTCGAAAGATAGGAGGGCTATTTAAAAGTCTACCATTTA
CCACTACTGCTCTAATCATCGGAACTCTAGCACTTACAGGAATGCCCTTCTTAACAGGATTTT
ACTCCAAAGACTTAATTATTGAAACCGCCAATACGTCATACACCAACGCCTGAGCCTTACTA
ACAACCCTTATCGCCACATCCATAACTGCCGCCTACAGTACTCGAATTGTATTTTTTACACTT
CTAGGACAACCTCGCTATAGTCCCATTATCACCATTAACGAGAATAACCAGTTCCTGACTAA
CTCTGTCAAACGATTGCTACTAGGAAGTATCTTTGCGGGATTCCTAATCTCTCATAACATTAC
ACCCACCACTGTCCCACAAATAACCATGCCCTGTCACCTAAAAATGATAGCCCTTACAGTAA
CAATCCTAGGCTTTATTCTAGCACTAGAACTTAACCTAGCCATACAAAATCTCAAATTTGACT
ACCCTTTAAGCACGTTCAAATTTTCAAATCAACTAGGATATTTCCCTACTACTCTGCATCGTC
TCGCACCAATAATAGGTCTATCAATAAGCCAAAAAACAGCATCCATACTGCTAGATGCAATC
TGACTAGAGAATGTTCTACCAAAGTCCATCTCCTACTTTCAAATAAAATCCTCAATCACAGT
CTCCAACCAAAAGGGACTAATTAAACTATACTTTCTCTCCTTTATAATTACACTAGTCTTAAG
TCTCTTTATACTTAATTTCCACGAGTGACTTCCATAATTACCAATACACCAATAAGGAGAGAT
CATCCAGTAACAATAACTAATCAAGTACCATAACTATATAAAGCTGCAATTCCCATAGCCTC
TTCACTAAAAAATCCTGAATCCCCTGTGTCATAAATCACCCAATCTCCTATTCCATTAAACTT
AAATACTACCTCGGCTTCATCATCCTTTAAAATATAACAAACAGCCAGTAACTCAGACAACA
GACCTACAATAAATGCACCTAGTACAGCCTTATTAGAAAGTCAAACCTCAGGATATTGCTCA
GTGGCTATGGCCGTAGTGTACCCGAACACTACAAGTATACCCCCTAAATAAATTAAAAAGAC

CATTAAACCCAAAAAAGATCCTCCAAAGCTTAACACAATACCACACCCTACACCCCCACTAA
CAATCAAGACTAAACCTCCGTAGATAGGAGAGGGCTTAGAAGAAAACCCCGCAAAACTAAC
TACAAAAACGATACTTAAAATAGATACAATGTATGTTATCATCATTCTTACATGGAATCTAA
CCATGACCAATGACATGAAAAATCATCGTTGTATTTCAACTATAAGAACCCCAATGATCAAC
ATCCGAAAAACTCATCCATTAGTTAAAATTATCAACAACTCATTCATTGACCTTCCAACACC
ATCAAACATTTCAACATGATGAAACTTTGGGTCCCTGTTAGGAGTGTGTCTGATCTTGCAAA
TCTTAACAGGCTTATTTCTAGCCATACACTATACATCAGATACAGCTACAGCCTTTTCATCAG
TCGCACACATTTGTCGAGACGTCAACTATGGTTGATTTATCCGATATATACATGCCAATGGG
GCCTCTATATTTTTTATCTGCCTATTTATACACGTAGGGCGAGGCTTATACTATGGATCATAC
CTATTTCCAGAGACATGGAATATCGGAATTATTCTCCTACTTACAGTTATAGCCACAGCATTC
ATAGGGTATGTACTACCTTGAGGACAAATATCCTTCTGAGGAGCAACCGTCATTACTAACCT
ACTATCAGCAATTCCTTACATTGGCACTAATCTAGTGGAGTGAGTCTGAGGGGGTTTCTCCG
TAGATAAAGCAACACTAACCCGATTTTTTGCTTTTCACTTTATCCTTCCATTTATCATCTCAGC
ACTAGCAATAGTCCATCTATTATTCCTTCACGAAACAGGATCTAATAACCCCTCCGGAATTC
CATCTGACCCAGACAAAATCCCATTTTACCCCTATCATACAATTAAAGACATCCTAGGCGTC
CTATTTCTTGTCCTCGCCTTAATAACCCTGGCTTTATTCTCACCAGACCTGTTAGGAGACCCT
GATAACTATACCCCTGCAAATCCACTAAGTACCCCGCCACATATTAAGCCTGAATGGTACTT
TCTATTTGCCTACGCTATCCTGCGATCTATTCCTAATAAACTAGGAGGGGTGCTAGCTCTAAT
CTTCTCTATTCTAATTCTAACTATTATTCCACTATTACATACATCCAAACAACGAAGCATGAT
ATTCCGACCTCTAAGTCAATGCTTATTCTGACTCCTAGTAGCAGACCTACTCACACTAACATG
AATTGGAGGACAGCCAGTAGAACACCCCTTCATTATTATTGGGCAATTGGCCTCTATTCTCT
ACTTTACAATTCTTCTAGTACTTATACCTATCACTAGCATTATTGAGAATAGCCTCTCAAAAT
GAAGAGTCTTTGTAGTATATTAATTACTTTGGTCTTGTAAACCAAAAACGGAGATTCCTCTAC
TTCTCCCTAAGACTCAAGGAAGGAGCAACAACCCCACTACCAGCACCCAAAGCTGGTGTTCT
ACTTAAACTATCCCCTG
>SRR6109216 (circular)
ATACTATAAATCCACCTCTCATTTTATTCACTTCATACATGTTATTACACACTCTGTGCCATC
ATAGTATGTTTTCATACATCCTCCCTTCTTTCACACCCTATGTATGTCGTACATTAATGGTGTA
CCCCCCCTCCCCCCTATGTATATCGTGCATTAATGGCGTGCCCCATGCATATAAGCATGTACA
TACTGTGCTTGGCTTTACATGAGGATACTCATTACAAGAACTTATTTCAAGCGATAGTCTATG
AGCATGTATTTCACTTAGTCCAAGAGCTTGATCACCAAGCCTCGAGAAACCAGCAATCCTTG
CGAGTACGTGTACCTCTTCTCGCTCCGGGCCCATAATTTGTGGGGGTTTCTATACTGAAACTA
TACCTGGCATCTGGTTCTTACTTCAGGGCCATGTTAGCGTCAACTCAATCCTACTAACCCTTC
AAATGGGACATCTCGATGGACTAATGACTAATCAGCCCATGATCACACATAACTGTGGTGTC
ATGCATTTGGTATTTTTTAATTTTTAGGGGGGGAACTTGCTATGACTCAGCTATGACCGTAAA
GGTCTCGTCGCAGTCAAATCAGCTGTAGCTGGGCTTATTCATCTTTCGAGGCTCCTCATAGAC
ACCCATAAGGTGCAATTCAGTCAATGGTCACAGGACATAACACTATAGATCACCCGGACTG
GCGTTACGTGTACGTACGTGTACGTACGTGTACGTACGTGTACGTACGTGTACGTACGTGTA
CGCACGTGTACGTACGTGTACGCACGTGTACGTACGTGTACGCACGTGTACGTACGTGTACG
TACGTGTACGTACGTGTACGCACGTGTACGTACGTGTACGCACGTGTACGTACGTGTACGTA
CGTGTACGCACGTGTACGCACGTGTACGCACGTGTACGCACGTGTACGCACGTGTACGCACG
TGTACGCACGTGTACGTACGTGTACGTACGTGTACGTACGTGTACGCGTACGTATTTTAGAT
ACTAAGTTAGCTTAGACAAACCCCCCTTACCCCCCGTAACTTCAAGAAGCTTACATATACTT
ATGGATGTCCTGCCAAACCCCAAAAACAAGACTAAATATATGCGCAAACATGAAGTCACTT
ACACCTAAACCCATATAATTAAGCTAACCCCCCAGCCAATGTTGCAACAACTACGGACATGG
GACTCTAAATTTTAATTTATCTATAGATATTTTTCTTTTACTGTGTCTCCCCAGCATTGATTTT
TTAATTATCATTATTCCACACCACCAATTTCCATTGAGCTATTTCACATGAGTTCCAAATCAA
TTATGTTCATGTAGCTTAACGAATAAAGCAAGGTACTGAAAATGCCTAGATGGGTCACGCTA
CCCCATAGACATAAAGGTTTGGTCCTAGCCTTCCTATTAGCCATTAACAAGATTACACATGT
AAGTCTCCACGCTCCAGTGAAAATGCCCCTTAAGTCCTCTTAGACGACCTAAAGGAGCGGGT
ATCAAGCACACCTTATGGTAGCTCACAACGCCTTGCTTAGCCACACCCCCACGGAAACAGC

AGTGATAAAAATTAAGCTATGAACGAAAGTTCGACTAAGCTATGTTAATACTAGGGTTGGTA
AATCTCGTGCCAGCCACCGCGGTCATACGATTAACTCGAGTTAATAGGCCTACGGCGTAAAG
CGTGTAAAAGAAAAAATCTCCTCTACTAAAGTTAAAGTATGATTAAGCTGTAAAAAGCTACC
ATTAATACTAAAATAAACTACGAAAGTGACTTTAAAATTTCTGATTACACGATAGCTAGGGC
CCAAACTGGGATTAGATACCCCACTATGCCTAGCTCTAAACATAGATATTTTACTAAACAAA
ACTATTCGCCAGAGAACTACTAGCAACAGCTTAAAACTCAAAGGACTTGGCGGTGCTTTATA
TCCCCCTAGAGGAGCCTGTTCTGTAATCGATAAACCCCGATAGACCTCACCATCCCTTGCTA
ATTCAGTTTATATACCGCCATCTTCAGCAAACCCTTAAAAGGAAAAAAAGTAAGCATAACTA
CCCTACATAAAAAAAGTTAGGTCAAGGTGTAACCTATGGGCTGGGAAGAAATGGGCTACATT
TTCTATTCAAGAACAACTTCTACGAAAACTTTTATGAAACTAAAAGCTAAAGGCGGATTTAG
TAGTAAATTAAGAATAGAGAGCTTAATTGAACAGGGCAATGAAGCACGCACACACCGCCCG
TCACCCTCCTCGAGTGATATAATTTAATTATAACCTATTTAAACTAAGCAAAGCATAAGAGG
AGACAAGTCGTAACAAGGTAAGCATACTGGAAAGTGTGCTTGGATGAGCCAAAGTGTAGCT
TAAACAAAGCGTCTGGCTTACATCCAGAAGATTTCATTAATATATGACTACTTTGAACCCAA
AGCTAGCCCAAGCAACAATGACTAGTAAAACCATTATGAAACATTCAAACAAAACATTTAG
TAGCATGACTAGAGTATAGGAGATAGAAATTTTTAACTGGAGCTATAGAGAGAGTACCGCA
AGGGAATGATGAAAGATTACCTAAAGTGATAAACAGCAAAGATTGCCCCTTCTACCTTTTGT
ATAATGAGTTAGCTAGAAATAACTTAACAAAGAGAACTTAAGCTAAGTCCCCCGAAACCAG
ACGAGCTACCTATGAACAATCCACTGGGATGAACTCATCTATGTTGCAAAATAGTGAGAAG
ATCCATAGGTAGAGGTGAAAGGCCTAACGAGCCTGGTGATAGCTGGTTGCCCAGAATAGAA
TTTTAGTTCGACTTTAAACCTGCCTACAAAACTAATAATTCTAATGCAGATTTAAAATATATT
CTAAAAAGGTACAGCTTTTTAGAGTTAAGGATACAGCCTTACTTAGAGAGTAAATATTTATA
TAAGCCATAGTAGGCCTAGAGGCAGCCATCAATTAAGAAAGCGTTAAAGCTCAACATCTCT
ATTAACTTAATACCAAGAATATTTAATCAACTCCTAATGTATTACTGGGCCAATCTATTTAAA
TATAGAAGTGATAATGCTAATATGAGTAACAAGAAATATTTCTCCCATGCATAAGCTTATAA
CAGCAACGGATAACCACTGATAGTTAACAACAACATAGAAATAACCTAATGATAAAACACC
TATTAAATCAATTGTTAGTCCAACACAGGCATGCAATCAGGGAAAGATTAAAAGAAGTGAA
AGGAACTCGGCAAACACAAACCCCGCCTGTTTACCAAAAACATCACCTCCAGCATTTCCAGT
ATTGGAGGCACTGCCTGCCCGGTGACATCAGTTAAACGGCCGCGGTATTCTGACCGTGCAAA
GGTAGCATAATCATTTGTTCTCTAAATAAGGACTTGTATGAACGGCCACACGAGGGTTTAAC
TGTCTCTTACTTCCAATCAGTGAAATTGACCTCCCCGTGAAGAGGCGGGGATAAGACAATAA
GACGAGAAGACCCTATGGAGCTTTAATTAACTAATTCAAAAAGAAACTACTAACGACCCAA
CAGGAATAATATATCTCTTTTATGAATTAGCAATTTAGGTTGGGGCGACCTCGGAGGACAAA
ATAGCCTCCGAGTGATTATAAATCTAGACTTACCAGTCAAAATGCTTAATCACTTATTGATCC
AAAAATTCTTTTGATCAACGGAACAAGTTACCCTAGGGATAACAGCGCAATCCTATCCGAGA
GTCCATATCGACAATAGGGTTTACGACCTCGATGTTGGATCAGGACATCCTAATGGTGCAGC
AGCTATTAAAGGTTCGTTTGTTCAACGATTAAAGTCCTACGTGATCTGAGTTCAGACCGGAG
CAATCCAGGTCGGTTTCTATCTATTCAAATAATTTCTCCCAGTACGAAAGGACAAGAGAAAT
AAGGCCTACTTCTCTGAAGCGCCTTAAGACCAATAGATGAATTTATCTAAATCTAGTAAATC
TAACTCCAATATTGCCCAAGAGACAGGGCTTTGTTAGGGTGGCAGAGCCCGGTAATTGTGCA
AAACTTAAACTCTTGTGTCCAGAGGTTCAATTCCTCTCCCTAGCATATGTTTATAATTAACAT
CTTCTCACTAATTGTACCTATTCTTCTTGCTGTAGCCTTTCTGACTCTAGTAGAACGAAAAGT
ACTAGGCTACATACAGCTTCGAAAAGGCCCAAATATTGTAGGTCCATATGGTCTCCTACAAC
CAATCGCAGATGCCGTAAAACTCTTTACTAAAGAACCTTTACGACCACTTACGTCATCCATA
ACTATATTTATTATAGCCCCTATTCTAGCCTTAACACTAGCTCTGACCATGTGAGTCCCCTTA
CCAATACCGCACCCCCTTATTAACATAAACCTAGGAGTACTATTCATACTAGCTATATCAAG
TCTAGCTGTATACTCTATTCTCTGATCTGGATGAGCCTCAAACTCAAAATATGCCCTAATTGG
AGCCCTACGAGCCGTAGCTCAAACGATCTCATATGAAGTCACACTAGCCATTATCCTTTTAT
CCGTTCTACTAATAAATGGCTCATTTACGCTATCCACGCTAATCATTACCCAAGAACGTCTTT
GATTAATCTTCCCTGCATGACCACTAGCTATAATATGATTTATTTCTACCCTAGCAGAAACAA
ATCGAGCCCCCTTTGACCTAACAGAAGGAGAATCAGAACTAGTCTCTGGGTTTAATGTCGAA

TATGCAGCAGGTCCATTTGCCCTGTTCTTCCTAGCAGAATATGCCAACATCATCATAATAAAT
GCTCTTACAACTATTCTATTCTTCGGAGCATTTCACAGCCCCTACATACCAGAATTATATATT
GTTAATTTTACCGTAAAAACTTTACTCCTCACAATCTCTTTTCTATGAATCCGAGCATCATAT
CCTCGATTCCGATATGACCAACTAATACACCTACTATGAAAAAACTTTCTACCTCTCACACTA
GCTCTATGTATATGACATGTAACTCTACCTATTATTACAGCAAGCATTCCACCTCAAACGTAA
GAAATATGTCTGACAAAAGAATTACTTTGATAGAGTAAGCCATAGGGGTTTAAGTCCCCTTA
TTTCTAGAATCATAGGAATCGAACCTAATCTTAAGAACTCAAAAATCTTCGTGCTACCTAAC
TACACCATATCCTAAAGTAAGGTCAGCTAAATAAGCTATCGGGCCCATACCCCGAAAATGTT
GGTTTATCCCCTTCCCATACTAATAAAACCAGCCATCCTTATTACCATTACATCCACCGTTGT
CTTAGGAACTATAATTGTTCTATTTAGCTCCCACTGATTCATAATCTGAGTTGGCTTTGAAAT
AAATATACTAGCCATTATCCCAATTCTAATAAAAAAATATAACCCACGAGCCATAGAAGCTT
CCACAAAATATTTTCTAACACAAGCTACAGCATCCATGCTTCTAATGCTAAGCATCATCATC
AATCTACTATGCTCAGGACACTGGACAGTTTCAACAATTCCCAATCCAGTAGCATCTACCAT
AATTACTATTGCTCTAACAATAAAACTTGGCCTATCACCATTTCACTTTTGAGTCCCAGAAGT
TACACAAGGCATTTCCTTATCTTCAGGAATAATCCTATTAACATGACAAAAAATTGCACCTTT
ATCCATTCTATACCAAATTTCACCATCCGTTAACTCAAATCTCTTACTAATAATAGCTATCAC
ATCCATACTGGTAGGAGGCTGAGGAGGATTAAATCAAACCCAACTCCGAAAAATCTTGGCA
TACTCCTCAATTACCCATATAGGCTGAATGGCTGCTATTATAGTATATAATCCTACCCTAGCA
ATTCTTAACCTAACAATTTACATCATAATAACTCTAGGAACATTTATACTATTCATACATAGC
TCATCTACAACAACACTATCACTATCTTATACATGAAATAAATTCCCATTAATAGCCCCACTA
ATCTTAATACTTATACTATCACTAGGAGGCCTCCCTCCTCTCTCAGGCTTTATCCCCAAATGA
ATAATTATTCAAGAATTAACAAAAAACGACATAATTATTATACCTACACTTATAGCCATTAC
AGCCCTACTAAACCTATATTTTTACACACGCCTAACATATACTACAGCACTAACTATATTTCC
CGCAACAAATAATATAAAAATAAAATGACAATTCGAAACCACAAAAAAGACAACTCTCCTA
GCCCCTCTAATCGTGACATCAACTATACTACTACCACTAACCCCAATACTAGCAGCTCTTGAT
TAGAGATTTAGGCTAAACAGACCAAGGGCCTTCAAAGCCTCAAGTAAGTGAAAATCCACTT
AATCTCTGCAATATTTAAGGACTGCAAGAATCTATCTCACATCAACTGAACGCAAAACAATC
GCTTTAATTAAGCTAAGCCCTTCTAGATCGGTGGGCTTTTACCCCACAAAACTTTAGTTAACA
GCTAAAAACCCTAGACAACTGGCTTCAATCTACTTCTCCCGCCGCGTAGGAAAAAAAGGCG
GGAGAAGCCCCGGCAGAATTGAAGCTGCTTCTTTGAATTTGCAATTCAATATGATTACTCAC
CACAGAGCCTGGTAAAAAGAGGACTTAACCCCCATTCTTAGATTTACAGTCTAGTGCTTTTA
TCAGCCATTTTACCTATGTTCATTAACCGATGACTGTTTTCCACCAACCACAAAGATATTGGC
ACTCTTTATCTTCTGTTTGGCGCATGAGCTGGTATAACGGGCACTGCTCTTAGCCTTCTAATC
CGTGCTGAATTAGGTCAGCCTGGAGCTCTGTTAGGAGATGACCAAATTTACAATGTAGTTGT
AACTGCCCACGCATTCGTAATAATCTTCTTTATAGTTATGCCTATTATGATCGGAGGCTTTGG
AAATTGACTAGTACCTTTGATAATTGGTGCGCCTGATATAGCATTTCCCCGAATAAACAATA
TGAGTTTCTGATTACTACCTCCATCCTTTCTTCTCCTTCTGGCTTCTTCCATAGTAGAAGCAGG
CGCAGGGACTGGATGAACTGTATACCCCCCTCTAGCAGGTAACTTAGCCCATGCAGGAGCAT
CAGTAGACCTAACAATCTTTTCTCTACATCTGGCAGGGGTTTCTTCTATTTTAGGGGCTATTA
ACTTTATTACCACTATCATCAACATGAAACCCCCTGCAATATCTCAATATCAAACTCCCCTAT
TCGTGTGATCAGTACTTATCACAGCAGTACTTCTCCTTCTATCTTTACCAGTCTTAGCAGCTG
GAATTACTATACTACTTACAGATCGAAATCTCAATACTACTTTCTTCGACCCGGCCGGAGGA
GGGGATCCCATTCTATATCAACATCTATTTTGATTCTTTGGCCATCCTGAAGTCTATATTCTA
ATCCTCCCTGGGTTTGGAATAATTTCACACATTGTCACATACTATTCAGGAAAGAAAGAGCC
CTTTGGTTATATAGGAATAGTGTGGGCAATAATGTCAATTGGGTTCTTAGGATTTATTGTATG
GGCCCATCATATATTTACTGTAGGCATAGATGTTGATACACGAGCCTATTTTACCTCGGCCAC
TATAATTATTGCAATCCCGACAGGAGTTAAAGTATTCAGCTGACTAGCTACCCTGCATGGAG
GAAATATTAAGTGATCCCCTGCCATAATGTGAGCCCTAGGTTTCATTTTTCTATTTACAGTAG
GGGGTCTTACAGGCATTGTTCTAGCTAACTCATCTCTGGACATTGTCCTCCATGACACATACT
ACGTAGTAGCACACTTCCACTATGTACTATCAATAGGGGCCGTGTTCGCTATTATAGGAGGC
TTCGTCCATTGATTCCCATTATTTTCGGGCTATACACTTAATAATACATGAGCAAAAATTCAC

TTCGTAATTATATTTGTTGGAGTCAATATAACATTCTTCCCCCAGCACTTCCTAGGTCTATCA
GGTATGCCTCGACGATATTCTGATTATCCAGACGCCTATACAGCATGAAACACAGTGTCTTC
AATGGGTTCATTCATTTCACTAACAGCAGTTATGTTAATAATTTTCATAATTTGAGAAGCCTT
CGCATCTAAACGAGAAGTGGCAGTGATTGAGCTTACTACAACTAATATTGAATGGCTCCACG
GATGTCCTCCCCCATACCATACATTCGAAGAGCCCGCCTACGTCATACTGAAATAAGAAAGG
AAGGAATCGAACCCTCTAGAATTGGTTTCAAGCCAACATCATAACCACTATGTCTCTCTCAA
TAAAGAGGTATTAGTAAAAATTACATAACTTTGTCAAAGTTGAATTATAGGTGAAAATCCTT
TATATCTCTATGGCGCATCCCTTTCAAACAGGCCTTCAAGATGCAACTTCCCCCATCATGGAA
GAACTCCTACACTTCCATGATCATACACTAATAATTGTATTCTTAATTAGCTCCTTAGTTCTC
TATATCATTTCAATTATATTAACTACTAAATTAACACATACAAATACAATAGACGCACAAGA
AGTGGAGACAGTATGAACTATTCTACCAGCTATCATCTTAATTATAATTGCACTTCCATCACT
ACGAATTCTTTATATGATAGATGAAATTAATAGCCCATCATTAACTGTAAAGACTATGGGGC
ATCAATGATACTGAAGCTATGAGTATACAGATTATGAAGACTTGAGCTTCGATTCTTATATA
ACCCCAACACAAGAATTAAAGCCCGGAGAGCTGCGATTACTAGAGGTAGATAATCGAGTAG
TACTACCCATAGAAATAACAATTCGCATATTAATTTCCTCAGAAGATGTGCTACATTCGTGA
GCTGTCCCATCTCTAGGACTAAAAACTGATGCAATCCCGGGGCGATTAAATCAAACAACTCT
TATGGCCATGCGACCAGGACTATATTATGGCCAATGCTCAGAGATTTGCGGTTCTAATCATA
GCTTTATACCCATTGTACTTGAAATAGTTCCGCTGTCTTACTTTGAAAAATGATCCGCTTCAA
TACTATAAAATCATTAAGAAGCTTTATAGCGTTAACCTTTTAAGTTAAAGACTGAGAGTGCA
AGTCTCTCCTTAATGGAAATGCCACAATTAGACACATCCACATGATTTATTACAATCCTATCC
ATGATCTTAACACTATTTATTATATTTCAGTTAAAAGTCTCAAAACACAACTATCCTGAGTTT
CCTGAATTAAAAACAACCCCACTATCAAAAAAACCTATGCCTTGAGAAGAAAAATGAACGA
AAATCTGTTCGCCTCTTTTACTACCCCAATGATAATAGGAGTACCAATTGTGGTTCTGATTAT
TATTTTCCCAAGTATCTTATTTCCCTCACCTAATCGATTAATCAATAACCGCCTAGCTACTGT
CCAACAATGATTAATTCAATTAGTATCTAAGCATATACTGTCTATTCACAATTATAAAGGAC
AAACCTGAGCGCTAATACTAATGTCATTAATTTTATTTATTGGCTCAACTAACCTTTTAGGCC
TATTACCACATTCATTCACACCTACTACACAACTATCAATAAATTTAGGAATGGCCATTCCTC
TGTGAGCAGGTACAGTAGTTATAGGATTTCGACACAAAACTAAGGCATCTCTGGCCCACTTT
CTACCCCAAGGAACACCTCTTCCTCTGATCCCAGTGTTAGTAATTATCGAGACAATTAGTCTG
TTTATTCAACCTGTGGCCCTAGCTGTACGACTGACCGCTAATATTACTGCAGGCCATCTACTT
ATTCATTTAATCGGAGGGGCTACCCTAGTTTTAATAAACATTAACCCTGTAACAGCCCTAATT
ACCTTTATTATTCTTATTCTACTTACTATTCTCGAATTAGCTGTAGCTCTTATCCAAGCCTATG
TTTTCACCCTACTAGTTAGTCTATACTTACATGATAATACCTAATGATCCACCAAACACATGC
ATATCATATAGTTAACCCAAGCCCATGGCCACTTACAGGAGCTCTCTCGGCTCTTCTTATAAC
ATCAGGACTAATTATATGATTTCACTTTAACTCGGTATACTTACTAATACTAGGCCTCACCAC
CAACATACTTACCATGTACCAATGATGACGAGATATCGTCCGGGAAAGTACCTTCCAAGGCC
ATCACACCCCAATCGTCCAAAAAGGACTACGATATGGAATAGTCTTATTTATTGTATCAGAA
GTATTTTTCTTCGCGGGATTCTTTTGGGCCTTCTACCACTCAAGCCTAGCACCTACTCCTGAA
TTAGGAGCATGTTGACCACCTACAGGTATCATCCCCTTAAATCCGCTAGAAGTTCCACTACTT
AACACCTCTGTACTTCTAGCATCTGGGGTATCTATCACTTGAGCTCATCATAGTCTAATAGAA
GGAGACCGTAAACATATGCTCCAAGCCCTATTTATTACTATTTCACTAGGAGTTTATTTTACA
CTTCTACAAGCCTCAGAATACTATGAGACATCCTTTACTATTTCAGATGGAGTGTATGGTTCA
ACCTTCTTTATAGCAACTGGATTTCATGGGCTTCATGTAATTATTGGTTCAACTTTTCTTACTG
TGTGTTTCTACGACAACTGCACTTTCACTTTACATCCAACCATCACTTTGGCTTTGAAGCGG
CAGCATGATACTGACACTTCGTAGACGTTGTATGGCTTTTCCTTTATGTTTCTATTTACTGAT
GAGGATCCTACTTCTTTAGTATTAACTAGTACGATTGACTTCCAATCAATAGGCTCTGGTACA
AGCCAGAAAGAAGTAATAAACCTAATTCTAGCACTTCTAACCAACGTATTATTAGCCTCTCT
GCTCGTTCTTATTGCATTCTGACTGCCACAACTAAATATCTATGCAGAAAAAGTAAGTCCTTA
TGAATGTGGATTTGATCCTATAGGATCAGCACGCCTTCCTTTCTCTATAAAATTTTTCTAGT
AGCTATTACATTCCTTTTATTCGACTTAGAAATTGCTCTACTCCTCCCGCTTCCTTGAGCATTA
CAGACTAACAACTTAATAACTATGCTTATTATAGCTCTATTACTCATTTCCCTATTAGCCGCA

AGCCTAGCTTATGAGTGAACCGAAAAGGGGCTAGAATGGGCCGAATATGATAATTAGTTTA
AACTAAAATAAGTGATTTCGACTCACTAGATTATGACTAACATCATAATTATCAAATGTCTG
TGGTCTATATTAATCTCTTTCTAGCCTTTACTATATCCTTAATAGGGCTACTCATCTACCGATC
CCATTTAATATCCTCCCTATTATGCTTAGAAGGCATGATGCTGTCACTATTTGTTATACTAAC
AGTAACAATCCTAAGTAACCACTTCACACTAGCCAGTATGACTCCAATCATTCTATTAGTATT
CGCTGCCTGTGAAGCAGCGTTGGGGCTTTCTCTGCTAGTAATAGTCTCTAATACCTATGGAA
CTGACTACGTACAAAATCTTAACCTCCTACAATGCTAAAAATTATCCTCCCTACCATAATACT
AGTACCCCTTACATGAATATCAAAGCCTAACATAATCTGAATTAATACAACAACCTATAGTC
TACTCATCAGCCTTATTAGTCTGACATATCTCAATCAACTCAGTGATAATAGTCTAAACTTCT
CATTAACATTCTTTACCGACTCCCTATCAGCCCCCCTGTTAGTTCTCACAACATGACTACTGC
CCTTAATACTTATAGCTAGTCAATTTCACCTGTCAAAAGAGACCCTGACCCGGAAAAAATTA
TATATCACAATACTAATTCTCCTGCAACTATTCTTAATTATAACATTTTCCGCTACTGAGTTA
ATCATATTCTATATTTTATTTGAAGCTACCCTGGTACCGACTTTAGTTATCATTACTCGATGA
GGAAACCAAACAGAACGACTAAACGCGGGCCTCTATTTTTTATTCTACACCTTAGTAGGATC
CTTGCCCTTACTAGTTGCACTGTTATATGCCCAAAACAGCTTAGGATCACTAAATTTTCTCAT
AGTACAGTACTGAACTCAGCCCTTGCCAAATTCCTGATCTAACATTTTCATGTGGCTGGCATG
TATAATAGCATTCATAGTAAAAATACCGCTATATGGCTTACACCTATGACTACCCAAAGCAC
ATGTAGAAGCTCCTATTGCAGGATCCATAGTACTTGCTGCAGTACTTCTAAAACTAGGGGGA
TACGGTATAATGCGGATTACAACAATACTCAACCCTCTAACAAATTTTATAGCCTACCCCTTC
ATAATGCTCTCACTATGAGGAATAATCATAACAAGTTCTATTTGTTTACGCCAGACAGACCT
AAAGTCCCTAATTGCATACTCCTCTGTTAGTCACATAGCCCTAGTCATTGTAGCAATTCTAAT
CCAATCCCCATGAAGCTACATAGGAGCTACTGCTCTAATAATCGCCCATGGGTTAACATCTT
CTATGCTATTCTGTCTTGCAAACTCTAACTACGAGCGAACCCATAGCCGAACTATAATTCTCG
CACGGGGCTTGCAAACGCTTCTTCCACTTATAGCAGCTTGATGACTATTAGCAGGCCTAACT
AACTTAGCTCTTCCTCCTACAATTAATCTGATTGGGGAACTGTTTGTAGTGATAGCCACATTT
TCATGATCCAATATTACCATTCTCTTAATAGGAGTTAACATTACCATTACTGCTCTCTACTCT
CTATATATACTAATTTCTACACAGCGAGGCAAGTATACAGACCACATCAAAAATATTAAGCC
ATCTTATACACGAGAAAATGCCTTAATAGCCTTACATTTACTACCCCTTTTACTTCTATCTCTT
AACCCTAAAGTAGTTCTAGGCTTTATTTACTGTAAATATAGTTTAACAAAAACATTAGATTGT
GAATCTAGTAATAAAAGCTTAAATCTTTTTATTTACCGAAAAAGCATGCAAGAACTGCTAAT
TCATGCACCCATGCATAAAATCATGGCTTTTTCAACTTTTAGGGGATAGAAGTAATCCATTG
GTCTTAGGAGCCAAAAAATTGGTGCAACTCCAAATAAAAGTAATTAACCTACTTACCTCCTT
CACCATTACAACCCTATTTATATTAATCATGCCCATTATCCTAACCTGTACCCCAGCATATGA
AAATAAACTTTACCCGCAATATGTAAAAACCACAGTCTCTTATGCCTTCACAATTAGCATAA
TTCCCATAGTAATATTCATCCACTCCGGACAAGAAGCAATCATCTCAAACTGACACTGAATA
ACCATCCAAACCCTAAAACTCAGTCTAAGCTTCAAATTAGATTACTTTTCAATAATCTTCGTA
CCAGTGGCCCTATTCGTCACATGGTCAATTATAGAATTCTCTATATGATATATACACGCAGAT
CCTAACATTAACCGATTCTTTAAATATCTACTTATATTCCTCGTCACGATAATAATTCTAGTA
ACCGCAAATAATCTATTTCAGCTATTCATCGGCTGAGAGGGAGTAGGAATCATATCATTTCT
CCTCATTGGATGATGATATGGACGAACAGATGCTAATACGGCTGCCCTACAAGCAGTACTAT
ACAACCGCATTGGAGATGTTGGTTTTATCATGGCCATAGCTTGATTCTTACTCAATACAAATG
CATGGGACTTCCAACAAATCTTTATAATCGAACATAAAAACCTCAATATTCCACTGGCAGGA
CTACTTCTAGCAGCTACCGGAAAGTCTGCCCAATTTGGACTTCATCCATGACTCCCATCAGCC
ATAGAAGGCCCCACTCCTGTCTCAGCACTACTTCACTCTAGCACAATAGTAGTAGCAGGCGT
ATTTCTTCTAGTCCGATTTCACCCCTTAATAGAACATAACAAGACAATTCAAACGGTTACCCT
ATGCTTAGGGGCAATAACAACCTTGTTTACAGCAATCTGCGCCCTTACCCAAAATGATATCA
AAAAAATTATTGCCTTCTCCACCTCGAGCCAACTAGGACTAATAATTGTAACAATTGGTATT
AATCAGCCTCATCTGGCCTTTCTCCATATCTGCACGCACGCATTCTTCAAGGCCATGCTATTT
CTATGCTCCGGATCTATTATTCATAGTCTAAACAACGAACAAGATATTCGAAAGATAGGAGG
GCTATTTAAAAGTCTACCATTTACCACTACTGCTCTAATCATCGGAACTCTAGCACTTACAGG
AATGCCCTTCTTAACAGGATTTTACTCCAAAGACTTAATTATTGAAACCGCCAATACGTCAT

ACACCAACGCCTGAGCCTTACTAACAACCCTTATCGCCACATCCATAACTGCCGCCTACAGT
ACTCGAATTGTATTTTTTACACTTCTAGGACAACCTCGCTATAGTCCCATTATCACCATTAAC
GAGAATAACCAGTTCCTGACTAACTCTGTCAAACGATTGCTACTAGGAAGTATCTTTGCGGG
ATTCCTAATCTCTCATAACATTACACCCACCACTGTCCCACAAATAACCATGCCCTGTCACCT
AAAAATGATAGCCCTTACAGTAACAATCCTAGGCTTTATTCTAGCACTAGAACTTAACCTAG
CCATACAAAATCTCAAATTTGACTACCCGTTAAGCACGTTCAAATTTTCAAATCAACTAGGA
TATTTCCCTACTACTCTGCATCGTCTCGCACCAATAATAGGTCTATCAATAAGCCAAAAAAC
AGCATCCATACTGCTAGATGCAATCTGACTAGAGAATGTTCTACCAAAGTCCATCTCCTACT
TTCAAATAAAATCCTCAATCACAGTCTCCAACCAAAAGGGACTAATTAAACTATACTTTCTC
TCCTTTATAATTACACTAATCTTAAGTCTCTTTATACTTAATTTCCACGAGTGACTTCCATAAT
TACCAATACACCAATAAGGAGAGATCATCCAGTAACAATAACTAATCAAGTACCATAACTA
TATAAAGCTGCAATTCCCATAGCCTCTTCACTAAAAAATCCTGAATCCCCTGTATCATAAATC
ACCCAATCTCCTATTCCATTAAACTTAAATACTACCTCGGCTTCATCATCCTTTAAAATATAA
CAAACAGCCAGTAACTCAGACAACAGACCTACAATAAATGCACCTAGTACAGCCTTATTAG
AAAGTCAAACCTCAGGATATTGCTCAGTGGCTATGGCCGTAGTGTACCCGAACACTACAAGT
ATACCCCCTAAATAAATTAAAAAGACCATTAAACCCAAAAAAGATCCTCCAAAGCTTAACA
CAATACCACACCCTACACCCCCACTAACAATCAAGACTAAACCTCCGTAGATAGGAGAGGG
CTTAGAAGAAAACCCCGCAAAACTAACTACAAAAACGATACTTAAAATAGATACAATGTAT
GTTATCATCATTCTTACATGGAATCTAACCATGACCAATGACATGAAAAATCATCGTTGTATT
TCAACTATAAGAACCCCAATGATCAACATCCGAAAAACTCATCCATTAGTTAAAATTGTCAA
CAACTCATTCATTGACCTTCCAACACCATCAAACATTTCAACATGATGGAACTTTGGGTCCCT
GTTAGGAGTGTGTCTGATCTTGCAAATCTTAACAGGCTTATTTCTAGCCATACACTATACATC
AGATACAGCTACAGCCTTTTCATCAGTCGCACACATTTGTCGAGACGTCAACTATGGTTGAT
TTATCCGATATATACATGCCAATGGGGCCTCTATATTTTTTATCTGCCTATTTATACACGTAG
GGCGAGGCTTATACTATGGATCATACCTATTTCCAGAGACATGGAATATCGGAATTATTCTC
CTACTTACAGTTATAGCCACAGCATTCATAGGATATGTACTACCTTGAGGACAAATATCCTT
CTGAGGAGCAACCGTCATTACTAACCTACTATCAGCAATTCCTTACATTGGCACTAATCTAG
TGGAGTGAATCTGAGGGGGTTTCTCCGTAGATAAAGCAACACTAACCCGATTTTTTGCTTTTC
ACTTTATCCTTCCATTTATCATCTCAGCACTAGCAATAGTCCATCTATTATTCCTTCACGAAA
CAGGATCTAATAACCCCTCCGGAATTCCATCTGACCCAGACAAAATCCCATTTTACCCCTAT
CATACAATTAAAGACATCCTAGGCGTCCTATTTCTTGTCCTCGCCTTAATAACTCTGGCTTTA
TTCTCACCAGACCTGTTAGGAGACCCTGATAACTATACCCCTGCAAACCCACTAAGTACCCC
GCCACATATTAAGCCTGAATGGTACTTTCTATTTGCCTACGCTATCCTGCGATCTATCCCTAA
TAAACTAGGAGGGGTGCTAGCTCTAATCTTCTCTATTCTAATTCTAACTATTATTCCACTATT
ACATACATCCAAACAACGAAGCATGATATTCCGACCTCTAAGTCAATGCTTATTCTGACTCC
TAGTAGCAGACCTACTCACACTAACATGAATTGGAGGACAGCCAGTAGAACACCCCTTCATT
ATTATTGGGCAATTGGCCTCTATTCTCTACTTTACAATTCTTCTAGTACTTATACCTATCACTA
GCATTATTGAGAATAGCCTCTCAAAATGAAGAGTCTTTGTAGTATATTAATTACTTTGGTCTT
GTAAACCAAAAACGGAGATTCCTCTACTTCTCCCTAAGACTCAAGGAAGGAGCAACAACCC
CACTACCAGCACCCAAAGCTGGTGTTCTACTTAAACTATCCCCTG

>SRR6109212 Circular ReOrganized
GTACATACCATTATTTCACTTTATGTCCTATTTATTTCATATATATCATTCCATGTACTGTACC
ATCACAGTATGTTCTCGAATACTTTCTCCTTTGTTTTTTCCCCCCCTATGTATATCGTGCATT
AATGGCGTGCCCCATGCATATAAGCATGTACATACTGTGCTTGGCTTTACATGAGGACTTAC
GTTCCGAAAAGTTTATTTCAGGCGTATAGTCTGTAAGCATGTATTTCACTTAGTCCGGGAGCT
TAATCACCAGGCCTCGAGAAACCAGCAATCCTTGCGAGTACGTGTACCTCTTCTCGCTCCGG
GCCCATGGAATGTGGGGGTTTCTATGTTGAAACTATACCTGGCATCTGGTTCTTACCTCAGG
GCCATGATAGCTCTAGATTCCAATCCTACTAACCCTTCAAATGGGACATCTCGATGGACTAA
TGACTAATCAGCCCATGATCACACATAACTGTGGTGTCATGCATTTGGTATCTTTTAATTTTT

AGGGGGGGAACTTGCTATGACTCAGCTATGACCGTAAAGGTCTCGTCGCAGTCAAATAATTT
GTAGCTGGGCTTATTTATCTTTCACGGGTCGGGCATAGATACCCATAAGGGGCTATTCAGTC
AATGGTTACGGGGCATATAGTATGTAAATACCACTGAATCGAACGAACACGCACGTGTACG
CACGTGTACGCATATGTACGCATATGTACGTACGTGTACGCACGTGTACGTACGTGTACGTA
CGTGTACGTACGTGTACGCACGTGTACGCACGTGTACGCACGTGTACGCACGTGTACGCACG
TGTACGCACGTGTACGCACGTGTACGCACGTGTACGCGCGCGTTTTTAGATATTAACTTAGC
TTAATCAAACCCCCCCTTACCCCCCGTAACTTCAAGGAGCTTACGCACATTTATGATTGTCCTG
CCAAACCCCAAAAACAAGACTAAATGCACGTGCAAACATGAAGCTATTACACCCAAAATCC
CACATAATAAGCTAAAATTCCCAGCCAAATAATCACTACAATCACAGGCATGAGACTTTAAA
TTAAGATCTATCTATAGATATTTTTTTTTTACTCTGTCTCCCTCCTATTGATTTTTCCGTTATT
ATCACTTTTTTAGCCACGAGCTTCTACTAAATTATCCCATATAAATCCCAAAACCAACCGCGT
TTATGTAGCTTAATAATAAAGCAAGGCACTGAAAATGCCTAGACGAGTTGTATAACCCCATA
AACATAAAGGTTTGGTCCTAGCCTTCCCATTAGCTATTAACAAGATTACACATGTAAGTCTC
CGCGCTCCAGTGAAAATGCCCTTTGGATCTTAAAGTGATCTGAAGGAGCGGGCATCAAGCAC
ACCCTTCCCCGGTAGCTTATAACGCCTTGCTCAGCCACACCCCCACGGGATACAGCAGTGAT
AAAAATTAAGCCATAAACGAAAGTTTGACTAAGCTATGTTGATTAAAGGGTTGGTTAATTTC
GTGCCAGCCACCGCGGTTATACGATTGACCCAAGTTAATAGGCCCACGGCGTAAAGCGTGTG
AAAGAAAAAAATTTTTCCCCATTAAAGTTAAAGTTTAATCAAGCTGTAAAAAGCTACCAATA
ACACTAAAATAGACTACGAAAGTGACTTTAATACTCTCAACCACACGACAGCTAAGATCCA
AACTGGGATTAGATACCCCACTATGCTTAGCCTTAAACATAAATAATTTATCAAACAAAATT
ATTCGCCAGAGAACTACTAGCAACGGCTTAAAACTCAAAGGACTTGGCGGTGCTTTAAACCC
CCCTAGAGGAGCCTGTTCTGTAATCGATAAACCCCGATAGACCTCACCACCCCTTGCTAATC
CAGTCTATATACCGCCATCTTCAGCAAACCCTTAAAAGGAAAAAGAGTAAGCACAATCATCT
TGCATAAAAAAGTTAGGTCAAGGTGTAACTCATGGGATGGGAAGAAATGGGCTACATTTTCT
ATTCAAGAACAACCTACGAAAGTTTTTATGAAACTAAAAACTAAAGGTGGATTTAGTAGTAA
ACCAAGAATAGAGAGCTTGGTTGAATAAGGCAATGAAGCACGCACACACCGCCCGTCACCC
TCCTCAAGTGGCACAAGTCAAATATAACCTATTAAAACCAAATAAAACGCAAGAGGAGACA
AGTCGTAACAAGGTAAGCGTACTGGAAAGTGTGCTTGGATAAACCAAAGTGTAGCTTAAAC
AAAGTATCTGGCTTACACCCAGAAGATTTCACGTATGTGACCGCTTTGAGCCCAGAGCTAGC
CCAGACAATAACCGATTAAACTACCACAGGCCAATTAAATAAAACATTCAGTAGCGCAATT
AAAGTATAGGAGATAGAAATTCTTTAGATCGGAGCTATAGAGAAAGTACCGCAAGGGAACG
ATGAAAGATTACCCAAAGTGATAAACAGCAAAGATTACCCCTTCTACCTTTTGCATAATGAG
TTAGCCAGAAATAACCTAACAAAGAGAACTTAAGCTAGGTCCCCCGAAACCAGACGAGCTA
CCTATGAACAATCCACTGGGGTAAACTCATCTATGTTGCAAAATAGTGAGAAGATTTGTAGG
TAGAGGTGAAAAGCCTAACGAGCCTGGTGATAGCTGGTTGCCCAGAATAGAACTTCAGTTC
AACTTTAAACTTGCCTACAAAACTTAGATAATTTTAATGCAAGTTTAAAATATATTCTAAAA
AGGTACAGCTTTTTAGAATCAAGGATACAACCTTACTTAGAGAGTAAATACTGATTAAACCA
TAGTAGGCCTAGAAGCAGCCATCAATTAAGAAAGCGTTTAAGCTCAACATCCATATCAACTT
AATACCAAAAGTATTTAATCAACTCCTAATATAACAACTGGGCTAATCTATTTTAATATAGA
AGCAATAATGCTAATATGAGTAACGAGAGATACCTCTCCAACGCATAAGCCTATAACAGCA
ACGGATAACCACTGATAGTTAACAACAACGTAGAAATAACCCAACAATAAAACATCTACCA
AACCAATTGTTAATCCAACACAGGTATGCGACTAAGGAAAGATTAAAAGAAGTAAAAGGAA
CTCGGCAAACACAAATCCCGCCTGTTTACCAAAAACATCACCTCCAGCATTCCCAGTATTGG
AGGCACTGCCTGCCCGGTGACATTAGTTAAACGGCCGCGGTATTCTGACCGTGCAAAGGTAG
CATAATCATTTGTTCTCTAAATAAGGACTTGTATGAAAGGCCACACGAGGGTTTAACTGTCT
CTTACTTCCAATCAGTGAAATTGACCTTCCCGTGAAGAGGCGGGAATAAAACAATAAGACG
AGAAGACCCTATGGAGCTTTAATTAACTAGCTCAAAAGAATCTATCTACCAGACCGACAGG
AACAACATACTCCTTCCATGAGCTAACAATTTAGGTTGGGGCGACCTCGGAGCATAAAACAA
CCTCCGAGTGATATTAATCTAGACGTACCAGTCAAAATGCTCACTTACTTATTGATCCAAAA
ACCTCTTTGATCAACGGAACAAGTTACCCTAGGGATAACAGCGCAATCCTATTTAAGAGTCC
ATATCGACAATAGGGTTTACGACCTCGATGTTGGATCAGGACATCCTAATGGTGCAGCAGCT

ATTAAGGGTTCGTTTGTTCAACGATTAAAGTCCTACGTGATCTGAGTTCAGACCGGAGCAAT
CCAGGTCGGTTTCTATCTATTTAAACAACCTCTCCCGGTACGAAAGGACAAGAGAAGTAAGG
CCTCCCTCACTGAGGCGCCTTAAGACCAATAGATGATTTAATCTAAATCCAGTAAGTCTATC
CCCAATACTGCCCAAGAGACAGGGCTTTGTTAGGGTGGCAGAGCCCGGTGATTGCATAAAA
CTTAAACCTTTATATTCAGGGGTTCAAATCCTCTCCCTAACATTATGTTTATAATCAACACTA
TCTCACTAGTTGTACCCATTCTCCTTGCCGTAGCCTTCTTAACACTAGTAGAGCGAAAAATAC
TAGGCTATATACAACTTCGTAAAGGACCAAACATTGTAGGGCCCTACGGTCTCCTACAACCT
GTCGCAGATGCTGTAAAACTTTTCACCAAAGAGCCTCTACGCCCACTCACATCATCTGCGAC
TATATTCATTATGGCCCCTATTCTGGCTCTAACACTGGCCCTAACCATGTGAATTCCCCTACC
AATGCCATATCCCCTTGTCAATATAAACCTCGGAGTACTATTCATACTAGCAATATCAAGCC
TAGCTGTATACTCTATCCTTTGATCAGGATGAGCTTCAAATTCAAAATACGCTCTGATCGGA
GCCCTACGGGCCGTAGCCCAGACAATTTCATATGAAGTCACATTAGCTATTATCCTCCTATC
AGTCCTACTAATGAATGGCTCATTCGCACTTTCCACTTTAATTACTACTCAAGAGCACCTCTG
ACTAATCTTCCCTGCATGACCCCTAGCCATGATATGATTTATTTCCACCCTAGCAGAAACTAA
CCGAGCCCCCTTCGACCTAGCAGAAGGAGAATCAGAACTGGTCTCAGGTTTTAACGTTGAGT
ACGCGGCAGGTCCGTTCGCCCTATTTTTCCTAGCAGAATATACTAATATCATTATAATAAATG
CCCTCACGACTATCCTGTTCTTCGGGGCATTTCATAATCCCTACATACCAGAACTGTACACTG
TCAACTTCACCGTAAAAACACTACTCTTAACAGCTTCCTTCCTATGAATCCGTGCATCATATC
CCCGATTCCGATATGACCAACTAATGCATCTACTATGAAAAAACTTCCTACCCCTTACACTA
GCCCTATGCATGTGACATGTAACACTACCCATTATCACAGCAAGCATTCCCCCCCAAATATA
AGAAATATGTCTGACAAAAGAATTACTTTGATAGAGTAAATAATAGAGGTTGGAATCCTCTT
ATTTCTAGAATTATAGGAATTGAACCTAATCCTAAGAACTCAAAAATCTTCGTGCTACCTAA
TTACACCATATCCTAAAGTAAGGTCAGCTAAATAAGCTATCGGGCCCATACCCCGAAAATGT
TGGTTTATCCCCTTCCCGTACTAATTAAACCTCCCATTCTTATCATTATCACATCCACCATTGT
CCTAGGAACCATAATCGTTTTATTTAGCTCCCATTGACTCATAATCTGAATTGGCTTTGAAAT
AAATATACTAGCTATTATCCCAATCCTAATGAAAAAATTTAACCCACGAGCCATAGAAGCCT
CTACAAAATATTTCCTCACACAAGCCACTGCATCCATGCTTCTAATACTAGGCATTATTATCA
ACCTACTACACTCAGGGCACTGAACAGTCTCAATAATCCCTAACCCAATCGCATCAACCGTA
ATTACCATTGCCCTAGCAATAAAACTCGGCCTATCCCCCTTCCACTTCTGAGTCCCAGAAGTC
ACACAAGGCGTTCCTTTATCCTCAGGAATAATTCTACTAACATGACAAAAAATCGCACCTCT
ATCCGTCTTATATCAAATCTCACCATCCATCAACCCAAACCTACTGGTAGCAATAGCCGCCA
CATCCGTATTAGTAGGGGGCTGAGGAGGACTAAACCAAACTCAACTTCGAAAAATCCTAGC
ATACTCTTCAATCGCTCACATGGGCTGAATAGCCGCCATCATAGTATACAACCCCACTCTAA
TAATCCTTAACCTTGCAATCTATATCATAATAACTCTGGGGACATTCATACTATTTATGCACA
GCCTATCCACAACAACACTATCACTGTCCCATACATGAAATAAACTACCACTAACTGCCTCA
CTAATTCTAGCACTTATATTATCACTAGGCGGCCTCCCTCCCCTCTCAGGCTTCATCCCCAAA
TGAATAATTATTCAAGAACTAACAAAAAATGATATAATCGTCATGCCCACATTCATAGCCAT
CACAGCCTTACTAAATCTTTATTTTTACATGCGTCTAACATACGCCACAGCATTAACAATATT
CCCCTCAGCAAATAACATAAAAATAAAATGACAGTTTGAAAGCACAAAAAAAATAATTCTC
TTGCCCCCTCTAATCGTAGCATCAACCATACTCCTCCCACTAACTCCATTACTATCAATCATG
GACTAGAGATTTAGGCTAAAAAGACCAAGGGCCTTCAAAGCCCTAAGTAAGTGAAACCCAC
TTAATCTCTGCAATTATCTAAGGACTGCAAGAACATATCTCACATCAATTGAACGCAAAACA
ACCGCTTTAATTAAGCTAAGCCCTCCTAGATTGGTGGGCTTTTATCCCACAAAATTTTAGTTA
ACAGCTAAAAACCCCAAACAACTGGCTTCAATCTACTTCTCCCGCCGCGTGGGAAAAAAAG
GCGGGAGAAGCCCCGGCAGAATTGAATCTGCTTCTTTGAATTTGCAATTCAATATGATTATT
CACCACAGAGCTTGGCAAAAAGGGGACTCAACCTCTATTCTTAGATTTACAGTCTAGTGCTT
TTATCAGCCATTTTACCTATGTTCATAAACCGATGACTATTCTCTACAAATCATAAAGATATT
GGCACTCTCTATCTTCTATTCGGTGCATGAGCCGGAATAGTAGGTACTGCTCTCAGCCTTTTG
ATTCGTGCCGAACTAGGTCAGCCCGGGACCCTGTTGGGGGATGATCAGATCTACAATGTAGT
CGTAACTGCCCATGCATTCGTGATAATCTTCTTTATGGTTATGCCTATTATAATTGGGGGGTT
TGGGAACTGATTAGTGCCCTTAATAATTGGTGCTCCTGACATAGCGTTTCCTCGAATGAATA

ACATAAGCTTCTGACTGCTGCCACCATCTTTCTTGTTGCTTCTAGCTTCTTCTATAGTAGAAG
CAGGTGCAGGAACTGGGTGGACTGTCTACCCCCCTCTAGCGGGCAATCTGGCCCATGCAGGA
GCATCAGTAGACCTAACAATCTTTTCTCTACACTTAGCAGGCATCTCTTCTATTCTGGGAGCC
ATCAATTTCATCACTACTATCATTAACATAAAACCTCCTGCAATATCTCAATACCAAACCCCT
CTGTTTGTGTGATCAGTCCTAATCACGGCAGTGCTTCTTCTTTTATCTCTGCCAGTCTTGGCG
GCTGGGATTACCATACTACTTACAGATCGAAACCTCAACACTACCTTTTTTGACCCAGCCGG
AGGGGGAGATCCTATTCTGTACCAACACTTGTTTTGATTCTTCGGACACCCTGAAGTTTATAT
CCTAATTCTTCCAGGGTTCGGAATGATCTCTCACATTGTCACTTATTACTCAGGAAAAAAAG
AGCCTTTCGGCTACATGGGTATAGTCTGAGCGATAATATCCATTGGATTCTTAGGATTTATCG
TGTGAGCTCATCATATGTTTACCGTAGGTATAGATGTCGACACACGAGCTTACTTCACTTCAG
CTACCATAATTATTGCAATCCCAACAGGAGTCAAAGTATTTAGCTGACTAGCTACCCTGCAC
GGAGGGAATATTAAATGATCTCCTGCTATAATATGAGCCCTAGGCTTTATTTTTCTGTTTACA
GTAGGGGGCCTTACAGGAATTGTCCTAGCTAATTCGTCTCTAGACATTGTTCTCCATGACAC
ATATTATGTGGTAGCCCATTTCCACTATGTGCTGTCAATAGGAGCTGTCTTTGCCATTATAGG
AGGATTTGTCCACTGATTTCCGCTGTTTTCAGGCTATACACTTAATAATACATGAGCAAAGAT
TCACTTCATAATCATATTCGTTGGGGTTAATATAACTTTCTTTCCCCAACATTTCCTGGGCCT
GTCGGGAATGCCTCGGCGATATTCCGACTATCCAGATGCCTACACAATATGAAATACAGTAT
CTTCTATAGGCTCATTCATTTCACTAACAGCAGTTATACTAATAATTTTCATGATTTGGGAGG
CCTTTGCATCAAAGCGAGAGGTAGCAGTGGTAGAACTCACTTCAACCAATATTGAATGACTA
CATGGATGTCCTCCTCCGTACCATACATTTGAAGAACCCGCTTACGTTATACTAAAATAAGA
AAGGAAGGAATCGAACCCTCTGGAATTGGTTTCAAGCCAATATCATAACCACTATGCCTCTC
TCAATAAAGAGATATTAGTAAAAATTACATAACTTTGTCAGGGTTAAATTATAGGTGAAAAT
CCTTTATATCTTTATGGCATATCCCCTTCAAATAGGTCTTCAAGACGCAACTTCTCCTATCAT
GGAGGAACTCCTACATTTTCATGACCATACACTAATAATTGTATTCCTAATTAGCTCCTTAGT
TCTCTATATTATTTCAATTATATTAACTGCCAAATTAACACACACAAATACAATAGATGCAC
AAGAAGTAGAGACGGTATGAACCATCTTGCCGGCTATTATTCTAATTCTAATTGCACTCCCA
TCGCTACGGATTCTTTATATAATAGATGAAATCAATAACCCCTCACTGACCGTAAAAACCAT
GGGCCATCAATGATATTGAAGTTACGAGTATACAGATTACGAAGATCTGAGCTTTGACTCTT
ATATAACCCCAACACAAGAACTAAAACCTGGAGAACTGCGACTATTAGAAGTGGACAATCG
AGTAGTACTGCCCATAGAAATAACGATCCGTATGCTGATCTCATCAGAAGATGTCTTGCACT
CATGGGCTGTGCCATCCCTAGGATTAAAAACTGATGCAATCCCAGGACGGCTAAACCAAAC
AACTCTCATAGCTATACGACCAGGATTATATTACGGCCAATGCTCAGAAATTTGTGGCTCCA
ATCACAGTTTTATACCTATTGTTCTCGAGCTAGTTCCACTATCTTACTTTGAAAAATGGTCTG
CCTCGATACTATAGAATCATTAAGAAGCTAATATAGCGTTAACCTTTTAAGTTAAAGACTGA
GAATGCAGACTTCTCCTTAATGAAGATGCCACAACTAGACACATCAACATGATCTATCACAA
TCCTATCTATAGTTCTAGCACTATTCATTATATTCCAGTTAAAGATCTCAAAATATAAATACC
CCGAAACCCCCGAACCGAAATCCCCTTCACCATCAAAGAAACGTATACCTTGAGAAGAAAA
ATGAACGAAAATCTATTCACCTCTTTTATTACCCCGATGATAATGGGGGTTCCCATTGTAGTC
TTAATCGTTATGTTTCCGAGCATTTTATTCCCTTCTCCCAGTCGACTGATCGACAATCGCTTA
GTGTCTATTCAACAATGACTAGTCTGGCTAACATCAAAACAAATGCTATCCATTCATAATCG
TAAAGGGCAAACCTGAGCACTAATGTTGATATCCCTAATCCTATTTATTGGCTCAACTAATCT
TCTGGGTCTATTGCCACACTCATTCACACCTACCACGCAACTGTCAATGAATCTGGGAATGG
CCATTCCCTTGTGAGCAGGTACAGTTGCCACTGGATTTCGATATAAGACCAAAGCATCCTTA
GCTCACTTTCTACCTCAAGGAACACCTTTCCCTCTAATCCCTATACTTGTTATTATCGAAACA
ATCAGTCTATTCATCCAACCTATGGCCTTAGCCGTACGATTAACTGCTAATATTACTGCAGGT
CACTTGCTCATCCATTTAATTGGAGGGGCTACTCTAGCTCTAACAAGTATTAGCACTATTACA
GCCCTTATCACCTTTATTATTCTAGTGCTACTTACTATTCTCGAGTTTGCTGTAGCCCTCATTC
AGGCCTACGTCTTTACCCTGCTAGTAAGCCTATACTTACACGATAACACTTAATGACTCACC
AAACACATGCATATCATATAGTTAACCCAAGTCCATGACCACTAACAGGAGCCCTTTCAGCC
CTTCTTATAACATCAGGACTAATCATATGATTCCACTTTAACTCTATATTCTTGCTATCGCTA
GGCCTTACAACCAACATACTTACTATATACCAATGATGACGGGATATTATTCGAGAGAGTAC

CTTTCAGGGTCATCACACCCCCGTTGTCCAAAAAGGACTGCGGTACGGAATAGTCCTATTTA
TCGTATCAGAAGTATTCTTCTTCGCAGGGTTCTTCTGAGCTTTTTATCACTCAAGCCTAGCAC
CCACTCCCGAACTAGGGGCATGCTGACCACCCACAGGTATTACTCCCCTGAACCCGCTAGAA
GTACCACTTCTCAATACTTCAGTACTTCTTGCATCCGGAGTGTCCATTACCTGAGCTCACCAC
AGCTTAATAGAGGGAAGCCGCAAGCACATGCTTCAGGCCCTATTTATTACTATCTCTCTAGG
CGTGTACTTCACACTTCTACAAGCCTCAGAGTATTATGAGGCGTCCTTCACTATCTCAGATGG
AGTCTATGGCTCAACTTTCTTCATAGCGACTGGATTTCACGGACTTCATGTAATTATTGGCTC
AACTTTTCTCACAGTCTGTTTCCTACGGCAACTACATTTTCATTTTACATCAAGCCACCATTTC
GGCTTTGAAGCAGCAGCATGATATTGACACTTCGTAGATGTCGTATGACTATTCCTATATGTT
TCTATCTATTGATGAGGGTCTTGCTTCTTTAGTATCGATCAGTACAATTGACTTCCAATCAGT
TAGCTCTGGTGTAATCCAGAAAGAAACAATCAACTTAATCTTAGCACTATTTACTAACATAC
TACTAGCTTCCTTACTCGTCCTTATTGCCTTCTGACTACCACAATTAAATATCTATGCAGAAA
AAGCAAGCCCCTATGAATGCGGGTTTGACCCCGTAGGATCGGCACGCCTCCCTTTTTCAATA
AAATTTTTCCTAGTAGCTATCACATTTCTACTATTTGACCTAGAAATCGCGCTACTCCTCCCC
CTTCCCTGAGCATTACAAACTGACAATCTAATAACGATACTCATCATAGCACTACTGCTTATC
TCCCTATTAGCCGCAAGTCTGGCCTATGAATGAGCCGAAAAAGGTCTAGAATGGGCCGAAT
ATGATAATTAGTTTAAACCAAAACAAATGATTTCGACTCATTAGATTATGAATAATAACATA
ATTATCAAATGCCTGTGGTCTATGTCAATATTTTCCTAGCCTTCATCGTATCCCTAATAGGGC
TACTTATTTACCGATCACATCTAATGTCCTCCCTACTTTGCCTGGAGGGTATAATGCTATCTT
TATTTGTCATACTAACAGTAACAGTTTTAAATAATCATTTTACACTAGCTAACATAGCTCCCA
TTATCTTACTAGTATTTGCTGCCTGCGAAGCAGCACTAGGACTATCCCTGCTAGTAATAGTCT
CCAACACTTATGGAACCGATTACGTACAAAATCTTAATCTCTTACAATGCTAAAAATTATCTT
TCCCACTATAATACTAGTGCCCCTTACATGAGCATCAAAACCCAGTATAATCTGGATTAATA
CAACAGCTTACAGCCTACTCATTTGTCTTATCAGCTTGACATATTTCAACCAGCTCAGTGATA
ATAGCCTAAACTTCTCATTATTATTCTTTACCGACTCCCTATCAGCCCCCTTACTAGCTCTTAC
AACATGACTACTGCCTCTGATACTAATAGCAAGTCAATCCCACCTATCAAAGGAGACCCTGA
CCCGAAAGAAACTATATATCACTATACTAGTCCTCCTACAGCTATTCCTGATTATAACCTTTT
CTGCTATAGAGCTAATCCTGTTCTATATCTTGTTCGAAGCCACCCTAATACCCACTCTAATTA
TTATTACTCGATGAGGGAATCAAACAGAACGATTAAACGCGGGCCTTTACTTTCTATTTTAT
ACTCTAGTAGGATCTTTACCACTGCTAATTGCACTACTACATACTCAAAACAATCTAGGTAC
CCTAAATCTTCTCATAATACAATACTGAATCCAGCCCCTGCCAAACTCCTGATCCAGTGTCTT
CCTGTGACTAGCATGTATAATAGCATTCATAGTAAAAATACCTCTATATGGCCTTCACTTGTG
ATTGCCAAAAGCACATGTAGAAGCCCCTATCGCAGGATCTATAGTGCTTGCCGCCGTACTCC
TAAAACTAGGAGGCTACGGCATGATACGAATTACAACACTACTTGACCCTCTAACAAGCTTC
ATGGCCTACCCCTTCATAATACTCTCACTATGGGGCATAATCATAACAAGTTCTATTTGCCTG
CGCCAAACGGATTTAAAGTCCCTAATTGCTTACTCCTCTGTTAGTCATATAGCCCTAGTCATT
GTAGCAGTATTGATCCAAACTCCATGAAGCTATATAGGAGCAACAGCCTTAATAGTTGCCCA
TGGACTAACATCCTCCATGCTATTCTGTCTTGCAAACTCCAACTATGAACGAGTCCATAGCC
GAACTATAATCCTTGCGCGAGGTCTACAAACACTCCTTCCACTTATAGCAGCTTGATGACTA
CTAGCAAGTCTTACTAATCTGGCACTCCCTCCTACCATCAATCTCATCGGAGAACTATTTGTA
GTAGTAGCCACATTTTCATGATCTAACATCACCATCACTCTAATAGGAGCCAACATCATCAT
TACTGCCCTCTATTCTCTCTACATACTAATCACCACACAACGAGGCAAGTGCACAGACCATA
TCAAAAGCATCAAGCCATCCTTCACACGAGAAAACGCCCTAATAGCTCTCCACCTCCTACCC
CTTCTACTTCTATCTCTCAACCCCAAAGTAATTCTAGGACCCATTTACTGTAAATATAGTTTA
ACAAAAACATTAGATTGTGAGTCTAATAATAAAAGCCCAGGCCTTTTTATTTACCGAAAAAG
TACGCAAGAACTGCTAATTCATGCATCCATGCATAAAATCATGGCTTTTTCAACTTTTAAAG
GATAGAAGTAATCCATTGGTCTTAGGAATCAAAAAATTGGTGCAACTCCAAATAAAAGTAA
TTAATCTGTTTGCCTCCTCTATTATCACAACCTTGCTCATATTAACGATACCAATTATCCTTAC
CGGCGCTTCAACACACAAAAATAAATTCTACCCACAGTATGTAAAAACTACAATTTCCTATG
CTTTCGCAGTCAGCACTGTTCCTATAATGATATTCCTCTACTCAGGGCAAGAGATAATTATCT
CAAACTGACATTGAATAACTATTCAAACCCTAAAGCTAACTCTAAGCTTTAAGCTAGACTAT

TTCTCGATAATTTTTATACCAGTAGCCCTGTTCGTCACATGATCCATCATAGAATTTTCTATTT
GATATATATACACTGACCCCAACATCAACCGATTCTTCAAATATCTACTTATATTCCTCATTA
CTATAATAATTTTAGTAACCGCAAATAATCTGTTCCAACTATTCATCGGCTGAGAAGGGGTA
GGAATCATATCTTTCCTCCTTATTGGGTGATGATACGGACGAACAGATGCCAATACAGCTGC
TCTACAGGCAGTCGTATATAACCGCATCGGAGACGTAGGCTTTATTATAGCTATAGCCTGAT
TTTTACTCAATACAAATGCATGAGACTTCCAACAAATCTTTGCAATCGAACATGACAACCTT
AATATCCCACTAGCAGGACTACTCCTAGCAGCCACTGGAAAATCAGCCCAATTCGGCCTTCA
TCCATGACTTCCATCAGCTATAGAAGGACCCACTCCTGTTTCAGCCCTACTCCACTCCAGTAC
AATAGTAGTAGCAGGGGTATTTCTTCTAATCCGATTTCACCCCTTAATGGAACATAATAAGA
CCATCCAAACAGCCGCCCTATGCTTAGGAGCAATAACAACCCTATTTACAGCAGCCTGCGCT
CTCACCCAAAATGACATTAAAAAGATTATTGCCTTCTCCACCTCAAGCCAACTAGGATTAAT
AATCGTAACAATTGGGATTAACCAACCCCATTTAGCATTTCTTCATATCTGCACACACGCATT
TTTCAAGGCCATACTGTTTATATGCTCTGGATCCATTATCCACAGTCTAAATGACGAACAAG
ACATCCGAAAAATAGGAGGCCTATTCAAAGTCCTACCACTTACCACCACTGCACTAATTATT
GGAACTCTAGCACTCACAGGCATACCTTTCCTAACAGGATTTTACTCTAAAGACCTAATTAT
CGAGACCGCCAATACATCGTATACCAACGCCTGAGCCCTACTAATAACTCTCGTCGCCACAT
CCATAACCGCTGCCTACAGTACTCGAATTGTATTTTTCGCACTTCTAGGACAACCTCGCTTTA
ACCCTGCTATTATCATCAACGAGAATAATCCTCTCTTGGTTAACTCCATCAAGCGTCTACTTC
TAGGAAGTATTTTCGCAGGATACCTAATCTCCCACAATATTACACCTACTACCATCCCACAA
ATAACCATACCCTACTATCTAAAAACAACAGCCCTCGCGGTCACTATACTAGGCTTTATCCT
AGCACTAGAACTTAATACTACCATACAAAGTCTCAAATTTAACTATCCACAAAATGTATTCA
AATTCTCCAATCAACTAGGGTACTTCCCCATTATTATTCACCGCTTCCTACCAACAATGAGCT
TATCAATAAGCCAAAAGATGGCATCTATATTACTAGACACAATCTGACTAGAAAATGTATTA
CCAAAATCCATCTCCTACTTCCAAATAAAATCTTCAATCACCGTCTCCAACCAGAAAGGACT
AATCAAACTATACTTCCTCTCTTTCATGATCACACTAACCCTAAGCTTCCTCGTACTTAATTT
CCACGAGTGACTTCCATAATTACTAATACACCAATAAGAAGAGACCACCCAGTAACAATAA
CTAGCCAAGTCCCATAACTATACAAAGCCGCAATCCCCATGGCCTCCTCACTAAAAAACCCT
GAATCCCCCGTGTCATAAATTACTCAATCACCTACCCCATTAAACTTAAATACAATCTCAACT
TCATCATCTTTTAGAACATAGCAGGCAATCAGTAACTCAGATAATAAACCCACAATAAACGC
ACCTAATACAGCTTTATTAGAAACCCAGACCTCAGGATACTGCTCGGTAGCCATAGCCGTGG
TATAACCAAAAACCACAAGCATCCCACCCAAATAAACTAAAAAAACTATCAAACCTAAAAA
AGACCCCCCAAAACTCAATACAATACCACACCCAACACCCCCACTAATAATTAAAACCAATC
CTCCATAAATTGGAGAAGGCTTAGAAGAAAACCCCACAAAACTCATCACAAAGACAATACT
TAAAATAAACACGATATACATTATCATTATTCTCACATGGAATCTAACCATGACTAATGACA
TGAAAAATCACCGTTGTACTTCAACTACAAGAACCTTAATGACCAACATCCGAAAAACCCAT
CCATTAGCCAAAATCATCAACAACTCACTCATTGATCTCCCAGCACCATCAAATATCTCAGC
ATGATGAAACTTTGGATCCCTCCTCGGAGTATGCCTAATCCTACAGATTCTGACAGGCCTATT
TCTAGCTATACACTACACATCAGACGCGACTACAGCCTTTTCATCAGTCGCCCATATTTGCCG
AGACGTCCATTACGGATGAATTATCCGATACATACATGCAAACGGAGCCTCCATGTTCTTCA
TCTGCCTATTCATACACGTAGGACGGGGCTTGTATTATGGCTCATACCTACTCTCAGAAACAT
GAAACATTGGCATCATCCTCCTATTTACAGTTATAGCCACCGCATTCATAGGATACGTCCTAC
CCTGAGGCCAAATATCCTTCTGAGGAGCGACTGTCATTACCAACCTCCTATCAGCCATTCCCT
ATATTGGAACGGATCTAGTAGAGTGAATCTGAGGGGGCTTTTCCGTAGATAAAGCAACCCTA
ACACGATTCTTTGCTTTCCACTTTATCCTTCCATTTATCATCCTAGCACTAGCAGCAGTTCATC
TATTGTTCCTACACGAAACAGGATCCAACAACCCCTCTGGAATCCCATCCGACTCGGACAAA
ATCCCATTCCACCCATACTATACAATTAAGGACGCCCTAGGCGCCCTACTTCTCATTCTAGCC
CTAGCAACTCTAGTTCTATTCTCGCCCGACTTACTAGGAGACCCTGACAACTATACCCCCGC
AAACCCACTGAGCACCCCGCCCCACATCAAGCCCGAGTGATACTTTCTATTTGCTTACGCTA
TCCTACGATCCATCCCCAACAAACTAGGAGGAGTACTAGCGCTAATCTTCTCTATCCTAATC
CTAGCCATTATCCCCCTTCTACACACATCTAAACAACGAGGAATAATGTTCCGACCCCTAAG
CCAATGCCTATTTTGACTCCTAGTAGCAGACCTACTAACACTAACATGAATCGGAGGACAAC

CAGTAGAACATCCCTTCACCATTATCGGACAACTAGCCTCCATCCTCTACTTCACAATCCTCC
TGGTGCTCATGCCCATCGCTGGAATCATTGAAAACAACCTCTCAAAATGAAGAGTCTTTGTA
GTATAGTAATTACCTTGGTCTTGTAAGCCAAAAACGGAGAATATCTACCCTCCCCAAGACTC
AAGGAAGAAGCAACAGCCCCACCATCAGCACCCAAAGCTGATGTTCTATTTAAACTATTCCC
TG
>SRR6109214 Circular ReOrganized
GTACATACCATTATTTCACTTTATGTCCTATTTATTTCATATATATCATTCCATGTACTGTACC
ATCACAGTATGTTCTCGAATACTTTCTCCTTTGTTTTTTTCCCCCCCTATGTATATCGTGCAT
TAATGGCGTGCCCCATGCATATAAGCATGTACATACTATGCTTGGCTTTACATGAGGACTTA
CGTTCCGAAAAGTTTATTTCAGGCGTATAGTCTGTAAGCATGTATTTCACTTAGTCCGGGAGC
TTAGTCACCAGGCCTCGAGAAACCAGCAATCCTTGCGAGTACGTGTACCTCTTCTCGCTCCG
GGCCCATGGAATGTGGGGGTTTCTATGTTGAAACTATACCTGGCATCTGGTTCTTACCTCAG
GGCCATGATAGCTCTAGATTCCAATCCTACTAACCCTTCAAATGGGACATCTCGATGGACTA
ATGACTAATCAGCCCATGATCACACATAACTGTGGTGTCATGCATTTGGTATCTTTTAATTTT
TAGGGGGGGAACTTGCTATGACTCAGCTATGACCGTAAAGGTCTCGTCGCAGTCAAATAATT
TGTAGCTGGGCTTATTTATCTTTCACGGGTCGGGCATAGATACCCATAAGGGGCTATTCAGT
CAATGGTTACGGGGCATATAGTATGTAAATACCACTGAATCGAACGAACACGCACGTGTAC
GCATATGTACGCATATGTACGCATATGTACGCATGTGTACGCATGTGTACGCATGTGTACGC
ATGTGTACGCATGTGTACGCATGTGTACGCATGTGTACGCATGTGTACGCACGTGTACGCAC
GTGTACGCACGTGTACGCACGTGTACGCACGTGTACGCACGTGTACGCACGTGTACGCACGT
GTACGCACGTGTACGCACGTGTACGCACGTGTACGCACGTGTACGCACGTGTACGCACGTGT
ACGCGCGCGTTTTAGATATTAACTTAGCTTAATCAAACCCCCCTTACCCCCCGTAACTTCAA
GGAGCTTACGCACATTTATGATTGTCCTGCCAAACCCCAAAAACAAGACTAAATGCACGTGC
AAACATGAAGCTATTACACCCAAAATCCCACATAATAAGCTAAAATTCCCAGCCAAATAATC
ACTACAATCACAGGCATGAGACTTTAAATTAAGATCTATCTATAGATATTTTTTTTTTTACTC
TGTCTCCCTCCTATTGATTTTTCCGTTATTATCACTTTTTTAGCCACGAGCTTCTACTAAATTA
TCCCATATAAATCCCAAAACCAACCGCGTTTATGTAGCTTAATAATAAAGCAAGGCACTGAA
AATGCCTAGACGAGTTGTATAACCCCATAGACATAAAGGTTTGGTCCTAGCCTTCCCATTAG
CTATTAACAAGATTACACATGTAAGTCTCCGCGCTCCAGTGAAAATGCCCTTTGGATCTTAA
AGTGATCTGAAGGAGCGGGCATCAAGCACACCCCTCCCCGGTAGCTTATAACGCCTTGCTCA
GCCACACCCCCACGGGATACAGCAGTGATAAAAATTAAGCCATAAACGAAAGTTTGACTAA
GCTATGTTGATTAAAGGGTTGGTTAATTTCGTGCCAGCCACCGCGGTTATACGATTGACCCA
AGTTAATAGGCCCACGGCGTAAAGCGTGTGAAAGAAAAAAATTTTTCCCCATTAAAGTTAA
AGTTTAATCAAGCTGTAAAAAGCTACCAATAACACTAAAATAGACTACGAAAGTGACTTTA
ATACTCTCAACCACACGACAGCTAAGATCCAAACTGGGATTAGATACCCCACTATGCTTAGC
CTTAAACATAAATAATTTATCAAACAAAATTATTCGCCAGAGAACTACTAGCAACGGCTTAA
AACTCAAAGGACTTGGCGGTGCTTTAAACCCCCCTAGAGGAGCCTGTTCTGTAATCGATAAA
CCCCGATAGACCTCACCACCCCTTGCTAATCCAGTCTATATACCGCCATCTTCAGCAAACCCT
TAAAAGGAAAAAGAGTAAGCACAATCATCTTGCATAAAAAAGTTAGGTCAAGGTGTAACTC
ATGGGATGGGAAGAAATGGGCTACATTTTCTATTCAAGAACAACCTACGAAAGTTTTTATGA
AACTAAAAACTAAAGGTGGATTTAGTAGTAAACCAAGAATAGAGAGCTTGGTTGAATAAGG
CAATGAAGCACGCACACACCGCCCGTCACCCTCCTCAAGTGGCACAAGTCAAATATAACCTA
TTAAAACCAAATAAAACGCAAGAGGAGACAAGTCGTAACAAGGTAAGCGTACTGGAAAGT
GTGCTTGGATAAACCAAAGTGTAGCTTAAACAAAGTATCTGGCTTACACCCAGAAGATTTCA
CGTATGTGACCGCTTTGAGCCCAGAGCTAGCCCAGACAATAACCGATTAAACTACCACAGGC
CAATTAAATAAAACATTCAGTAGCGCAATTAAAGTATAGGAGATAGAAATTCTTTAGATCGG
AGCTATAGAGAAAGTACCGCAAGGGAACGATGAAAGATTACCCAAAGTGATAAACAGCAA
AGATTACCCCTTCTACCTTTTGCATAATGAGTTAGCCAGAAATAACCTAACAAAGAGAACTT
AAGCTAGGTCCCCCGAAACCAGACGAGCTACCTATGAACAATCCACTGGGGTAAACTCATCT
ATGTTGCAAAATAGTGAGAAGATTTGTAGGTAGAGGTGAAAAGCCTAACGAGCCTGGTGAT
AGCTGGTTGCCCAGAATAGAACTTCAGTTCAACTTTAAACTTGCCTACAAAACTTAGATAAT

TTTAATGCAAGTTTAAAATATATTCTAAAAAGGTACAGCTTTTTAGAATCAAGGATACAACC
TTACTTAGAGAGTAAATACTGATTAAACCATAGTAGGCCTAGAAGCAGCCATCAATTAAGA
AAGCGTTTAAGCTCAACATCCATGTCAACTTAATACCAAAAGTATTTAATCAACTCCTAATA
TAACAACTGGGCTAATCTATTTTAATATAGAAGCAATAATGCTAATATGAGTAACGAGAGAT
ACCTCTCCAACGCATAAGCCTATAACAGCAACGGATAACCACTGATAGTTAACAACAACGT
AGAAATAACCCAACAATAAAACATCTACCAAACCAATTGTTAATCCAACACAGGTATGCGA
CTAAGGAAAGATTAAAAGAAGTAAAAGGAACTCGGCAAACACAAATCCCGCCTGTTTACCA
AAAACATCACCTCCAGCATTCCCAGTATTGGAGGCACTGCCTGCCCGGTGACATTAGTTAAA
CGGCCGCGGTATTCTGACCGTGCAAAGGTAGCATAATCATTTGTTCTCTAAATAAGGACTTG
TATGAAAGGCCACACGAGGGTTTAACTGTCTCTTACTTCCAATCAGTGAAATTGACCTTCCC
GTGAAGAGGCGGGAATAAAACAATAAGACGAGAAGACCCTATGGAGCTTTAATTAACTAGC
TCAAAAGAATCTATCTACCAGACCGACAGGAACAACATACTCCTTCCATGAGCTAACAATTT
AGGTTGGGGCGACCTCGGAGCATAAAACAACCTCCGAGTGATATTAATCTAGACGTACCAG
TCAAAATGCTCACTTACTTATTGATCCAAAAACCTCTTTGATCAACGGAACAAGTTACCCTA
GGGATAACAGCGCAATCCTATTTAAGAGTCCATATCGACAATAGGGTTTACGACCTCGATGT
TGGATCAGGACATCCTAATGGTGCAGCAGCTATTAAGGGTTCGTTTGTTCAACGATTAAAGT
CCTACGTGATCTGAGTTCAGACCGGAGCAATCCAGGTCGGTTTCTATCTATTTAAACAACCT
CTCCCGGTACGAAAGGACAAGAGAAGTAAGGCCTCCCTCACTGAGGCGCCTTAAGACCAAT
AGATGATTTAATCTAAATCCAGTAAGTCTATCCCCAATACTGCCCAAGAGACAGGGCTTTGT
TAGGGTGGCAGAGCCCGGTGATTGCATAAAACTTAAACCTTTATATTCAGGGGTTCAAATCC
TCTCCCTAACATTATGTTTATAATCAACACTATCTCACTAGTTGTACCCATTCTCCTTGCCGTA
GCCTTCTTAACACTAGTAGAGCGAAAAGTACTAGGCTATATACAACTTCGTAAAGGACCAAA
CATTGTAGGGCCCTACGGTCTCCTACAACCTGTCGCAGATGCTGTAAAACTTTTCACCAAAG
AGCCTCTACGCCCACTCACATCATCTACGACCATATTCATTATGGCCCCTATTCTGGCTCTAA
CACTAGCCCTAACCATGTGAATTCCCCTACCAATGCCATATCCCCTTGTCAATATAAACCTCG
GAGTACTATTCATACTAGCAATATCAAGCCTAGCTGTATACTCTATCCTTTGATCAGGATGA
GCTTCAAATTCAAAATACGCTCTGATCGGAGCCCTACGGGCCGTAGCCCAGACAATTTCATA
TGAAGTCACATTAGCTATTATCCTCCTATCAGTCCTACTAATGAATGGCTCATTCGCACTTTC
CACTTTAATTACTACTCAAGAGCACCTCTGACTAATCTTCCCTGCATGACCCCTAGCCATGAT
ATGATTTATTTCCACCCTAGCAGAAACCAACCGAGCCCCCTTCGACCTAGCAGAAGGAGAAT
CAGAACTGGTCTCAGGTTTTAACGTTGAGTACGCGGCAGGTCCGTTCGCCCTATTTTTCCTAG
CAGAATATACTAATATCATTATAATAAATGCCCTCACGACTATCCTGTTCTTCGGGGCATTTC
ATAATCCCTACATACCAGAACTGTACACTGTCAATTTCACCGTAAAAACACTGCTCTTAACA
GCTTCCTTCCTATGAATCCGTGCATCATATCCCCGATTCCGATATGACCAACTAATGCATCTG
CTATGAAAAAACTTCCTACCCCTTACACTAGCCCTGTGCATGTGACATGTAACACTACCCATT
ATCACAGCAAGCATTCCCCCCCAAACATAAGAAATATGTCTGACAAAAGAATTACTTTGATA
GAGTAAATAATAGAGGTTGGAATCCTCTTATTTCTAGAATTATAGGAATTGAACCTAATCCT
AAGAACTCAAAAATCTTCGTGCTACCTAATTACACCATATCCTAAAGTAAGGTCAGCTAAAT
AAGCTATCGGGCCCATACCCCGAAAATGTTGGTTTATCCCCTTCCCGTACTAATTAAACCTCC
CATTCTTATCATTATCACATCCACCATTGTCCTAGGAACCATAATCGTTTTATTTAGCTCCCA
TTGACTCATAATCTGAATTGGCTTTGAAATAAATATACTAGCTATTATCCCAATCCTAATGAA
AAAATTTAACCCACGAGCCATAGAAGCCTCTACAAAATATTTCCTCACACAAGCCACTGCAT
CCATGCTTCTAATACTAGGCATTATTATCAACCTACTACACTCAGGGCACTGAACAGTCTCA
ATAATCCCTAACCCAATCGCATCAACCGTAATTACCATTGCCCTAGCAATAAAACTCGGCCT
ATCCCCCTTCCACTTCTGAGTCCCAGAAGTCACACAAGGCGTTCCTTTATCCTCAGGAATAAT
TCTACTAACATGACAAAAAATCGCACCTCTATCCGTCTTATATCAAATCTCACCATCCATCAA
CCCAAACCTACTGGTAGCAATAGCCGCCACATCCGTATTAGTAGGGGGCTGAGGAGGATTA
AACCAAACTCAACTTCGAAAAATCCTAGCATACTCTTCAATCACTCACATGGGCTGAATAGC
CGCCATCATAGTATATAACCCCACTCTAATAATCCTTAACCTTGCAATCTATATCATAATAAC
TCTGGGGACATTTATACTATTTATGCACAGCCTATCCACAACAACACTATCACTGTCCCATAC
ATGAAATAAACTACCACTAATTGCCTCACTAATTCTAGCACTTATATTATCACTAGGCGGCCT

CCCTCCCCTCTCAGGCTTCATCCCCAAATGAATAATTATTCAAGAACTAACAAAAAATGATA
TAATCGTCATGCCCACATTCATAGCCATCACAGCCTTACTAAATCTTTATTTTTACATGCGTC
TAACATACGCCACAGCATTAACAATATTCCCCTCAGCAAATAACATAAAAATAAAATGACA
GTTTGAAAGCACAAAAAAAATAATTCTCTTGCCCCCTCTAATCGTAGCATCAACCATACTCC
TCCCACTAACTCCATTACTATCAATCATGGACTAGAGATTTAGGCTAAAAAGACCAAGGGCC
TTCAAAGCCCTAAGTAAGTGAAACCCACTTAATCTCTGCAATTATCTAAGGACTGCAAGAAC
ATATCTCACATCAATTGAACGCAAAACAACCGCTTTAATTAAGCTAAGCCCTCCTAGATTGG
TGGGCTTTTATCCCACAAAATTTTAGTTAACAGCTAAAAACCCCAAACAACTGGCTTCAATC
TACTTCTCCCGCCGCGTGGGAAAAAAAGGCGGGAGAAGCCCCGGCAGAATTGAATCTGCTT
CTTTGAATTTGCAATTCAATATGATTATTCACCACAGAGCTTGGCAAAAAGGGGACTCAACC
TCTATTCTTAGATTTACAGTCTAGTGCTTTTATCAGCCATTTTACCCATGTTCATAAACCGAT
GACTATTCTCTACAAATCATAAAGATATTGGCACTCTCTATCTTCTATTCGGTGCATGAGCCG
GAATAGTAGGTACTGCTCTCAGCCTTTTGATTCGTGCCGAACTAGGTCAGCCCGGGACCCTG
TTGGGGGATGATCAGATCTACAATGTAGTCGTAACTGCCCATGCATTCGTGATAATCTTCTTT
ATGGTTATGCCTATTATAATTGGGGGGTTTGGGAACTGATTAGTGCCCTTAATAATTGGTGCT
CCTGACATAGCGTTTCCTCGAATGAATAACATAAGCTTCTGACTGCTGCCACCATCTTTCTTG
TTGCTTCTAGCTTCTTCTATAGTAGAAGCAGGTGCAGGAACTGGGTGGACTGTCTACCCCCCT
CTAGCGGGCAATCTGGCCCATGCAGGAGCATCAGTAGACCTAACAATCTTTTCTCTACACTT
AGCAGGCATCTCTTCTATTCTGGGAGCCATCAATTTCATCACTACTATCATTAACATAAAACC
TCCTGCAATATCTCAATACCAAACCCCTCTGTTTGTGTGATCAGTCCTAATCACGGCAGTGCT
TCTTCTTTTATCTCTGCCAGTCTTGGCGGCTGGGATTACCATACTACTTACAGATCGAAACCT
CAACACTACCTTTTTTGACCCAGCCGGAGGGGGAGATCCTATTCTGTACCAACACTTGTTTTG
ATTCTTCGGACACCCTGAAGTTTATATCCTAATTCTTCCAGGGTTCGGAATGATCTCTCACAT
TGTCACTTATTACTCAGGAAAAAAAGAGCCTTTCGGCTACATGGGTATAGTCTGAGCGATAA
TATCCATTGGATTCTTAGGATTTATCGTGTGAGCTCATCATATGTTTACCGTAGGTATAGATG
TCGACACACGAGCTTACTTCACTTCAGCTACCATAATTATTGCAATCCCAACAGGAGTCAAA
GTATTTAGCTGACTAGCTACCCTGCACGGAGGGAATATTAAATGATCTCCTGCTATAATATG
AGCCCTAGGCTTTATTTTTCTGTTTACAGTAGGGGGCCTTACAGGAATTGTCCTAGCTAATTC
GTCTCTAGACATTGTTCTCCATGACACATATTATGTGGTAGCCCATTTCCACTATGTGCTGTC
AATAGGAGCTGTCTTTGCCATCATAGGAGGATTTGTCCACTGATTTCCACTGTTTCAGGCTA
TACACTTAATAATACATGAGCAAAGATTCACTTCATAATCATATTCGTTGGGGTTAATATAA
CTTTCTTTCCCCAACATTTCCTGGGCCTGTCGGGAATGCCTCGGCGATATTCCGACTATCCAG
ATGCCTACACAATATGAAATACAGTATCTTCTATAGGCTCATTCATTTCACTAACAGCAGTTA
TACTAATAATTTTCATGATTTGGGAGGCCTTTGCATCAAAGCGAGAGGTAGCAGTGGTAGAA
CTCACTTCAACCAATATTGAATGACTACATGGATGTCCTCCTCCGTACCATACATTTGAAGA
ACCCGCTTACGTTATACTAAAATAAGAAAGGAAGGAATCGAACCCTCTGGAATTGGTTTCAA
GCCAATATCATAACCACTATGCCTCTCTCAATAAAGAGATATTAGTAAAAATTACATAACTT
TGTCAGGGTTAAATTATAGGTGAAAATCCTTTATATCTTTATGGCATATCCCCTTCAAATAGG
TCTTCAAGACGCAACTTCTCCTATCATGGAGGAACTCCTACATTTTCATGACCATACACTAAT
AATTGTATTCCTAATTAGCTCCTTAGTTCTCTATATTATTTCAATTATATTAACTGCCAAATTA
ACACACACAAATACAATAGATGCACAAGAAGTAGAGACGGTATGAACCATCTTGCCGGCTA
TTATTCTAATTCTAATTGCACTCCCATCGCTACGGATTCTTTATATAATAGATGAAATCAATA
ACCCCTCACTGACCGTAAAAACCATGGGCCATCAATGATATTGAAGTTACGAGTATACAGAT
TACGAAGATCTGAGCTTTGACTCTTATATAACCCCAACACAAGAACTAAAACCTGGAGAACT
GCGACTATTAGAAGTGGACAATCGAGTAGTACTGCCCATAGAAATAACGATCCGTATGCTG
ATCTCATCAGAAGATGTCTTGCACTCATGGGCTGTGCCATCCCTAGGATTAAAAACTGATGC
AATCCCAGGACGGCTAAACCAAACAACTCTCATAGCTATACGACCAGGATTATACTACGGCC
AATGCTCAGAAATTTGTGGCTCCAATCACAGTTTTATACCTATTGTTCTCGAGCTAGTTCCAC
TATCTTACTTTGAAAAATGGTCTGCCTCGATACTATAGAATCATTAAGAAGCTAATATAGCG
TTAACCTTTTAAGTTAAAGACTGAGAATGCAGACTTCTCCTTAATGAAGATGCCACAACTAG
ACACATCAACATGATCTATCACAATCCTATCTATAGTTCTAGCACTATTCATTATATTCCAGT

TAAAGATCTCAAAATATAAATACCCCAAAACCCCCGAACCGAAATCCCCTTCACCATCAAAG
AAACTTATACCTTGAGAAGAAAAATGAACGAAAATCTATTCACCTCTTTTATTACCCCGATG
ATAATGGGGGTCCCCATTGTAGTCTTAATCGTTATGTTTCCGAGCATTTTATTCCCTTCTCCC
AGTCGACTGATCGACAATCGCTTAGTGTCTATTCAACAATGACTAGTCTGGCTAACATCAAA
ACAAATGCTATCCATTCATAATCGTAAAGGGCAAACCTGAGCACTAATGTTGATATCCCTAA
TCCTATTTATTGGCTCAACTAATCTTCTGGGTCTATTACCACACTCATTCACACCTACCACAC
AACTGTCAATGAATCTGGGAATGGCCATTCCCTTGTGAGCAGGTACAGTTGCCACTGGATTT
CGATATAAGACCAAAGCATCCTTAGCTCACTTTCTACCTCAAGGAACACCTTTCCCTCTAATC
CCTATACTTGTTATTATCGAAACAATCAGTCTATTCATCCAACCTATGGCCTTAGCCGTACGA
TTAACTGCTAATATTACTGCAGGTCACTTGCTCATCCATTTAATTGGAGGGGCTACTCTAGCT
CTAACAAGTATTAGCACTATTACAGCCCTCATCACCTTTATTATTCTAGTGCTACTTACTATT
CTCGAGTTTGCTGTAGCCCTCATTCAGGCCTACGTCTTTACCCTGCTAGTAAGCCTATACTTA
CATGATAACACTTAATGACTCACCAAACACATGCATATCATATAGTTAACCCAAGTCCATGA
CCACTAACAGGAGCCCTTTCAGCCCTTCTTATAACATCAGGACTAATCATATGATTCCACTTT
AACTCTATATTCTTGCTATCGCTAGGCCTTACAACCAACATACTTACTATATACCAATGATGA
CGGGATATTATTCGAGAGAGTACCTTTCAGGGTCATCACACCCCCGTTGTCCAAAAAGGACT
GCGGTACGGAATAGTCCTATTTATCGTATCAGAAGTATTCTTCTTCGCAGGGTTCTTCTGAGC
TTTTTATCACTCAAGCCTAGCACCCACTCCCGAACTGGGGGCATGCTGACCACCCACAGGTA
TTACTCCCCTGAACCCGCTAGAAGTACCACTTCTCAATACTTCAGTACTTCTTGCATCCGGAG
TGTCCATTACCTGAGCTCACCACAGCTTAATAGAGGGAAGCCGCAAGCACATGCTTCAGGCC
CTATTTATTACTATCTCTCTAGGCGTGTACTTCACACTTCTACAAGCCTCAGAGTATTATGAG
GCGTCCTTCACTATCTCAGATGGAGTCTATGGCTCAACTTTCTTCATAGCGACTGGATTTCAC
GGACTTCATGTAATTATTGGCTCAACTTTTCTCACAGTCTGTTTCCTACGGCAACTACATTTT
CATTTTACATCAAGCCACCATTTCGGCTTTGAAGCAGCAGCATGATATTGACACTTCGTAGA
TGTCGTATGACTATTCCTATACGTTTCTATCTATTGATGAGGGTCTTGCTTCTTTAGTATCGAT
CAGTACAATTGACTTCCAATCAGTTAGCTCTGGTGTAATCCAGAAAGAAACAATCAACTTAA
TCCTAGCACTATTTACTAACATACTACTAGCTTCCTTACTCGTCCTTATTGCCTTCTGACTACC
ACAATTAAATATCTATGCAGAAAAAGCAAGCCCCTATGAATGCGGGTTTGACCCCGTAGGAT
CGGCACGCCTCCCTTTTTCAATAAAATTTTTCCTAGTAGCTATCACATTTCTACTATTTGACCT
AGAAATTGCGCTACTCCTCCCCCTTCCCTGAGCATCACAAACTGACAATCTAATAACGATAC
TCATCATAGCACTACTGCTTATCTCCCTATTAGCCGCAAGTCTGGCCTATGAATGAGCCGAA
AAAGGTCTAGAATGGGCCGAATATGATAATTAGTTTAAACCAAAACAAATGATTTCGACTCA
TTAGATTATGAATAATAACATAATTATCAAATGCCTGTGGTCTATGTCAATATTTTCCTAGCC
TTCATCGTATCCCTAATAGGGCTACTTATTTACCGATCACATCTAATGTCCTCCCTACTTTGC
CTGGAGGGTATAATGCTATCTTTATTTGTCATACTAACAGTAACAGTTTTAAATAATCATTTT
ACACTAGCTAACATAGCTCCCATTATCTTACTAGTATTTGCTGCCTGCGAAGCAGCACTAGG
ACTATCCCTGCTAGTAATAGTCTCCAACACTTATGGAACCGATTACGTACAAAATCTTAATCT
CTTACAATGCTAAAAATTATCTTTCCCACTATAATACTAGTGCCCCTTACATGAGCATCAAAA
CCCAGTATAATCTGGATTAATACAACAGCTTACAGCCTACTCATTTGTCTTATCAGCTTGACA
TATTTCAACCAGCTCTGTGATAATAGCCTAAACTTCTCATTATTATTCTTTACCGACTCCCTAT
CAGCCCCCTTACTAGCTCTTACAACATGACTACTGCCTCTGATACTAATAGCAAGTCAATCCC
ACCTATCAAAGGAGACCCTGACCCGAAAGAAACTATATATCACTATACTAGTCCTCCTACAG
CTATTCCTGATTATAACCTTTTCTGCTATAGAGCTAATCCTGTTCTATATCTTGTTCGAAGCCA
CCCTAATACCCACTCTAATTATTATTACTCGATGAGGGAATCAAACAGAACGATTAAACGCG
GGCCTTTACTTTCTATTTTATACTCTAGTAGGATCTTTACCACTGCTAATTGCACTACTACATA
CTCAAAACAATCTAGGTACCCTAAATCTTCTCATAATACAATACTGAATCCAGCCTCTGCCA
AACTCCTGATCCAGTGTCTTCCTGTGACTAGCATGTATAATAGCATTCATAGTAAAAATACCT
CTGTATGGCCTTCACTTGTGATTGCCAAAAGCACATGTAGAAGCCCCTATCGCAGGATCTAT
AGTGCTTGCCGCCGTACTCCTAAAACTAGGAGGCTACGGCATGATACGAATTACAACACTAC
TTGACCCTCTAACAAGCTTCATGGCCTACCCCTTCATAATACTCTCACTATGGGGCATAATCA
TAACAAGTTCTATTTGCCTGCGCCAAACGGATTTAAAGTCCCTAATTGCTTACTCCTCTGTTA

GTCATATAGCCCTAGTCATTGTAGCAGTATTGATCCAAACTCCATGAAGCTATATAGGAGCA
ACAGCCTTAATAGTTGCCCATGGACTAACATCCTCCATGCTATTCTGTCTTGCAAACTCCAAC
TATGAACGAGTCCATAGCCGAACTATAATCCTTGCGCGAGGTCTACAAACACTCCTTCCACT
TATAGCAGCTTGATGACTACTAGCAAGTCTTACTAATCTGGCACTCCCTCCTACCATCAATCT
CATCGGAGAACTATTTGTGGTAGTAGCCACATTTTCATGATCTAACATCACCATCACTCTAAT
AGGAGCCAACATCATCATTACTGCCCTCTATTCTCTCTACATACTAATCACCACACAACGAG
GCAAGTGCACAGACCATATCAAAAGCATCAAGCCATCCTTCACACGAGAAAACGCCCTAAT
AGCTCTCCACCTCCTACCCCTTCTACTTCTATCTCTCAACCCCAAAGTAATTCTGGGGCCCAT
TTACTGTAAATATAGTTTAACAAAAACATTAGATTGTGAATCTAATAATAAAAGCCCAGGCC
TTTTTATTTACCGAAAAAGTACGCAAGAACTGCTAATTCATGCATCCATGCATAAAATCATG
GCTTTTTCAACTTTTAAAGGATAGAAGTAATCCATTGGTCTTAGGAATCAAAAAATTGGTGC
AACTCCAAATAAAAGTAATTAATCTGTTTGCCTCCTCTATTATCACAACCTTGCTCATATTAA
CGATACCAATTATCCTTACCGGCGCTTCAACACACAAAAATAAATTCTACCCACAGTATGTA
AAAACTACAATTTCCTATGCTTTCGCAGTCAGCACTGTTCCTATAATGATATTCCTCTACTCA
GGGCAAGAGATAATTATCTCAAACTGACATTGAATGACTATTCAAACCCTAAAGCTAACTCT
AAGCTTTAAGCTAGACTATTTCTCGATAATTTTTATACCAGTAGCCCTGTTCGTCACATGATC
CATCATAGAATTTTCTATTTGATATATACACACTGATCCCAACATCAACCGATTCTTCAAATA
TCTACTTATATTCCTCATTACTATAATAATTTTAGTAACCGCAAATAATCTGTTCCAACTATT
CATCGGCTGAGAAGGGGTAGGAATCATATCTTTCCTCCTTATTGGGTGATGATACGGACGAA
CAGATGCCAATACAGCTGCTCTACAGGCAGTCGTATATAACCGCATCGGAGACGTAGGCTTT
ATTATAGCTATAGCCTGATTTTTACTCAATACAAATGCATGAGACTTCCAACAAATCTTTGCA
ATCGAACATGACAACCTTAATATCCCACTAGCAGGACTACTCCTAGCAGCCACTGGAAAATC
AGCCCAATTCGGCCTTCATCCATGACTTCCATCAGCTATAGAAGGACCCACTCCTGTTTCAGC
CCTACTCCACTCCAGTACAATAGTAGTAGCAGGGGTATTTCTTCTAATCCGATTTCACCCCTT
AATGGAACATAATAAGACCATCCAAACAGCCGCCCTATGCTTAGGAGCAATAACAACCCTA
TTTACAGCAGCCTGCGCTCTCACCCAAAATGACATTAAAAAGATTATTGCCTTCTCCACCTCA
AGCCAACTAGGATTAATAATCGTAACAATTGGGATTAACCAACCCCATTTAGCATTTCTTCA
TATCTGCACACACGCATTTTTCAAGGCCATACTGTTTATATGCTCTGGATCCATTATCCACAG
TCTAAATGACGAACAAGACATCCGAAAAATAGGAGGCCTATTCAAAGTCTTACCACTTACCA
CCACTGCACTAATTATTGGAACTCTAGCACTCACAGGTATACCTTTCCTAACAGGATTTTACT
CTAAAGACCTAATTATCGAGACCGCCAATACGTCGTATACCAACGCCTGAGCCCTACTAATA
ACTCTCGTCGCCACATCCATAACCGCTGCCTACAGTACTCGAATTGTATTTTTCGCACTTCTA
GGACAACCTCGCTTTAACCCTGCTATTATCATCAACGAGAATAATCCTCTCTTGGTTAACTCC
ATCAAGCGTCTACTTCTAGGAAGTATTTTCGCAGGATACCTAATCTCCCACAATATTACACCT
ACTACCATCCCACAAATAACCATACCCTACTATCTAAAAACAACAGCCCTCGCGGTCACTAT
ACTAGGCTTTATCCTAGCACTAGAACTTAATACTACCATACAAAGTCTCAAATTTAACTATCC
ACAAAATGTATTCAAATTCTCCAATCAACTAGGGTACTTCCCTATTATTATTCACCGCTTCCT
ACCAACAATGAGCTTATCAATAAGCCAAAAGATGGCATCTATATTACTAGACACAATCTGAC
TAGAAAATGTATTACCAAAATCCATCTCCTACTTCCAAATAAAATCTTCAATCACCGTCTCCA
ACCAGAAAGGACTAATCAAACTATACTTCCTCTCTTTCATGATCACACTAACCCTAAGCTTCC
TCGTACTTAATTTCCACGAGTGACTTCCATAATTACTAATACACCAATAAGAAGAGACCACC
CAGTAACAATAACTAGCCAAGTCCCATAACTATACAAAGCCGCAATCCCCATGGCCTCCTCA
CTAAAAAACCCTGAATCCCCCGTGTCATAAATTACTCAATCACCTACCCCATTAAACTTAAA
TACAATCTCAACTTCATCATCTTTTAGAACATAGCAGGCAATCAGTAACTCAGATAATAAAC
CCACAATAAACGCACCTAATACAGCTTTATTAGAAACCCAGACCTCAGGATACTGCTCGGTA
GCCATAGCCGTGGTATAACCAAAAACCACAAGCATCCCACCCAAATAAACTAAAAAAACTA
TTAAACCTAAAAAAGGCCCCCCAAAACTCAATACAATACCACACCCAACACCCCCACTAAT
AATTAAAACCAATCCTCCATAAATTGGAGAAGGCTTAGAAGAAAACCCCACAAAACTCATC
ACAAAGACAATACTTAAAATAAACACGATATACATTATCATTATTCTCACATGGAATCCAAC
CATGACTAATGACATGAAAAATCACCGTTGTACTTCAACTACAAGAACCTTAATGACCAACA
TCCGAAAAACCCATCCATTAGCCAAAATCATCAACAACTCACTCATTGATCTCCCAGCACCA

TCAAATATCTCAGCATGATGAAACTTTGGATCCCTCCTCGGAGTATGCCTAATCCTACAGATT
CTGACAGGCCTATTTCTAGCTATACACTACACATCAGACGCGACTACAGCCTTTTCATCAGTC
GCCCATATTTGCCGAGACGTCCATTACGGATGAATTATCCGATACATACATGCAAACGGAGC
CTCCATGTTCTTCATCTGCCTATTCATACACGTAGGACGAGGCTTGTATTATGGCTCATACCT
ACTCTCAGAAACATGAAACATTGGCATCATCCTCCTATTTACAGTTATAGCCACCGCATTCAT
AGGATACGTCCTACCCTGAGGCCAAATATCCTTCTGAGGAGCGACTGTCATTACCAACCTCC
TATCAGCCATTCCCTATATTGGAACGGATCTAGTAGAGTGAATCTGAGGGGGCTTTTCCGTA
GATAAAGCAACCCTAACACGATTCTTTGCTTTCCACTTTATCCTTCCATTTATCATCCTAGCA
CTAGCAGCAGTCCATCTATTGTTCCTACACGAAACAGGATCCAACAACCCCTCTGGAATCCC
ATCCGACTCGGACAAAATCCCATTCCACCCATACTATACAATTAAGGACGCCCTAGGCGCCC
TACTTCTCATTCTAGCCCTAGCAACTCTAGTTCTATTCTCGCCCGACTTACTAGGAGACCCTG
ACAACTATACCCCCGCAAACCCACTGAGCACCCCGCCCCACATCAAGCCCGAGTGATACTTT
CTATTTGCTTACGCTATCCTACGATCCATCCCCAACAAACTAGGAGGAGTACTAGCGCTAAT
CTTCTCTATCCTAATCCTAGCCATTATCCCCCTTCTACACACATCCAAACAACGAGGAATAAT
GTTCCGACCCCTAAGCCAATGCCTATTTTGACTCCTAGTAGCAGACCTACTAACACTAACAT
GAATCGGAGGACAACCAGTAGAACATCCCTTCACCATTATCGGACAGCTAGCCTCCATCCTC
TACTTCACAATCCTCCTGGTGCTCATGCCCATCGCTGGAATCATTGAAAACAACCTCTCAAA
ATGAAGAGTCTTTGTAGTATAGTAATTACCTTGGTCTTGTAAGCCAAAAACGGAGAATATCT
ACCCTCCCCAAGACTCAAGGAAGAAGCAACAGCCCCACCATCAGCACCCAAAGCTGATGTT
CTATTTAAACTATTCCCTG

>SRR7477311 (circular) REORGANIZED
GTTAATGTAGCTTAAAACCAAAGCAAAGCATTGAAAATGCTTAGATGAGCCCACAAACTCC
ATAAACACACAGGTTTGGTCCCAGCCTTTTTATTAATTTTCAATAGGATTACACATGCAAGTA
TCCGCCCCCCAGTGAAAATGCCCTTCAAGTTATCACAGTACCTGAAGGAGCTGGCATCAAGC
ACGCCAATAAACGCAGCTAACGACGCCTTGCAGAGCCACACCCCCACGGGAAACAGCAGTG
ATAAAAATTAGGCTATAAACGAAAGTTCGACCTAGCCATATTGCATTGGGTTGGTAAATCTC
GTGCCAGCCACCGCGGTCATACGATTAACCCTAGCTAATAAATAACCGGCGTAAAGCGTGCT
AAGATGAATCCAATCCAAATAAAGTTAAGCCCTGACCAGGCCGTAAAAAGCCGTGGCTACC
GTAAAAATAAACTACGAAAGTAACTTTAATTCACATCGACACACGATAGCTAAGACCCAAA
CTGGGATTAGATACCCCACTATGCTTAGCCCTAAACTTAAATAATTCACCAGACAAAATTAT
TCGCCAGAGAACTACTGGCAACAGCCTAAAACTCAAAGGACTTGGCGGTGCTTTACATCCCC
CTAGAGGAGCCTGTTCTGTAATCGATAAACCCCGATAAACCCTACCAATTCTAGCTAATACA
GCCTATATACCGCCATCTCCAGCAAACCCTAAAAAGGAAGCACAGTAAGCAAGACTATGAA
AACATAAATACGTTAGGTCAAGGTGTAGCCCATGAATTGGAAAGAAATGGGCTACATTTTCT
AAAATAGAACACAAACGAACGCCCTAATGAAAACGAGGGCCAAAGGAGGATTTAGCAGTA
AGCTGAGAATAGAAAGCTCAACTGAACCCGGCCCTAAAGCACGCACACACCGCCCGTCACC
CTCTTCAAATCCCCAAGAATACCTAAATATATTAGACAAACACCAAGGCATGAGAAGAGAC
AAGTCGTAACAAGGTAAGCATACTGGAAGGTGTGCTTGGATCACCAAAGTGTAGCTTAAAC
AAAGCATCTGGCCTACACCCAGAAGATCTCAATAACATGACCACTTTGAACAAATCCTAGCC
CAACCAACACCCAACATACAACCAAACAGAGCACATAAACCAAAGCATTTACTAGACTAAA
GTATAGGCGATAGAAATTACACAACGGCGCTATAGAAAAAGTACCGCAAGGGAAAGATGA
AAGATGCATTCAAAGTACAAAAAAGCAAAGATTACCCCTTGTACCTTTTGCATAATGGACTA
ACTAGAAACACCCTAGCAAAGAGAACTTAAGCTAGAAACCCCGAAACCAGACGAGCTACCT
ACGAGCAGTTTAAAGAACCCACTCATCTATGTGGCAAAATAGTGAGAAGACTTATAGGTAG
AGGTGAAAAGCCTAACGAGCCTGGTGATAGCTGGTTGTCCAAGAAATGAATCTAAGTTCAA
CTTGAAGCATACCCAAAAGCCCAAAAACTATAATGTAGGCTTCAAGTATAGTCTAAAAAGG
TACAGCTTTTTAGAAACAGAATTAAATCTTAATTAGTGAGTAAACAATACAACAACCATAGT
TGGCCTAAAAGCAGCCATCAATTAAGAAAGCGTCAAAGCTCAACAATAAGACATAAATAAT
ACCACAATAAAAATCAACTCCTAAACCAATATTGGACTAATCTATCAATAAATAGAAGAA
ATACTGTTAGTATGAGTAACAAGAAATAGATCTCCTTGCACAAGCTTATATCAGAACGGATG

ACCCACTGATAATTAACAACAAAACAAATCAAACCCAAAAATAGAACTTTGTTAAATAGAT
TGTTAACCCAACACAGGCGTGCATATTCTCAAAGGAAAGGTTAAAACAAATGAAAGGAACT
CGGCAAACACAAACCCCGCCTGTTTACCAAAAACATCACCTCTAGCATAACCAGTATTAGAG
GCACTGCCTGCCCAGTGACTCGCGTTAAACGGCCGCGGTATTCTGACCGTGCAAAGGTAGCA
TAATCACTTGTTCTCTAAATAAGGACTAGTATGAACGGCTAGACGAGGGTTTTACTGTCTCTC
ATCTGTAACCAGTGAAATTGACCTCCCCGTGAAGAGGCGGGAATAATATAATAAGACGAGA
AGACCCTATGGAGCTTTAATTAAACAGCTTAAAATTAACCCAACACCACTCTAACAGAGATA
TAACAAACCAATTAGCCTAAGCTGTCAATTTTGGTTGGGGTGACCTCGGAGCAAAAAACAAC
CTACGAGCGGTCAAATCCAGACTAACAAGTCCAGATAATCCGTTAATTGATCCAACAACTTG
ATCAACGGAACAAGTTACCCTAGGGATAACAGCGCAATCCTATTCGAGAGTCCATATCGAC
AATAGGGTTTACGACCTCGATGTTGGATCAGGACATCCCAATGGTGCAGAAGCTATTAATGG
TTCGTTTGTTCAACGATTAAAGTCCTACGTGATCTGAGTTCAGACCGGAGTAATCCAGGTCG
GTTTCTATCTATTAATACACTCCTCCCAGTACGAAAGGACAAGAGAAGTAGGGCCTACCTCA
CACAGGCGCCCTCGAATCAATAAATGACTACCCTCTTAATTTAGCCAATTCACAACAACCCC
CAGTCCTAGAACCAGGGCCAGCTAGGGTAGCAAAGCACGGCAACTGCACAAGACTTAAGCT
CTTGTAACAGAGGTTCAACTCCCCTCCCTAGCAGCAATGTACACAATCAACATAATAATAAT
GATCATCCCCATCCTACTAGCCGTGGCATTCCTAACGCTAGTAGAACGCAAAGTGCTAGGAT
ACATGCAACTACGAAAGGGCCCAAACATCGTGGGCCCTTGAGGCCTGCTACAACCAATCGC
CGACGCAGTAAAACTATTCACTAAAGAGCCCCTACGACCCCTAACATCCTCAATCACAATAT
TCATCATGGCACCTATCCTAGCACTGACACTCGCACTCACCATGTGAGTACCACTACCAATG
CCACACCCACTAGTCAACATAAACCTAGGAGTGCTATTCATACTGGCCATATCAAGCCTCGC
CGTATACTCCATCTTATGATCAGGGTGAGCCTCAAACTCCAAATACGCCCTCATTGGAGCAC
TACGTGCAGTGGCCCAAACCATCTCATACGAAGTGACACTAGCAATTATCCTGCTGTCCTTA
CTGTTAATAAGCGGGTCCTTCACCCTCTCCACCCTAATCACAACCCAAGAAAAGCTATGATT
ACTAGTGCCCGCATGACCCCTGGCCATAATATGATTCATCTCGACCCTAGCCGAGACAAATC
GAGCACCCTTCGACCTAACAGAAGGAGAATCTGAGCTCGTATCCGGCTTTAACGTTGAATAC
GCAGCAGGCCCATTCGCACTCTTCTTCCTTGCCGAATACGCAAACATCATCATAATAAACAT
CTTGTCAGTGACGCTCTTCATAGGAGCCTTTCACGACCCCCACACCCCAAGCCTCTACACAG
TTAATTTTGTAGTAAAAACACTCGCCCTTACCGCCCTATTCCTATGAATCCGAGCATCCTACC
CACGATTCCGCTACGACCAACTAATGCACCTACTATGAAAAAACTTCCTACCACTAACCCTA
GCACTGTGCATGTGACACGTATCAATACCAATCGCCCTGTCAAGCATTCCCCCACAATCATA
AGAAATATGTCTGACAAAAGAATTACTTTGATAGAGTAAATAATAGAGGTTTAAATCCCCTT
ATTTCTAGAAAAACAGGCATTGAACCTGCACCTGAGAGCTCAAAAATCTCCGTGCTACCAAC
TTACACCACAATCTACAGTAAGGTCAGCTAAGCAAGCTATCGGGCCCATACCCCGAAAATGT
TGGATCACAGCCTTCCCGTACTAATAAACCTCACTATACTAGCCATCCTAACAACCCTATTCC
TAGGGACCATGCTCGTCCTAATCAGCTCTCACTGGCTAATGATTTGAATTGGATTCGAGATA
AACATACTAGCAATAGTCCCTATCCTAATGAAAAACTTTAGCCCACGGGCCATAGAAGCAGC
CACAAAATACTTCCTAATCCAAGCCACCGCGTCCATACTACTAATGCTAGCCATTACCCTAG
ACCTGATATCTTCTGGACAATGAACCATCACAAAAACACACAACACCCTGCCATCAGCCATC
ATCACCGTATCTATGGCCATAAAACTAGGAATAGCACCATTCCACTTCTGAGTGCCAGAAGT
AACACAAGGAAGCCCACTATCATCAGGCATGCTACTCCTAACCTGACAAAAAGTCGCACCA
ATATCAATCCTATACCAAATAATACCCACAATCAACACAAATATACTAACAACCATAGCCGC
ACTCTCAATCCTCATCGGCGGTTGAGGAGGGCTAAACCAGACCCAACTACGAAAAATCATA
GCATACTCCTCAATCGCCCACATAGGCTGAATAGCAATAATCATGACATACAACCCGGACAT
TGCCATTCTAAACCTACTAGTCTACATCATAATGACACTATCTATATTCGCAATCCTACTATA
CAACTCATCAACAACAACCCTATCACTATCCCACCTATCAAACAAGACACCACTAATCACAG
CCCTCGCACTACTAATCCTACTATCACTAGGGGGCCTCCCCCCACTGACCGGATTCATGCCC
AAATGAATAATCATCCAAGAACTAACCAAAAACAACATAGTAATAATACCAACCGTGATAG
CAATAACAGCACTACTCAACCTATACTTCTACGTACGCCTGGCGTACTCCACAGCACTAACC
ATACTCCCAACTACCAACAACATAAAAATAAAATGACAATTCGAAACAACAAAAATCATAA
AACTAACCCCACCTCTAATCATTTTATCCACAATAGCACTCCCACTCACCCCTATAATATCAA

TCCTAAACTAGGGACTTAGGCTACACAGACCGAGAGCCTTCAAAGCTCTAAGCAAATACAA
ACTATTTAGTCCCTGATATAAAGACTGTAGAAATTCAACCTACATCACCTAAACGCAAATCA
GGCGCTTTAATTAAGCTAAATCCTCACTAGATTGGTGGGATACAAACCCACGAAACTTTAGT
TAACAGCTAAACACCCTAATCAACTGGCTTCAATCTACTTCTCCCGCCATAAAAAAGAAAGT
GGCGGGAGAAGTCCGGGCAGAATTGAAGCTGCTTCTTTGAATTTGCAATTCAATGTGTAAAC
ACCTCAGGACCTGGCAAGAAGAGGACTCCAACCTCTGTGTTCAGATTTACAGTCTAATGCTT
ACTCGGCCATCTTACCTATGTTCACCAACCGCTGGCTCTTTTCAACAAATCACAAAGACATTG
GCACACTATATCTCTTATTTGGCGCCTGGGCCGGAATAGTAGGCACCGCACTAAGTCTTCTA
ATTCGCGCTGAGCTAGGTCAGCCTGGGACCCTCTTAGGGGACGACCAAATTTACAATGTGAT
CGTTACCGCACATGCATTTGTAATAATCTTCTTCATAGTCATGCCAATCATAATCGGGGGCTT
TGGAAACTGACTAGTGCCCCTGATAATTGGAGCCCCTGACATGGCATTCCCCCGTATAAACA
ACATAAGTTTCTGACTGCTCCCCCCCTCTTTCCTACTTCTTCTGGCCTCTTCCATAGTCGAAGC
AGGGGCTGGAACCGGCTGAACTGTATACCCCCCTTTAGCAGGAAACTTAGCACACGCAGGA
GCATCTGTAGACCTAACCATCTTCTCTCTTCATCTAGCGGGTGTCTCATCAATCCTTGGGGCT
ATCAATTTTATTACAACAATCATCAACATAAAACCCCCTGCAATAAACCAATACCAAACCCC
ACTATTCGTGTGATCAGTCCTAATTACGGCCGTGCTTCTGCTACTATCTCTACCCGTACTAGC
CGCTGGCATTACCATACTGTTAACCGACCGTAATCTAAATACAACCTTTTTTGACCCCGCAG
GAGGAGGTGACCCCATTCTATACCAACACCTATTCTGATTCTTCGGACACCCCGAAGTGTAC
ATTCTCATCCTTCCTGGATTTGGAATAATCTCCCACATCGTAACCTATTACTCCGGGAAAAAA
GAGCCCTTTGGATACATGGGCATAGTCTGAGCAATAATATCCATTGGCTTCCTGGGCTTCATT
GTATGGGCACACCACATGTTCACAGTGGGAATAGACGTTGATACACGGGCCTACTTCACATC
AGCCACCATAATTATTGCTATCCCCACTGGAGTAAAGGTGTTTAGTTGACTAGCAACCCTGC
ACGGAGGAAACGTAAAATGGGCCCCAGCTATACTATGGGCCCTGGGCTTTATCTTCCTGTTT
ACAGTCGGGGGTCTAACTGGCATCGTGCTGGCCAACTCGTCCCTAGATATCGTCCTCCATGA
TACCTACTACGTAGTAGCCCACTTCCACTACGTTCTCTCCATGGGAGCAGTTTTCGCCATCAT
AGGAGGCTTCGTCCATTGATTCCCCCTGTTCTCAGGATACACGCTCAACAACACATGGGCAA
AAGTTCACTTTACAATCATATTCGTGGGCGTAAACATGACCTTTTTCCCCCAACACTTCCTTG
GACTGTCTGGAATACCTCGACGATACTCGGACTACCCAGACGCTTACACAATGTGAAACACT
GTGTCCTCCATAGGATCCTTCATCTCATTAACCGCCGTAATACTCATGGCCTTCATAATCTGA
GAGGCATTCGCCTCTAAACGGGAAGTCCTAATAGTGGAGTCCACAAATACCAACCTCGAAT
GACTACACGGCTGCCCACCACCCTACCACACATTCGAAGAACCTGCTTTCGTAAATCTGGTC
AAAACAAGAGAGGAAGGAATCGAACCCTCAAACAATGGTTTCAAGCCAATATCATAACCCC
TATGTCTCTCTCAATCACAGAAGTATTAGTAAAATTTACATAACTTTGTCAAAGTTAAATTAT
AGGTTCAAGCCCTTTATACTTCCATGGCGTACCCGCTCCAATTAGGCTTTCAAGATGCCACAT
CCCCAATCATAGAAGAATTACTCCACTTCCACGATCACACACTAATAATCGTGTTCTTGATC
AGCTCCCTAGTACTATACATTATCTCTCTCATACTAACAACCAAACTCACCCACACAAGTAC
AATAGACGCTCAAGAAGTAGAAACCATTTGAACCATCCTCCCCGCAATTATCCTAATCCTAA
TCGCCCTGCCCTCCCTACGCATCCTCTACATAATGGACGAGATTAACAACCCCGCCCTGACA
GTAAAAACAATGGGCCATCAATGATACTGAAGCTACGAATACACAGACTACGAAGACCTAA
GCTTCGACTCATACATAATCCCAACTCAAGACCTAAAACCCGGCGAACTCCGACTTCTAGAA
GTGGACAACCGACTTGTACTACCCATAGACACAACCATCCGCATGCTCATCTCATCTGAAGA
CGTCCTGCACTCTTGAGCCATCCCATCCCTGGGCCTAAAAACAGATGCTATCCCGGGGCGTC
TAAACCAAACAACCCTGATATCAACCCGGCCCGGCCTATTCTACGGACAGTGCTCAGAAATC
TGTGGCTCAAATCACAGCTTCATGCCAATTGTCCTCGAACTAGTACCCCTAAAAACATTTGA
AAACTGAACTACGTCCCTACTGTAATTCATTGAGAAGCTAATAGCGCTAGCCTTTTAAGTTA
GAGATTGAGAGCACGCCTCTCCTCAATGACATGCCTCAACTAGACACTACAACATGATCCAT
TACAATTATATCCATGATTCTGACCCTCTTTGTCCTATTCCAATTAAAAATCTCTAAACACTA
CTACCCGCACGACGCAGAGACCTCAACAAAAACACACCACAAAACACTCACCCCCTGAGAA
AAAAAATGAACGAAAATCTATTCACCTCTTTCATTACCCCAGTAATAATAGGGATCCCTATT
GTAACAATTATCATTATGTTCCCAGTAATCCTCTTCCCAACATCAAACCGACTAATCAACAA
CCGCATTGTATCCATACAACAATGACTCCTAAAACAAACATCCAAACAAATAATAAGCATCC

ACAACTACAAAGGACAGACCTGAACCCTGATATTAATAACACTAATCATTTTCATCGCATCT
ACTAACCTACTAGGCCTGCTACCCCACTCATTCACCCCCACAGCCCAACTGTCAATAAACCT
GAGCATAGCCGTTCCTCTATGGGCAGCCACCGTAGTCACAGGTTTTCGACACAATACAAAAA
CATCTTTAGCCCACTTCCTACCCCAGGGAACACCAACCCCCCTTATCCCAGTGCTAGTGATCA
TTGAAACAATCAGCCTGCTAATCCAACCCATAGCGCTCGCAGTACGACTGACAGCCAACATC
ACCGCCGGCCACCTGTTAATACACCTAATCGGAAGCGCAACCCTTGCCCTAATATCAATCAA
CCTCACCGTGGCCACAACTACCTTCATCGTCCTAGTCCTGCTTACAATCCTTGAATTCGCAGT
CGCACTCATTCAGGCCTACGTATTCACTCTTCTAATCAGCCTCTACTTACATGATAACACATA
ATGACCCACCAAACCCACTCATACCACATAGTAAACCCAAGCCCTTGACCCCTAACCGGGGC
CCTATCTGCCCTCCTAATAACATCGGGCCTAGCAATATGATTCCACTTCAACTCTACAATACT
ACTCCTCCTAGGACTAACAACGAACCTCTTAACAATATATCAATGATGGCGTGACATTGTAC
GAGAAAGCACCTTTCAAGGCCACCACACACCCACAGTCCAAAAAGGACTACGATACGGCAT
AATCCTCTTCATTGTCTCAGAAGTATTTTTCTTCGCTGGCTTCTTCTGAGCATTCTACCACTCA
AGCCTAGCACCTGCCCCCGAACTAGGAGGATGCTGACCCCCCACAGGCATCAACCCGCTAA
ACCCACTGGAAGTACCTCTACTCAACACATCCGTTCTTTTAGCCTCAGGAGTATCAATTACAT
GGGCACACCATAGCTTAATAGAAGGCAGCCGAAACCACATAACCCAGGCCCTGCTCATCAC
AATCCTCCTAGGCATTTACTTCACACTACTGCAGGTCTCAGAATATTACGAAGCACCCTTCAC
AATCTCCGACGGCGTGTACGGCTCTACCTTCTTTGTGGCAACTGGATTCCACGGCCTACACGT
CATCATCGGCACCTCCTTCCTAACTGTGTGCCTACTACGACAACTAAAATACCACTTTACATC
AAACCACCACTTCGGATTCGAAGCTGCCGCCTGATACTGACACTTCGTAGATGTAGTGTGAC
TGTTCCTGTATGTCTCCATTTACTGATGAGGTTCCTATTTTCTAAGTATGCACAGTACAGTTG
ACTTCCAATCAACAAGCTCTGGCCACAACCCAGAAGAGAATAATAAACCTACTCCTAGCAAT
AACAACAAACACCATACTAGCATGCCTGCTCATACTAATCGCCTTCTGACTCCCTCAATTAA
ACACGTACTCGGAAAAAATCACCCCCTACGAATGCGGATTCGACCCCATGGGATCGGCACG
ACTACCATTCTCCATAAAATTCTTCCTAATCGCCATCACGTTCCTACTATTCGACCTAGAAAT
CGCACTACTCCTGCCACTCCCCTGAGCATCACAAACAAACAACCTAGGCACCATAATCACCG
TAGCCTTGGCCCTCATCCTACTGCTAGCAATTAGCCTGACCTACGAATGAACACAAAAAGGC
CTAGAATGAACTGAGTATGGTAGCTAGTTTACACAAAACAAATGATTTCGACTCATTAAACT
ATGACTTACTCATAGCTACCAAATGTCCCTAATTTACATTAACACAACACTGGCATTCACTAT
CTCCCTAATGGGGATATTAATGTACCGATCACACCTAATGTCCTCACTACTATGTCTAGAGG
GCATAATACTCTCCCTATTCGTAATAATCACCATTACAATCCTAACTAACCACTTCACACTAG
CCAACATAGCACCCATCATCCTCCTCGTACTAGCAGCATGCGAGGCTGCCCTAGGCCTCTCT
CTACTAGTACTTGTATCTAACACATACGGCACGGACTACGTACAAAACCTAAACCTGCTACA
ATGCTAAAACTAATTGTCCCCACAGCCATACTAATTCCCCTAACATGACTATCGAACAAAAA
CATAGTGTGAATCAACGTAACATCACACAGCATGCTAATCAGCCTAACATCTCTAGGCATCT
TGGGCCAGCATGACCACAACAACATAAGCCTCTCAGCCATCTTCTTCTCTGACCCCCTATCA
GCACCCTTAATCGTATTAACAACATGACTCCTCCCCCTAATACTAACAGCCAGCCAAGCCCA
CCTCTCCAATGAGCCCCTGGGCCTAAAAAAACTATACATTACCATACTAATCACCCTCCAAG
CACTACTAATCATAACATTCGCCTCCTCTGAGTTCATTATATTCTACATCCTATTTGAAGCAA
CACTCGTACCAACCCTAATCATCATCACACGATGAGGAAACCAAGCAGAGCGACTAAACGC
AGGGTCCTACTTCCTCTTCTACACAATAGTAGGGTCACTCCCCCTTCTAGTAGTACTCACATA
CACCCAAAACATAACAGGAACCCTAAACATACTAGTACTACAATACTGAGCGAAACCCATA
AACGACTCTTGATCCAACATGCTAATATGACTAGCATGCATAATAGCATTCATAGTAAAAAT
ACCCCTATACGGACTACACCTCTGACTGCCAAAAGCCCACGTAGAGGCCCCCATTGCAGGCT
CCATAGTACTTGCAGCCGTACTACTAAAACTAGGCGGATACGGCATAATACGCATCACTATC
ATGCTAGAACCAATAACAACATTCATAGCATACCCCTTCCTAATACTATCCCTGTGAGGAAT
AATCATAACAAGCTCCATCTGCCTTCGCCAAACCGACCTAAAATCACTAATCGCCTACTCCT
CCGTAAGCCACATAGCACTGGTAATCGTCGCAATCCTAATCCAAACCCCATGAAGCTACATA
GGGGCCACCGCCCTCATAATCGCCCACGGTCTAACATCCTCCATACTATTTGCCTAGCAAA
CACAAACTATGAGCGAACCCACAGCCGAACTATAATACTAGCACGAGGACTGCAAACCCTC
CTACCCCTAATAGCTGCCTGATGACTCCTAGCAAGCCTAACCAACCTGGCCCTCCCCCCCAG

CATCAACCTAATCGGAGAACTATTCGTAGTAGTATCAACATTCTCGTGGTCTAACACCACCA
TCATCCTTACTGGAACAAACATTATCATCACAGCCACCTACTCCCTATACATACTAATATCCA
CCCAACGTGGAAAATACACCCACCACATCAACAACATCTGCCCCTCCTTCACCCGAGAAAAC
GCTCTAATGGCACTCCACATACTGCCCCTACTAATGCTATCAACCAACCCCAAAATCATCCT
AGGGTGCCTTTACTGTAAGTATAGTTTAATAAAACCTCAGATTGTGGATCTGACAATAGAAG
ACCACAACTTCTTACTTACCAAAAAAGCATGCAAGAACTGCTAATTCCTGCCCCCATGTATA
AAAACATGGCTTTTTTAAACTTTTAGAGGATGACAGACACCCGTTGGTCTTAGGAACCAAAA
GACTTGGTGCAACTCCAAGTAAAAGTAATTAACACCCTAACCTGCACCTCCCTTATAACACT
AACAACACTAATCATACCAATCATAATCACCCCCACAAATGCCTACACAAGCAAAAACTAC
CCCCACCACGTAAAAAACATAGTCGCACTAGCCTTCGCCGTCAGCATAATCCCAGCAATAGC
ATTTATCTACTCAAACGAAGAAATAACCATCTTCAACTGACACTGAACAACAATCCAAACAA
TAAAAATAACAATAAGCTTCAAACTAGACTATTTCTCCACAACATTCATACCAGTGGCCCTA
TTCGTCACATGGTCTATCATAGAATTCTCTCTATGATATATAAAATCAGACCCCCACATCAAC
CGATTCTTCAAGTACCTACTAATCTTCCTAATCACAATAATAATCCTAGTATCCGCCAACAAC
ATATTCCAACTCTTCATCGGCTGAGAAGGAGTAGGAATCATATCTTTCCTCCTAATCGGCTG
ATGGCACGGACGAACAGATGCAAACACAGCAGCCATACAAGCCATCCTATACAACCGCATC
GGAGACATTGGACTAATCCTGTCAATGGCATGATTCCTCACAAACCTAAACTCATGGGACCT
CCAACAAATCCTCATGCTAAACCCCGAGAACACAAACATCCCCCTAGCGGGCCTACTACTAG
CAGCAACTGGAAAATCCGCACAATTTGGGCTACACCCATGACTCCCCTCAGCCATAGAAGGC
CCAACCCCAGTATCAGCCCTACTCCACTCCAGCACAATAGTAGTCGCAGGGGTATTCCTACT
AATCCGATTCCACCCCCTAATAGAAAACAACAAAACAATCCAAACAACAACCCTATGCCTA
GGGGCCATTACAACCCTGTTCACAGCCATCTGTGCTCTCACCCAAAACGACATCAAAAAGAT
CGTGGCCTTCTCAACCTCAAGCCAACTAGGTCTAATAATAGTCACCATCGGAATCAACCAAC
CACACCTAGCCTTCATACACATCTGCACCCACGCCTTCTTCAAAGCCATACTATTCATGTGCT
CCGGATCGATCATCCACAGCCTAAACGACGAACAAGACATCCGCAAAATAGGAGGACTTTT
CCACGCCCTCCCCACTACCACCTCCGCTCTCATTATTGGCAGCCTAGCCCTAACAGGAACCC
CATTCCTAACAGGCTTCTACTCTAAAGACCTAATCATCGAAACCGCTAACATATCCCACACA
AACGCCTGAGCCCTGCTCATCACACTAGTAGCCACCTCCCTCACAGCCGCCTACAGCACACG
AATCATCTTCTTCACACTATTAGGACAACCCCGATCCAACACCCTAATCAACATCAACGAAA
ACAACCCCTCACTAACAAACCCAATCAAACGCCTAATGCTAGGGAGCATCTTCGCCGGATTC
CTACTATACAACAACATCCCCCCAACAACCATGCCCCAAACAACCATACCACAATACCTAAA
ATACACCGCCCTCACCACCACAATCCTAGGCCTAATCATAGCCCTAGAACTGTGCAACCTGT
CCCTAAACCTAAGCCACAAACCCCCCTCAAGTGCCTTCAAATTCTCAACCTTACTAGGCTAC
TTCCCCACAATCATTCACCGCTCAGGACCCCTCTGATCACTAACAACAAGCCAAAAACTAGC
ATCCCTTATTCTAGACCTAATCTGACTAGAAAATATTATACCAAAGTCAATCTCACACTTCCA
CATAAAAGCCTCCTTAATAACATCCAACCAAAAAGGCTCAATCAAACTATACTTCCTGTCCT
TCGCAGTAACAATAATCCTCGCTCTCCTCATATTCTATTCCCCCGAGTAACCTCCATAACAAC
AACAACACAAATAAACAAAGACCACCCGGTCATAATCACAAACCAAGCACCATGACTATAA
AGAGCAGCAACACCCGCAGCTTCCTCACTAAAAAAACTAGACCCCCCAACATCATAAGTCG
CCCAATCACCAGGACCACCAAGATCAAAAACCACACTCAACCCCTCACCCTTCAACATATAA
AGCACCAAAACCAACTCTATGACCACACCCAAAACAAAAGCACCGAGCACAACCACATTAG
ACACCCAAACTTCAGGATACTCCTCAATGGCTATGGCCGCCGTATAACCAAAAACAACCAAC
ATCCCCCCAAGATAAATCAAAAAAACCATTAAACCCAAAAAAGACCCACCAAAATTAACTA
CAACCCCACAACCTGCACCGCCAGCTACAACCAGACTAAACCCCCCATAAATAGGAGAAGG
CTTTGAGGAAAACCCTAAAAAACTAATCACAAAAATAACACTCAAAATAAACACAACATAT
GTCATTATTCCCACATGGACTCAAACCATGACCTATGACACGAAAAATCATCGTTGTAATTA
AACTACGGAAACCTAATGACAAACATCCGAAAAACACACCCCCTACTCAAAATCATCAACA
ACTCCTTTATTGATCTCCCCACCCCCTCTAACATCTCAGCATGATGAAACTTCGGATCACTAC
TAGGAATTTGCTTAGTCCTACAAATTCTCACAGGACTATTTCTAGCCATACACTACACAGCA
GACACAGCAACCGCATTCTCATCAGTAACTCACATCTGCCGAGATGTAAACTACGGATGAAT
TATCCGATACATACACGCTAACGGAGCTTCCCTGTTCTTCATCTGCCTATTCGCACACATCGG

ACGAGGCATCTACTACGGATCCTTTGCCTACAAAGAGACATGAAACATCGGTATCCTGCTCC
TGTTTGCAGTAATAGCAACAGCCTTTATGGGATACGTCCTACCATGAGGACAAATGTCCTTC
TGAGGTGCTACAGTAATTACAAACCTTTTATCCGCAATACCCTACATCGGGTCTAGCCTAGT
AGAGTGAGTCTGAGGGGGATTCTCGGTAGACAAAGCAACTCTCACTCGATTCTTCGCTCTTC
ACTTCATCCTTCCCTTCGTAATTCTTGCCCTAGTACTAGTACACTTACTATTCTTACACGAAA
CCGGATCCAACAACCCAATAGGAATCGTATCCAACCCCGACGTAATCCCCTTCCACCCATAC
TACACAGCCAAGGACACCCTTGGCCTATTCATCATGCTCACAGCACTAATATCCTTAGCCCT
ATTCTTCCCCGACCTACTAGGAGACCCAGACAATTATACACCCGCAAACCCCCTAAACACAC
CACCCCACATCAAACCAGAATGGTACTTCCTATTCGCATACGCAATCCTACGCTCAATTCCC
AACAAATTGGGAGGAGTACTAGCACTAATCCTATCCATCCTCGTACTAGCACTAATCCCACT
ACTACACACATCAAAACAACGAACTATAATGTTCCGACCCCTGAGCCAAACCATCTTCTGAC
TCCTGGTGGCCGACCTACTAGTTCTCACATGAATCGGAGGACAACCCGTGGAACACCCCTTC
ATCCTAATCGGACAAGTGGCCTCTATCCTTTATTTCACACTAATCCTAGCAGCAATGCCAATC
GCAGGTATTATCGAAAACAATCTCATAAAATGAAGAGTCTTTGTAGTATATGTCAATACACT
GGTCTTGTAAACCAGCAAAGGAATTAACCCTCCCCAAGACTCAGGAAGAAGACAAAAGCCC
TACCATCAGCACCCAAAGCTGAAATTCTCAATAAACTACTTCCTGCAAACAATTAAAGTCCA
ACAAGCTTTATATAGTATATGCCATGTATAATCGTGCATTAATGGTTTGCCCCATGCATATAA
GCAGGTACATTATATTATTATAGTACATAGGACATATTATGTATAATCGTGCATTATTGATCT
AGTACATACATATAAGCAGGTACATTATATTATTATAGTACATAGGACATATTATGTATAAT
CGTGCATTATTGATCTAGTACATGCATATAAGCAGGTACATAATATCCTTAGAGTACATAGT
ACATATTATTATTGATCGGACATAGCACATCAAGTCAAATCATTTCCAGTCAACATGCGTAT
CCCTACCACTGAAGGCCGCCTAATCACCATGCCGCGTGAAATCATCAACCCGCTCATAGTCG
TGTCCCTCTTCTCGCTCCGGGCCCATATGGACTGTGGGGTAGTTAAAGTGAAACTATACCTG
GCATCTGGTTCTTACTTCATGTTCATCTTATATCTAGCCGCTCACTCGTTCCTCTTAAATAAGA
CATCTCGATGGATTAATTACTAATCAGCCCATGCCTAACATAACTGTGCTGTCATGCCTTTGG
TATCTTTTAATTTTCGGGGTGCGAGGTTCAACTGGGTCACCGACCTTCGAGCAGGTGGATAA
CTTGTAGATAGACATTCATTGAATATTATTGGTCGTACATACTAACTCCTAGGTGTTATTCAG
TCAATGGTTACAGGACATAGAGAATTTTACAACAAAATTTTGAGTTGAGCCTACAGAAATCA
TAGATCACAATCGCAGACACCAACAAATACTAAAGTAATTAATTTAAATTTATAATTTTAGG
TTTGATATATCAAACCCCCCCTTACCCCCCCAAAGCCTCCACGTACTAAACATCTTGTCAAAC
CCCAAAAGCAAGAATACACACGTACACAAAGGCGCTATAAGCACAAAGTATTACGTAAGTA
TATAGATATACAAAACTACTAGCGCAGCTTTCTGTCCACTAGTTAAATACTTAGGTGCTTGA
ACATATATGGCTGGAAGGCCATGTAGTGGCTACGTAATGATTTCTATTAGCCACAATAAAAA
CAATATACAATAAATACAAATATAGATCAATTCTTATAGGCGCCGATAGCATAAACTATCTG
CCCCCTATGTACAATTGAATTAAA
>SRR7498027 (circular) REORGANIZED
GTTAATGTAGCTTAAAACCAAAGCAAAGCATTGAAAATGCTTAGATGAGTCCACAAACTCC
ATAAACACACAGGTTTGGTCCCAGCCTTTTTATTAATTTTCAATAGGATTACACATGCAAGTA
TCCGCCCCCCAGTGAAGATGCCCTTCAAGTTATCACAGTACCTGAAGGAGCTGGCATCAAGC
ACGCCAATAAACGCAGCTAACGACGCCTTGCAGAGCCACACCCCCACGGGAAACAGCAGTG
ATAAAAATTAGGCTATAAACGAAAGTTCGACCTAGCCATATTGCATTGGGTTGGTAAATCTC
GTGCCAGCCACCGCGGTCATACGATTAACCCTAGCTAATAAAAAACCGGCGTAAAGCGTGC
TAAGATAAATCCAATCCAAATAAAGTTAAGCCCTGACCAGGCCGTAAAAAGCCGTGGTTAC
CGTAAAAATAAACTACGAAAGTAACTTTAATTCACATCGACACACGATAGCTAAGACCCAA
ACTGGGATTAGATACCCCACTATGCTTAGCCCTAAACCTAAATAATTCACCAGACAAAATTA
TTCGCCAGAGAACTACTGGCAACAGCCTAAAACTCAAAGGACTTGGCGGTGCTTTACATCCC
CCTAGAGGAGCCTGTTCTGTAATCGATAAACCCCGATAAACCCTACCAATTCTAGCTAATAC
AGCCTATATACCGCCATCTCCAGCAAACCCTAAAAAGGAAGCACAGTAAGCAAGACTATGA
AAACATAAATACGTTAGGTCAAGGTGTAGCCCATGAATTGGGAAGAAATGGGCTACATTTTC
TAAAATAGAACACAAACGAACGCCCTAATGAAAACGAGGGCCAAAGGAGGATTTAGCAGT
AAGCTGAGAATAGAAAGCTCAACTGAACCCGGCCCTAAAGCACGCACACACCGCCCGTCAC

CCTCTTCAAATCCCCAAGAATACCTAAACATATTAGACAAACACCAAGGCATGAGAAGAGA
CAAGTCGTAACAAGGTAAGCATACTGGAAGGTGTGCTTGGATCACCAAAGTGTAGCTTAAA
CAAAGCATCTGGCCTACACCCAGAAGATCTCAATAACATGACCACTTTGAACAAATCCTAGC
CCAACCAACACCCAACATACAACCAAACAGAGCACATAAACCAAAGCATTTACTAGACTAA
AGTATAGGCGATAGAAATTACACAACGGCGCTATAGAAAAAGTACCGCAAGGGAAAGATG
AAAGATGCATTCAAAGTACAAAAAAGCAAAGATTACCCCTTGTACCTTTTGCATAATGGACT
AACTAGAAACACCCTAGCAAAGAGAACTTAAGCTAGAAACCCCGAAACCAGACGAGCTACC
TACGAGCAGTTTAAAGAACCCACTCATCTATGTGGCAAAATAGTGAGAAGACTTGTAGGTA
GAGGTGAAAAGCCTAACGAGCCTGGTGATAGCTGGTTGTCCAAGAAATGAATCTAAGTTCA
ACTTGAAGCATACCCAAAAGCCCAAAAACTATAATGTAGGCTTCAAGTATAGTCTAAAAAG
GTACAGCTTTTTAGAAACAGAATTAAATCTTAATTAGTGAGTAAACAATACAACAACCATAG
TTGGCCTAAAAGCAGCCATCAATTAAGAAAGCGTCAAAGCTCAACAATAAGACATAAATAA
TACCACAAATAAAAATCAACTCCTAAACCAATATTGGACCAATCTATCAATAAATAGAAGA
AATACTGTTAGTATGAGTAACAAGAAATAGATCTCCTTGCACAAGCTTATATCAGAACGGAT
GACCCACTGATAATTAACAACGAAACAAATCAAACCCAAAAATAGAACTTTGTTAAATAGA
TTGTTAACCCAACACAGGCGTGCATATTCTCAAAGGAAAGGTTAAAACAAATGAAAGGAAC
TCGGCAAACACAAGCCCCGCCTGTTTACCAAAAACATCACCTCTAGCATAACCAGTATTAGA
GGCACTGCCTGCCCAGTGACTCGCGTTAAACGGCCGCGGTATTCTGACCGTGCAAAGGTAGC
ATAATCACTTGTTCTCTAAATAAGGACTAGTATGAACGGCTAGACGAGGGTTTTACTGTCTC
TCATCTGTAACCAGTGAAATTGACCTCCCCGTGAAGAGGCGGGAATAATATAATAAGACGA
GAAGACCCTATGGAGCTTTAATTAAACAGCTTAAAATTAACCCAACACCACTCTAACAGAGA
TATAACAAACCAATTAGCCTAAGCTGTCAATTTTGGTTGGGGTGACCTCGGAGCAAAAAACA
ACCTACGAGCGGTCAAATCCAGACTAACAAGTCCAGATAATCCGTTAATTGATCCAACAACT
TGATCAACGGAACAAGTTACCCTAGGGATAACAGCGCAATCCTATTCGAGAGTCCATATCGA
CAATAGGGTTTACGACCTCGATGTTGGATCAGGACATCCCAATGGTGCAGAAGCTATTAATG
GTTCGTTTGTTCAACGATTAAAGTCCTACGTGATCTGAGTTCAGACCGGAGTAATCCAGGTC
GGTTTCTATCTATTAATACACTTCTCCCAGTACGAAAGGACAAGAGAAGTAGGGCCTACCTC
ACACAGGCGCCCTCGAATCAATAAATGACTACCCTCTTAATTTAGCCAATTCACAACAACCC
CCAGTCCTAGAACCAGGGCCAGCTAGGGTAGCAAAGCACGGCAACTGCACAAGACTTAAGC
TCTTGTAACAGAGGTTCAACTCCCCTCCCTAGCAGCAATGTACACAATCAACATAATAATAA
TGATCATCCCCATCCTACTAGCCGTGGCATTCCTAACGCTAGTAGAACGCAAAGTGCTAGGA
TACATGCAACTACGAAAGGGCCCAAACATCGTGGGCCCTTGAGGCCTGCTACAACCAATCG
CCGACGCAGTAAAACTATTCACTAAAGAACCCCTACGACCCCTAACATCCTCAATCACAATA
TTCATCATGGCACCTATCCTAGCACTGACACTCGCACTCACCATGTGAGTACCACTACCAAT
GCCACACCCACTAGTCAACATAAACCTAGGAGTGCTATTCATACTGGCCATATCAAGCCTCG
CCGTATACTCCATCTTATGATCAGGATGAGCCTCAAACTCCAAATACGCCCTCATTGGAGCA
CTACGTGCAGTGGCCCAAACCATCTCATACGAAGTGACACTAGCAATTATCCTGCTGTCCTT
ACTGTTAATAAGCGGGTCCTTCACCCTCTCCACCCTAATCACAACCCAAGAAAAGCTATGAT
TACTAGTGCCCGCATGACCCCTGGCCATAATATGATTCATCTCGACCCTAGCCGAGACAAAT
CGAGCACCCTTCGACCTAACAGAAGGAGAATCTGAACTCGTATCCGGCTTTAACGTTGAATA
CGCAGCAGGCCCATTCGCACTCTTCTTCCTTGCCGAATACGCAAACATCATCATAATAAACA
TCTTGTCAGTGACACTCTTCATAGGAGCCTTTCACGACCCCCACACCCCAAGCCTCTACACA
GTTAATTTTGTAGTAAAAACACTCGCCCTTACCGCCCTATTCCTATGAATCCGAGCATCCTAC
CCACGATTCCGCTACGACCAACTAATGCACCTACTATGAAAAAACTTCCTACCACTAACCCT
AGCACTGTGCATGTGACACGTATCAATACCAATCGCCCTGTCAAGCATTCCCCCACAATCAT
AAGAAATATGTCTGACAAAAGAATTACTTTGATAGAGTAAATAATAGAGGTTTAAATCCCCT
TATTTCTAGAAAAACAGGCATTGAACCTGCACCTGAGAACTCAAAAATCTCCGTGCTACCAA
CTTACACCACAATCTACAGTAAGGTCAGCTAAGCAAGCTATCGGGCCCATACCCCGAAAATG
TTGGATCACAACCTTCCCGTACTAATAAACCTCACTATACTAGCCATCCTAACAACCCTATTC
CTAGGGACCATGCTCGTCCTAATCAGCTCTCACTGACTAATGATTTGAATTGGATTCGAGAT
AAACATACTAGCAATAGTCCCTATCCTAATGAAAAACTTTAGCCCACGAGCCATAGAAGCA

GCCACAAAATACTTCCTAATCCAAGCCACCGCGTCCATACTACTAATGCTAGCCATTACCCT
AGACCTGATATCTTCTGGACAATGAACCATCACAAAAACACACAACACCCTACCATCAGCCA
TCATCACCGTAGCTATGGCCATAAAACTAGGAATAGCACCATTCCACTTCTGAGTGCCAGAA
GTAACACAAGGAAGCCCACTATCATCAGGCATGCTACTCCTAACCTGACAAAAAGTCGCAC
CAATATCAATCCTATACCAAATAATACCCACAATCAACACAAATATACTGACAACCATAGCC
GCACTCTCAATCCTCATCGGCGGTTGAGGAGGGCTAAACCAGACCCAACTACGAAAAATCA
TAGCATACTCCTCAATCGCCCACATAGGCTGAATAGCAATAATCATAACATACAACCCGGAC
ATTGCCATTCTAAACCTACTAGTCTACATCATAATGACACTATCTATATTCGCAATCCTACTA
TACAACTCATCAACAACAACCCTATCACTATCCCACCTATCAAACAAGACACCACTAATCAC
AGCCCTCGCACTACTAATCCTACTATCACTAGGAGGCCTCCCCCCACTGACCGGATTCATGC
CCAAATGAATAATCATCCAAGAACTAACCAAAAACAACATAGTAATAATACCAACCGTGAT
AGCAATAACAGCACTACTCAACCTATACTTCTACGTACGCCTGGCGTACTCCACAGCACTAA
CCATACTCCCAACCACCAACAACATAAAAATAAAATGACAATTCGAAACAACAAAAATCAT
AAAACTAACCCCACCTCTAATCATTTTATCCACAATAGCACTCCCACTCACCCCTATAATATC
AATCCTAAACTAGGGACTTAGGCTACACAGACCGAGAGCCTTCAAAGCTCTAAGCAAATAC
AAACTATTTAGTCCCTGATATAAAGACTGTAGAAATTCAACCTACATCACCTAAACGCAAAT
CAGGCGCTTTAATTAAGCTAAATCCTCACTAGATTGGTGGGATACAAACCCACGAAACTTTA
GTTAACAGCTAAACACCCTAATCAACTGGCTTCAATCTACTTCTCCCGCCATAAAGAAGAAA
GTGGCGGGAGAAGTCCGGGCAGAATTGAAGCTGCTTCTTTGAATTTGCAATTCAATGTGTAA
ACACCTCAGGACCTGGCAAGAAGAGGACTCCAACCTCTGTGTTCAGATTTACAGTCTAATGC
TTACTCGGCCATCTTACCTATGTTCACCAACCGCTGGCTCTTTTCAACAAATCACAAAGACAT
TGGCACACTATATCTCTTATTTGGCGCCTGGGCCGGAATAGTAGGCACCGCACTAAGTCTTC
TAATTCGCGCTGAGCTAGGTCAGCCTGGGACCCTCTTAGGGGACGACCAAATTTACAATGTG
ATCGTTACCGCACATGCATTTGTAATAATCTTCTTCATAGTCATGCCAATCATAATCGGAGGC
TTTGGAAACTGACTAGTACCCCTGATAATTGGAGCCCCTGACATGGCATTCCCCCGTATAAA
CAACATAAGTTTCTGACTGCTCCCCCCCTCTTTCCTACTTCTTCTGGCCTCTTCCATAGTCGAA
GCAGGGGCTGGAACCGGCTGAACTGTATACCCCCCTTTAGCAGGAAATTTAGCACACGCAG
GAGCATCTGTAGACCTAACCATCTTCTCTCTTCATCTAGCGGGTGTCTCATCAATCCTTGGGG
CTATCAATTTTATTACAACAATCATCAACATAAAACCCCCTGCAATAAACCAATACCAAACC
CCACTATTCGTGTGATCAGTCCTAATTACGGCCGTGCTTCTGCTACTATCTCTACCCGTACTA
GCTGCTGGCATTACCATACTGTTAACCGACCGTAATCTAAATACAACCTTTTTTGACCCCGCA
GGAGGAGGGGACCCCATTCTATACCAACACCTATTCTGATTCTTCGGACACCCCGAAGTGTA
CATTCTCATCCTTCCTGGATTTGGAATAATCTCCCACATCGTAACCTATTACTCCGGGAAAAA
AGAGCCCTTTGGATACATGGGCATAGTCTGAGCAATAATATCCATTGGCTTCCTGGGCTTCA
TTGTATGGGCACACCACATGTTCACAGTGGGAATAGACGTTGATACACGGGCCTACTTCACA
TCAGCCACCATAATTATTGCTATCCCCACTGGAGTAAAGGTGTTTAGTTGACTAGCAACCCT
GCACGGAGGAAACGTAAAATGGGCCCCAGCTATACTATGGGCCCTGGGCTTTATCTTCCTGT
TTACAGTCGGGGGTCTAACTGGCATCGTGCTGGCCAACTCGTCCCTAGATATCGTCCTCCAT
GATACCTACTACGTAGTAGCCCACTTCCACTACGTTCTCTCCATGGGAGCAGTTTTCGCCATC
ATAGGAGGCTTCGTCCATTGATTCCCCCTGTTCTCAGGATACACGCTCAACAACACATGGGC
AAAAGTTCACTTTACAATCATATTCGTGGGCGTAAACATGACCTTTTTCCCCCAACACTTCCT
TGGGCTGTCTGGAATACCTCGACGATACTCGGACTACCCAGACGCTTACACAATGTGAAACA
CTGTGTCCTCCATAGGATCCTTCATCTCATTAACCGCCGTAATACTCATGGCCTTCATAATCT
GAGAGGCATTCGCCTCTAAACGGGAAGTCCTAATAGTGGAGTCTACAAATACCAACCTCGA
ATGACTACACGGCTGCCCACCACCCTACCACACATTCGAAGAACCTGCTTTCGTAAATCTGG
TCAAAACAAGAGAGGAAGGAATCGAACCCTCAAACAATGGTTTCAAGCCAATATCATAGCC
CCTATGTCTCTCTCAATCACAGAAGTATTAGTAAAATTTACATAACTTTGTCAAAGTTAAATT
ATAGGTTCAAGCCCTTTATACTTCCATGGCGTACCCGCTCCAATTAGGCTTTCAAGATGCCAC
GTCCCCAATCATAGAAGAATTACTCCACTTCCACGATCACACACTAATAATCGTGTTCTTGAT
CAGCTCCCTAGTACTATACATTATCTCTCTCATACTAACAACCAAACTCACCCACACAAGTA
CAATAGACGCTCAAGAAGTAGAAACCATTTGAACCATCCTCCCCGCAGTTATCCTAATCCTA

ATCGCCCTGCCCTCCCTACGCATCCTCTACATAATGGACGAGATTAACAACCCCGCCCTGAC
AGTAAAAACAATGGGCCATCAATGATACTGAAGCTACGAATACACAGACTACGAAGACCTA
AGCTTCGACTCATACATAATCCCAACTCAAGACCTAAAACCCGGCGAACTCCGACTTCTAGA
AGTGGACAACCGACTTGTACTACCCATAGACACAACCATCCGCATGCTCATCTCATCTGAAG
ACGTCCTGCACTCTTGAGCCGTCCCATCCCTGGGCCTAAAAACAGATGCTATCCCGGGGCGT
CTAAACCAAACAACCCTGATATCAACCCGGCCCGGCCTATTCTACGGACAGTGCTCAGAAAT
CTGTGGCTCAAATCACAGCTTCATGCCAATTGTCCTCGAACTAGTACCCCTAAAAACATTTG
AAAACTGAACTACGTCCCTACTGTAATTCATTGAGAAGCTAATAGCGCTAGCCTTTTAAGTT
AGAGATTGAGAGCACGCCTCTCCTCAATGACATGCCTCAACTAGACACTACAACATGATCCA
TTACAATTATATCCATGATCCTGACCCTCTTTGTCCTATTCCAATTAAAAATCTCTAAACACT
ACTACCCACACGACGCAGAGACCTCAACAAAAACACACCACAAAACACTCACCCCCTGAGA
AAAAAAATGAACGAAAATCTATTCACCTCTTTCATTACCCCAGTGATAATAGGGATCCCTAT
TGTAACAATTATCATTATGTTCCCAGTAATCCTCTTCCCAACATCAAACCGACTAATCAACAA
CCGCATTGTATCCATACAACAATGACTCCTAAAACAAACATCCAAACAAATAATAAGCATCC
ACAACTACAAAGGACAGACCTGAACCCTGATATTAATAACACTAATCATTTTCATCGCATCT
ACTAACCTACTAGGCCTGCTACCCCACTCATTCACCCCCACAGCCCAACTGTCAATAAACCT
GAGCATAGCCGTTCCTCTATGGGCAGCCACCGTAGTCACAGGTTTTCGACACAATACAAAAA
CATCTTTAGCCCACTTCCTACCCCAGGGAACACCAACCCCCCTTATCCCAGTGCTAGTGATCA
TTGAAACAATCAGCCTGCTAATCCAACCCATAGCGCTCGCAGTACGACTGACAGCCAACATC
ACCGCCGGCCACCTGTTAATACACCTAATCGGAAGCGCAACCCTTGCCCTAATATCAATCAA
CCTCACCGTGGCCACAACTACCTTCATCGTCCTAGTCCTGCTTACAATCCTTGAATTCGCAGT
CGCACTCATTCAGGCCTACGTCTTCACCCTTCTAATCAGCCTCTACTTACATGATAACACATA
ATGACCCACCAAACCCACTCATACCACATAGTAAACCCAAGCCCTTGACCCCTAACCGGGGC
CCTATCTGCCCTCCTAATAACATCGGGCCTAGCAATATGATTCCACTTCAACTCTACAACACT
ACTCCTCCTAGGACTAACAACAAACCTCTTAACAATATATCAATGATGACGTGACATTGTAC
GAGAAAGCACCTTTCAAGGCCACCACACACCCACAGTCCAAAAAGGACTACGATACGGCAT
AATCCTGTTCATTGTCTCAGAAGTATTTTTCTTCGCTGGCTTCTTCTGAGCATTCTACCACTCA
AGCCTAGCACCTACCCCCGAACTAGGAGGATGCTGACCCCCCACAGGCATCAACCCGCTAA
ACCCACTGGAAGTACCTCTACTCAACACATCCGTTCTTTTAGCCTCAGGAGTATCAATTACAT
GGGCACACCATAGCTTAATAGAAGGCAGCCGAAACCACATAATCCAGGCCCTGCTCATCAC
AATTCTCCTAGGCATTTACTTCACACTACTGCAGGTCTCAGAATATTACGAAGCACCCTTCAC
AATCTCCGACGGCGTGTACGGCTCTACCTTCTTTGTGGCAACTGGATTCCACGGCCTACACGT
CATCATCGGCACCTCCTTCCTAACTGTGTGCCTACTACGACAACTAAAATACCACTTTACATC
AAACCACCACTTCGGATTCGAAGCTGCCGCCTGATACTGACACTTCGTAGATGTAGTGTGAC
TGTTCCTGTATGTCTCCATTTACTGATGAGGTTCCTATTTTCTAAGTATGCACAGTACAGTTG
ACTTCCAATCAACAAGCTCTGGCCACAACCCAGAAGAGAATAATAAACCTACTCCTAGCAAT
AACAACAAACACCATACTAGCATGCCTGCTCATACTAATCGCCTTCTGACTCCCTCAATTAA
ACACGTACTCGGAAAAGATCACCCCCTACGAATGCGGATTCGACCCCATGGGATCGGCACG
ACTACCATTCTCCATAAAATTCTTCCTAATCGCCATCACGTTCCTACTATTCGACCTAGAAAT
CGCACTACTCCTGCCACTCCCCTGAGCATCACAAACAAACAACCTAGGCACCATAATCACCG
TAGCCTTGGTCCTCATCCTACTGCTAGCAATTAGCCTGGCCTACGAATGAACACAAAAAGGC
CTAGAATGAACTGAGTATGGTAGCTAGTTTACACAAAACAAATGATTTCGACTCATTAAACT
ATGACTTACTCATAGCTACCAAATGTCCCTAATTTACATTAACACAACACTGGCATTCACTAT
CTCCCTAATGGGGATATTAATGTACCGATCACACCTAATGTCCTCACTACTATGTCTAGAGG
GCATAATACTCTCCCTATTCGTAATAATCACCATTACAATCCTAACTAACCACTTCACACTAG
CCAACATAGCACCCATCATCCTCCTCGTACTAGCAGCATGCGAGGCTGCCCTAGGCCTCTCT
CTACTAGTACTTGTATCTAACACATACGGCACGGACTACGTACAAAACCTAAACCTGCTACA
ATGCTAAAACTAATTGTCCCCACAGCCATACTAATTCCCCTAACATGACTATCGAACAAAAA
CATAGTGTGAATCAACGTAACATCACACAGCATGCTAATCAGCCTAACATCTCTAGGCATCT
TGGGCCAGCATGACCACAACAACATAAGCCTCTCAGCCATCTTCTTCTCTGACCCCCTATCA
GCACCCTTAATCGTATTAACAACATGACTCCTCCCCCTAATACTAACAGCCAGCCAAGCCCA

CCTCTCCAATGAGCCCCTGGGCCTAAAAAAACTATACATTACCATACTAATCACCCTCCAAG
CACTACTAATCATAACATTCGCCTCCTCTGAGTTCATTATATTCTACATCCTGTTTGAAGCAA
CACTCGTACCAACCCTAATCATCATCACACGATGAGGAAACCAAGCAGAGCGACTAAACGC
AGGGTCCTACTTCCTCTTCTACACAATAGTAGGATCACTCCCCCTTCTAGTAGTACTCACATA
CACCCAAAACATAACAGGAACCCTAAACATACTAGTACTACAATACTGAGCGAAACCCATA
AACGACTCTTGATCCAACATGCTAATATGACTAGCATGCATAATAGCATTCATAGTAAAAAT
ACCCCTATACGGACTACACCTCTGACTGCCAAAAGCCCACGTAGAGGCCCCCATTGCAGGCT
CCATAGTACTTGCAGCCGTACTACTAAAACTAGGCGGATACGGCATAATACGCATCACTATC
ATGCTAGAACCAATAACAACATTCATAGCATACCCCTTCCTAATACTATCCCTGTGAGGAAT
AATCATAACAAGCTCCATCTGCCTTCGCCAAACCGACCTAAAATCACTAATCGCCTACTCCT
CCGTAAGCCACATAGCACTGGTAATCGTCGCAATCCTAATCCAAACCCCATGAAGCTACATG
GGGGCCACCGCTCTCATAATCGCCCACGGTCTAACATCCTCCATACTATTTTGCCTAGCAAA
CACAAACTATGAGCGAACCCACAGCCGAACTATAATACTAGCACGAGGACTGCAAACCCTC
CTACCCCTAATAGCTGCCTGATGACTCCTAGCAAGCCTAACCAACCTGGCCCTCCCCCCCAG
CATCAACCTAATCGGAGAACTATTCGTAGTAGTATCAACATTCTCGTGGTCCAACACCACCA
TCATCCTTACTGGAACAAACATTATCATCACAGCCACCTACTCCCTATACATACTAATATCCA
CCCAACGTGGAAAATACACCCACCACATCAACAACATCTGCCCCTCCTTCACCCGAGAAAAC
GCTCTAATGGCACTCCACATACTGCCCCTACTAATGCTATCAACCAACCCCAAAATCATCCT
AGGGTGCCTTTACTGTAAGTATAGTTTAATAAAACCTCAGATTGTGGATCTGACAATAGAAG
ACCACAACTTCTTACTTACCAAAAAAGCATGCAAGAACTGCTAATTCCTGCCCCCATGTATA
AAAACATGGCTTTTTTAAACTTTTAGAGGATGACAGACACCCGTTGGTCTTAGGAACCAAAA
GACTTGGTGCAACTCCAAGTAAAAGTAATTAACACCCTAACCTGCACCTCCCTTATAACACT
AACAACACTAATCATACCAATCATAATCACCCCCACAAATGCCTACACAAGCAAAAACTAC
CCCCACCACGTAAAAAACATAGTCGCACTAGCCTTCGCCGTCAGCATAATCCCAGCAATAGC
ATTTATATACTCAAACGAAGAAATAACCATCTTCAACTGACACTGAACAACAATCCAAACAA
TAAAAATAACAATAAGCTTCAAACTAGACTATTTCTCCACAACATTCATACCAGTGGCCCTA
TTCGTCACATGGTCTATCATAGAATTCTCTCTATGATATATAAAATCAGACCCCCACATCAAC
CGATTCTTCAAGTACCTACTAATCTTCCTAATCACAATAATAATCCTAGTATCCGCCAACAAC
ATATTCCAACTCTTCATCGGCTGAGAAGGAGTAGGAATCATATCTTTCCTCCTAATCGGCTG
ATGGCACGGACGAACAGATGCAAACACAGCAGCCATACAGGCCATCCTATACAACCGCATC
GGAGACATTGGACTAATCCTGTCAATGGCATGATTCCTCACAAACCTAAACTCATGGGACCT
CCAACAAATCCTCATGCTAAACCCCGAGAACACAAACATCCCCCTAGCGGGCCTACTACTAG
CAGCAACTGGAAAATCCGCACAATTTGGGCTACACCCATGACTCCCCTCAGCCATAGAAGGC
CCAACCCCAGTATCAGCCCTACTCCACTCCAGCACAATAGTAGTCGCAGGGGTATTCCTACT
AATCCGATTCCACCCCCTAATAGAAAACAACAAAACAATCCAAACAACAACCCTATGCCTA
GGGGCCATTACAACCCTGTTCACAGCCATCTGTGCTCTCACCCAAAACGACATCAAAAAGAT
CGTGGCCTTCTCAACCTCAAGCCAACTAGGTCTAATAATAGTCACCATCGGAATCAACCAAC
CACACCTAGCCTTCATACACATCTGCACCCACGCCTTTTTCAAAGCCATACTATTCATGTGCT
CCGGATCGATCATCCACAGCCTAAACGACGAACAAGACATCCGCAAAATAGGAGGACTTTT
CCACGCCCTCCCCACTACCACCTCCGCTCTCATTATTGGCAGCCTAGCCCTAACAGGAACCC
CATTCCTAACAGGCTTCTACTCTAAAGACCTAATCATCGAAACCGCTAACATATCCCACACA
AACGCCTGAGCCCTGCTCATCACACTAGTAGCCACCTCCCTCACAGCCGCCTACAGCACACG
AATCATCTTCTTCACACTATTAGGACAACCCCGATCCAACACCCTAATCAACATCAACGAAA
ACAACCCCTCACTAACAAACCCAATCAAACGCCTAATGCTAGGGAGCATCTTCGCCGGATTC
CTACTATACAACAACATCCCCCCAACAACCATGCCCCAAACAACTATACCACAATACCTAAA
ATACACCGCCCTCGCCACTACAATCCTAGGCCTAATCATAGCCCTAGAACTGTGCAACCTGT
CCCTAAACCTAAGCCACAAACCCCCCTCAAGTGCCTTCAAATTCTCAACCTTACTAGGCTAC
TTCCCCACAATCATTCACCGCTCAGGACCCCTCTGATCACTAACAACAAGCCAAAAACTAGC
ATCCCTTATTCTAGACCTAATCTGACTAGAAAATATCATACCAAAGTCAATCTCACACTTCCA
CATAAAAGCCTCCCTAATAACATCCAACCAAAAAGGCTCAATCAAACTATACTTCCTGTCCT
TCGCAGTGACAATAGTCCTCGCTCTCCTCATATTCTATTCCCCCGAGTAACCTCCATAACAAC

AACAACACAAATAAACAAAGACCACCCGGTCATAATCACAAACCAAGCACCATGGCTATAA
AGAGCAGCAACACCCGCAGCTTCCTCACTAAAAAAACTAGACCCCCCAACATCATAAGTCG
CCCAATCACCAGGACCACCAAGATCAAAAACCACACTCAACCCCTCACCCTTCAACATATAA
AGCACTAAAACCAACTCTATGACCACACCCAAAACAAAAGCACCGAGCACAACCACATTAG
ACACCCAAACTTCAGGATACTCCTCAATGGCTATGGCCGCCGTATAACCAAAAACAACCAAC
ATCCCCCCAAGATAAATCAAAAAAACCATTAAACCCAAAAAAGACCCACCAAAATTAACTA
CAACCCCACAACCTGCACCGCCAGCTACAACCAGACTAAACCCCCCATAAATAGGAGAAGG
CTTTGAAGAAAACCCTAAAAAACTAATCACAAAAATAACACTCAAAATAAACACAACATAT
GTCATTGTTCCCACATGGACTCAAACCATGACCTATGACACGAAAAATCATCGTTGTAATTA
AACTACGGAAACCTAATGACAAACATCCGAAAAACACACCCCCTACTCAAAATCATCAACA
ACTCCTTTATTGATCTCCCCACCCCCTCTAACATCTCAGCATGATGAAACTTCGGATCACTAC
TAGGAATCTGCTTAGTCCTACAAATTCTCACAGGACTATTTCTAGCCATACACTACACAGCA
GACACAGCAACCGCATTCTCATCAGTAACTCACATCTGCCGAGATGTAAACTACGGATGAAT
TATCCGATACATACACGCTAACGGAGCTTCCCTGTTCTTCATCTGCCTATTCGCACACATCGG
ACGAGGCATCTACTACGGATCCTTTGCCTACAAAGAGACATGAAACATCGGTATCCTGCTCC
TATTTGCAGTAATAGCAACAGCCTTTATGGGATACGTCCTACCATGAGGACAAATGTCCTTC
TGAGGTGCTACGGTAATTACAAACCTTTTATCCGCAATACCCTACATCGGGTCTAGCCTAGT
AGAGTGAGTCTGAGGGGGATTCTCGGTAGACAAAGCAACTCTCACTCGATTCTTCGCTCTTC
ACTTCATCCTTCCCTTCGTAATTCTTGCCCTAGTACTAGTACACTTACTATTCTTACACGAAA
CCGGATCCAACAACCCAATAGGAATCGTATCCAACCCCGACGTAATCCCCTTCCACCCATAC
TACACAGCCAAGGACACCCTTGGCCTATTCATCATGCTCACAGCACTAATATCCTTAGCCCT
ATTCTTCCCCGACCTACTAGGAGACCCAGACAATTATACACCCGCAAACCCCCTAAACACAC
CACCCCACATCAAACCAGAATGGTACTTCCTATTCGCATACGCAATCCTACGCTCAATTCCC
AACAAATTGGGAGGAGTACTAGCACTAATCCTATCCATCCTCGTACTAGCACTAATCCCACT
ACTACACACATCAAAACAACGAACTATAATGTTCCGACCCCTGAGCCAAACCATCTTCTGAC
TCCTGGTGGCCGACCTACTAGTTCTCACATGAATCGGAGGACAACCCGTGGAACACCCCTTC
ATCCTAATCGGACAAGTAGCCTCTATCCTTTATTTCACACTAATCCTAGCAGCAATGCCAATC
GCAGGTATCATCGAAAACAATCTCATAAAATGAAGAGTCTTTGTAGTATATGTCAATACACT
GGTCTTGTAAACCAGCAAAGGAATTAACCCTCCCCAAGACTCAGGAAGAAGACAAAAGCCC
TACCATCAGCACCCAAAGCTGAAATTCTCAATAAACTACTTCCTGCAAACAATTAAAGTCCA
ACAAGCTTTATATAGTATATGCCATGTATAATCGTGCATTAATGGTTTGCCCCATGCATATAA
GCAGGTACATTATATTATTATAGTACATAGGACATATTATGTATAATCGTGCATTATTGATCT
AGTACATACATATAAGCAGGTACATTATATTATTATAGTACATAGGACATATTATGTATAAT
CGTGCATTATTGATCTAGTACATGCATATAAGCAGGTACATAATATCCTTAGAGTACATAGT
ACATATTATTATTGATCGGACATAGCACATCAAGTCAAATCATTTCCAGTCAACATGCGTAT
CCCTACCACTGAAGGCCGCCTAATCACCATGCCGCGTGAAATCATCAACCCGCTCATAGTCG
TGTCCCTCTTCTCGCTCCGGGCCCATACGGACTGTGGGGTAGTTAAAGTGAAACTATACCTG
GCATCTGGTTCTTACTTCATGTTCATCTTATATCTAGCCGCTCACTCGTTCCTCTTAAATAAGA
CATCTCGATGGATTAATTACTAATCAGCCCATGCCTAACATAACTGTGCTGTCATGCCTTTGG
TATCTTTTAATTTTCGGGGTGCGAGGTTCAACTGGGTCACCGACCTTCGAGCAGGTTGATAA
CTTGTAGATAGACATTCATTGAATATTATTGGTCGTACATACTAACTCCTAGGTGTTATTCAG
TCAATGGTTACAGGACATAGAGAATTTTACAACAAAATTTTGAGTTGAGCCTACAGAAATCA
TAGATCACAATCGCAGACACCAACAAATACTAAAGTAATTAATTTAAATTTATAATTTTAGG
TTTGATATATCAAACCCCCCCTTACCCCCCCAAAGCCTCCACGTACTAAACATCTTGTCAAAC
CCCAAAAGCAAGAATACACACGTACACAAAGGCGCTATAAGCACAAAGTATTACGTAAGTA
TATAGATATACAAAACTACTAGCGCAGCTTTCTGTCCACTAGTTAAATACTTAGGTGCTTGA
ACATATATGGCTGGAAGGCCATGTAGTGACTACGTAATGATTCCTATTAGCCACAATAAAAA
CAATATACAATAAATACAAATATAGATCAATTCTTATAGGCGCCGATAGCATAAACTATCTG
CCCCCTATGTACAATTGAATTAAA
>SRR7507293 (circular) REORGANIZED

GTTAATGTAGCTTAAAACCAAAGCAAAGCATTGAAAATGCTTAGATGAGTCCACAAACTCC
ATAAACACACAGGTTTGGTCCCAGCCTTTTTATTAATTTTCAATAGGATTACACATGCAAGTA
TCCGCCCCCCAGTGAAGATGCCCTTCAAGTTATCACAGTACCTGAAGGAGCTGGCATCAAGC
ACGCCAATAAACGCAGCTAACGACGCCTTGCAGAGCCACACCCCCACGGGAAACAGCAGTG
ATAAAAATTAGGCTATAAACGAAAGTTCGACCTAGCCATATTGCATTGGGTTGGTAAATCTC
GTGCCAGCCACCGCGGTCATACGATTAACCCTAGCTAATAAAAAACCGGCGTAAAGCGTGC
TAAGATAAATCCAATCCAAATAAAGTTAAGCCCTGACCAGGCCGTAAAAAGCCGTGGTTAC
CGTAAAAATAAACTACGAAAGTAACTTTAATTCACATCGACACACGATAGCTAAGACCCAA
ACTGGGATTAGATACCCCACTATGCTTAGCCCTAAACCTAAATAATTCACCAGACAAAATTA
TTCGCCAGAGAACTACTGGCAACAGCCTAAAACTCAAAGGACTTGGCGGTGCTTTACATCCC
CCTAGAGGAGCCTGTTCTGTAATCGATAAACCCCGATAAACCCTACCAATTCTAGCTAATAC
AGCCTATATACCGCCATCTCCAGCAAACCCTAAAAAGGAAGCACAGTAAGCAAGACTATGA
AAACATAAATACGTTAGGTCAAGGTGTAGCCCATGAATTGGGAAGAAATGGGCTACATTTTC
TAAAATAGAACACAAACGAACGCCCTAATGAAAACGAGGGCCAAAGGAGGATTTAGCAGT
AAGCTGAGAATAGAAAGCTCAACTGAACCCGGCCCTAAAGCACGCACACACCGCCCGTCAC
CCTCTTCAAATCCCCAAGAATACCTAAACATATTAGACAAACACCAAGGCATGAGAAGAGA
CAAGTCGTAACAAGGTAAGCATACTGGAAGGTGTGCTTGGATCACCAAAGTGTAGCTTAAA
CAAAGCATCTGGCCTACACCCAGAAGATCTCAATAACATGACCACTTTGAACAAATCCTAGC
CCAACCAACACCCAACATACAACCAAACAGAGCACATAAACCAAAGCATTTACTAGACTAA
AGTATAGGCGATAGAAATTACACAACGGCGCTATAGAAAAAGTACCGCAAGGGAAAGATG
AAAGATGCATTCAAAGTACAAAAAAGCAAAGATTACCCCTTGTACCTTTTGCATAATGGACT
AACTAGAAACACCCTAGCAAAGAGAACTTAAGCTAGAAACCCCGAAACCAGACGAGCTACC
TACGAGCAGTTTAAAGAACCCACTCATCTATGTGGCAAAATAGTGAGAAGACTTGTAGGTA
GAGGTGAAAAGCCTAACGAGCCTGGTGATAGCTGGTTGTCCAAGAAATGAATCTAAGTTCA
ACTTGAAGCATACCCAAAAGCCCAAAAACTATAATGTAGGCTTCAAGTATAGTCTAAAAAG
GTACAGCTTTTTAGAAACAGAATTAAATCTTAATTAGTGAGTAAACAATACAACAACCATAG
TTGGCCTAAAAGCAGCCATCAATTAAGAAAGCGTCAAAGCTCAACAATAAGACATAAATAA
TACCACAAATAAAAATCAACTCCTAAACCAATATTGGACCAATCTATCAATAAATAGAAGA
AATACTGTTAGTATGAGTAACAAGAAATAGATCTCCTTGCACAAGCTTATATCAGAACGGAT
GACCCACTGATAATTAACAACGAAACAAATCAAACCCAAAAATAGAACTTTGTTAAATAGA
TTGTTAACCCAACACAGGCGTGCATATTCTCAAAGGAAAGGTTAAAACAAATGAAAGGAAC
TCGGCAAACACAAGCCCCGCCTGTTTACCAAAAACATCACCTCTAGCATAACCAGTATTAGA
GGCACTGCCTGCCCAGTGACTCGCGTTAAACGGCCGCGGTATTCTGACCGTGCAAAGGTAGC
ATAATCACTTGTTCTCTAAATAAGGACTAGTATGAACGGCTAGACGAGGGTTTTACTGTCTC
TCATCTGTAACCAGTGAAATTGACCTCCCCGTGAAGAGGCGGGAATAATATAATAAGACGA
GAAGACCCTATGGAGCTTTAATTAAACAGCTTAAAATTAACCCAACACCACTCTAACAGAGA
TATAACAAACCAATTAGCCTAAGCTGTCAATTTTGGTTGGGGTGACCTCGGAGCAAAAAACA
ACCTACGAGCGGTCAAATCCAGACTAACAAGTCCAGATAATCCGTTAATTGATCCAACAACT
TGATCAACGGAACAAGTTACCCTAGGGATAACAGCGCAATCCTATTCGAGAGTCCATATCGA
CAATAGGGTTTACGACCTCGATGTTGGATCAGGACATCCCAATGGTGCAGAAGCTATTAATG
GTTCGTTTGTTCAACGATTAAAGTCCTACGTGATCTGAGTTCAGACCGGAGTAATCCAGGTC
GGTTTCTATCTATTAATACACTTCTCCCAGTACGAAAGGACAAGAGAAGTAGGGCCTACCTC
ACACAGGCGCCCTCGAATCAATAAATGACTACCCTCTTAATTTAGCCAATTCACAACAACCC
CCAGTCCTAGAACCAGGGCCAGCTAGGGTAGCAAAGCACGGCAACTGCACAAGACTTAAGC
TCTTGTAACAGAGGTTCAACTCCCCTCCCTAGCAGCAATGTACACAATCAACATAATAATAA
TGATCATCCCCATCCTACTAGCCGTGGCATTCCTAACGCTAGTAGAACGCAAAGTGCTAGGA
TACATGCAACTACGAAAGGGCCCAAACATCGTGGGCCCTTGAGGCCTGCTACAACCAATCG
CCGACGCAGTAAAACTATTCACTAAAGAACCCCTACGACCCCTAACATCCTCAATCACAATA
TTCATCATGGCACCTATCCTAGCACTGACACTCGCACTCACCATGTGAGTACCACTACCAAT
GCCACACCCACTAGTCAACATAAACCTAGGAGTGCTATTCATACTGGCCATATCAAGCCTCG
CCGTATACTCCATCTTATGATCAGGATGAGCCTCAAACTCCAAATACGCCCTCATTGGAGCA

CTACGTGCAGTGGCCCAAACCATCTCATACGAAGTGACACTAGCAATTATCCTGCTGTCCTT
ACTGTTAATAAGCGGGTCCTTCACCCTCTCCACCCTAATCACAACCCAAGAAAAGCTATGAT
TACTAGTGCCCGCATGACCCCTGGCCATAATATGATTCATCTCGACCCTAGCCGAGACAAAT
CGAGCACCCTTCGACCTAACAGAAGGAGAATCTGAACTCGTATCCGGCTTTAACGTTGAATA
CGCAGCAGGCCCATTCGCACTCTTCTTCCTTGCCGAATACGCAAACATCATCATAATAAACA
TCTTGTCAGTGACACTCTTCATAGGAGCCTTTCACGACCCCCACACCCCAAGCCTCTACACA
GTTAATTTTGTAGTAAAAACACTCGCCCTTACCGCCCTATTCCTATGAATCCGAGCATCCTAC
CCACGATTCCGCTACGACCAACTAATGCACCTACTATGAAAAAACTTCCTACCACTAACCCT
AGCACTGTGCATGTGACACGTATCAATACCAATCGCCCTGTCAAGCATTCCCCCACAATCAT
AAGAAATATGTCTGACAAAAGAATTACTTTGATAGAGTAAATAATAGAGGTTTAAATCCCCT
TATTTCTAGAAAAACAGGCATTGAACCTGCACCTGAGAACTCAAAAATCTCCGTGCTACCAA
CTTACACCACAATCTACAGTAAGGTCAGCTAAGCAAGCTATCGGGCCCATACCCCGAAAATG
TTGGATCACAACCTTCCCGTACTAATAAACCTCACTATACTAGCCATCCTAACAACCCTATTC
CTAGGGACCATGCTCGTCCTAATCAGCTCTCACTGACTAATGATTTGAATTGGATTCGAGAT
AAACATACTAGCAATAGTCCCTATCCTAATGAAAAACTTTAGCCCACGAGCCATAGAAGCA
GCCACAAAATACTTCCTAATCCAAGCCACCGCGTCCATACTACTAATGCTAGCCATTACCCT
AGACCTGATATCTTCTGGACAATGAACCATCACAAAAACACACAACACCCTACCATCAGCCA
TCATCACCGTAGCTATGGCCATAAAACTAGGAATAGCACCATTCCACTTCTGAGTGCCAGAA
GTAACACAAGGAAGCCCACTATCATCAGGCATGCTACTCCTAACCTGACAAAAAGTCGCAC
CAATATCAATCCTATACCAAATAATACCCACAATCAACACAAATATACTGACAACCATAGCC
GCACTCTCAATCCTCATCGGCGGTTGAGGAGGGCTAAACCAGACCCAACTACGAAAAATCA
TAGCATACTCCTCAATCGCCCACATAGGCTGAATAGCAATAATCATAACATACAACCCGGAC
ATTGCCATTCTAAACCTACTAGTCTACATCATAATGACACTATCTATATTCGCAATCCTACTA
TACAACTCATCAACAACAACCCTATCACTATCCCACCTATCAAACAAGACACCACTAATCAC
AGCCCTCGCACTACTAATCCTACTATCACTAGGAGGCCTCCCCCCACTGACCGGATTCATGC
CCAAATGAATAATCATCCAAGAACTAACCAAAAACAACATAGTAATAATACCAACCGTGAT
AGCAATAACAGCACTACTCAACCTATACTTCTACGTACGCCTGGCGTACTCCACAGCACTAA
CCATACTCCCAACCACCAACAACATAAAAATAAAATGACAATTCGAAACAACAAAAATCAT
AAAACTAACCCCACCTCTAATCATTTTATCCACAATAGCACTCCCACTCACCCCTATAATATC
AATCCTAAACTAGGGACTTAGGCTACACAGACCGAGAGCCTTCAAAGCTCTAAGCAAATAC
AAACTATTTAGTCCCTGATATAAAGACTGTAGAAATTCAACCTACATCACCTAAACGCAAAT
CAGGCGCTTTAATTAAGCTAAATCCTCACTAGATTGGTGGGATACAAACCCACGAAACTTTA
GTTAACAGCTAAACACCCTAATCAACTGGCTTCAATCTACTTCTCCCGCCATAAAGAAGAAA
GTGGCGGGAGAAGTCCGGGCAGAATTGAAGCTGCTTCTTTGAATTTGCAATTCAATGTGTAA
ACACCTCAGGACCTGGCAAGAAGAGGACTCCAACCTCTGTGTTCAGATTTACAGTCTAATGC
TTACTCGGCCATCTTACCTATGTTCACCAACCGCTGGCTCTTTTCAACAAATCACAAAGACAT
TGGCACACTATATCTCTTATTTGGCGCCTGGGCCGGAATAGTAGGCACCGCACTAAGTCTTC
TAATTCGCGCTGAGCTAGGTCAGCCTGGGACCCTCTTAGGGGACGACCAAATTTACAATGTG
ATCGTTACCGCACATGCATTTGTAATAATCTTCTTCATAGTCATGCCAATCATAATCGGAGGC
TTTGGAAACTGACTAGTACCCCTGATAATTGGAGCCCCTGACATGGCATTCCCCCGTATAAA
CAACATAAGTTTCTGACTGCTCCCCCCCTCTTTCCTACTTCTTCTGGCCTCTTCCATAGTCGAA
GCAGGGGCTGGAACCGGCTGAACTGTATACCCCCCTTTAGCAGGAAATTTAGCACACGCAG
GAGCATCTGTAGACCTAACCATCTTCTCTCTTCATCTAGCGGGTGTCTCATCAATCCTTGGGG
CTATCAATTTTATTACAACAATCATCAACATAAAACCCCCTGCAATAAACCAATACCAAACC
CCACTATTCGTGTGATCAGTCCTAATTACGGCCGTGCTTCTGCTACTATCTCTACCCGTACTA
GCTGCTGGCATTACCATACTGTTAACCGACCGTAATCTAAATACAACCTTTTTGACCCCGCA
GGAGGAGGGGACCCCATTCTATACCAACACCTATTCTGATTCTTCGGACACCCCGAAGTGTA
CATTCTCATCCTTCCTGGATTTGGAATAATCTCCCACATCGTAACCTATTACTCCGGGAAAAA
AGAGCCCTTTGGATACATGGGCATAGTCTGAGCAATAATATCCATTGGCTTCCTGGGCTTCA
TTGTATGGGCACACCACATGTTCACAGTGGGAATAGACGTTGATACACGGGCCTACTTCACA
TCAGCCACCATAATTATTGCTATCCCCACTGGAGTAAAGGTGTTTAGTTGACTAGCAACCCT

GCACGGAGGAAACGTAAAATGGGCCCCAGCTATACTATGGGCCCTGGGCTTTATCTTCCTGT
TTACAGTCGGGGGTCTAACTGGCATCGTGCTGGCCAACTCGTCCCTAGATATCGTCCTCCAT
GATACCTACTACGTAGTAGCCCACTTCCACTACGTTCTCTCCATGGGAGCAGTTTTCGCCATC
ATAGGAGGCTTCGTCCATTGATTCCCCCTGTTCTCAGGATACACGCTCAACAACACATGGGC
AAAAGTTCACTTTACAATCATATTCGTGGGCGTAAACATGACCTTTTTCCCCCAACACTTCCT
TGGGCTGTCTGGAATACCTCGACGATACTCGGACTACCCAGACGCTTACACAATGTGAAACA
CTGTGTCCTCCATAGGATCCTTCATCTCATTAACCGCCGTAATACTCATGGCCTTCATAATCT
GAGAGGCATTCGCCTCTAAACGGGAAGTCCTAATAGTGGAGTCTACAAATACCAACCTCGA
ATGACTACACGGCTGCCCACCACCCTACCACACATTCGAAGAACCTGCTTTCGTAAATCTGG
TCAAAACAAGAGAGGAAGGAATCGAACCCTCAAACAATGGTTTCAAGCCAATATCATAGCC
CCTATGTCTCTCTCAATCACAGAAGTATTAGTAAAATTTACATAACTTTGTCAAAGTTAAATT
ATAGGTTCAAGCCCTTTATACTTCCATGGCGTACCCGCTCCAATTAGGCTTTCAAGATGCCAC
GTCCCCAATCATAGAAGAATTACTCCACTTCCACGATCACACACTAATAATCGTGTTCTTGAT
CAGCTCCCTAGTACTATACATTATCTCTCTCATACTAACAACCAAACTCACCCACACAAGTA
CAATAGACGCTCAAGAAGTAGAAACCATTTGAACCATCCTCCCCGCAGTTATCCTAATCCTA
ATCGCCCTGCCCTCCCTACGCATCCTCTACATAATGGACGAGATTAACAACCCCGCCCTGAC
AGTAAAAACAATGGGCCATCAATGATACTGAAGCTACGAATACACAGACTACGAAGACCTA
AGCTTCGACTCATACATAATCCCAACTCAAGACCTAAAACCCGGCGAACTCCGACTTCTAGA
AGTGGACAACCGACTTGTACTACCCATAGACACAACCATCCGCATGCTCATCTCATCTGAAG
ACGTCCTGCACTCTTGAGCCGTCCCATCCCTGGGCCTAAAAACAGATGCTATCCCGGGGCGT
CTAAACCAAACAACCCTGATATCAACCCGGCCCGGCCTATTCTACGGACAGTGCTCAGAAAT
CTGTGGCTCAAATCACAGCTTCATGCCAATTGTCCTCGAACTAGTACCCCTAAAAACATTTG
AAAACTGAACTACGTCCCTACTGTAATTCATTGAGAAGCTAATAGCGCTAGCCTTTTAAGTT
AGAGATTGAGAGCACGCCTCTCCTCAATGACATGCCTCAACTAGACACTACAACATGATCCA
TTACAATTATATCCATGATCCTGACCCTCTTTGTCCTATTCCAATTAAAAATCTCTAAACACT
ACTACCCACACGACGCAGAGACCTCAACAAAAACACACCACAAAACACTCACCCCCTGAGA
AAAAAAATGAACGAAAATCTATTCACCTCTTTCATTACCCCAGTGATAATAGGGATCCCTAT
TGTAACAATTATCATTATGTTCCCAGTAATCCTCTTCCCAACATCAAACCGACTAATCAACAA
CCGCATTGTATCCATACAACAATGACTCCTAAAACAAACATCCAAACAAATAATAAGCATCC
ACAACTACAAAGGACAGACCTGAACCCTGATATTAATAACACTAATCATTTTCATCGCATCT
ACTAACCTACTAGGCCTGCTACCCCACTCATTCACCCCCACAGCCCAACTGTCAATAAACCT
GAGCATAGCCGTTCCTCTATGGGCAGCCACCGTAGTCACAGGTTTTCGACACAATACAAAAA
CATCTTTAGCCCACTTCCTACCCCAGGGAACACCAACCCCCCTTATCCCAGTGCTAGTGATCA
TTGAAACAATCAGCCTGCTAATCCAACCCATAGCGCTCGCAGTACGACTGACAGCCAACATC
ACCGCCGGCCACCTGTTAATACACCTAATCGGAAGCGCAACCCTTGCCCTAATATCAATCAA
CCTCACCGTGGCCACAACTACCTTCATCGTCCTAGTCCTGCTTACAATCCTTGAATTCGCAGT
CGCACTCATTCAGGCCTACGTCTTCACCCTTCTAATCAGCCTCTACTTACATGATAACACATA
ATGACCCACCAAACCCACTCATACCACATAGTAAACCCAAGCCCTTGACCCCTAACCGGGGC
CCTATCTGCCCTCCTAATAACATCGGGCCTAGCAATATGATTCCACTTCAACTCTACAACACT
ACTCCTCCTAGGACTAACAACAAACCTCTTAACAATATATCAATGATGACGTGACATTGTAC
GAGAAAGCACCTTTCAAGGCCACCACACACCCACAGTCCAAAAAGGACTACGATACGGCAT
AATCCTGTTCATTGTCTCAGAAGTATTTTTCTTCGCTGGCTTCTTCTGAGCATTCTACCACTCA
AGCCTAGCACCTACCCCCGAACTAGGAGGATGCTGACCCCCCACAGGCATCAACCCGCTAA
ACCCACTGGAAGTACCTCTACTCAACACATCCGTTCTTTTAGCCTCAGGAGTATCAATTACAT
GGGCACACCATAGCTTAATAGAAGGCAGCCGAAACCACATAATCCAGGCCCTGCTCATCAC
AATTCTCCTAGGCATTTACTTCACACTACTGCAGGTCTCAGAATATTACGAAGCACCCTTCAC
AATCTCCGACGGCGTGTACGGCTCTACCTTCTTTGTGGCAACTGGATTCCACGGCCTACACGT
CATCATCGGCACCTCCTTCCTAACTGTGTGCCTACTACGACAACTAAAATACCACTTTACATC
AAACCACCACTTCGGATTCGAAGCTGCCGCCTGATACTGACACTTCGTAGATGTAGTGTGAC
TGTTCCTGTATGTCTCCATTTACTGATGAGGTTCCTATTTCTAAGTATGCACAGTACAGTTG
ACTTCCAATCAACAAGCTCTGGCCACAACCCAGAAGAGAATAATAAACCTACTCCTAGCAAT

AACAACAAACACCATACTAGCATGCCTGCTCATACTAATCGCCTTCTGACTCCCTCAATTAA
ACACGTACTCGGAAAAGATCACCCCCTACGAATGCGGATTCGACCCCATGGGATCGGCACG
ACTACCATTCTCCATAAAATTCTTCCTAATCGCCATCACGTTCCTACTATTCGACCTAGAAAT
CGCACTACTCCTGCCACTCCCCTGAGCATCACAAACAAACAACCTAGGCACCATAATCACCG
TAGCCTTGGTCCTCATCCTACTGCTAGCAATTAGCCTGGCCTACGAATGAACACAAAAAGGC
CTAGAATGAACTGAGTATGGTAGCTAGTTTACACAAAACAAATGATTTCGACTCATTAAACT
ATGACTTACTCATAGCTACCAAATGTCCCTAATTTACATTAACACAACACTGGCATTCACTAT
CTCCCTAATGGGGATATTAATGTACCGATCACACCTAATGTCCTCACTACTATGTCTAGAGG
GCATAATACTCTCCCTATTCGTAATAATCACCATTACAATCCTAACTAACCACTTCACACTAG
CCAACATAGCACCCATCATCCTCCTCGTACTAGCAGCATGCGAGGCTGCCCTAGGCCTCTCT
CTACTAGTACTTGTATCTAACACATACGGCACGGACTACGTACAAAACCTAAACCTGCTACA
ATGCTAAAACTAATTGTCCCCACAGCCATACTAATTCCCCTAACATGACTATCGAACAAAAA
CATAGTGTGAATCAACGTAACATCACACAGCATGCTAATCAGCCTAACATCTCTAGGCATCT
TGGGCCAGCATGACCACAACAACATAAGCCTCTCAGCCATCTTCTTCTCTGACCCCCTATCA
GCACCCTTAATCGTATTAACAACATGACTCCTCCCCCTAATACTAACAGCCAGCCAAGCCCA
CCTCTCCAATGAGCCCCTGGGCCTAAAAAAACTATACATTACCATACTAATCACCCTCCAAG
CACTACTAATCATAACATTCGCCTCCTCTGAGTTCATTATATTCTACATCCTGTTTGAAGCAA
CACTCGTACCAACCCTAATCATCATCACACGATGAGGAAACCAAGCAGAGCGACTAAACGC
AGGGTCCTACTTCCTCTTCTACACAATAGTAGGATCACTCCCCCTTCTAGTAGTACTCACATA
CACCCAAAACATAACAGGAACCCTAAACATACTAGTACTACAATACTGAGCGAAACCCATA
AACGACTCTTGATCCAACATGCTAATATGACTAGCATGCATAATAGCATTCATAGTAAAAAT
ACCCCTATACGGACTACACCTCTGACTGCCAAAAGCCCACGTAGAGGCCCCCATTGCAGGCT
CCATAGTACTTGCAGCCGTACTACTAAAACTAGGCGGATACGGCATAATACGCATCACTATC
ATGCTAGAACCAATAACAACATTCATAGCATACCCCTTCCTAATACTATCCCTGTGAGGAAT
AATCATAACAAGCTCCATCTGCCTTCGCCAAACCGACCTAAAATCACTAATCGCCTACTCCT
CCGTAAGCCACATAGCACTGGTAATCGTCGCAATCCTAATCCAAACCCCATGAAGCTACATG
GGGGCCACCGCTCTCATAATCGCCCACGGTCTAACATCCTCCATACTATTTTGCCTAGCAAA
CACAAACTATGAGCGAACCCACAGCCGAACTATAATACTAGCACGAGGACTGCAAACCCTC
CTACCCCTAATAGCTGCCTGATGACTCCTAGCAAGCCTAACCAACCTGGCCCTCCCCCCCAG
CATCAACCTAATCGGAGAACTATTCGTAGTAGTATCAACATTCTCGTGGTCCAACACCACCA
TCATCCTTACTGGAACAAACATTATCATCACAGCCACCTACTCCCTATACATACTAATATCCA
CCCAACGTGGAAAATACACCCACCACATCAACAACATCTGCCCCTCCTTCACCCGAGAAAAC
GCTCTAATGGCACTCCACATACTGCCCCTACTAATGCTATCAACCAACCCCAAAATCATCCT
AGGGTGCCTTTACTGTAAGTATAGTTTAATAAAACCTCAGATTGTGGATCTGACAATAGAAG
ACCACAACTTCTTACTTACCAAAAAAGCATGCAAGAACTGCTAATTCCTGCCCCCATGTATA
AAAACATGGCTTTTTAAACTTTTAGAGGATGACAGACACCCGTTGGTCTTAGGAACCAAAA
GACTTGGTGCAACTCCAAGTAAAAGTAATTAACACCCTAACCTGCACCTCCCTTATAACACT
AACAACACTAATCATACCAATCATAATCACCCCCACAAATGCCTACACAAGCAAAAACTAC
CCCCACCACGTAAAAAACATAGTCGCACTAGCCTTCGCCGTCAGCATAATCCCAGCAATAGC
ATTTATATACTCAAACGAAGAAATAACCATCTTCAACTGACACTGAACAACAATCCAAACAA
TAAAAATAACAATAAGCTTCAAACTAGACTATTTCTCCACAACATTCATACCAGTGGCCCTA
TTCGTCACATGGTCTATCATAGAATTCTCTCTATGATATATAAAATCAGACCCCCACATCAAC
CGATTCTTCAAGTACCTACTAATCTTCCTAATCACAATAATAATCCTAGTATCCGCCAACAAC
ATATTCCAACTCTTCATCGGCTGAGAAGGAGTAGGAATCATATCTTTCCTCCTAATCGGCTG
ATGGCACGGACGAACAGATGCAAACACAGCAGCCATACAGGCCATCCTATACAACCGCATC
GGAGACATTGGACTAATCCTGTCAATGGCATGATTCCTCACAAACCTAAACTCATGGGACCT
CCAACAAATCCTCATGCTAAACCCCGAGAACACAAACATCCCCCTAGCGGGCCTACTACTAG
CAGCAACTGGAAAATCCGCACAATTTGGGCTACACCCATGACTCCCCTCAGCCATAGAAGGC
CCAACCCCAGTATCAGCCCTACTCCACTCCAGCACAATAGTAGTCGCAGGGGTATTCCTACT
AATCCGATTCCACCCCCTAATAGAAAACAACAAAACAATCCAAACAACAACCCTATGCCTA
GGGGCCATTACAACCCTGTTCACAGCCATCTGTGCTCTCACCCAAAACGACATCAAAAAGAT

CGTGGCCTTCTCAACCTCAAGCCAACTAGGTCTAATAATAGTCACCATCGGAATCAACCAAC
CACACCTAGCCTTCATACACATCTGCACCCACGCCTTTTTCAAAGCCATACTATTCATGTGCT
CCGGATCGATCATCCACAGCCTAAACGACGAACAAGACATCCGCAAAATAGGAGGACTTTT
CCACGCCCTCCCCACTACCACCTCCGCTCTCATTATTGGCAGCCTAGCCCTAACAGGAACCC
CATTCCTAACAGGCTTCTACTCTAAAGACCTAATCATCGAAACCGCTAACATATCCCACACA
AACGCCTGAGCCCTGCTCATCACACTAGTAGCCACCTCCCTCACAGCCGCCTACAGCACACG
AATCATCTTCTTCACACTATTAGGACAACCCCGATCCAACACCCTAATCAACATCAACGAAA
ACAACCCCTCACTAACAAACCCAATCAAACGCCTAATGCTAGGGAGCATCTTCGCCGGATTC
CTACTATACAACAACATCCCCCCAACAACCATGCCCCAAACAACTATACCACAATACCTAAA
ATACACCGCCCTCGCCACTACAATCCTAGGCCTAATCATAGCCCTAGAACTGTGCAACCTGT
CCCTAAACCTAAGCCACAAACCCCCCTCAAGTGCCTTCAAATTCTCAACCTTACTAGGCTAC
TTCCCCACAATCATTCACCGCTCAGGACCCCTCTGATCACTAACAACAAGCCAAAAACTAGC
ATCCCTTATTCTAGACCTAATCTGACTAGAAAATATCATACCAAAGTCAATCTCACACTTCCA
CATAAAAGCCTCCCTAATAACATCCAACCAAAAAGGCTCAATCAAACTATACTTCCTGTCCT
TCGCAGTGACAATAGTCCTCGCTCTCCTCATATTCTATTCCCCCGAGTAACCTCCATAACAAC
AACAACACAAATAAACAAAGACCACCCGGTCATAATCACAAACCAAGCACCATGGCTATAA
AGAGCAGCAACACCCGCAGCTTCCTCACTAAAAAAACTAGACCCCCCAACATCATAAGTCG
CCCAATCACCAGGACCACCAAGATCAAAAACCACACTCAACCCCTCACCCTTCAACATATAA
AGCACTAAAACCAACTCTATGACCACACCCAAAACAAAAGCACCGAGCACAACCACATTAG
ACACCCAAACTTCAGGATACTCCTCAATGGCTATGGCCGCCGTATAACCAAAAACAACCAAC
ATCCCCCCAAGATAAATCAAAAAAACCATTAAACCCAAAAAAGACCCACCAAAATTAACTA
CAACCCCACAACCTGCACCGCCAGCTACAACCAGACTAAACCCCCCATAAATAGGAGAAGG
CTTTGAAGAAAACCCTAAAAAACTAATCACAAAAATAACACTCAAAATAAACACAACATAT
GTCATTGTTCCCACATGGACTCAAACCATGACCTATGACACGAAAAATCATCGTTGTAATTA
AACTACGGAAACCTAATGACAAACATCCGAAAAACACACCCCCTACTCAAAATCATCAACA
ACTCCTTTATTGATCTCCCCACCCCCTCTAACATCTCAGCATGATGAAACTTCGGATCACTAC
TAGGAATCTGCTTAGTCCTACAAATTCTCACAGGACTATTTCTAGCCATACACTACACAGCA
GACACAGCAACCGCATTCTCATCAGTAACTCACATCTGCCGAGATGTAAACTACGGATGAAT
TATCCGATACATACACGCTAACGGAGCTTCCCTGTTCTTCATCTGCCTATTCGCACACATCGG
ACGAGGCATCTACTACGGATCCTTTGCCTACAAAGAGACATGAAACATCGGTATCCTGCTCC
TATTTGCAGTAATAGCAACAGCCTTTATGGGATACGTCCTACCATGAGGACAAATGTCCTTC
TGAGGTGCTACGGTAATTACAAACCTTTTATCCGCAATACCCTACATCGGGTCTAGCCTAGT
AGAGTGAGTCTGAGGGGGATTCTCGGTAGACAAAGCAACTCTCACTCGATTCTTCGCTCTTC
ACTTCATCCTTCCCTTCGTAATTCTTGCCCTAGTACTAGTACACTTACTATTCTTACACGAAA
CCGGATCCAACAACCCAATAGGAATCGTATCCAACCCCGACGTAATCCCCTTCCACCCATAC
TACACAGCCAAGGACACCCTTGGCCTATTCATCATGCTCACAGCACTAATATCCTTAGCCCT
ATTCTTCCCCGACCTACTAGGAGACCCAGACAATTATACACCCGCAAACCCCCTAAACACAC
CACCCCACATCAAACCAGAATGGTACTTCCTATTCGCATACGCAATCCTACGCTCAATTCCC
AACAAATTGGGAGGAGTACTAGCACTAATCCTATCCATCCTCGTACTAGCACTAATCCCACT
ACTACACACATCAAAACAACGAACTATAATGTTCCGACCCCTGAGCCAAACCATCTTCTGAC
TCCTGGTGGCCGACCTACTAGTTCTCACATGAATCGGAGGACAACCCGTGGAACACCCCTTC
ATCCTAATCGGACAAGTAGCCTCTATCCTTTATTTCACACTAATCCTAGCAGCAATGCCAATC
GCAGGTATCATCGAAAACAATCTCATAAAATGAAGAGTCTTTGTAGTATATGTCAATACACT
GGTCTTGTAAACCAGCAAAGGAATTAACCCTCCCCAAGACTCAGGAAGAAGACAAAAGCCC
TACCATCAGCACCCAAAGCTGAAATTCTCAATAAACTACTTCCTGCAAACAATTAAAGTCCA
ACAAGCTTTATATAGTATATGCCATGTATAATCGTGCATTAATGGTTTGCCCCATGCATATAA
GCAGGTACATTATATTATTATAGTACATAGGACATATTATGTATAATCGTGCATTATTGATCT
AGTACATACATATAAGCAGGTACATTATATTATTATAGTACATAGGACATATTATGTATAAT
CGTGCATTATTGATCTAGTACATGCATATAAGCAGGTACATAATATCCTTAGAGTACATAGT
ACATATTATTATTGATCGGACATAGCACATCAAGTCAAATCATTTCCAGTCAACATGCGTAT
CCCTACCACTGAAGGCCGCCTAATCACCATGCCGCGTGAAATCATCAACCCGCTCATAGTCG

TGTCCCTCTTCTCGCTCCGGGCCCATACGGACTGTGGGGTAGTTAAAGTGAAACTATACCTG
GCATCTGGTTCTTACTTCATGTTCATCTTATATCTAGCCGCTCACTCGTTCCTCTTAAATAAGA
CATCTCGATGGATTAATTACTAATCAGCCCATGCCTAACATAACTGTGCTGTCATGCCTTTGG
TATCTTTTAATTTTCGGGGTGCGAGGTTCAACTGGGTCACCGACCTTCGAGCAGGTTGATAA
CTTGTAGATAGACATTCATTGAATATTATTGGTCGTACATACTAACTCCTAGGTGTTATTCAG
TCAATGGTTACAGGACATAGAGAATTTTACAACAAAATTTTGAGTTGAGCCTACAGAAATCA
TAGATCACAATCGCAGACACCAACAAATACTAAAGTAATTAATTTAAATTTATAATTTTAGG
TTTGATATATCAAACCCCCCCTTACCCCCCCAAAGCCTCCACGTACTAAACATCTTGTCAAAC
CCCAAAAGCAAGAATACACACGTACACAAAGGCGCTATAAGCACAAAGTATTACGTAAGTA
TATAGATATACAAAACTACTAGCGCAGCTTTCTGTCCACTAGTTAAATACTTAGGTGCTTGA
ACATATATGGCTGGAAGGCCATGTAGTGACTACGTAATGATTCCTATTAGCCACAATAAAAA
CAATATACAATAAATACAAATATAGATCAATTCTTATAGGCGCCGATAGCATAAACTATCTG
CCCCCTATGTACAATTGAATTAAA
>SRR7524043 (circular) REORGANIZED
GTTAATGTAGCTTAAAACCAAAGCAAAGCATTGAAAATGCTTAGATGAGCCCACAAACTCC
ATAAACACACAGGTTTGGTCCCAGCCTTTTTATTAATTTTCAATAGGATTACACATGCAAGTA
TCCGCCCCCCAGTGAAAATGCCCTTCAAGTTATCACAGTACCTGAAGGAGCTGGCATCAAGC
ACGCCAATAAACGCAGCTAACGACGCCTTGCAGAGCCACACCCCCACGGGAAACAGCAGTG
ATAAAAATTAGGCTATAAACGAAAGTTCGACCTAGCCATATTGCATTGGGTTGGTAAATCTC
GTGCCAGCCACCGCGGTCATACGATTAACCCTAGCTAATAAAAAACCGGCGTAAAGCGTGC
TAAGATAAATCCAATCCAAATAAAGTTAAGCCCTGACCAGGCCGTAAAAAGCCGTGGTTAC
CGTAAAAATAAACTACGAAAGTAACTTTAATTCACATCGACACACGATAGCTAAGACCCAA
ACTGGGATTAGATACCCCACTATGCTTAGCCCTAAACCTAAATAATTCACCAGACAAAATTA
TTCGCCAGAGAACTACTGGCAACAGCCTAAAACTCAAAGGACTTGGCGGTGCTTTACATCCC
CCTAGAGGAGCCTGTTCTGTAATCGATAAACCCCGATAAACCCTACCAATTCTAGCTAATAC
AGCCTATATACCGCCATCTCCAGCAAACCCTAAAAAGGAAGCACAGTAAGCAAGACTATGA
AAACATAAATACGTTAGGTCAAGGTGTAGCCCATGAATTGGGAAGAAATGGGCTACATTTTC
TAAAATAGAACACAAACGAACGCCCTAATGAAAACGAGGGCCAAAGGAGGATTTAGCAGT
AAGCTGAGAATAGAAAGCTCAACTGAACCCGGCCCTAAAGCACGCACACACCGCCCGTCAC
CCTCTTCAAATCCCCAAGAATACCTAAACATATTAGACAAACACCAAGGCATGAGAAGAGA
CAAGTCGTAACAAGGTAAGCATACTGGAAGGTGTGCTTGGATCACCAAAGTGTAGCTTAAA
CAAAGCATCTGGCCTACACCCAGAAGATCTCAATAACATGACCACTTTGAACAAATCCTAGC
CCAACCAACACCCAACATACAACCAAACAGAGCACATAAACCAAAGCATTTACTAGACTAA
AGTATAGGCGATAGAAATTACACAACGGCGCTATAGAAAAAGTACCGCAAGGGAAAGATG
AAAGATGCATTCAAAGTACAAAAAAGCAAAGATTACCCCTTGTACCTTTTGCATAATGGACT
AACTAGAAACACCCTAGCAAAGAGAACTTAAGCTAGAAACCCCGAAACCAGACGAGCTACC
TACGAGCAGTTTAAAGAACCCACTCATCTATGTGGCAAAATAGTGAGAAGACTTGTAGGTA
GAGGTGAAAAGCCTAACGAGCCTGGTGATAGCTGGTTGTCCAAGAAATGAATCTAAGTTCA
ACTTGAAGCATACCCAAAAGCCCAAAAACTATAATGTAGGCTTCAAGTATAGTCTAAAAAG
GTACAGCTTTTTAGAAACAGAATTAAATCTTAATTAGTGAGTAAACAATACAACAACCATAG
TTGGCCTAAAAGCAGCCATCAATTAAGAAAGCGTCAAAGCTCAACAATAAGACATAAATAA
TACCACAAATAAAAATCAACTCCTAAACCAATATTGGACCAATCTATCAATAAATAGAAGA
AATACTGTTAGTATGAGTAACAAGAAATAGATCTCCTTGCACAAGCTTATATCAGAACGGAT
GACCCACTGATAATTAACAACGAAACAAATCAAACCCAAAAATAGAACTTTGTTAAATAGA
TTGTTAACCCAACACAGGCGTGCATATTCTCAAAGGAAAGGTTAAAACAAATGAAAGGAAC
TCGGCAAACACAAGCCCCGCCTGTTTACCAAAAACATCACCTCTAGCATAACCAGTATTAGA
GGCACTGCCTGCCCAGTGACTCGCGTTAAACGGCCGCGGTATTCTGACCGTGCAAAGGTAGC
ATAATCACTTGTTCTCTAAATAAGGACTAGTATGAACGGCTAGACGAGGGTTTTACTGTCTC
TCATCTGTAACCAGTGAAATTGACCTCCCCGTGAAGAGGCGGGAATAATATAATAAGACGA
GAAGACCCTATGGAGCTTTAATTAAACAGCTTAAAATTAACCCAACACCACTCTAACAGAGA
TATAACAAACCAATTAGCCTAAGCTGTCAATTTTGGTTGGGGTGACCTCGGAGCAAAAAACA

ACCTACGAGCGGTCAAATCCAGACTAACAAGTCCAGATAATCCGTTAATTGATCCAACAACT
TGATCAACGGAACAAGTTACCCTAGGGATAACAGCGCAATCCTATTCGAGAGTCCATATCGA
CAATAGGGTTTACGACCTCGATGTTGGATCAGGACATCCCAATGGTGCAGAAGCTATTAATG
GTTCGTTTGTTCAACGATTAAAGTCCTACGTGATCTGAGTTCAGACCGGAGTAATCCAGGTC
GGTTTCTATCTATTAATACACTTCTCCCAGTACGAAAGGACAAGAGAAGTAGGGCCTACCTC
ACACAGGCGCCCTCGAATCAATAAATGACTACCCTCTTAATTTAGCCAATTCACAACAACCC
CCAGTCCTAGAACCAGGGCCAGCTAGGGTAGCAAAGCACGGCAACTGCACAAGACTTAAGC
TCTTGTAACAGAGGTTCAACTCCCCTCCCTAGCAGCAATGTACACAATCAACATAATAATAA
TGATCATCCCCATCCTACTAGCCGTGGCATTCCTAACGCTAGTAGAACGCAAAGTGCTAGGA
TACATGCAACTACGAAAGGGCCCAAACATCGTGGGCCCTTGAGGCCTGCTACAACCAATCG
CCGACGCAGTAAAACTATTCACTAAAGAACCCCTACGACCCCTAACATCCTCAACCACAATA
TTCATCATGGCACCTATCCTAGCACTGACACTCGCACTCACCATGTGAGTACCACTACCAAT
GCCACACCCACTAGTCAACATAAACCTAGGAGTGCTATTCATACTGGCCATATCAAGCCTCG
CCGTATACTCCATCTTATGATCAGGATGAGCCTCAAACTCCAAATACGCCCTCATTGGAGCA
CTACGTGCAGTGGCCCAAACCATCTCATACGAAGTGACACTAGCAATTATCCTGCTGTCCTT
ACTGTTAATAAGCGGGTCCTTCACCCTCTCCACCCTAATCACAACCCAAGAAAAGCTATGAC
TACTAGTGCCCGCATGACCCCTGGCCATAATATGATTCATCTCGACCCTAGCCGAGACAAAT
CGAGCACCCTTCGACCTAACAGAAGGAGAATCTGAACTCGTATCCGGCTTTAACGTTGAATA
CGCAGCAGGCCCATTCGCACTCTTCTTCCTTGCCGAATACGCAAACATCATCATAATAAACA
TCTTGTCAGTGACGCTCTTCATAGGAGCCTTTCACGACCCCCACACCCCAAGCCTCTACACA
GTTAATTTTGTAGTAAAAACACTCGCCCTTACCGCCCTATTCCTATGAATCCGAGCATCCTAC
CCACGATTCCGCTACGACCAACTAATGCACCTACTATGAAAAAACTTCCTACCACTAACCCT
AGCACTGTGCATGTGACACGTATCAATACCAATCGCCCTGTCAAGCATTCCCCCACAATCAT
AAGAAATATGTCTGACAAAAGAATTACTTTGATAGAGTAAATAATAGAGGTTTAAATCCCCT
TATTTCTAGAAAAACAGGCATTGAACCTGCACCTGAGAACTCAAAAATCTCCGTGCTACCAA
CTTACACCACAATCTACAGTAAGGTCAGCTAAGCAAGCTATCGGGCCCATACCCCGAAAATG
TTGGATCACAACCTTCCCGTACTAATAAACCTCACTATACTAGCCATCCTAACAACCCTATTC
CTAGGGACCATGCTCGTCCTAATCAGCTCTCACTGACTAATGATTTGAATTGGATTCGAGAT
AAACATACTAGCAATAGTCCCTATCCTAATGAAAAACTTTAGCCCACGGGCCATAGAAGCA
GCCACAAAATACTTCCTAATCCAAGCCACCGCGTCCATACTACTAATGCTAGCCATTACCCT
AGACCTGATATCTTCTGGACAATGAACCATCACAAAAACACACAACACCCTACCATCAGCCA
TCATCACCGTAGCTATGGCCATAAAACTAGGAATAGCTCCATTCCACTTCTGAGTGCCAGAA
GTAACACAAGGAAGCCCACTATCATCAGGCATGCTACTCCTAACCTGACAAAAAGTCGCAC
CAATATCAATCCTATACCAAATAATACCCACAATCAACACAAATATACTGACAACCATAGCC
GCACTCTCAATCCTCATCGGCGGTTGAGGAGGGCTAAACCAGACCCAACTACGAAAAATCA
TAGCATACTCCTCAATCGCCCACATAGGCTGAATAGCAATAATCATAACATACAACCCGGAC
ATTGCCATTCTAAACCTACTAGTCTACATCATAATGACACTATCTATATTCGCAATCCTACTA
TACAACTCATCAACAACAACCCTATCACTATCCCACCTATCAAACAAGACACCACTAATCAC
AGCCCTCGCACTACTAATCCTACTATCACTAGGAGGCCTCCCCCCACTGACCGGATTCATGC
CCAAATGAATAATCATCCAAGAACTAACCAAAAACAACATAGTAATAATACCAACCGTGAT
AGCAATAACAGCACTACTCAACCTATACTTCTACGTACGCCTGGCGTACTCCACAGCACTAA
CCATACTCCCAACCACCAACAACATAAAAATAAAGTGACAATTCGAAACAACAAAAATCAT
AAAACTAACCCCACCTCTAATCATTTTATCCACAATAGCACTCCCACTCACCCCTATAATATC
AATCCTAAACTAGGGACTTAGGCTACACAGACCGAGAGCCTTCAAAGCTCTAAGCAAATAC
AAACTATTTAGTCCCTGATATAAAGACTGTAGAAATTCAACCTACATCACCTAAACGCAAAT
CAGGCGCTTTAATTAAGCTAAATCCTCACTAGATTGGTGGGATACAAACCCACGAAACTTTA
GTTAACAGCTAAACACCCTAATCAACTGGCTTCAATCTACTTCTCCCGCCATAAAGAAGAAA
GTGGCGGGAGAAGTCCGGGCAGAATTGAAGCTGCTTCTTTGAATTTGCAATTCAATGTGTAA
ACACCTCAGGACCTGGCAAGAAGAGGACTCCAACCTCTGTGTTCAGATTTACAGTCTAATGC
TTACTCGGCCATCTTACCTATGTTCACCAACCGCTGGCTCTTTTCAACAAATCACAAAGACAT
TGGCACACTATATCTCTTATTTGGCGCCTGGGCCGGAATAGTAGGCACCGCACTAAGTCTTC

TAATTCGCGCTGAGCTAGGTCAGCCTGGGACCCTCTTAGGGGACGACCAAATTTACAATGTG
ATCGTTACCGCACATGCATTTGTAATAATCTTCTTCATAGTCATGCCAATCATAATCGGGGGC
TTTGGAAACTGACTAGTACCCCTGATAATTGGAGCCCCTGACATGGCATTCCCCCGTATAAA
CAACATAAGTTTCTGACTGCTCCCCCCCTCTTTCCTACTTCTTCTGGCCTCTTCCATAGTCGAA
GCGGGGGCTGGAACCGGCTGAACTGTATACCCCCCTTTAGCAGGAAATTTAGCACACGCAG
GAGCATCTGTAGACCTAACCATCTTCTCTCTTCATCTAGCGGGTGTCTCATCAATCCTTGGGG
CTATCAATTTTATTACAACAATCATCAACATAAAACCCCCTGCAATAAACCAATACCAAACC
CCACTATTCGTGTGATCAGTCCTAATTACGGCCGTGCTTCTGCTACTATCTCTACCCGTACTA
GCTGCTGGCATTACCATACTGTTAACCGACCGTAATCTAAATACAACCTTTTTTGACCCCGCA
GGAGGAGGTGACCCCATTCTATACCAACACCTATTCTGATTCTTCGGACACCCCGAAGTGTA
CATTCTCATCCTTCCTGGATTTGGAATAATCTCCCACATCGTAACCTATTACTCCGGGAAAAA
AGAGCCCTTTGGATACATGGGCATAGTCTGAGCAATAATATCCATTGGCTTCCTGGGCTTCA
TTGTATGGGCACACCACATGTTCACAGTGGGAATAGACGTTGATACACGGGCCTACTTCACA
TCAGCCACCATAATTATTGCTATCCCCACTGGAGTAAAGGTGTTTAGTTGACTAGCAACCCT
GCACGGAGGAAACGTAAAATGGGCCCCAGCTATACTATGGGCCCTGGGCTTTATCTTCCTGT
TTACAGTCGGGGGTCTAACTGGCATCGTGCTGGCCAACTCGTCCCTAGATATCGTCCTCCAT
GATACCTACTACGTAGTAGCCCACTTCCACTACGTTCTCTCCATGGGAGCAGTTTTCGCCATC
ATAGGAGGCTTCGTCCATTGATTCCCCCTGTTCTCAGGATACACGCTCAACAACACATGGGC
AAAAGTTCACTTTACAATCATATTCGTGGGCGTAAACATGACCTTTTTCCCCCAACACTTCCT
TGGGCTGTCTGGAATACCTCGACGATACTCGGACTACCCAGACGCTTACACAATGTGAAACA
CTGTGTCCTCCATAGGATCCTTCATCTCATTAACCGCCGTAATACTCATGGCCTTCATAATCT
GAGAGGCATTCGCCTCTAAACGGGAAGTCCTAATAGTGGAGTCCACAAATACCAACCTCGA
ATGACTACACGGCTGCCCACCACCCTACCACACATTCGAAGAACCTGCTTTCGTAAATCTGG
TCAAAACAAGAGAGGAAGGAATCGAACCCTCAAACAATGGTTTCAAGCCAATATCATAGCC
CCTATGTCTCTCTCAATCACAGAAGTATTAGTAAAATTTACATAACTTTGTCAAAGTTAAATT
ATAGGTTCAAGCCCTTTATACTTCCATGGCGTACCCGCTCCAATTAGGCTTTCAAGATGCCAC
GTCCCCAATCATAGAAGAATTACTCCACTTCCACGATCACACACTAATAATCGTGTTCTTGAT
CAGCTCCCTAGTACTATACATTATCTCTCTCATACTAACAACCAAACTCACCCACACAAGTA
CAATAGACGCTCAAGAAGTAGAAACCATTTGAACCATCCTCCCCGCAGTTATCCTAATCCTA
ATCGCCCTGCCCTCCCTACGCATCCTCTACATAATGGACGAGATTAACAACCCCGCCCTGAC
AGTAAAAACAATGGGCCATCAATGATACTGAAGCTACGAATACACAGACTACGAAGACCTA
AGCTTCGACTCATACATAATCCCAACTCAAGACCTAAAACCCGGCGAACTCCGACTTCTAGA
AGTGGACAACCGACTTGTACTACCCATAGACACAACCATCCGCATGCTCATCTCATCTGAAG
ACGTCCTGCACTCTTGAGCCGTCCCATCCCTGGGCCTAAAAACAGATGCTATCCCGGGGCGT
CTAAACCAAACAACCCTGATATCAACCCGGCCCGGCCTATTCTACGGACAGTGCTCAGAAAT
CTGTGGCTCAAATCACAGCTTCATGCCAATTGTCCTCGAACTAGTACCCCTAAAAACATTTG
AAAACTGAACTACGTCCCTACTGTAATTCATTGAGAAGCTAATAGCGCTAGCCTTTTAAGTT
AGAGATTGAGAGCACGCCTCTCCTCAATGACATGCCTCAACTAGACACTACAACATGATCCA
TTACAATTATATCCATGATCCTGACCCTCTTTGTCCTATTCCAATTAAAAATCTCTAAACACT
ACTACCCACACGACGCAGAGACCTCAACAAAAACACACCACAAAACACTCACCCCCTGAGA
AAAAAAATGAACGAAAATCTATTCACCTCTTTCATTACCCCAGTGATAATAGGGATCCCTAT
TGTAACAATTATCATTATGTTCCCAGTAATCCTCTTCCCAACATCAAACCGACTAATCAACAA
CCGCATTGTATCCATACAACAATGGCTCCTAAAACAAACATCCAAACAAATAATAAGCATCC
ACAACTACAAAGGACAGACCTGAACCCTGATATTAATAACACTAATCATTTTCATCGCATCT
ACTAACCTACTAGGCCTGCTACCCCACTCATTCACCCCCACAGCCCAACTGTCAATAAACCT
GAGCATAGCCGTTCCTCTATGGGCAGCCACCGTAGTCACAGGTTTTCGACACAATACAAAAA
CATCTTTAGCCCACTTCCTACCCCAGGGAACACCAACCCCCCTTATCCCAGTGCTAGTGATCA
TTGAAACAATCAGCCTGCTAATCCAACCCATAGCGCTCGCAGTACGACTGACAGCCAACATC
ACCGCCGGCCACCTGTTAATACACCTAATCGGAAGCGCAACCCTTGCCCTAATATCAATCAA
CCTCACCGTGGCCACAACTACCTTCATCGTCCTAGTCCTGCTTACAATCCTTGAATTCGCAGT
CGCACTCATTCAGGCCTACGTCTTCACCCTTCTAATCAGCCTCTACTTACATGATAACACATA

ATGACCCACCAAACCCACTCATACCACATAGTAAACCCAAGCCCTTGACCCCTAACCGGGGC
CCTATCTGCCCTCCTAATAACATCGGGCCTAGCAATATGATTCCACTTCAACTCTACAACACT
ACTCCTCCTAGGACTAACAACAAACCTCTTAACAATATATCAATGATGACGTGACATTGTAC
GAGAAAGCACCTTTCAAGGCCACCACACACCCACAGTCCAAAAAGGACTACGATACGGCAT
AATCCTGTTCATTGTCTCAGAAGTATTTTTCTTCGCTGGCTTCTTCTGAGCATTCTACCACTCA
AGCCTAGCACCTACCCCCGAACTAGGAGGATGCTGACCCCCCACAGGCATCAACCCGCTAA
ACCCACTGGAAGTACCTCTACTCAACACATCCGTTCTTTTAGCCTCAGGAGTATCAATTACAT
GGGCACACCATAGCTTAATAGAAGGCAGCCGAAACCACATAATCCAGGCCCTGCTCATCAC
AATCCTCCTAGGCATTTACTTCACACTACTGCAGGTCTCAGAATATTACGAAGCACCCTTCAC
AATCTCCGACGGCGTGTACGGCTCTACCTTCTTTGTGGCAACTGGATTCCACGGCCTACACGT
CATCATCGGCACCTCCTTCCTAACTGTGTGCCTACTACGACAACTAAAATACCACTTTACATC
AAACCACCACTTAGGATTCGAAGCTGCCGCCTGATACTGACACTTCGTAGATGTAGTGTGAC
TGTTCCTGTATGTCTCCATTTACTGATGAGGTTCCTATTTTCTAAGTATGCACAGTACAGTTG
ACTTCCAATCAACAAGCTCTGGCCACAACCCAGAAGAGAATAATAAACCTACTCCTAGCAAT
AACAACAAACACCATACTAGCATGCCTGCTCATACTAATCGCCTTCTGACTCCCTCAATTAA
ACACGTACTCGGAAAAGATCACCCCCTACGAATGCGGATTCGACCCCATGGGATCGGCACG
ACTACCATTCTCCATAAAATTCTTCCTAATCGCCATCACATTCCTACTATTCGACCTAGAAAT
CGCACTACTCCTGCCACTCCCCTGAGCATCACAAACAAACAACCTAGGCACCATAATCACCG
TAGCCTTGGTCCTCATCCTACTGCTAGCAATTAGCCTGGCCTACGAATGAACACAAAAAGGC
CTAGAATGAACTGAGTATGGTAGCTAGTTTACACAAAACAAATGATTTCGACTCATTAAACT
ATGACTTACTCATAGCTACCAAATGTCCCTAATTTACATTAACACAACACTGGCATTCACTAT
CTCCCTAATGGGGATATTAATGTACCGATCACACCTAATGTCCTCACTACTATGTCTAGAGG
GCATAATACTCTCCCTATTCGTAATAATCACCATTACAATCCTAACTAACCACTTCACACTAG
CCAACATAGCACCCATCATCCTCCTCGTACTAGCAGCATGCGAGGCTGCCCTAGGCCTCTCT
CTACTAGTACTTGTATCTAACACATACGGCACGGACTACGTACAAAACCTAAACCTGCTACA
ATGCTAAAACTAATTGTCCCCACAGCCATACTAATTCCCCTAACATGACTATCGAACAAAAA
CATAGTGTGAATCAACGTAACATCACACAGCATGCTAATCAGCCTAACATCTCTAGGCATCT
TGGGCCAGCATGACCACAACAACATAAGCCTCTCAGCCATCTTCTTCTCTGACCCCCTATCA
GCACCCTTAATCGTATTAACAACATGACTCCTCCCCCTAATACTAACAGCCAGCCAAGCCCA
CCTCTCCAATGAGCCCCTGGGCCTAAAAAAACTATACATTACCATACTAATCACCCTCCAAG
CACTACTAATCATAACATTCGCCTCCTCTGAGTTCATTATATTCTACATCCTGTTTGAAGCAA
CACTCGTACCAACCCTAATCATCATCACACGATGAGGAAACCAAGCAGAGCGACTAAACGC
AGGGTCCTACTTCCTCTTCTACACAATAGTAGGGTCACTCCCCCTTCTAGTAGTACTCACATA
CACCCAAAACATAACAGGAACCCTAAACATACTAGTACTACAATACTGAGCGAAACCCATA
AACGACTCTTGATCCAACATGCTAATATGACTAGCATGCATAATAGCATTCATAGTAAAAAT
ACCCCTATACGGACTACACCTCTGACTGCCAAAAGCCCACGTAGAGGCCCCCATTGCAGGCT
CCATAGTACTTGCAGCCGTACTACTAAAACTAGGCGGATACGGCATAATACGCATCACTATC
ATGCTAGAACCAATAACAACATTCATAGCATACCCCTTCCTAATACTATCCCTGTGAGGAAT
AATCATAACAAGCTCCATCTGCCTTCGCCAAACCGACCTAAAATCACTAATCGCCTACTCCT
CCGTAAGCCACATAGCACTAGTAATCGTCGCAATCCTAATCCAAACCCCATGAAGCTACATG
GGGGCCACCGCTCTCATAATCGCCCACGGTCTAACATCCTCCATACTATTTTGCCTAGCAAA
CACAAACTATGAGCGAACCCACAGCCGAACTATAATACTAGCACGAGGACTGCAAACCCTC
CTACCCCTAATAGCTGCCTGATGACTCCTAGCAAGCCTAACCAACCTGGCCCTCCCCCCCAG
CATCAACCTAATCGGAGAACTATTCGTAGTAGTATCAACATTCTCGTGGTCCAACACCACCA
TCATCCTTACTGGAACAAACATTATCATCACAGCCACCTACTCCCTATACATACTAATATCCA
CCCAACGTGGAAAATACACCCACCACATCAACAACATCTGCCCCTCCTTCACCCGAGAAAAC
GCTCTAATGGCACTCCACATACTGCCCCTACTAATGCTATCAACCAACCCCAAAATCATCCT
AGGGTGCCTTTACTGTAAGTATAGTTTAATAAAACCTCAGACTGTGGATCTGACAATAGAAG
ACCACAACTTCTTACTTACCAAAAAAGCATGCAAGAACTGCTAATTCCTGCCCCCATGTATA
AAAACATGGCTTTTTTAAACTTTTAGAGGATGACAGACACCCGTTGGTCTTAGGAACCAAAA
GACTTGGTGCAACTCCAAGTAAAAGTAATTAACACCCTAACCTGCACCTCCCTTATAACACT

AACAACACTAATCATACCAATCATAATCACCCCCACAAATGCCTACACAAGCAAAAACTAC
CCCCACCACGTAAAAAACATAGTCGCACTAGCCTTCGCCGTCAGCATAATCCCAGCAATAGC
ATTTATATACTCAAACGAAGAAATAACCATCTTCAACTGACACTGAACAACAATCCAAACAA
TAAAAATAACAATAAGCTTCAAACTAGACTATTTCTCCACAACATTCATACCAGTGGCCCTA
TTCGTCACATGGTCTATCATAGAATTCTCTCTATGATATATAAAATCAGACCCCCACATCAAC
CGATTCTTCAAGTACCTACTAATCTTCCTAATCACAATAATAATCCTAGTATCCGCCAACAAC
ATATTCCAACTCTTCATCGGCTGAGAAGGAGTAGGAATCATATCTTTCCTCCTAATCGGCTG
ATGGCACGGACGAACAGATGCAAACACAGCAGCCATACAGGCCATCCTATACAACCGCATC
GGAGACATTGGACTAATCCTGTCAATGGCATGATTCCTCACAAACCTAAACTCATGGGACCT
CCAACAAATCCTCATGCTAAACCCCGAGAACACAAACATCCCCCTAGCGGGCCTACTACTAG
CAGCAACTGGAAAATCCGCACAATTTGGGCTACACCCATGACTCCCCTCAGCCATAGAAGGC
CCAACCCCAGTATCAGCCCTACTCCACTCCAGCACAATAGTAGTCGCAGGGGTATTCCTACT
AATCCGATTCCACCCCCTAATAGAAAACAACAAAACAATCCAAACAACAACCCTATGCCTA
GGGGCCATTACAACCCTGTTCACAGCCATCTGTGCTCTCACCCAAAACGACATCAAAAAGAT
CGTGGCCTTCTCAACCTCAAGCCAACTAGGTCTAATAATAGTCACCATCGGAATCAACCAAC
CACACCTAGCCTTCATACACATCTGCACCCACGCCTTTTTCAAAGCCATACTATTCATGTGCT
CCGGATCGATCATCCACAGCCTAAACGACGAACAAGACATCCGCAAAATAGGAGGACTTTT
CCACGCCCTCCCCACTACCACCTCCGCTCTCATTATTGGCAGCCTAGCCCTAACAGGAACCC
CATTCCTAACAGGCTTCTACTCTAAAGACCTAATCATCGAAACCGCTAACATATCCCACACA
AACGCCTGAGCCCTGCTCATCACACTAGTAGCCACCTCCCTCACAGCCGCCTACAGCACACG
AATCATCTTCTTCACACTATTAGGACAACCCCGATCCAACACCCTAATCAACATCAACGAAA
ACAACCCCTCACTAACAAACCCAATCAAACGCCTAATGCTAGGGAGCATCTTCGCCGGATTC
CTACTATACAACAACATCCCCCCAACAACCATGCCCCAAACAACTATACCACAATACCTAAA
ATACACCGCCCTCGCCACTACAATCCTAGGCCTAATCATAGCCCTAGAACTGTGCAACCTGT
CCCTAAACCTAAGCCACAAACCCCCCTCAAGTGCCTTCAAATTCTCAACCTTACTAGGCTAC
TTCCCCACAATCATTCACCGCTCAGGACCCCTCTGATCACTAACAACAAGCCAAAAACTAGC
ATCCCTTATTCTAGACCTAATCTGACTAGAAAATATTATACCAAAGTCAATCTCACACTTCCA
CATAAAAGCCTCCCTAATAACATCCAACCAAAAAGGCTCAATCAAACTATACTTCCTGTCCT
TCGCAGTAACAATAATCCTCGCTCTCCTCATATTCTATTCCCCCGAGTAACCTCCATAACAAC
AACAACACAAATAAACAAAGACCACCCGGTCATAATCACAAACCAAGCACCATGGCTATAA
AGAGCAGCAACACCCGCAGCTTCCTCACTAAAAAAACTAGACCCCCCAACATCATAAGTCG
CCCAATCACCAGGACCACCAAGATCAAAAACCACACTCAACCCCTCACCCTTCAACATATAA
AGCACTAAAACCAACTCTATGACCACACCCAAAACAAAAGCACCGAGCACAACCACATTAG
ACACCCAAACTTCAGGATACTCCTCAATGGCTATGGCCGCCGTATAACCAAAAACAACCAAC
ATCCCCCCAAGATAAATCAAAAAAACCATTAAACCCAAAAAAGACCCACCAAAATTAACTA
CAACCCCACAACCTGCACCGCCAGCTACAACCAGACTAAACCCCCCATAAATAGGAGAAGG
CTTTGAAGAAAACCCTAAAAAACTAATCACAAAAATAACACTCAAAATAAACACAACATAT
GTCATTGTTCCCACATGGACTCAAACCATGACCTATGACACGAAAAATCATCGTTGTAATTA
AACTACGGAAACCTAATGACAAACATCCGAAAAACACACCCCCTACTCAAAATCATCAACA
ACTCCTTTATTGATCTCCCCACCCCCTCTAACATCTCAGCATGATGAAACTTCGGATCACTAC
TAGGAATCTGCTTAGTCCTACAAATTCTCACAGGACTATTTCTAGCCATACACTACACAGCA
GACACAGCAACCGCATTCTCATCAGTAACTCACATCTGCCGAGATGTAAACTACGGATGAAT
TATCCGATACATACACGCTAACGGAGCTTCCCTGTTCTTCATCTGCCTATTCGCACACATCGG
ACGAGGCATCTACTACGGATCCTTTGCCTACAAAGAGACATGAAACATCGGTATCCTGCTCC
TATTTGCAGTAATAGCAACAGCCTTTATGGGATACGTCCTACCATGAGGACAAATGTCCTTC
TGAGGTGCTACGGTAATTACAAACCTTTTATCCGCAATACCCTACATCGGGTCTAGCCTAGT
AGAGTGAGTCTGAGGGGGATTCTCGGTAGACAAAGCAACTCTCACTCGATTCTTCGCTCTTC
ACTTCATCCTTCCCTTCGTAATTCTTGCCCTAGTACTAGTACACTTACTATTCTTACACGAAA
CCGGATCCAACAACCCAATAGGAATCGTATCCAACCCCGACGTAATCCCCTTCCACCCATAC
TACACAGCCAAGGACACCCTTGGCCTATTCATCATGCTCACAGCACTAATATCCTTAGCCCT
ATTCTTCCCCGACCTACTAGGAGACCCAGACAATTATACACCCGCAAACCCCCTAAACACAC

CACCCCACATCAAACCAGAATGGTACTTCCTATTCGCATACGCAATCCTACGCTCAATTCCC
AACAAATTGGGAGGAGTACTAGCACTAATCCTATCCATCCTCGTACTAGCACTAATCCCACT
ACTACACACATCAAAACAACGAACTATAATGTTCCGACCCCTGAGCCAAACCATCTTCTGAC
TCCTGGTGGCCGACCTACTAGTTCTCACATGAATCGGAGGACAGCCCGTGGAACACCCCTTC
ATCCTAATCGGACAAGTGGCCTCTATCCTTTATTTCACACTAATCCTAGCAGCAATGCCAATC
GCAGGTATCATCGAAAACAATCTCATAAAATGAAGAGTCTTTGTAGTATATGTCAATACACT
GGTCTTGTAAACCAGCAAAGGAATTAACCCTCCCCAAGACTCAGGAAGAAGACAAAAGCCC
TACCATCAGCACCCAAAGCTGAAATTCTCAATAAACTACTTCCTGCAAACAATTAAAGTCCA
ACAAGCTTTATATAGTATATGCCATGTATAATCGTGCATTAATGGTTTGCCCCATGCATATAA
GCAGGTACATTATATTATTATAGTACATAGGACATATTATGTATAATCGTGCATTATTGATCT
AGTACATACATATAAGCAGGTACATTATATTATTATAGTACATAGGACATATTATGTATAAT
CGTGCATTATTGATCTAGTACATGCATATAAGCAGGTACATAATATCCTTAGAGTACATAGT
ACATATTATTATTGATCGGACATAGCACATCAAGTCAAATCATTTCCAGTCAACATGCGTAT
CCCTACCACTGAAGGCCGCCTAATCACCATGCCGCGTGAAATCATCAACCCGCTCATAGTCG
TGTCCCTCTTCTCGCTCCGGGCCCATACGGACTGTGGGGTAGTTAAAGTGAAACTATACCTG
GCATCTGGTTCTTACTTCATGTTCATCTTATATCTAGCCGCTCACTCGTTCCTCTTAAATAAGA
CATCTCGATGGATTAATTACTAATCAGCCCATGCCTAACATAACTGTGCTGTCATGCCTTTGG
TATCTTTTAATTTTCGGGGTGCGAGGTTCAACTGGGTCACCGACCTTCGAGCAGGTTGATAA
CTTGTAGATAGACATTCATTGAATATTATTGGTCGTACATACTAACTCCTAGGTGTTATTCAG
TCAATGGTTACAGGACATAGAGAATTTTACAACAAAATTTTGAGTTGAGCCTACAGAAATCA
TAGATCACAATCGCAGACACCAACAAATACTAAAGTAATTAATTTAAATTTATAATTTTAGG
TTTGATATATCAAACCCCCCCTTACCCCCCCAAAGCCTCCACGTACTAAACATCTTGTCAAAC
CCCAAAAGCAAGAATACACACGTACACAAAGGCGCTATAAGCACAAAGTATTACGTAAGTA
TATAGATATACAAAACTACTAGCGCAGCTTTCTGTCCACTAGTTAAATACTTAGGTGCTTGA
ACATATATGGCTGGAAGGCCATGTAGTGACTACGTAATGATTCCTATTAGCCACAATAAAAA
CAATATACAATAAATACAAATATAGATCAATTCTTATAGGCGCCGATAGCATAAACTATCTG
CCCCCTATGTACAATTGAATTAAA

>SRR6109207 (circular) ReOrganized
ATACTATAAATCCACCTCTCATTTATTCACTTCATACATGCTATTACACACTCTGTGCCATC
ATAGTATGTTTTCATACATCCTCCCTTCTTTCACACCCTATGTATATCGTACATTAATGGTGTA
CCCCCCCTCCCCCTATGTATATCGTGCATTAATGGCGTGCCCCATGCATATAAGCATGTACAT
ACTGTGCTTGGCTTTACATGAGGATACTCATTACAAGAACTTATTTCAAGCGATAGTCTATG
AGCATGTATTTCACTTAGTCCAAGAGCTTGATCACCAAGCCTCGAGAAACCAGCAATCCTTG
CGAGTACGTGTACCTCTTCTCGCTCCGGGCCCATAATTTGTGGGGGTTTCTATACTGAAACTA
TACCTGGCATCTGGTTCTTACCTCAGGGCCATGTTAGCGTCAACTCAATCCTACTAACCCTTC
AAATGGGACATCTCGATGGACTAATGACTAATCAGCCCATGATCACACATAACTGTGGTGTC
ATGCATTTGGTATTTTTTAATTTTAGGGGGGGAACTTGCTATGACTCAGCTATGACCGTAAAG
GTCTCGTCGCAGTCAAATCAGCTGTAGCTGGGCTTATTCATCTTTCGAGGCTCCTCATGGACA
CCCATAAGGTGCAATTCAGTCAATGGTCACAGGACATAACACTATAGATCACCCGGACTGGC
GTTACGTGTACGTACGTGTACGTACGTGTACGCACGTGTACGTACGTGTACGCACGTGTACG
TACGTGTACGTACGTGTACGCACGTGTACGCACGTGTACGCACGTGTACGCACGTGTACGCA
CGTGTACGTACGTGTACGCGTACGTATTTTAGATACTAAGTTAGCTTAGACAAACCCCCCTT
ACCCCCCGTAACTTCAAGAAGCTTACATATACTTATGGATGTCCTGCCAAACCCCAAAAACA
AGACTAAATATATGCGCAAACATGAAGTCACTTACACCTAAACCCATATAATTAAGCTAACC
CCCCAGCCAATGTTGCAACAACTACGGACATGGGACTCTAAATTTTAATTTATCTATAGATA
TTTTTCTTTTACTGTGTCTCCCCAGCATTGATTTTTTAATTATCATTATTCCACACCACCAATT
TCCATTGAGCTATTTCACATGAGTTCCAAATCAATTATGTTCATGTAGCTTAACGAATAAAGC
AAGGTACTGAAAATGCCTAGATGGGTCACGCTACCCCATAGACATAAAGGTTTGGTCCTAGC
CTTCCTATTAGCCATTAACAAGATTACACATGTAAGTCTCCACGCTCCAGTGAAAATGCCCC

TTAAGTCCTCTTAGACGACCTAAAGGAGCGGGTATCAAGCACACCTTATGGTAGCTCACAAC
GCCTTGCTTAGCCACACCCCCACGGGAAACAGCAGTGATAAAAATTAAGCTATGAACGAAA
GTTCGACTAAGCTATGTTAATACTAGGGTTGGTAAATCTCGTGCCAGCCACCGCGGTCATAC
GATTAACTCGAGTTAATAGGCCTACGGCGTAAAGCGTGTAAAAGAAAAAATCTCCTCTACTA
AAGTTAAAGTATGATTAAGCTGTAAAAAGCTACCATTAATACTAAAATAAACTACGAAAGT
GACTTTAAAATTTCTGATTACACGATAGCTAGGGCCCAAACTGGGATTAGATACCCCACTAT
GCCTAGCTCTAAACATAGATATTTTACTAAACAAAACTATTCGCCAGAGAACTACTAGCAAC
AGCTTAAAACTCAAAGGACTTGGCGGTGCTTTATATCCCCCTAGAGGAGCCTGTTCTGTAAT
CGATAAACCCCGATAGACCTCACCATCCCTTGCTAATTCAGTTTATATACCGCCATCTTCAGC
AAACCCTTAAAAGGAAAAAAAGTAAGCATAACTACCCTACATAAAAAAGTTAGGTCAAGGT
GTAACCTATGGGCTGGGAAGAAATGGGCTACATTTTCTATTCAAGAACAACTTCTACGAAAA
CTTTTATGAAACTAAAAGCTAAAGGCGGATTTAGTAGTAAATTAAGAATAGAGAGCTTAATT
GAACAGGGCAATGAAGCACGCACACACCGCCCGTCACCCTCCTCGAGTGATATAATTTAATT
ATAACCTATTTAAACTAAGCAAAGCATAAGAGGAGACAAGTCGTAACAAGGTAAGCATACT
GGAAAGTGTGCTTGGATGAGCCAAAGTGTAGCTTAAACAAAGCGTCTGGCTTACATCCAGA
AGATTTCATTAATATATGACTACTTTGAACCCAAAGCTAGCCCAAGCAACAATGACTAGTAA
AACCATTATGAAACATTCAAACAAAACATTTAGTAGCATGACTAGAGTATAGGAGATAGAA
ATTTTTAACTGGAGCTATAGAGAGAGTACCGCAAGGGAATGATGAAAGATTACCTAAAGTG
ATAAACAGCAAAGATTGCCCCTTCTACCTTTTGTATAATGAGTTAGCTAGAAATAACTTAAC
AAAGAGAACTTAAGCTAAGTCCCCCGAAACCAGACGAGCTACCTATGAACAATCCACTGGG
ATGAACTCATCTATGTTGCAAAATAGTGAGAAGATCCATAGGTAGAGGTGAAAGGCCTAAC
GAGCCTGGTGATAGCTGGTTGCCCAGAATAGAATTTTAGTTCGACTTTAAACCTGCCTACAA
AACTAATAATTCTAATGCAGATTTAAAATATATTCTAAAAAGGTACAGCTTTTTAGAGTTAA
GGATACAGCCTTACTTAGAGAGTAAATATTTATATAAGCCATAGTAGGCCTAGAGGCAGCCA
TCAATTAAGAAAGCGTTAAAGCTCAACATCTCTATTAACTTAATACCAAGAATATTTAATCA
ACTCCTAATGTATTACTGGGTCAATCTATTTAAATATAGAAGTGATAATGCTAATATGAGTA
ACAAGAAATATTTCTCCCATGCATAAGCTTATAACAGCAACGGATAACCACTGATAGTTAAC
AACAACATAGAAATAACCTAATGATAAAACACCTATTAAATCAATTGTTAGTCCAACACAG
GCATGCAATCAGGGAAAGATTAAAAGAAGTGAAAGGAACTCGGCAAATACAAACCCCGCCT
GTTTACCAAAAACATCACCTCCAGCATTTCCAGTATTGGAGGCACTGCCTGCCCGGTGACAT
CAGTTAAACGGCCGCGGTATTCTGACCGTGCAAAGGTAGCATAATCATTTGTTCTCTAAATA
AGGACTTGTATGAATGGCCACACGAGGGTTTAACTGTCTCTTACTTCCAATCAGTGAAATTG
ACCTCCCCGTGAAGAGGCGGGGATAAGACAATAAGACGAGAAGACCCTATGGAGCTTTAAT
TAACTAATTCAAAAAGAAACTACTAACGACCCAACAGGAATAATATATCTCTTTTATGAATT
AGCAATTTAGGTTGGGGCGACCTCGGAGGACAAAATAGCCTCCGAGTGATTATAAATCTAG
ACTTACCAGTCAAAATGCTTAATCACTTATTGATCCAAAAATTCTTTTGATCAACGGAACAA
GTTACCCTAGGGATAACAGCGCAATCCTATCCGAGAGTCCATATCGACAATAGGGTTTACGA
CCTCGATGTTGGATCAGGACATCCTAATGGTGCAGCAGCTATTAAAGGTTCGTTTGTTCAAC
GATTAAAGTCCTACGTGATCTGAGTTCAGACCGGAGCAATCCAGGTCGGTTTCTATCTATTC
AAATAATTTCTCCCAGTACGAAAGGACAAGAGAAATAAGGCCTACTTCTCTGAAGCGCCTTA
AGACCAATAGATGAATTTATCTAAATCTAGTAAATCTAACTCCAATATTGCCCAAGAGACAG
GGCTTTGTTAGGGTGGCAGAGCCCGGTAATTGTGCAAAACTTAAACTCTTGTGTCCAGAGGT
TCAATTCCTCTCCCTAGCATATGTTTATAATTAACATCTTCTCACTAATTGTACCTATTCTTCT
TGCTGTAGCCTTTCTGACTCTAGTAGAACGAAAAGTACTAGGCTACATACAGCTTCGAAAAG
GCCCAAATATTGTAGGTCCATATGGTCTCCTACAACCAATCGCAGATGCCGTAAAACTCTTT
ACTAAAGAACCTTTACGACCACTTACGTCATCCATAACTATATTTATTATAGCCCCTATTCTA
GCCTTAACACTAGCTCTGACCATGTGAGTCCCCTTACCAATACCGCACCCCCTTATTAACATA
AACCTAGGAGTACTATTCATACTAGCTATATCAAGTCTAGCTGTATACTCTATTCTCTGATCT
GGATGAGCCTCAAACTCAAAATATGCCCTAATTGGAGCCCTACGAGCCGTAGCTCAAACGAT
CTCATATGAAGTCACACTAGCCATTATCCTTTTATCCGTTCTACTAATAAATGGCTCATTTAC
GCTATCCACGCTAATCATTACCCAAGAACATCTTTGATTAATCTTCCCTGCATGACCACTAGC

TATAATATGATTTATTTCTACCCTAGCAGAAACAAATCGAGCCCCCTTTGACCTAACAGAAG
GAGAATCAGAACTAGTCTCTGGGTTTAATGTCGAATATGCAGCAGGTCCATTTGCCCTGTTC
TTCCTAGCAGAATATGCCAACATCATCATAATAAATGCTCTTACAACTATTCTATTCTTCGGA
GCATTTCACAGCCCCTACATACCAGAATTATACATTGTTAATTTTACCGTAAAAACTTTACTC
CTCACAATCTCTTTTCTATGAATCCGAGCATCATATCCTCGATTCCGATACGACCAACTAATA
CACCTACTATGAAAAAACTTTCTACCTCTCACACTAGCTCTATGTATATGACATGTAACTCTA
CCTATTATTACAGCAAGCATTCCACCTCAAACGTAAGAAATATGTCTGACAAAAGAATTACT
TTGATAGAGTAAGCCATAGGGGTTTAAGTCCCCTTATTTCTAGAATCATAGGAATCGAACCT
AATCTTAAGAACTCAAAAATCTTCGTGCTACCTAACTACACCATATCCTAAAGTAAGGTCAG
CTAAATAAGCTATCGGGCCCATACCCCGAAAATGTTGGTTTATCCCCTTCCCATACTAATAA
AACCAGCCATCCTTATTACCATTACATCCACCGTTGTCTTAGGAACTATAATTGTTCTATTTA
GCTCCCACTGATTCATAATCTGAGTTGGCTTTGAAATAAATATACTAGCCATTATCCCAATTC
TAATAAAAAAATATAACCCACGAGCCATAGAAGCTTCCACAAAATATTTCTAACACAAGCT
ACAGCATCCATGCTTCTAATGCTAAGCATCATCATCAATCTACTATGCTCAGGACACTGGAC
AGTTTCAACAATTCCCAATCCAGTAGCATCTACCATAATTACTATTGCTCTAACAATAAAACT
TGGCCTATCACCATTTCACTTTTGAGTCCCAGAAGTTACACAAGGCATTTCCTTATCTTCAGG
AATAATCCTATTAACATGACAAAAAATTGCACCTTTATCCATTCTATACCAAATTTCACCATC
CGTTAACTCAAATCTCTTACTAATAATAGCTATCACATCCATACTGGTAGGAGGCTGAGGAG
GATTAAATCAAACCCAACTCCGAAAAATCTTGGCATACTCCTCAATTACCCATATAGGCTGA
ATGGCTGCTATTATAGTATATAATCCTACCCTAGCAATTCTTAACCTAACAATTTACATCATA
ATAACTCTAGGAACATTTATACTATTCATACATAGCTCATCTACAACAACACTATCACTATCT
TATACATGAAATAAATTCCCATTAATAGCCCCACTAATCTTAATACTTATACTATCACTAGGA
GGCCTCCCTCCTCTCTCAGGCTTTATCCCCAAATGAATAATTATTCAAGAATTAACAAAAAA
CGACATAATTATTATACCTACACTTATAGCCATTACAGCCCTACTAAACCTATATTTTTACAC
ACGCCTAACATATACTACAGCACTAACTATATTTCCCGCAACAAATAATATAAAAATAAAAT
GACAATTCGAAACCACAAAAAAGACAACTCTCCTAGCCCCTCTAATCGTGACATCAACTATA
CTACTACCACTAACCCCAATACTAGCAGCTCTTGATTAGAGATTTAGGCTAAACAGACCAAG
GGCCTTCAAAGCCTCAAGTAAGTGAAAATCCACTTAATCTCTGCAATATTTAAGGACTGCAA
GAATCTACCTCACATCAACTGAACGCAAAACAATCGCTTTAATTAAGCTAAGCCCTTCTAGA
TCGGTGGGCTTTTACCCCACAAAACTTTAGTTAACAGCTAAAAACCCTAGACAACTGGCTTC
AATCTACTTCTCCCGCCGCGTAGGAAAAAAAGGCGGGAGAAGCCCCGGCAGAATTGAAGCT
GCTTCTTTGAATTTGCAATTCAATATGATTACTCACCACAGAGCCTGGTAAAAAGAGGACTT
AACCCCCATTCTTAGATTTACAGTCTAGTGCTTTTATCAGCCATTTTACCTATGTTCATTAACC
GATGACTGTTTTCCACCAACCACAAAGATATTGGCACTCTTTATCTTCTGTTTGGCGCATGAG
CTGGTATAACGGGCACTGCTCTTAGCCTTCTAATCCGTGCTGAATTAGGTCAGCCTGGAGCT
CTGTTAGGAGATGACCAAATTTACAATGTAGTTGTAACTGCCCACGCATTCGTAATAATCTT
CTTTATAGTTATGCCTATTATGATCGGAGGCTTTGGAAATTGACTAGTACCTTTGATAATTGG
TGCGCCTGATATAGCATTTCCCCGAATAAACAATATGAGTTTCTGATTACTACCTCCATCCTT
TCTTCTCCTTCTGGCTTCTTCCATAGTAGAAGCAGGCGCAGGGACTGGATGAACTGTATACC
CTCCTCTAGCAGGTAACTTAGCCCATGCAGGAGCATCAGTAGACCTAACAATCTTTTCTCTA
CATCTGGCAGGGGTTTCTTCTATTTTAGGGGCTATTAACTTTATTACCACTATCATCAACATG
AAACCCCCTGCAATATCTCAATATCAAACTCCCCTATTCGTGTGATCAGTACTTATCACAGCA
GTACTTCTCCTTCTATCTTTACCAGTCTTAGCAGCTGGAATTACTATACTACTTACAGATCGA
AATCTCAATACTACTTTCTTCGACCCGGCCGGAGGAGGGGATCCCATTCTATATCAACATCT
ATTTTGATTCTTTGGCCATCCTGAAGTCTATATTCTAATCCTCCCTGGGTTTGGAATAATTTCA
CACATTGTCACATACTATTCAGGAAAGAAAGAGCCCTTTGGTTATATGGGAATAGTGTGGGC
AATAATGTCAATTGGATTCTTAGGATTTATTGTATGGGCCCATCATATATTTACTGTAGGCAT
AGATGTTGATACACGAGCCTATTTTACCTCGGCCACTATAATTATTGCAATCCCGACAGGAG
TTAAAGTATTCAGCTGACTAGCTACCCTGCATGGAGGAAATATTAAGTGATCCCCTGCCATA
ATGTGAGCCCTAGGTTTCATTTTTCTATTTACAGTAGGGGGTCTTACAGGCATTGTTCTAGCT
AACTCATCTCTGGACATTGTCCTCCATGACACATACTACGTAGTAGCACACTTCCACTATGTA

CTATCAATAGGGGCCGTGTTCGCTATTATAGGAGGCTTCGTCCATTGATTCCCATTATTTTCG
GGCTATACACTTAATAATACATGAGCAAAAATTCACTTCGTAATTATATTTGTTGGAGTCAA
TATAACATTCTTCCCCCAGCACTTCCTAGGTCTATCAGGTATGCCTCGACGATATTCTGATTA
TCCAGACGCCTATACAGCATGAAACACAGTGTCTTCAATGGGTTCATTCATTTCACTAACAG
CAGTTATGTTAATAATTTTCATAATTTGAGAAGCCTTCGCATCTAAACGAGAAGTGGCAGTG
ATTGAGCTTACTACAACTAATATTGAATGGCTCCACGGATGTCCTCCCCCATACCATACATTC
GAAGAGCCCGCCCACGTCATACTGAAATAAGAAAGGAAGGAATCGAACCCCTCTAGAATTGG
TTTCAAGCCAGCATCATAACCACTATGTCTCTCTCAATAAAGAGGTATTAGTAAAAATTACA
TAACTTTGTCAAAGTTGAATTATAGGTGAAAATCCTTTATATCTCTATGGCGCATCCCTTTCA
AACAGGCCTTCAAGATGCAACTTCCCCCATCATGGAAGAACTCCTACACTTCCATGATCATA
CACTAATAATTGTATTCTTAATTAGTTCCTTAGTTCTCTATATCATTTCAATTATATTAACTAC
TAAATTAACACATACAAATACAATAGACGCACAAGAAGTGGAGACAGTATGAACTATTCTA
CCAGCTATCATCTTAATTATAATTGCACTTCCATCACTACGAATTCTTTATATGATAGATGAA
ATTAATAACCCATCATTAACTGTAAAGACTATGGGGCATCAATGATACTGAAGCTATGAGTA
TACAGATTATGAAGACTTGAGCTTCGATTCTTATATAACCCCAACACAAGAATTAAAGCCCG
GAGAGCTGCGATTACTAGAGGTAGATAATCGAGTAGTACTACCCATAGAAATAACAATTCG
CATATTAATTTCCTCAGAAGATGTGCTACATTCGTGAGCTGTCCCATCTCTAGGACTAAAAA
CTGATGCAATCCCGGGGCGATTAAATCAAACAACTCTTATGGCCATGCGACCAGGACTATAT
TATGGCCAATGCTCAGAGATTTGCGGTTCTAATCATAGCTTTATACCCATTGTACTTGAAATA
GTTCCGCTGTCTTACTTTGAAAAATGATCCGCTTCAATACTATAAAATCATTAAGAAGCTTTA
TAGCGTTAACCTTTTAAGTTAAAGACTGAGAGTGCAAGTCTCTCCTTAATGGAAATGCCACA
ATTAGACACATCCACATGATTTATTACAATCCTATCCATGATCTTAACACTATTTATTATATT
TCAGTTAAAAGTCTCAAAACACAACTATCCTGAGTTTCCTGAATTAAAAACAACCCCACTAT
CAAAAAAACCTATGCCTTGAGAAGAAAAATGAACGAAAATCTGTTCGCCTCTTTTACTACCC
CAATGATAATAGGAGTACCAATTGTGGTTCTGATTATTATTTTCCCAAGTATCTTATTTCCCT
CACCTAATCGATTAATCAATAATCGCCTAGCTACTGTCCAACAATGATTAATTCAATTAGTAT
CTAAGCATATACTGTCTATTCACAATTATAAAGGACAAACCTGAGCGCTAATACTAATGTCA
TTAATTTTATTTATTGGCTCAACTAACCTTTTAGGCCTATTACCACATTCATTCACACCTACTA
CACAACTATCAATAAATTTAGGAATGGCCATTCCTCTGTGAGCAGGTACAGTAGTTATAGGA
TTTCGACACAAAACTAAGGCATCTCTGGCCCACTTTCTACCCCAAGGAACACCTCTTCCTCTG
ATCCCAATGTTAGTAATTATCGAGACAATTAGTCTGTTTATTCAACCTGTGGCCCTAGCTGTA
CGACTGACCGCTAATATTACTGCAGGCCATCTACTTATTCATTTAATCGGAGGGGCTACCCT
AGTTTTAATAAACATTAACCCTGTAACAGCCCTAATTACCTTTATTATTCTTATTCTACTTACT
ATTCTCGAATTAGCTGTAGCTCTTATCCAAGCCTATGTTTTCACCCTACTAGTTAGTCTATAC
TTACATGATAATACCTAATGATCCACCAAACACATGCATATCATATAGTTAACCCAAGCCCA
TGGCCACTTACAGGAGCTCTCTCGGCTCTTCTTATAACATCAGGACTAATTATATGATTTCAC
TTTAACTCGGTATACTTACTAATACTAGGCCTCACCACCAACATACTTACCATGTACCAATGA
TGACGAGATATCGTCCGGGAAAGTACCTTCCAAGGCCATCACACCCCAATCGTCCAAAAAG
GACTACGATATGGAATAGTCTTATTTATTGTATCAGAAGTATTTTTCTTCGCGGGATTCTTTT
GGGCCTTCTACCACTCAAGCCTAGCACCTACTCCTGAATTAGGAGCATGTTGACCACCTACA
GGTATCATCCCCTTAAATCCGCTAGAAGTTCCACTACTTAACACCTCTGTACTTCTAGCATCT
GGGGTATCTATCACTTGAGCTCATCATAGTCTAATAGAAGGAGACCGCAAACATATGCTCCA
AGCCCTATTTATTACTATTTCACTAGGAGTTTATTTTACACTTCTACAAGCCTCAGAATACTA
TGAGACATCCTTTACTATTTCAGATGGAGTGTATGGTTCAACCTTCTTTATAGCAACTGGATT
TCATGGGCTTCATGTAATTATTGGTTCAACTTTTCTTACTGTGTGTTTTCTACGACAACTGCAC
TTTCACTTTACATCCAACCATCACTTTGGCTTTGAAGCGGCAGCATGATACTGACACTTCGTA
GACGTTGTATGGCTTTTCCTTTATGTTTCTATTTACTGATGAGGATCCTACTTCTTTAGTATTA
ACTAGTACGATTGACCTCCAATCAATAGGCTCTGGTACAAGCCAGAAAGAAGTAATAAACC
TAATTCTAGCACTTCTAACCAACGTATTATTAGCCTCTCTGCTCGTTCTTATTGCATTCTGACT
GCCACAACTAAATATCTATGCAGAAAAAGTAAGTCCTTATGAATGTGGATTTGATCCTATAG
GATCAGCACGCCTTCCTTTCTCTATAAAATTTTTTCTAGTAGCTATTACATTCCTTTTATTCGA

CTTAGAAATTGCTCTACTCCTCCCGCTTCCTTGAGCATTACAGACTAACAACTTAATAACTAT
ACTTATTATAGCTCTATTACTCATTTCCCTATTAGCCGCAAGCCTAGCTTATGAGTGAACCGA
AAAAGGGCTAGAATGGGCCGAATATGATAATTAGTTTAAACTAAAATAAGTGATTTCGACTC
ACTAGATTATGACTAACATCATAATTATCAAATGTCTGTGGTCTATATTAATCTCTTTCTAGC
CTTTACTATATCCTTAATAGGGCTACTCATCTATCGATCCCATTTAATATCCTCCCTATTATGC
TTAGAAGGCATGATGCTGTCACTATTTGTTATACTAGCAGTAACAATCCTAAGTAACCACTT
CACACTAGCCAGTATGACTCCAATCATTCTATTAGTATTCGCTGCCTGTGAAGCAGCGTTGG
GGCTTTCTCTGCTAGTAATAGTCTCTAATACCTATGGAACTGACTACGTACAAAATCTTAACC
TCCTACAATGCTAAAAATTATCCTCCCTACCATAATACTAGTACCCCTTACATGAATATCAAA
GCCTAACATAATCTGAATTAATACAACAACCTATAGTCTACTCATCAGCCTTATTAGTCTGAC
ATATCTCAATCAACTCAGTGATAATAGTCTAAACTTCTCATTAACATTCTTTACCGACTCCCT
ATCAGCCCCCCTGTTAGTTCTCACAACATGACTACTGCCCTTAATACTTATAGCTAGTCAATT
TCACCTGTCAAAAGAGACCCTGACCCGGAAAAAATTATATATCACAATACTAATTCTCCTGC
AACTATTCTTAATTATAACATTTTCTGCTACTGAGTTAATCATATTCTATATTTTATTTGAAGC
TACCCTGGTACCGACTTTAGTTATCATTACTCGATGAGGAAACCAAACAGAACGACTAAACG
CGGGCCTCTATTTTTTATTCTACACCTTAGTAGGATCCTTGCCCTTACTAGTTGCACTGTTATA
TGCCCAAAACAGCTTAGGATCACTAAATTTTCTCATAGTACAGTACTGAACTCAGCCCTTGC
CAAATTCCTGATCTAACATTTTCATGTGGCTGGCATGTATAATAGCATTCATAGTAAAAATA
CCGCTATATGGCTTACACCTATGACTACCTAAAGCACATGTAGAAGCTCCTATTGCAGGATC
CATAGTACTTGCTGCAGTACTTCTAAAACTAGGGGGATACGGTATAATGCGGATTACAACAA
TACTCAACCCTCTAACAAATTTTATAGCCTACCCCTTCATAATGCTCTCACTATGAGGAATAA
TCATAACAAGTTCTATTTGTTTACGCCAGACAGACCTAAAGTCCCTAATTGCATACTCCTCTG
TTAGTCACATAGCCCTAGTCATTGTAGCAATTCTAATCCAATCCCCATGAAGCTACATAGGA
GCTACTGCTCTAATAATCGCCCATGGGTTAACATCTTCTATGCTATTCTGTCTTGCAAACTCT
AACTACGAGCGAACCCATAGCCGAACTATAATTCTCGCACGGGGCTTGCAAACGCTTCTTCC
ACTTATAGCAGCTTGATGACTATTAGCAGGCCTAACTAACTTAGCTCTTCCTCCTACAATTAA
TCTGATTGGGGAACTGTTTGTAGTGATAGCCACATTTTCATGATCCAATATTACCATTCTCTT
AATAGGAGTTAACATTACCATTACTGCTCTCTACTCTCTATATATACTAATTTCTACACAGCG
AGGCAAGTATACAGACCACATCAAAAATATTAAGCCATCTTATACACGAGAAAATGCCTTA
ATAGCCTTACATTTACTACCCCTTTTACTTCTATCTCTTAACCCTAAAGTAGTTCTAGGCTTTA
TTTACTGTAAATATAGTTTAACAAAAACATTAGATTGTGAATCTAGTAATAAAAGCTTAAAT
CTTTTTATTTACCGAAAAAGCATGCAAGAACTGCTAATTCATGCACCCATGCATAAAATCAT
GGCTTTTTCAACTTTTAGGGGATAGAAGTAATCCATTGGTCTTAGGAGCCAAAAAATTGGTG
CAACTCCAAATAAAAGTAATTAACCTACTTACCTCCTTCACCATTACAACCCTATTTATATTA
ATCATGCCCATTATCCTAACCTGTACCCCAGCATATGAAAATAAACTTTACCCGCAATATGT
AAAAACCACAGTCTCTTATGCCTTCACAATTAGCATAATTCCCATAGTAATATTCATCCACTC
CGGACAAGAAGCAATCATCTCAAACTGACACTGAATAACCATCCAAACCCTAAAACTCAGT
CTAAGCTTCAAATTAGATTACTTTTCAATAATCTTCGTACCAGTGGCCCTATTCGTCACATGG
TCAATTATAGAATTCTCTATATGATATATACACGCAGATCCTAACATTAACCGATTCTTTAAA
TATCTACTTATATTCCTCGTCACGATAATAATTCTAGTAACCGCAAATAATCTATTTCAGCTA
TTTATCGGCTGAGAGGGAGTAGGAATCATATCATTTCTCCTCATTGGATGATGATATGGACG
AACAGATGCTAATACGGCTGCCCTACAAGCAGTACTATACAACCGCATTGGAGATGTTGGTT
TTATCATGGCCATAGCTTGATTCTTACTCAATACAAATGCATGGGACTTCCAACAAATCTTTA
TAATCGAGCATAAAAACCTCAATATTCCACTGGCAGGACTACTTCTAGCAGCTACCGGAAAG
TCTGCCCAATTTGGACTTCACCCATGACTCCCATCAGCCATAGAAGGCCCCACTCCTGTCTCA
GCACTACTTCACTCTAGCACAATAGTAGTAGCAGGCGTATTTCTTCTAGTCCGATTTCACCCC
TTAATAGAACATAACAAGACAATTCAAACGGTTACCCTATGCTTAGGGGCAATAACAACCTT
GTTTACAGCAATCTGCGCCCTTACCCAAAATGATATCAAAAAAATTATTGCCTTCTCCACCTC
GAGCCAACTAGGACTAATAATTGTAACAATTGGTATTAATCAGCCTCATCTGGCCTTTCTCC
ATATCTGCACGCACGCATTCTTCAAGGCCATGCTATTTCTATGCTCCGGATCTATTATTCATA
GTCTAAACAACGAACAAGATATTCGAAAGATAGGAGGGCTATTTAAAAGTCTACCATTTACC

ACTACTGCTCTAATCATCGGAACTCTAGCACTTACAGGAATGCCCTTCTTAACAGGATTTTAC
TCCAAAGACTTAATTATTGAAACCGCCAATACGTCATACACCAACGCCTGAGCCTTACTAAC
AACCCTTATCGCCACATCCATAACTGCCGCCTACAGTACTCGAATTGTATTTTTTACACTTCT
AGGACAACCTCGCTATAGTCCCATTATCACCATTAACGAGAATAACCAGTTCCTGACTAACT
CTGTCAAACGATTGCTACTAGGAAGTATCTTTGCGGGATTCCTAATCTCTCATAACATTACAC
CCACCACTGTCCCACAAATAACCATGCCCTGTCACCTAAAAATGATAGCCCTTACAGTAACA
ATCCTAGGCTTTATTCTAGCACTAGAACTTAACCTAGCCATACAAAATCTCAAATTTGACTAC
CCTTTAAGCACGTTCAAATTTTCAAATCAACTAGGATATTTCCCTACTACTCTGCATCGTCTC
GCACCAATAATAGGTCTATCAATAAGCCAAAAAACAGCATCCATACTGCTAGATGCAATCTG
ACTAGAGAATGTTCTACCAAAGTCCATCTCCTACTTTCAAATAAAATCCTCAATCACAGTCTC
CAACCAAAAGGGACTAATTAAACTATACTTTCTCTCCTTTATAATTACACTAATCTTAAGTCT
CTTTATACTTAATTTCCACGAGTGACTTCCATAATTACCAATACACCAATAAGGAGAGATCA
TCCAGTAACAATAACTAATCAAGTACCATAACTATATAAAGCTGCAATTCCCATAGCCTCTT
CACTAAAAAATCCTGAATCCCCTGTGTCATAAATCACCCAATCTCCTATTCCATTAAACTTAA
ATACTACCTCGGCTTCATCATCCTTTAAAATATAACAAACAGCCAGTAACTCAGACAACAGA
CCTACAATAAATGCACCTAGTACAGCCTTATTAGAAAGTCAAACCTCAGGATATTGCTCAGT
GGCTATGGCCGTAGTGTACCCGAACACTACAAGTATACCCCCTAAATAAATTAAAAAGACC
ATTAAACCCAAAAAAGATCCTCCAAAGCTTAACACAATACCACACCCTACACCCCCACTAAC
AATCAAGACTAAACCTCCGTAGATAGGAGAGGGCTTAGAAGAAAACCCCGCAAAACTAACT
ACAAAAACGATACTTAAAATAGATACAATGTATGTTATCATCATTCTTACATGGAATCTAAC
CATGACCAATGACATGAAAAATCATCGTTGTATTTCAACTATAAGAACCCCAATGATCAACA
TCCGAAAAACTCATCCATTAGTTAAAATTATCAACAACTCATTCATTGACCTTCCAACACCAT
CAAACATTTCAACATGATGGAACTTTGGGTCCCTGTTAGGAGTGTGTCTGATCTTGCAAATCT
TAACAGGCTTATTTCTAGCCATACACTATACATCAGATACAGCTACAGCCTTTTCATCAGTCG
CACACATTTGTCGAGACGTCAACTATGGTTGATTTATCCGATATATACATGCCAATGGGGCC
TCTATATTTTTTATCTGCCTATTTATACACGTAGGGCGAGGCTTATACTATGGATCATACCTA
TTTCCAGAGACATGGAATATCGGAATTATTCTCCTACTTACAGTTATAGCCACAGCATTCATA
GGGTATGTACTACCTTGAGGACAAATATCCTTCTGAGGAGCAACCGTCATTACTAACCTACT
ATCAGCAATTCCTTACATTGGCACTAATCTAGTGGAGTGAGTCTGAGGGGGTTTCTCCGTAG
ATAAAGCAACACTAACCCGATTTTTTGCTTTTCACTTTATCCTTCCATTTATCATCTCAGCACT
AGCAATAGTCCATCTATTATTCCTTCACGAAACAGGATCTAATAACCCCTCCGGAATTCCAT
CTGACCCAGACAAAATCCCATTTTACCCCTATCATACAATTAAAGACATCCTAGGCGTCCTA
TTTCTTGTCCTCGCCTTAATAACCCTGGCTTTATTCTCACCAGACCTGTTAGGAGACCCTGAT
AACTATACCCCTGCAAATCCACTAAGTACCCCGCCACATATTAAGCCTGAATGGTACTTTCT
ATTTGCCTACGCTATCCTGCGATCTATTCCTAATAAACTAGGAGGGGTGCTAGCTCTAATCTT
CTCTATTCTAATTCTAACTATTATTCCACTATTACATACATCCAAACAACGAAGCATGATATT
CCGACCTCTAAGTCAATGCTTATTCTGACTCCTAGTAGCAGACCTACTCACACTAACATGAA
TTGGAGGACAGCCAGTAGAACACCCCTTCATTATTATTGGGCAATTGGCCTCTATTCTCTACT
TTACAATTCTTCTAGTACTTATACCTATCACTAGCATTATTGAGAATAGCCTCTCAAAATGAA
GAGTCTTTGTAGTATATTAATTACTTTGGTCTTGTAAACCAAAAACGGAGATTCCTCTACTTC
TCCCTAAGACTCAAGGAAGGAGCAACAACCCCACTACCAGCACCCAAAGCTGGTGTTCTACT
TAAACTATCCCCTG
>SRR6109208 (circular) ReOrganized
ATACTATAAATCCACCTCTCATTTTATTCACTTCATACATGTTATTACACACTCTGTGCCATC
ATAGTATGTTTTCATACATCCTCCCTTCTTTCACACCCTATGTATATCGTACATTAATGGTGTA
CCCCCCCTCCCCCCTATGTATATCGTGCATTAATGGCGTGCCCCATGCATATAAGCATGTACA
TACTGTGCTTGGCTTTACATGAGGATACTCATTACAAGAACTTATTTCAAGCGATAGTCTATG
AGCATGTATTTCACTTAGTCCAAGAGCTTGATCACCAAGCCTCGAGAAACCAGCAATCCTTG
CGAGTACGTGTACCTCTTCTCGCTCCGGGCCCATAATTTGTGGGGGTTTCTATACTGAAACTA
TACCTGGCATCTGGTTCTTACCTCAGGGCCATGTTAGCGTCAACTCAATCCTACTAACCCTTC
AAATGGGACATCTCGATGGACTAATGACTAATCAGCCCATGATCACACATAACTGTGGTGTC

ATGCATTTGGTATTTTTTAATTTTAGGGGGGGAACTTGCTATGACTCAGCTATGACCGTAAAG
GTCTCGTCGCAGTCAAATCAGCTGTAGCTGGGCTTATTCATCTTTCGAGGCTCCTCATAGACA
CCCATAAGGTGCAATTCAGTCAATGGTCACAGGACATAACACTATAGATCACCCGGACTGGC
GTTACGTGTACGCACGTGTACGCACGTGTACGTACGTGTACGCACGTGTACGTACGTGTACG
CACGTGTACGTACGTGTACGCACGTGTACGTACGTGTACGCACGTGTACGTACGTGTACGCA
CGTGTACGCACGTGTACGCACGTGTACGTACGTGTACGCACGTGTACGTACGTGTACGCATG
TGTACGTACGTGTACGCACGTGTACGTACGTGTACGTACGTGTACGCACGTGTACGTACGTG
TACGCACGTGTACGCACGTGTACGTACGTGTACGCACGTGTACGCACGTGTACGCACGTGTA
CGCGTACGTATTTTAGATACTAAGTTAGCTTAGACAAACCCCCCTTACCCCCCGTAACTTCAA
GAAGCTTACATATACTTATGGATGTCCTGCCAAACCCCAAAAACAAGACTAAATATATGCGC
AAACATGAAGTCACTTACACCTAAACCCATATAATTAAGCTAACCCCCCAGCCAATGTTGCA
ACAACTACGGACATGGGACTCTAAATTTTAATTTATCTATAGATATTTTTCTTTTACTGTGTC
TCCCCAGCATTGATTTTTTAACTATCATTATTCCACACCACCAATTTCCATTGAGCTATTTCAC
ATGAGTTCCAAATCAATTATGTTCATGTAGCTTAACGAATAAAGCAAGGTACTGAAAATGCC
TAGATGGGTCACGCTACCCCATAGACATAAAGGTTTGGTCCTAGCCTTCCTATTAGCCATTA
ACAAGATTACACATGTAAGTCTCCACGCTCCAGTGAAAATGCCCCTTAAGTCCTCTTAGACG
ACCTAAAGGAGCGGGTATCAAGCACACCTTATGGTAGCTCACAACGCCTTGCTTAGCCACAC
CCCCACGGGAAACAGCAGTGATAAAAATTAAGCTATGAACGAAAGTTCGACTAAGCTATGT
TAATACTAGGGTTGGTAAATCTCGTGCCAGCCACCGCGGTCATACGATTAACTCGAGTTAAT
AGGCCTACGGCGTAAAGCGTGTAAAAGAAAAAATCTCCTCTACTAAAGTTAAAGTATGATT
AAGCTGTAAAAAGCTACCATTAATACTAAAATAAACTACGAAAGTGACTTTAAAATTTCTGA
TTACACGATAGCTAGGGCCCAAACTGGGATTAGATACCCCACTATGCCTAGCTCTAAACATA
GATATTTTACTAAACAAAACTATTCGCCAGAGAACTACTAGCAACAGCTTAAAACTCAAAGG
ACTTGGCGGTGCTTTATATCCCCCTAGAGGAGCCTGTTCTGTAATCGATAAACCCCGATAGA
CCTCACCATCCCTTGCTAATTCAGTTTATATACCGCCATCTTCAGCAAACCCTTAAAAGGAAA
AAAAGTAAGCATAACTACCCTACATAAAAAAGTTAGGTCAAGGTGTAACCTATGGGCTGGG
AAGAAATGGGCTACATTTTCTATTCAAGAACAACTTCTACGAAAACTTTTATGAAACTAAAA
GCTAAAGGCGGATTTAGTAGTAAATTAAGAATAGAGAGCTTAATTGAACAGGGCAATGAAG
CACGCACACACCGCCCGTCACCCTCCTCGAGTGATATAATTTAATTATAACCTATTTAAACTA
AGCAAAGCATAAGAGGAGACAAGTCGTAACAAGGTAAGCATACTGGAAAGTGTGCTTGGAT
GAGCCAAAGTGTAGCTTAAACAAAGCGTCTGGCTTACATCCAGAAGATTTCATTAATATATG
ACTACTTTGAACCCAAAGCTAGCCCAAGCAACAATGACTAGTAAAACCATTATGAAACATTC
AAACAAAACATTTAGTAGCATGACTAGAGTATAGGAGATAGAAATTTTTAACTGGAGCTAT
AGAGAGAGTACCGCAAGGGAATGATGAAAGATTACCTAAAGTGATAAACAGCAAAGATTGC
CCCTTCTACCTTTTGTATAATGAGTTAGCTAGAAATAACTTAACAAAGAGAACTTAAGCTAA
GTCCCCCGAAACCAGACGAGCTACCTATGAACAATCCACTGGGATGAACTCATCTATGTTGC
AAAATAGTGAGAAGATCCATAGGTAGAGGTGAAAGGCCTAACGAGCCTGGTGATAGCTGGT
TGCCCAGAATAGAATTTTAGTTCGACTTTAAACCTGCCTACAAAACTAATAATTCTAATGCA
GATTTAAAATATATTCTAAAAAGGTACAGCTTTTTAGAGTTAAGGATACAGCCTTACTTAGA
GAGTAAATATTTATATAAGCCATAGTAGGCCTAGAGGCAGCCATCAATTAAGAAAGCGTTA
AAGCTCAACATCTCTATTAACTTAATACCAAGAATATTTAATCAACTCCTAATGTATTACTGG
GTCAATCTATTTAAATATAGAAGTGATAATGCTAATATGAGTAACAAGAAATATTTCTCCCA
TGCATAAGCTTATAACAGCAACGGATAACCACTGATAGTTAACAACAACATAGAAATAACC
TAATGATAAAACACCTATTAAATCAATTGTTAGTCCAACACAGGCATGCAATCAGGGAAAG
ATTAAAAGAAGTGAAAGGAACTCGGCAAACACAAACCCCGCCTGTTTACCAAAAACATCAC
CTCCAGCATTTCCAGTATTGGAGGCACTGCCTGCCCGGTGACATCAGTTAAACGGCCGCGGT
ATTCTGACCGTGCAAAGGTAGCATAATCATTTGTTCTCTAAATAAGGACTTGTATGAACGGC
CACACGAGGGTTTAACTGTCTCTTACTTCCAATCAGTGAAATTGACCTCCCCGTGAAGAGGC
GGGGATAAGACAATAAGACGAGAAGACCCTATGGAGCTTTAATTAACTAATTCAAAAAGAA
ACTACTAACGACCCAACAGGAATAATATATCTCTTTTATGAATTAGCAATTTAGGTTGGGGC
GACCTCGGAGGACAAAATAGCCTCCGAGTGATTATAAATCTAGACTTACCAGTCAAAATGCT

TAATCACTTATTGATCCAAAAATTCTTTTGATCAACGGAACAAGTTACCCTAGGGATAACAG
CGCAATCCTATCCGAGAGTCCATATCGACAATAGGGTTTACGACCTCGATGTTGGATCAGGA
CATCCTAATGGTGCAGCAGCTATTAAAGGTTCGTTTGTTCAACGATTAAAGTCCTACGTGAT
CTGAGTTCAGACCGGAGCAATCCAGGTCGGTTTCTATCTATTCAAATAATTTCTCCCAGTACG
AAAGGACAAGAGAAATAAGGCCTACTTCTCTGAAGCGCCTTAAGACCAATAGATGAATTTA
TCTAAATCTAGTAAATCTAACTCCAATATTGCCCAAGAGACAGGGCTTTGTTAGGGTGGCAG
AGCCCGGTAATTGTGCAAAACTTAAACTCTTGTGTCCAGAGGTTCAATTCCTCTCCCTAGCAT
ATGTTTATAATTAACATCTTCTCACTAATTGTACCTATTCTTCTTGCTGTAGCCTTTCTGACTC
TAGTAGAACGAAAAGTACTAGGCTACATACAGCTTCGAAAAGGCCCAAATATTGTAGGTCC
ATATGGTCTCCTACAACCAATCGCAGATGCCGTAAAACTCTTTACTAAAGAACCTTTACGAC
CACTTACGTCATCCATAACTATATTTATTATAGCCCCTATTCTAGCCTTAACACTAGCTCTGA
CCATGTGAGTCCCCTTACCAATACCGCACCCCCTTATTAACATAAACCTAGGAGTACTATTC
ATACTAGCTATATCAAGTCTAGCTGTATACTCTATTCTCTGATCTGGATGAGCCTCAAACTCA
AAATATGCCCTAATTGGAGCCCTACGAGCCGTAGCTCAAACGATCTCATATGAAGTCACACT
AGCCATTATCCTTTTATCCGTTCTACTAATAAATGGCTCATTTACGCTATCCACGCTAATCAT
TACCCAAGAACATCTTTGATTAATCTTCCCTGCATGACCACTAGCTATAATATGATTTATTTC
TACCCTAGCAGAAACAAATCGAGCCCCCTTTGACCTAACAGAAGGAGAATCAGAACTAGTC
TCTGGGTTTAATGTCGAATATGCAGCAGGTCCATTTGCCCTGTTCTTCCTAGCAGAATATGCC
AACATCATCATAATAAATGCTCTTACAACTATTCTATTCTTCGGAGCATTTCACAGCCCCTAC
ATACCAGAATTATACATTGTTAATTTTACCGTAAAAACTTTACTCCTCACAATCTCTTTTCTA
TGAATCCGAGCATCATATCCTCGATTCCGATACGACCAACTAATACACCTACTATGAAAAAA
CTTTCTACCTCTCACACTAGCTCTATGTATATGACATGTAACTCTACCTATTATTACAGCAAG
CATTCCACCTCAAACGTAAGAAATATGTCTGACAAAAGAATTACTTTGATAGAGTAAGCCAT
AGGGGTTTAAGTCCCCTTATTTCTAGAATCATAGGAATCGAACCTAATCTTAAGAACTCAAA
AATCTTCGTGCTACCTAACTACACCATATCCTAAAGTAAGGTCAGCTAAATAAGCTATCGGG
CCCATACCCCGAAAATGTTGGTTTATCCCCTTCCCATACTAATAAAACCAGCCATCCTTATTA
CCATTATATCCACCGTTGTCTTAGGAACTATAATTGTTCTATTTAGCTCCCACTGATTCATAA
TCTGAGTTGGCTTTGAAATAAATATACTAGCCATTATCCCAATTCTAATAAAAAAATATAAC
CCACGAGCCATAGAAGCTTCCACAAAATATTTCTAACACAAGCTACAGCATCCATGCTTCT
AATGCTAAGCATCATCATCAATCTACTATGCTCAGGACACTGGACAGTTTCAACAATTCCCA
ATCCAGTAGCATCTACCATAATTACTATTGCTCTAACAATAAAACTTGGCCTATCACCATTTC
ACTTTTGAGTCCCAGAAGTTACACAAGGCATTTCCTTATCTTCAGGAATAATCCTATTAACAT
GACAAAAAATTGCACCTTTATCCATTCTATACCAAATTTCACCATCCGTTAACTCAAATCTCT
TACTAATAATAGCTATCACATCCATACTGGTAGGAGGCTGAGGAGGATTAAATCAAACCCA
ACTCCGAAAAATCTTGGCATACTCCTCAATTACCCATATAGGCTGAATGGCTGCTATTATAG
TATATAATCCTACCCTAGCAATTCTTAACCTAACAATTTACATCATAATAACTCTAGGAACAT
TTATACTATTCATACATAGCTCATCTACAACAACACTATCACTATCTTATACATGAAATAAAT
TCCCATTAATAGCCCCACTAATCTTAATACTTATACTATCACTAGGAGGCCTCCCTCCTCTCT
CAGGCTTTATCCCCAAATGAATAATTATTCAAGAATTAACAAAAAACGACATAATTATTATA
CCTACACTTATAGCCATTACAGCCCTACTAAACCTATATTTTTACACACGCCTAACATATACT
ACAGCACTAACTATATTTCCCGCAACAAATAATATAAAAATAAAATGACAATTCAAAACCA
CAAAAAAGACAACTCTCCTAGCCCCTCTAATCGTGACATCAACTATACTACTACCACTAACC
CCAATACTAGCAGCTCTTGATTAGAGATTTAGGCTAAACAGACCAAGGGCCTTCAAAGCCTC
AAGTAAGTGAAAATCCACTTAATCTCTGCAATATTTAAGGACTGCAAGAATCTATCTCACAT
CAACTGAACGCAAAACAATCGCTTTAATTAAGCTAAGCCCTTCTAGATCGGTGGGCTTTTAC
CCCACAAAACTTTAGTTAACAGCTAAAAACCCTAGACAACTGGCTTCAATCTACTTCTCCCG
CCGCGTAGGAAAAAAAGGCGGGAGAAGCCCCGGCAGAATTGAAGCTGCTTCTTTGAATTTG
CAATTCAATATGATTACTCACCACAGAGCCTGGTAAAAAGAGGACTTAACCCCCATTCTTAG
ATTTACAGTCTAGTGCTTTTATCAGCCATTTTACCTATGTTCATTAACCGATGACTGTTTTCCA
CCAACCACAAGGATATTGGCACTCTTTATCTTCTGTTTGGCGCATGAGCTGGTATAACGGGC
ACTGCTCTTAGCCTTCTAATCCGTGCTGAATTAGGTCAGCCTGGAGCTCTGTTAGGAGATGA

CCAAATTTACAATGTAGTTGTAACTGCCCACGCATTCGTAATAATCTTCTTTATAGTTATGCC
TATTATGATCGGAGGCTTTGGAAATTGACTAGTACCTTTGATAATTGGTGCGCCTGATATAG
CATTTCCCCGAATAAACAATATGAGTTTCTGATTACTACCTCCATCCTTTCTTCTCCTTCTGGC
TTCTTCCATAGTAGAAGCAGGCGCAGGGACTGGATGAACTGTATACCCCCCTCTAGCAGGTA
ACTTAGCCCATGCAGGAGCATCAGTAGACCTAACAATCTTTTCTCTACATCTGGCAGGGGTT
TCTTCTATTTTAGGGGCTATTAACTTTATTACCACTATCATCAACATGAAACCCCCTGCAATA
TCTCAATATCAAACTCCCCTATTCGTGTGATCAGTACTTATCACAGCAGTACTTCTCCTTCTA
TCTTTACCAGTCTTAGCAGCCGGAATTACTATACTACTTACAGATCGAAATCTCAATACTACT
TTCTTCGACCCGGCCGGAGGAGGGGATCCCATTCTATATCAACATCTATTTTGATTCTTTGGC
CATCCTGAAGTCTATATTCTAATCCTCCCTGGGTTTGGAATAATTTCACACATTGTCACATAC
TATTCAGGAAAGAAAGAGCCCTTTGGTTATATGGGAATAGTGTGGGCAATAATGTCAATTGG
GTTCTTAGGATTTATTGTATGGGCCCATCATATATTTACTGTAGGCATAGATGTTGATACACG
AGCCTATTTTACCTCGGCCACTATAATTATTGCAATCCCGACAGGAGTTAAAGTATTCAGCT
GACTAGCTACCCTGCATGGAGGAAATATTAAGTGATCCCCTGCCATAATGTGAGCCCTAGGT
TTCATTTTTCTATTTACAGTAGGGGGTCTTACAGGCATTGTTCTAGCTAACTCATCTCTGGAC
ATTGTCCTCCATGACACATACTACGTAGTAGCACACTTCCACTATGTACTATCAATAGGGGC
CGTGTTCGCTATTATAGGAGGCTTCGTCCATTGATTCCCATTATTTTCGGGCTATACACTTAA
TAATACATGAGCAAAAATTCACTTCGTAATTATATTTGTTGGAGTCAATATAACATTCTTCCC
CCAGCACTTCCTAGGTCTATCAGGTATGCCTCGACGATATTCTGATTATCCAGACGCCTATAC
AGCATGAAACACAGTGTCTTCAATGGGTTCATTCATTTCACTAACAGCAGTTATGTTAATAA
TTTTCATAATTTGAGAAGCCTTCGCATCTAAACGAGAAGTGGCAGTGATTGAGCTTACTACA
ACTAATATTGAATGGCTCCACGGATGTCCTCCCCCATACCATACATTCGAAGAGCCCGCCTA
CGTCATACTGAAATAAGAAAGGAAGGAATCGAACCCTCTAGAATTGGTTTCAAGCCAACAT
CATAACCACTATGTCTCTCTCAATAAAGAGGTATTAGTAAAAATTACATAACTTTGTCAAAG
TTGAATTATAGGTGAAAATCCTTTATATCTCTATGGCGCATCCCTTTCAAACAGGCCTTCAAG
ATGCAACTTCCCCCATCATGGAAGAACTCCTACACTTCCATGATCATACACTAATAATTGTAT
TCTTAATTAGTTCCTTAGTTCTCTATATCATTTCAATTATATTAACTACTAAATTAACACATAC
AAATACAATAGACGCACAAGAAGTGGAGACAGTATGAACTATTCTACCAGCTATCATCTTA
ATTATAATTGCACTTCCATCACTACGAATTCTTTATATGATAGATGAAATTAATAACCCATCA
TTAACTGTAAAGACTATGGGGCATCAATGATACTGAAGCTATGAGTATACAGATTATGAAGA
CTTGAGCTTCGATTCTTATATAACCCCAACACAAGAATTAAAGCCCGGAGAGCTGCGATTAC
TAGAGGTAGATAATCGAGTAGTACTACCCATAGAAATAACAATTCGCATATTAATTTCCTCA
GAAGATGTGCTACATTCGTGAGCTGTCCCATCTCTAGGACTAAAAACTGATGCAATCCCGGG
GCGATTAAATCAAACAACTCTTATGGCCATGCGACCAGGACTATATTATGGCCAATGCTCAG
AGATTTGCGGTTCTAATCATAGCTTTATACCCATTGTACTTGAAATAGTTCCGCTGTCTTACT
TTGAAAAATGATCCGCTTCAATACTATAAAATCATTAAGAAGCTTTATAGCGTTAACCTTTTA
AGTTAAAGACTGAGAGTGCAAGTCTCTCCTTAATGGAAATGCCACAATTAGACACATCCACA
TGATTTATTACAATCCTATCCATGATCTTAACACTATTTATTATATTTCAGTTAAAAGTCTCA
AAACACAACTATCCTGAGTTTCCTGAATTAAAAACAACCCCACTATCAAAAAAACCTATGCC
TTGAGAAGAAAAATGAACGAAAATCTGTTCGCCTCTTTTACTACCCCAATGATAATAGGAGT
ACCAATTGTGGTTCTGATTATTATTTTCCCAAGTATCTTATTTCCCTCACCTAATCGATTAATC
AATAACCGCCTAGCTACTGTCCAACAATGATTAATTCAATTAGTATCTAAGCATATACTGTCT
ATTCACAATTATAAAGGACAAACCTGAGCGCTAATACTAATGTCATTAATTTTATTTATTGGC
TCAACTAACCTTTTAGGCCTATTACCACATTCATTCACACCTACTACACAACTATCAATAAAT
TTAGGAATGGCCATTCCTCTGTGAGCAGGTACAGTAGTTATAGGATTTCGACACAAAACTAA
GGCATCTCTGGCCCACTTTCTACCTCAAGGAACACCTCTTCCTCTGATCCCAGTGTTAGTAAT
TATCGAGACAATTAGTCTGTTTATTCAACCTGTGGCCCTAGCTGTACGACTGACCGCTAATAT
TACTGCAGGCCATCTACTTATTCATTTAATCGGAGGGGCTACCCTAGTTTTAATAAACATTAA
CCCTGTAACAGCCCTAATTACCTTTATTATTCTTATTCTACTTACTATTCTCGAATTAGCTGTA
GCTCTTATCCAAGCCTATGTTTTCACCCTACTAGTTAGTCTATACTTACATGATAATACCTAA
TGATCCACCAAACACATGCATATCATATAGTTAACCCAAGCCCATGGCCACTTACAGGAGCT

CTCTCGGCTCTTCTTATAACATCAGGACTAATTATATGATTTCACTTTAACTCGGTATACTTA
CTAATACTAGGCCTCACCACCAACATACTTACCATGTACCAATGATGACGAGATATCGTCCG
GGAAAGTACCTTCCAAGGCCATCACACCCCAATCGTCCAAAAAGGACTACGATATGGAATA
GTCTTATTTATTGTATCAGAAGTATTTTTCTTCGCGGGATTCTTTTGGGCCTTCTACCACTCAA
GCCTAGCACCTACTCCTGAATTAGGAGCATGTTGACCACCTACAGGTATCATCCCCTTAAAT
CCGCTAGAAGTTCCACTACTTAACACCTCTGTACTTTTAGCATCTGGGGTATCTATCACTTGA
GCCCATCATAGTCTAATAGAAGGAGACCGTAAACATATGCTCCAAGCCCTATTTATTACTAT
TTCACTAGGAGTTTATTTTACACTTCTACAAGCCTCAGAATACTATGAGACATCCTTTACTAT
TTCAGATGGAGTGTATGGTTCAACCTTCTTTATAGCAACTGGATTTCATGGGCTTCATGTAAT
TATTGGTTCAACTTTTCTTACTGTGTGTTTTCTACGACAACTGCACTTTCACTTTACATCCAAC
CATCACTTTGGCTTTGAAGCGGCAGCATGATACTGACACTTCGTAGACGTTGTATGGCTTTTC
CTTTATGTTTCTATTTACTGATGAGGATCCTACTTCTTTAGTATTAACTAGTACGATTGACTTC
CAATCAATAGGCTCTGGTACAAGCCAGAAAGAAGTAATAAACCTAATTCTAGCACTTCTAAC
CAACGTATTATTAGCCTCTCTGCTCGTTCTTATTGCATTCTGACTGCCACAACTAAATATCTA
TGCAGAAAAAGTAAGTCCTTATGAATGTGGATTTGATCCTGTAGGATCAGCACGCCTTCCTT
TCTCTATAAAATTTTTTCTAGTAGCTATTACATTCCTTTTATTCGACTTAGAAATTGCTCTACT
CCTCCCGCTTCCTTGAGCATTACAGACTAACAACTTAATAACTATACTTATTATAGCTCTATT
ACTCATTTCCCTATTAGCCGCAAGCCTAGCTTATGAGTGAACCGAAAAGGGGCTAGAATGGG
CCGAATATGATAATTAGTTTAAACTAAAATAAGTGATTTCGACTCACTAGATTATGACTAAC
ATCATAATTATCAAATGTCTGTGGTCTATATTAATCTCTTTCTAGCCTTTACTATATCCTTAAT
AGGGCTACTCATCTACCGATCCCATTTAATATCCTCCCTATTATGCTTAGAAGGCATGATGCT
GTCACTATTTGTTATACTAACAGTAACAATCCTAAGTAACCACTTCACACTAGCCAGTATGA
CTCCAATCATTCTATTAGTATTCGCTGCCTGTGAAGCAGCGTTGGGGCTTTCTCTGCTAGTAA
TAGTCTCTAATACCTATGGAACTGACTACGTACAAAATCTTAACCTCCTACAATGCTAAAAA
TTATCCTCCCTACCATAATACTAGTACCCCTTACATGAATATCAAAGCCTAACATAATCTGAA
TTAATACAACAACCTATAGTCTACTCATCAGCCTTATTAGTCTGACATATCTCAATCAACTCA
GTGATAATAGTCTAAACTTCTCATTAACATTCTTTACCGACTCCCTATCAGCCCCCCTGTTAG
TTCTCACAACATGACTACTGCCCTTAATACTTATAGCTAGTCAATTTCACCTGTCAAAAGAGA
CCCTGACCCGGAAAAAATTATATATCACAATACTAATTCTCCTGCAACTATTCTTAATTATAA
CATTTTCTGCTACTGAGTTAATCATATTCTATATTTTATTTGAAGCTACCCTGGTACCGACTTT
AGTTATCATTACTCGATGAGGAAACCAAACAGAACGACTAAACGCGGGCCTCTATTTTTTAT
TCTACACCTTAGTAGGATCCTTGCCCTTACTAGTTGCACTGTTATATGCCCAAAACAGCTTAG
GATCACTAAATTTTCTCATAGTACAGTACTGAACTCAGCCCTTGCCAAATTCCTGATCTAACA
TTTTCATGTGGCTGGCATGTATAATAGCATTCATAGTAAAAATACCGCTATATGGCTTACACC
TATGACTACCCAAAGCACATGTAGAAGCTCCTATTGCAGGATCCATAGTACTTGCTGCAGTA
CTTCTAAAACTAGGGGGATACGGTATAATGCGGATTACAACAATACTCAACCCTCTAACAAA
TTTTATAGCCTACCCCTTCATAATGCTCTCACTATGAGGAATAATCATAACAAGTTCTATTTG
TTTACGCCAGACAGACCTAAAGTCCCTAATTGCATACTCCTCTGTTAGTCACATAGCCCTAGT
CATTGTAGCAATTCTAATCCAATCCCCATGAAGCTACATAGGAGCTACTGCTCTAATAATCG
CCCATGGGTTAACATCTTCTATGCTATTCTGTCTTGCAAACTCTAACTACGAGCGAACCCATA
GCCGAACTATAATTCTCGCACGGGGCTTGCAAACGCTTCTTCCACTTATAGCAGCTTGATGA
CTATTAGCAGGCCTAACTAACTTAGCTCTTCCTCCTACAATTAATCTGATTGGGGAACTGTTT
GTAGTGATAGCCACATTTTCATGATCCAATATTACCATTCTCTTAATAGGAGTTAACATTACC
ATTACTGCTCTCTACTCTCTATATATACTAATTTCTACACAGCGAGGCAAGTATACAGACCAC
ATCAAAAATATTAAGCCATCTTATACACGAGAAAATGCCTTAATAGCCTTACATTTACTACC
CCTTTTACTTCTATCTCTTAACCCTAAAGTAGTTCTAGGCTTTATTTACTGTAAATATAGTTTA
ACAAAAACATTAGATTGTGAATCTAGTAATAAAAGCTTAAATCTTTTTATTTACCGAAAAAG
CATGCAAGAACTGCTAATTCATGCACCCATGCATAAAATCATGGCTTTTTCAACTTTTAGGG
GATAGAAGTAATCCATTGGTCTTAGGAGCCAAAAAATTGGTGCAACTCCAAATAAAAGTAA
TTAACCTACTTACCTCCTTCACCATTACAACTCTATTTATATTAATCATGCCCATTATCCTAAC
CTGTACCCCAGCATATGAAAATAAACTTTACCCGCAATATGTAAAAACCACAGTCTCTTATG

CCTTCACAATTAGCATAATTCCCATAGTAATATTCATCCACTCCGGACAAGAAGCAATCATC
TCAAACTGACACTGAATAACCATCCAAACCCTAAAACTCAGTCTAAGCTTCAAATTAGATTA
CTTTTCAATAATCTTCGTACCAGTGGCCCTATTCGTCACATGGTCAATTATAGAATTCTCTAT
ATGATATATACACGCAGATCCTAACATTAACCGATTCTTTAAATATCTACTTATATTCCTCGT
CACGATAATAATTCTAGTAACCGCAAATAATCTATTTCAGCTATTCATCGGCTGAGAGGGAG
TAGGAATCATATCATTTCTCCTCATTGGATGATGATATGGACGAACAGATGCTAATACGGCT
GCCCTACAAGCAGTACTATACAACCGCATTGGAGATGTTGGTTTTATCATGGCCATAGCTTG
ATTCTTACTCAATACTAATGCATGGGACTTCCAACAAATCTTTATAATCGAACATAAAAACC
TCAATATTCCACTGGCAGGACTACTTCTAGCAGCTACCGGAAAGTCTGCCCAATTTGGACTT
CATCCATGACTCCCATCAGCCATAGAAGGCCCCACTCCTGTCTCAGCACTACTTCACTCTAGC
ACAATAGTAGTAGCAGGCGTATTTCTTCTAGTCCGGTTTCACCCCTTAATAGAACATAACAA
GACAATTCAAACGGTTACCCTATGCTTAGGGGCAATAACAACCTTGTTTACAGCAATCTGCG
CCCTTACCCAAAATGATATCAAAAAAATTATTGCCTTCTCCACCTCGAGCCAACTAGGACTA
ATAATTGTAACAATTGGTATTAATCAGCCTCATCTGGCCTTTCTCCATATCTGCACGCACGCA
TTCTTCAAGGCCATGCTATTTCTATGCTCCGGATCTATTATTCATAGTCTAAACAACGAACAA
GATATTCGAAAGATAGGAGGGCTATTTAAAAGTCTACCATTTACCACTACTGCTCTAATCAT
CGGAACTCTAGCACTTACAGGAATGCCCTTCTTAACAGGATTTTACTCCAAAGACTTAATTA
TTGAAACCGCCAATACGTCATACACCAACGCCTGAGCCTTACTAACAACCCTTATCGCCACA
TCCATAACTGCCGCCTACAGTACTCGAATTGTATTTTTTACACTTCTAGGACAACCTCGCTAT
AGTCCCATTATCACCATTAACGAGAATAACCAGTTCCTGACTAACTCTGTCAAACGATTGCT
ACTAGGAAGTATCTTTGCGGGATTCTTAATCTCTCATAACATTACACCCACCACTGTCCCACA
AATAACCATGCCCTGTCACCTAAAAATGATAGCCCTTACAGTAACAATCCTAGGCTTTATTC
TAGCACTAGAACTTAACCTAGCCATACAAAATCTCAAATTTGACTACCCGTTAAGCACGTTC
AAATTTTCAAATCAACTAGGATATTTCCCTACTACTCTGCATCGTCTCGCACCAATAATAGGT
CTATCAATAAGCCAAAAAACAGCATCCATACTGCTAGATGCAATCTGACTAGAGAATGTTCT
ACCAAAGTCCATCTCCTACTTTCAAATAAAATCCTCAATCACAGTCTCCAACCAAAAGGGAC
TAATTAAACTATACTTTCTCTCCTTTATAATTACACTAATCTTAAGTCTCTTTATACTTAATTT
CCACGAGTGACTTCCATAATTACCAATACACCAATAAGGAGAGATCATCCAGTAACAATAA
CTAATCAAGTACCATAACTATATAAAGCTGCAATTCCCATAGCCTCTTCACTAAAAAATCCT
GAATCCCCTGTATCATAAATCACCCAATCTCCTATTCCATTAAACTTAAATACTACCTCGGCT
TCATCATCCTTTAAAATATAACAAACAGCCAGTAACTCAGACAACAGACCTACAATAAATGC
ACCTAGTACAGCCTTATTAGAAAGTCAAACCTCAGGATATTGCTCAGTGGCTATGGCCGTAG
TGTACCCGAACACTACAAGTATACCCCCTAAATAAATTAAAAAGACCATTAAACCCAAAAA
AGATCCTCCAAAGCTTAACACAATACCACACCCTACACCCCCACTAACAATCAAGACTAAAC
CTCCGTAGATAGGAGAGGGCTTAGAAGAAAACCCCGCAAAACTAACTACAAAAACGATACT
TAAAATAGATACAATGTATGTTATCATCATTCTTACATGGAATCTAACCATGACCAATGACA
TGAAAAATCATCGTTGTATTTCAACTATAAGAACCCCAATGATCAACATCCGAAAAACTCAT
CCATTAGTTAAAATTATCAACAACTCATTCATTGACCTTCCAACACCATCAAACATTTCAACA
TGATGGAACTTTGGGTCCCTGTTAGGAGTGTGTCTGATCTTGCAAATCTTAACAGGCTTATTT
CTAGCCATACACTATACATCAGATACAGCTACAGCCTTTTCATCAGTCGCACACATTTGTCG
AGACGTCAACTATGGTTGATTTATCCGATATATACATGCCAATGGGGCCTCTATATTTTTTAT
CTGCCTATTTATACACGTAGGGCGAGGCTTATACTATGGATCATACCTATTTCCAGAGACAT
GGAATATCGGAATTATTCTCCTACTTACAGTTATAGCCACAGCATTCATAGGATATGTACTA
CCTTGAGGACAAATATCCTTCTGAGGAGCAACCGTCATTACTAACCTACTATCAGCAATTCC
TTACATTGGCACTAATCTAGTGGAGTGAATCTGAGGGGGTTTCTCCGTAGATAAAGCAACAC
TAACCCGATTTTTTGCTTTTCACTTTATCCTTCCATTTATCATCTCAGCACTAGCAATAGTCCA
TCTATTATTCCTTCACGAAACAGGATCTAATAACCCCTCCGGAATTCCATCTGACCCAGACA
AAATCCCATTTTACCCCTATCATACAATTAAAGACATCCTAGGCGTCCTATTTCTTGTCCTCG
CCTTAATAACCCTGGCTTTATTCTCACCAGACCTGTTAGGAGACCCTGATAACTATACCCCTG
CAAATCCACTAAGTACCCCGCCACATATTAAGCCTGAATGGTACTTTCTATTTGCCTACGCTA
TCCTGCGATCTATCCCTAATAAACTAGGAGGGGTGCTAGCTCTAATCTTCTCTATTCTAATTC

TAACTATTATTCCACTATTACATACATCCAAACAACGAAGCATGATATTCCGACCTCTAAGT
CAATGCTTATTCTGACTCCTAGTAGCAGACCTACTCACACTAACATGAATTGGAGGACAGCC
AGTAGAACACCCCTTCATTATTATTGGGCAATTGGCCTCTATTCTCTACTTTACAATTCTTCT
AGTACTTATACCTATCACTAGCATTATTGAGAATAGCCTCTCAAAATGAAGAGTCTTTGTAG
TATATTAATTACTTTGGTCTTGTAAACCAAAAACGGAGATTCCTCTACTTCTCCCTAAGACTC
AAGGAAGGAGCAACAACCCCACTACCAGCACCCAAAGCTGGTGTTCTACTTAAACTATCCCC
TG
>SRR6109209 (circular) ReOrganized
ATACTATAAATCCACCTCTCATTTTATTCACTTCATACATGTTATTACACACTCTGTGCCATC
ATAGTATGTTTTCATACATCCTCCCTTCTTTCACACCCTATGTATATCGTACATTAATGGTGTA
CCCCCCCCTCCCCCCTATGTATATCGTGCATTAATGGCGTGCCCCATGCATATAAGCATGTAC
ATACTGTGCTTGGCTTTACATGAGGATACTCATTACAAGAACTTATTTCAAGCGATAGTCTAT
GAGCATGTATTTCACTTAGTCCAAGAGCTTGATCACCAAGCCTCGAGAAACCAGCAATCCTT
GCGAGTACGTGTACCTCTTCTCGCTCCGGGCCCATAATTTGTGGGGGTTTCTATACTGAAACT
ATACCTGGCATCTGGTTCTTACCTCAGGGCCATGTTAGCGTCAACTCAATCCTACTAACCCTT
CAAATGGGACATCTCGATGGACTAATGACTAATCAGCCCATGATCACACATAACTGTGGTGT
CATGCATTTGGTATTTTTTAATTTTAGGGGGGGAACTTGCTATGACTCAGCTATGACCGTAAA
GGTCTCGTCGCAGTCAAATCAGCTGTAGCTGGGCTTATTCATCTTTCGAGGCTCCTCATAGAC
ACCCATAAGGTGCAATTCAGTCAATGGTCACAGGACATAACACTATAGATCACCCGGACTG
GCGTTACGTGTACGCACGTGTACGCACGTGTACGTACGTGTACGCACGTGTACGTACGTGTA
CGCATGTGTACGTACGTGTACGCACGTGTACGTACGTGTACGTACGTGTACGCACGTGTACG
CACGTGTACGCACGTGTACGCACGTGTACGCACGTGTACGTACGTGTACGCACGTGTACGCA
CGTGTACGCACGTGTACGCGTACGTATTTTAGATACTAAGTTAGCTTAGACAAACCCCCCTT
ACCCCCCGTAACTTCAAGAAGCTTACATATACTTATGGATGTCCTGCCAAACCCCAAAAACA
AGACTAAATATATGCGCAAACATGAAGTCACTTACACCTAAACCCATATAATTAAGCTAACC
CCCCAGCCAATGTTGCAACAACTACGGACATGGGACTCTAAATTTTAATTTATCTATAGATA
TTTTTCTTTTACTGTGTCTCCCCAGCATTGATTTTTTAACTATCATTATTCCACACCACCAATT
TCCATTGAGCTATTTCACATGAGTTCCAAATCAATTATGTTCATGTAGCTTAACGAATAAAGC
AAGGTACTGAAAATGCCTAGATGGGTCACGCTACCCCATAGACATAAAGGTTTGGTCCTAGC
CTTCCTATTAGCCATTAACAAGATTACACATGTAAGTCTCCACGCTCCAGTGAAAATGCCCC
TTAAGTCCTCTTAGACGACCTAAAGGAGCGGGTATCAAGCACACCTTATGGTAGCTCACAAC
GCCTTGCTTAGCCACACCCCCACGGGAAACAGCAGTGATAAAAATTAAGCTATGAACGAAA
GTTCGACTAAGCTATGTTAATACTAGGGTTGGTAAATCTCGTGCCAGCCACCGCGGTCATAC
GATTAACTCGAGTTAATAGGCCTACGGCGTAAAGCGTGTAAAAGAAAAAATCTCCTCTACTA
AAGTTAAAGTATGATTAAGCTGTAAAAAGCTACCATTAATACTAAAATAAACTACGAAAGT
GACTTTAAAATTTCTGATTACACGATAGCTAGGGCCCAAACTGGGATTAGATACCCCACTAT
GCCTAGCTCTAAACATAGATATTTTACTAAACAAAACTATTCGCCAGAGAACTACTAGCAAC
AGCTTAAAACTCAAAGGACTTGGCGGTGCTTTATATCCCCCTAGAGGAGCCTGTTCTGTAAT
CGATAAACCCCGATAGACCTCACCATCCCTTGCTAATTCAGTTTATATACCGCCATCTTCAGC
AAACCCTTAAAAGGAAAAAAAGTAAGCATAACTACCCTACATAAAAAAGTTAGGTCAAGGT
GTAACCTATGGGCTGGGAAGAAATGGGCTACATTTTCTATTCAAGAACAACTTCTACGAAAA
CTTTTATGAAACTAAAAGCTAAAGGCGGATTTAGTAGTAAATTAAGAATAGAGAGCTTAATT
GAACAGGGCAATGAAGCACGCACACACCGCCCGTCACCCTCCTCGAGTGATATAATTTAATT
ATAACCTATTTAAACTAAGCAAAGCATAAGAGGAGACAAGTCGTAACAAGGTAAGCATACT
GGAAAGTGTGCTTGGATGAGCCAAAGTGTAGCTTAAACAAAGCGTCTGGCTTACATCCAGA
AGATTTCATTAATATATGACTACTTTGAACCCAAAGCTAGCCCAAGCAACAATGACTAGTAA
AACCATTATGAAACATTCAAACAAAACATTTAGTAGCATGACTAGAGTATAGGAGATAGAA
ATTTTTAACTGGAGCTATAGAGAGAGTACCGCAAGGGAATGATGAAAGATTACCTAAAGTG
ATAAACAGCAAAGATTGCCCCTTCTACCTTTTGTATAATGAGTTAGCTAGAAATAACTTAAC
AAAGAGAACTTAAGCTAAGTCCCCCGAAACCAGACGAGCTACCTATGAACAATCCACTGGG
ATGAACTCATCTATGTTGCAAAATAGTGAGAAGATCCATAGGTAGAGGTGAAAGGCCTAAC

GAGCCTGGTGATAGCTGGTTGCCCAGAATAGAATTTTAGTTCGACTTTAAACCTGCCTACAA
AACTAATAATTCTAATGCAGATTTAAAATATATTCTAAAAAGGTACAGCTTTTTAGAGTTAA
GGATACAGCCTTACTTAGAGAGTAAATATTTATATAAGCCATAGTAGGCCTAGAGGCAGCCA
TCAATTAAGAAAGCGTTAAAGCTCAACATCTCTATTAACTTAATACCAAGAATATTTAATCA
ACTCCTAATGTATTACTGGGTCAATCTATTTAAATATAGAAGTGATAATGCTAATATGAGTA
ACAAGAAATATTTCTCCCATGCATAAGCTTATAACAGCAACGGATAACCACTGATAGTTAAC
AACAACATAGAAATAACCTAATGATAAAACACCTATTAAATCAATTGTTAGTCCAACACAG
GCATGCAATCAGGGAAAGATTAAAAGAAGTGAAAGGAACTCGGCAAACACAAACCCCGCCT
GTTTACCAAAAACATCACCTCCAGCATTTCCAGTATTGGAGGCACTGCCTGCCCGGTGACAT
CAGTTAAACGGCCGCGGTATTCTGACCGTGCAAAGGTAGCATAATCATTTGTTCTCTAAATA
AGGACTTGTATGAACGGCCACACGAGGGTTTAACTGTCTCTTACTTCCAATCAGTGAAATTG
ACCTCCCCGTGAAGAGGCGGGGATAAGACAATAAGACGAGAAGACCCTATGGAGCTTTAAT
TAACTAATTCAAAAAGAAACTACTAACGACCCAACAGGAATAATATATCTCTTTTATGAATT
AGCAATTTAGGTTGGGGCGACCTCGGAGGACAAAATAGCCTCCGAGTGATTATAAATCTAG
ACTTACCAGTCAAAATGCTTAATCACTTATTGATCCAAAAATTCTTTTGATCAACGGAACAA
GTTACCCTAGGGATAACAGCGCAATCCTATCCGAGAGTCCATATCGACAATAGGGTTTACGA
CCTCGATGTTGGATCAGGACATCCTAATGGTGCAGCAGCTATTAAAGGTTCGTTTGTTCAAC
GATTAAAGTCCTACGTGATCTGAGTTCAGACCGGAGCAATCCAGGTCGGTTTCTATCTATTC
AAATAATTTCTCCCAGTACGAAAGGACAAGAGAAATAAGGCCTACTTCTCTGAAGCGCCTTA
AGACCAATAGATGAATTTATCTAAATCTAGTAAATCTAACTCCAATATTGCCCAAGAGACAG
GGCTTTGTTAGGGTGGCAGAGCCCGGTAATTGTGCAAAACTTAAACTCTTGTGTCCAGAGGT
TCAATTCCTCTCCCTAGCATATGTTTATAATTAACATCTTCTCACTAATTGTACCTATTCTTCT
TGCTGTAGCCTTTCTGACTCTAGTAGAACGAAAAGTACTAGGCTACATACAGCTTCGAAAAG
GCCCAAATATTGTAGGTCCATATGGTCTCCTACAACCAATCGCAGATGCCGTAAAACTCTTT
ACTAAAGAACCTTTACGACCACTTACGTCATCCATAACTATATTTATTATAGCCCCTATTCTA
GCCTTAACACTAGCTCTGACCATGTGAGTCCCCTTACCAATACCGCACCCCCTTATTAACATA
AACCTAGGAGTACTATTCATACTAGCTATATCAAGTCTAGCTGTATACTCTATTCTCTGATCT
GGATGAGCCTCAAACTCAAAATATGCCCTAATTGGAGCCCTACGAGCCGTAGCTCAAACGAT
CTCATATGAAGTCACACTAGCCATTATCCTTTTATCCGTTCTACTAATAAATGGCTCATTTAC
GCTATCCACGCTAATCATTACCCAAGAACATCTTTGATTAATCTTCCCTGCATGACCACTAGC
TATAATATGATTTATTTCTACCCTAGCAGAAACAAATCGAGCCCCCTTTGACCTAACAGAAG
GAGAATCAGAACTAGTCTCTGGGTTTAATGTCGAATATGCAGCAGGTCCATTTGCCCTGTTC
TTCCTAGCAGAATATGCCAACATCATCATAATAAATGCTCTTACAACTATTCTATTCTTCGGA
GCATTTCACAGCCCCTACATACCAGAATTATACATTGTTAATTTTACCGTAAAAACTTTACTC
CTCACAATCTCTTTTCTATGAATCCGAGCATCATATCCTCGATTCCGATACGACCAACTAATA
CACCTACTATGAAAAAACTTTCTACCTCTCACACTAGCTCTATGTATATGACATGTAACTCTA
CCTATTATTACAGCAAGCATTCCACCTCAAACGTAAGAAATATGTCTGACAAAAGAATTACT
TTGATAGAGTAAGCCATAGGGGTTTAAGTCCCCTTATTTCTAGAATCATAGGAATCGAACCT
AATCTTAAGAACTCAAAAATCTTCGTGCTACCTAACTACACCATATCCTAAAGTAAGGTCAG
CTAAATAAGCTATCGGGCCCATACCCCGAAAATGTTGGTTTATCCCCTTCCCATACTAATAA
AACCAGCCATCCTTATTACCATTATATCCACCGTTGTCTTAGGAACTATAATTGTTCTATTTA
GCTCCCACTGATTCATAATCTGAGTTGGCTTTGAAATAAATATACTAGCCATTATCCCAATTC
TAATAAAAAAATATAACCCACGAGCCATAGAAGCTTCCACAAAATATTTCTAACACAAGCT
ACAGCATCCATGCTTCTAATGCTAAGCATCATCATCAATCTACTATGCTCAGGACACTGGAC
AGTTTCAACAATTCCCAATCCAGTAGCATCTACCATAATTACTATTGCTCTAACAATAAAACT
TGGCCTATCACCATTTCACTTTTGAGTCCCAGAAGTTACACAAGGCATTTCCTTATCTTCAGG
AATAATCCTATTAACATGACAAAAAATTGCACCTTTATCCATTCTATACCAAATTTCACCATC
CGTTAACTCAAATCTCTTACTAATAATAGCTATCACATCCATACTGGTAGGAGGCTGAGGAG
GATTAAATCAAACCCAACTCCGAAAAATCTTGGCATACTCCTCAATTACCCATATAGGCTGA
ATGGCTGCTATTATAGTATATAATCCTACCCTAGCAATTCTTAACCTAACAATTTACATCATA
ATAACTCTAGGAACATTTATACTATTCATACATAGCTCATCTACAACAACACTATCACTATCT

TATACATGAAATAAATTCCCATTAATAGCCCCACTAATCTTAATACTTATACTATCACTAGGA
GGCCTCCCTCCTCTCTCAGGCTTTATCCCCAAATGAATAATTATTCAAGAATTAACAAAAAA
CGACATAATTATTATACCTACACTTATAGCCATTACAGCCCTACTAAACCTATATTTTTACAC
ACGCCTAACATATACTACAGCACTAACTATATTTCCCGCAACAAATAATATAAAAATAAAAT
GACAATTCAAAACCACAAAAAAGACAACTCTCCTAGCCCCTCTAATCGTGACATCAACTATA
CTACTACCACTAACCCCAATACTAGCAGCTCTTGATTAGAGATTTAGGCTAAACAGACCAAG
GGCCTTCAAAGCCTCAAGTAAGTGAAAATCCACTTAATCTCTGCAATATTTAAGGACTGCAA
GAATCTATCTCACATCAACTGAACGCAAAACAATCGCTTTAATTAAGCTAAGCCCTTCTAGA
TCGGTGGGCTTTTACCCCACAAAACTTTAGTTAACAGCTAAAAACCCTAGACAACTGGCTTC
AATCTACTTCTCCCGCCGCGTAGGAAAAAAAGGCGGGAGAAGCCCCGGCAGAATTGAAGCT
GCTTCTTTGAATTTGCAATTCAATATGATTACTCACCACAGAGCCTGGTAAAAAGAGGACTT
AACCCCCATTCTTAGATTTACAGTCTAGTGCTTTTATCAGCCATTTTACCTATGTTCATTAACC
GATGACTGTTTTCCACCAACCACAAGGATATTGGCACTCTTTATCTTCTGTTTGGCGCATGAG
CTGGTATAACGGGCACTGCTCTTAGCCTTCTAATCCGTGCTGAATTAGGTCAGCCTGGAGCT
CTGTTAGGAGATGACCAAATTTACAATGTAGTTGTAACTGCCCACGCATTCGTAATAATCTT
CTTTATAGTTATGCCTATTATGATCGGAGGCTTTGGAAATTGACTAGTACCTTTGATAATTGG
TGCGCCTGATATAGCATTTCCCCGAATAAACAATATGAGTTTCTGATTACTACCTCCATCCTT
TCTTCTCCTTCTGGCTTCTTCCATAGTAGAAGCAGGCGCAGGGACTGGATGAACTGTATACC
CCCCTCTAGCAGGTAACTTAGCCCATGCAGGAGCATCAGTAGACCTAACAATCTTTTCTCTA
CATCTGGCAGGGGTTTCTTCTATTTTAGGGGCTATTAACTTTATTACCACTATCATCAACATG
AAACCCCCTGCAATATCTCAATATCAAACTCCCCTATTCGTGTGATCAGTACTTATCACAGCA
GTACTTCTCCTTCTATCTTTACCAGTCTTAGCAGCCGGAATTACTATACTACTTACAGATCGA
AATCTCAATACTACTTTCTTCGACCCGGCCGGAGGAGGGGATCCCATTCTATATCAACATCT
ATTTTGATTCTTTGGCCATCCTGAAGTCTATATTCTAATCCTCCCTGGGTTTGGAATAATTTCA
CACATTGTCACATACTATTCAGGAAAGAAAGAGCCCTTTGGTTATATGGGAATAGTGTGGGC
AATAATGTCAATTGGGTTCTTAGGATTTATTGTATGGGCCCATCATATATTTACTGTAGGCAT
AGATGTTGATACACGAGCCTATTTTACCTCGGCCACTATAATTATTGCAATCCCGACAGGAG
TTAAAGTATTCAGCTGACTAGCTACCCTGCATGGAGGAAATATTAAGTGATCCCCTGCCATA
ATGTGAGCCCTAGGTTTCATTTTTCTATTTACAGTAGGGGGTCTTACAGGCATTGTTCTAGCT
AACTCATCTCTGGACATTGTCCTCCATGACACATACTACGTAGTAGCACACTTCCACTATGTA
CTATCAATAGGGGCCGTGTTCGCTATTATAGGAGGCTTCGTCCATTGATTCCCATTATTTTCG
GGCTATACACTTAATAATACATGAGCAAAAATTCACTTCGTAATTATATTTGTTGGAGTCAA
TATAACATTCTTCCCCCAGCACTTCCTAGGTCTATCAGGTATGCCTCGACGATATTCTGATTA
TCCAGACGCCTATACAGCATGAAACACAGTGTCTTCAATGGGTTCATTCATTTCACTAACAG
CAGTTATGTTAATAATTTTCATAATTTGAGAAGCCTTCGCATCTAAACGAGAAGTGGCAGTG
ATTGAGCTTACTACAACTAATATTGAATGGCTCCACGGATGTCCTCCCCCATACCATACATTC
GAAGAGCCCGCCTACGTCATACTGAAATAAGAAAGGAAGGAATCGAACCCTCTAGAATTGG
TTTCAAGCCAACATCATAACCACTATGTCTCTCTCAATAAAGAGGTATTAGTAAAAATTACA
TAACTTTGTCAAAGTTGAATTATAGGTGAAAATCCTTTATATCTCTATGGCGCATCCCTTTCA
AACAGGCCTTCAAGATGCAACTTCCCCCATCATGGAAGAACTCCTACACTTCCATGATCATA
CACTAATAATTGTATTCTTAATTAGTTCCTTAGTTCTCTATATCATTTCAATTATATTAACTAC
TAAATTAACACATACAAATACAATAGACGCACAAGAAGTGGAGACAGTATGAACTATTCTA
CCAGCTATCATCTTAATTATAATTGCACTTCCATCACTACGAATTCTTTATATGATAGATGAA
ATTAATAACCCATCATTAACTGTAAAGACTATGGGGCATCAATGATACTGAAGCTATGAGTA
TACAGATTATGAAGACTTGAGCTTCGATTCTTATATAACCCCAACACAAGAATTAAAGCCCG
GAGAGCTGCGATTACTAGAGGTAGATAATCGAGTAGTACTACCCATAGAAATAACAATTCG
CATATTAATTTCCTCAGAAGATGTGCTACATTCGTGAGCTGTCCCATCTCTAGGACTAAAAA
CTGATGCAATCCCGGGGCGATTAAATCAAACAACTCTTATGGCCATGCGACCAGGACTATAT
TATGGCCAATGCTCAGAGATTTGCGGTTCTAATCATAGCTTTATACCCATTGTACTTGAAATA
GTTCCGCTGTCTTACTTTGAAAAATGATCCGCTTCAATACTATAAAATCATTAAGAAGCTTTA
TAGCGTTAACCTTTTAAGTTAAAGACTGAGAGTGCAAGTCTCTCCTTAATGGAAATGCCACA

ATTAGACACATCCACATGATTTATTACAATCCTATCCATGATCTTAACACTATTTATTATATT
TCAGTTAAAAGTCTCAAAACACAACTATCCTGAGTTTCCTGAATTAAAAACAACCCCACTAT
CAAAAAAACCTATGCCTTGAGAAGAAAAATGAACGAAAATCTGTTCGCCTCTTTTACTACCC
CAATGATAATAGGAGTACCAATTGTGGTTCTGATTATTATTTCCCAAGTATCTTATTTCCCT
CACCTAATCGATTAATCAATAACCGCCTAGCTACTGTCCAACAATGATTAATTCAATTAGTA
TCTAAGCATATACTGTCTATTCACAATTATAAAGGACAAACCTGAGCGCTAATACTAATGTC
ATTAATTTTATTTATTGGCTCAACTAACCTTTTAGGCCTATTACCACATTCATTCACACCTACT
ACACAACTATCAATAAATTTAGGAATGGCCATTCCTCTGTGAGCAGGTACAGTAGTTATAGG
ATTTCGACACAAAACTAAGGCATCTCTGGCCCACTTTCTACCTCAAGGAACACCTCTTCCTCT
GATCCCAGTGTTAGTAATTATCGAGACAATTAGTCTGTTTATTCAACCTGTGGCCCTAGCTGT
ACGACTGACCGCTAATATTACTGCAGGCCATCTACTTATTCATTTAATCGGAGGGGCTACCC
TAGTTTTAATAAACATTAACCCTGTAACAGCCCTAATTACCTTTATTATTCTTATTCTACTTAC
TATTCTCGAATTAGCTGTAGCTCTTATCCAAGCCTATGTTTTCACCCTACTAGTTAGTCTATA
CTTACATGATAATACCTAATGATCCACCAAACACATGCATATCATATAGTTAACCCAAGCCC
ATGGCCACTTACAGGAGCTCTCTCGGCTCTTCTTATAACATCAGGACTAATTATATGATTTCA
CTTTAACTCGGTATACTTACTAATACTAGGCCTCACCACCAACATACTTACCATGTACCAATG
ATGACGAGATATCGTCCGGGAAAGTACCTTCCAAGGCCATCACACCCCAATCGTCCAAAAA
GGACTACGATATGGAATAGTCTTATTTATTGTATCAGAAGTATTTTTCTTCGCGGGATTCTTT
TGGGCCTTCTACCACTCAAGCCTAGCACCTACTCCTGAATTAGGAGCATGTTGACCACCTAC
AGGTATCATCCCCTTAAATCCGCTAGAAGTTCCACTACTTAACACCTCTGTACTTTTAGCATC
TGGGGTATCTATCACTTGAGCCCATCATAGTCTAATAGAAGGAGACCGTAAACATATGCTCC
AAGCCCTATTTATTACTATTTCACTAGGAGTTTATTTTACACTTCTACAAGCCTCAGAATACT
ATGAGACATCCTTTACTATTTCAGATGGAGTGTATGGTTCAACCTTCTTTATAGCAACTGGAT
TTCATGGGCTTCATGTAATTATTGGTTCAACTTTTCTTACTGTGTGTTTTCTACGACAACTGCA
CTTTCACTTTACATCCAACCATCACTTTGGCTTTGAAGCGGCAGCATGATACTGACACTTCGT
AGACGTTGTATGGCTTTTCCTTTATGTTTCTATTTACTGATGAGGATCCTACTTCTTTAGTATT
AACTAGTACGATTGACTTCCAATCAATAGGCTCTGGTACAAGCCAGAAAGAAGTAATAAAC
CTAATTCTAGCACTTCTAACCAACGTATTATTAGCCTCTCTGCTCGTTCTTATTGCATTCTGAC
TGCCACAACTAAATATCTATGCAGAAAAAGTAAGTCCTTATGAATGTGGATTTGATCCTGTA
GGATCAGCACGCCTTCCTTTCTCTATAAAATTTTTTCTAGTAGCTATTACATTCCTTTTATTCG
ACTTAGAAATTGCTCTACTCCTCCCGCTTCCTTGAGCATTACAGACTAACAACTTAATAACTA
TACTTATTATAGCTCTATTACTCATTTCCCTATTAGCCGCAAGCCTAGCTTATGAGTGAACCG
AAAAGGGGCTAGAATGGGCCGAATATGATAATTAGTTTAAACTAAAATAAGTGATTTCGAC
TCACTAGATTATGACTAACATCATAATTATCAAATGTCTGTGGTCTATATTAATCTCTTTCTA
GCCTTTACTATATCCTTAATAGGGCTACTCATCTACCGATCCCATTTAATATCCTCCCTATTAT
GCTTAGAAGGCATGATGCTGTCACTATTTGTTATACTAACAGTAACAATCCTAAGTAACCAC
TTCACACTAGCCAGTATGACTCCAATCATTCTATTAGTATTCGCTGCCTGTGAAGCAGCGTTG
GGGCTTTCTCTGCTAGTAATAGTCTCTAATACCTATGGAACTGACTACGTACAAAATCTTAAC
CTCCTACAATGCTAAAAATTATCCTCCCTACCATAATACTAGTACCCCTTACATGAATATCAA
AGCCTAACATAATCTGAATTAATACAACAACCTATAGTCTACTCATCAGCCTTATTAGTCTG
ACATATCTCAATCAACTCAGTGATAATAGTCTAAACTTCTCATTAACATTCTTTACCGACTCC
CTATCAGCCCCCCTGTTAGTTCTCACAACATGACTACTGCCCTTAATACTTATAGCTAGTCAA
TTTCACCTGTCAAAAGAGACCCTGACCCGGAAAAAATTATATATCACAATACTAATTCTCCT
GCAACTATTCTTAATTATAACATTTTCTGCTACTGAGTTAATCATATTCTATATTTTATTTGAA
GCTACCCTGGTACCGACTTTAGTTATCATTACTCGATGAGGAAACCAAACAGAACGACTAAA
CGCGGGCCTCTATTTTTTATTCTACACCTTAGTAGGATCCTTGCCCTTACTAGTTGCACTGTTA
TATGCCCAAAACAGCTTAGGATCACTAAATTTTCTCATAGTACAGTACTGAACTCAGCCCTT
GCCAAATTCCTGATCTAACATTTTCATGTGGCTGGCATGTATAATAGCATTCATAGTAAAAA
TACCGCTATATGGCTTACACCTATGACTACCCAAAGCACATGTAGAAGCTCCTATTGCAGGA
TCCATAGTACTTGCTGCAGTACTTCTAAAACTAGGGGGATACGGTATAATGCGGATTACAAC
AATACTCAACCCTCTAACAAATTTTATAGCCTACCCCTTCATAATGCTCTCACTATGAGGAAT

AATCATAACAAGTTCTATTTGTTTACGCCAGACAGACCTAAAGTCCCTAATTGCATACTCCTC
TGTTAGTCACATAGCCCTAGTCATTGTAGCAATTCTAATCCAATCCCCATGAAGCTACATAG
GAGCTACTGCTCTAATAATCGCCCATGGGTTAACATCTTCTATGCTATTCTGTCTTGCAAACT
CTAACTACGAGCGAACCCATAGCCGAACTATAATTCTCGCACGGGGCTTGCAAACGCTTCTT
CCACTTATAGCAGCTTGATGACTATTAGCAGGCCTAACTAACTTAGCTCTTCCTCCTACAATT
AATCTGATTGGGGAACTGTTTGTAGTGATAGCCACATTTTCATGATCCAATATTACCATTCTC
TTAATAGGAGTTAACATTACCATTACTGCTCTCTACTCTCTATATATACTAATTTCTACACAG
CGAGGCAAGTATACAGACCACATCAAAAATATTAAGCCATCTTATACACGAGAAAATGCCT
TAATAGCCTTACATTTACTACCCCTTTTACTTCTATCTCTTAACCCTAAAGTAGTTCTAGGCTT
TATTTACTGTAAATATAGTTTAACAAAAACATTAGATTGTGAATCTAGTAATAAAAGCTTAA
ATCTTTTTATTTACCGAAAAAGCATGCAAGAACTGCTAATTCATGCACCCATGCATAAAATC
ATGGCTTTTTCAACTTTTAGGGGATAGAAGTAATCCATTGGTCTTAGGAGCCAAAAAATTGG
TGCAACTCCAAATAAAAGTAATTAACCTACTTACCTCCTTCACCATTACAACTCTATTTATAT
TAATCATGCCCATTATCCTAACCTGTACCCCAGCATATGAAAATAAACTTTACCCGCAATAT
GTAAAAACCACAGTCTCTTATGCCTTCACAATTAGCATAATTCCCATAGTAATATTCATCCAC
TCCGGACAAGAAGCAATCATCTCAAACTGACACTGAATAACCATCCAAACCCTAAAACTCA
GTCTAAGCTTCAAATTAGATTACTTTTCAATAATCTTCGTACCAGTGGCCCTATTCGTCACAT
GGTCAATTATAGAATTCTCTATATGATATATACACGCAGATCCTAACATTAACCGATTCTTTA
AATATCTACTTATATTCCTCGTCACGATAATAATTCTAGTAACCGCAAATAATCTATTTCAGC
TATTCATCGGCTGAGAGGGAGTAGGAATCATATCATTTCTCCTCATTGGATGATGATATGGA
CGAACAGATGCTAATACGGCTGCCCTACAAGCAGTACTATACAACCGCATTGGAGATGTTGG
TTTTATCATGGCCATAGCTTGATTCTTACTCAATACTAATGCATGGGACTTCCAACAAATCTT
TATAATCGAACATAAAAACCTCAATATTCCACTGGCAGGACTACTTCTAGCAGCTACCGGAA
AGTCTGCCCAATTTGGACTTCATCCATGACTCCCATCAGCCATAGAAGGCCCCACTCCTGTCT
CAGCACTACTTCACTCTAGCACAATAGTAGTAGCAGGCGTATTTCTTCTAGTCCGGTTTCACC
CCTTAATAGAACATAACAAGACAATTCAAACGGTTACCCTATGCTTAGGGGCAATAACAACC
TTGTTTACAGCAATCTGCGCCCTTACCCAAAATGATATCAAAAAAATTATTGCCTTCTCCACC
TCGAGCCAACTAGGACTAATAATTGTAACAATTGGTATTAATCAGCCTCATCTGGCCTTTCTC
CATATCTGCACGCACGCATTCTTCAAGGCCATGCTATTTCTATGCTCCGGATCTATTATTCAT
AGTCTAAACAACGAACAAGATATTCGAAAGATAGGAGGGCTATTTAAAAGTCTACCATTTA
CCACTACTGCTCTAATCATCGGAACTCTAGCACTTACAGGAATGCCCTTCTTAACAGGATTTT
ACTCCAAAGACTTAATTATTGAAACCGCCAATACGTCATACACCAACGCCTGAGCCTTACTA
ACAACCCTTATCGCCACATCCATAACTGCCGCCTACAGTACTCGAATTGTATTTTTTACACTT
CTAGGACAACCTCGCTATAGTCCCATTATCACCATTAACGAGAATAACCAGTTCCTGACTAA
CTCTGTCAAACGATTGCTACTAGGAAGTATCTTTGCGGGATTCTTAATCTCTCATAACATTAC
ACCCACCACTGTCCCACAAATAACCATGCCCTGTCACCTAAAAATGATAGCCCTTACAGTAA
CAATCCTAGGCTTTATTCTAGCACTAGAACTTAACCTAGCCATACAAAATCTCAAATTTGACT
ACCCGTTAAGCACGTTCAAATTTTCAAATCAACTAGGATATTTCCCTACTACTCTGCATCGTC
TCGCACCAATAATAGGTCTATCAATAAGCCAAAAAACAGCATCCATACTGCTAGATGCAATC
TGACTAGAGAATGTTCTACCAAAGTCCATCTCCTACTTTCAAATAAAATCCTCAATCACAGT
CTCCAACCAAAAGGGACTAATTAAACTATACTTTCTCTCCTTTATAATTACACTAATCTTAAG
TCTCTTTATACTTAATTTCCACGAGTGACTTCCATAATTACCAATACACCAATAAGGAGAGAT
CATCCAGTAACAATAACTAATCAAGTACCATAACTATATAAAGCTGCAATTCCCATAGCCTC
TTCACTAAAAAATCCTGAATCCCCTGTATCATAAATCACCCAATCTCCTATTCCATTAAACTT
AAATACTACCTCGGCTTCATCATCCTTTAAAATATAACAAACAGCCAGTAACTCAGACAACA
GACCTACAATAAATGCACCTAGTACAGCCTTATTAGAAAGTCAAACCTCAGGATATTGCTCA
GTGGCTATGGCCGTAGTGTACCCGAACACTACAAGTATACCCCCTAAATAAATTAAAAAGAC
CATTAAACCCAAAAAAGATCCTCCAAAGCTTAACACAATACCACACCCTACACCCCCACTAA
CAATCAAGACTAAACCTCCGTAGATAGGAGAGGGCTTAGAAGAAAACCCCGCAAAACTAAC
TACAAAAACGATACTTAAAATAGATACAATGTATGTTATCATCATTCTTACATGGAATCTAA
CCATGACCAATGACATGAAAAATCATCGTTGTATTTCAACTATAAGAACCCCAATGATCAAC

ATCCGAAAAACTCATCCATTAGTTAAAATTATCAACAACTCATTCATTGACCTTCCAACACC
ATCAAACATTTCAACATGATGGAACTTTGGGTCCCTGTTAGGAGTGTGTCTGATCTTGCAAA
TCTTAACAGGCTTATTTCTAGCCATACACTATACATCAGATACAGCTACAGCCTTTTCATCAG
TCGCACACATTTGTCGAGACGTCAACTATGGTTGATTTATCCGATATATACATGCCAATGGG
GCCTCTATATTTTTTATCTGCCTATTTATACACGTAGGGCGAGGCTTATACTATGGATCATAC
CTATTTCCAGAGACATGGAATATCGGAATTATTCTCCTACTTACAGTTATAGCCACAGCATTC
ATAGGATATGTACTACCTTGAGGACAAATATCCTTCTGAGGAGCAACCGTCATTACTAACCT
ACTATCAGCAATTCCTTACATTGGCACTAATCTAGTGGAGTGAATCTGAGGGGGTTTCTCCG
TAGATAAAGCAACACTAACCCGATTTTTTGCTTTTCACTTTATCCTTCCATTTATCATCTCAGC
ACTAGCAATAGTCCATCTATTATTCCTTCACGAAACAGGATCTAATAACCCCTCCGGAATTC
CATCTGACCCAGACAAAATCCCATTTTACCCCTATCATACAATTAAAGACATCCTAGGCGTC
CTATTTCTTGTCCTCGCCTTAATAACCCTGGCTTTATTCTCACCAGACCTGTTAGGAGACCCT
GATAACTATACCCCTGCAAATCCACTAAGTACCCCGCCACATATTAAGCCTGAATGGTACTT
TCTATTTGCCTACGCTATCCTGCGATCTATCCCTAATAAACTAGGAGGGGTGCTAGCTCTAAT
CTTCTCTATTCTAATTCTAACTATTATTCCACTATTACATACATCCAAACAACGAAGCATGAT
ATTCCGACCTCTAAGTCAATGCTTATTCTGACTCCTAGTAGCAGACCTACTCACACTAACATG
AATTGGAGGACAGCCAGTAGAACACCCCTTCATTATTATTGGGCAATTGGCCTCTATTCTCT
ACTTTACAATTCTTCTAGTACTTATACCTATCACTAGCATTATTGAGAATAGCCTCTCAAAAT
GAAGAGTCTTTGTAGTATATTAATTACTTTGGTCTTGTAAACCAAAAACGGAGATTCCTCTAC
TTCTCCCTAAGACTCAAGGAAGGAGCAACAACCCCACTACCAGCACCCAAAGCTGGTGTTCT
ACTTAAACTATCCCCTG
>SRR6109210 AssemblyGraph Reorganized
ATACTATAAATCCACCTCTCATTTTATTCACTTCATACATGCTATTACACACTCTGTGCCATC
ATAGTATGTTTTCATACATCCTCCCTTCTTTCACACCCTATGTATATCGTACATTAATGGTGTA
CCCCCCCCTCCCCCCTATGTATATCGTGCATTAATGGCGTGCCCCATGCATATAAGCATGTAC
ATACTGTGCTTGGCTTTACATGAGGATACTCATTACAAGAACTTATTTCAAGCGATAGTCTAT
GAGCATGTATTTCACTTAGTCCAAGAGCTTGATCACCAAGCCTCGAGAAACCAGCAATCCTT
GCGAGTACGTGTACCTCTTCTCGCTCCGGGCCCATAATTTGTGGGGGTTTCTATACTGAAACT
ATACCTGGCATCTGGTTCTTACCTCAGGGCCATGTTAGCGTCAACTCAATCCTACTAACCCTT
CAAATGGGACATCTCGATGGACTAATGACTAATCAGCCCATGATCACACATAACTGTGGTGT
CATGCATTTGGTATTTTTTAATTTTAGGGGGGGAACTTGCTATGACTCAGCTATGACCGTAAA
GGTCTCGTCGCAGTCAAATCAGCTGTAGCTGGGCTTATTCATCTTTCGAGGCTCCTCATGGAT
ACCCATAAGGTGCAATTCAGTCAATGGTCACAGGACATAACACTATAGATCACCCGGACTG
GCGTTACGTGTACGTACGTGTACGTACGTGTACGTACGTGTACGCACGTGTACGTACGTGTA
CGTACGTGTACGTACGTGTACGCACGTGTACGTACGTGTACGCACCCGGACTGGCGTTACGT
GTACGTACGTGTACGTACGTGTACGTACGTGTACGCACGTGTACGTACGTGTACGTACGTGT
ACGTACGTGTACGCACGTGTACGTACGTGTACGCACGTGTACGTACGTGTACGCACGTGTAC
GCACGTGTACGCACGTGTACGTACGTGTACGTACGTGTACGTACGTGTACGTACGTGTACGC
ACGTGTACGCACGTGTACGCACGTGTACGCACGTGTACGCACGTGTACGCACGTGTACGTAC
GTGTACGCGTACGTATTTTAGATACTAAGTTAGCTTAGACAAACCCCCCTTACCCCCCGTAA
CTTCAAGAAGCTTACATATACTTATGGATGTCCTGCCAAACCCCAAAAACAAGACTAAATAT
ATGCGCAAACATGAAGTCACTTACACCTAAACCCATATAATTAAGCTAACCCCCCAGCCAAT
GTTGCAACAACTACGGACATGGGACTCTAAATTTTAATTTATCTATAGATATTTTTCTTTTAC
TGTGTCTCCCCAGCATTGATTTTTTAATTATCATTATTCCACACCACCAATTTCCATTGAGCTA
TTTCACATGAGTTCCAAATCAATTATGTTCATGTAGCTTAACGAATAAAGCAAGGTACTGAA
AATGCCTAGATGGGTCACGCTACCCCATAGACATAAAGGTTTGGTCCTAGCCTTCCTATTAG
CCATTAACAAGATTACACATGTAAGTCTCCACGCTCCAGTGAAAATGCCCCTTAAGTCCTCT
TAGACGACCTAAAGGAGCGGGTATCAAGCACACCTTATGGTAGCTCACAACGCCTTGCTTAG
CCACACCCCCACGGGAAACAGCAGTGATAAAAATTAAGCTATGAACGAAAGTTCGACTAAG
CTATGTTAATACTAGGGTTGGTAAATCTCGTGCCAGCCACCGCGGTCATACGATTAACTCGA
GTTAATAGGCCTACGGCGTAAAGCGTGTAAAAGAAAAAATCTCCTCTACTAAAGTTAAAGT

ATGATTAAGCTGTAAAAAGCTACCATTAATACTAAAATAAACTACGAAAGTGACTTTAAAAT
TTCTGATTACACGATAGCTAGGGCCCAAACTGGGATTAGATACCCCACTATGCCTAGCTCTA
AACATAGATATTTTACTAAACAAAACTATTCGCCAGAGAACTACTAGCAACAGCTTAAAACT
CAAAGGACTTGGCGGTGCTTTATATCCCCCTAGAGGAGCCTGTTCTGTAATCGATAAACCCC
GATAGACCTCACCATCCCTTGCTAATTCAGTTTATATACCGCCATCTTCAGCAAACCCTTAAA
AGGAAAAAAAGTAAGCATAACTACCCTACATAAAAAAGTTAGGTCAAGGTGTAACCTATGG
GCTGGGAAGAAATGGGCTACATTTTCTATTCAAGAACAACTTCTACGAAAACTTTTATGAAA
CTAAAAGCTAAAGGCGGATTTAGTAGTAAATTAAGAATAGAGAGCTTAATTGAACAGGGCA
ATGAAGCACGCACACACCGCCCGTCACCCTCCTCGAGTGATATAATTTAATTATAACCTATT
TAAACTAAGCAAAGCATAAGAGGAGACAAGTCGTAACAAGGTAAGCATACTGGAAAGTGTG
CTTGGATGAGCCAAAGTGTAGCTTAAACAAAGCGTCTGGCTTACATCCAGAAGATTTCATTA
ATATATGACTACTTTGAACCCAAAGCTAGCCCAAGCAACAATGACTAGTAAAACCATTATGA
AACATTCAAACAAAACATTTAGTAGCATGACTAGAGTATAGGAGATAGAAATTTTTAACTGG
AGCTATAGAGAGAGTACCGCAAGGGAATGATGAAAGATTACCTAAAGTGATAAACAGCAAA
GATTGCCCCTTCTACCTTTTGTATAATGAGTTAGCTAGAAATAACTTAACAAAGAGAACTTA
AGCTAAGTCCCCCGAAACCAGACGAGCTACCTATGAACAATCCACTGGGATGAACTCATCTA
TGTTGCAAAATAGTGAGAAGATCCATAGGTAGAGGTGAAAGGCCTAACGAGCCTGGTGATA
GCTGGTTGCCCAGAATAGAATTTTAGTTCGACTTTAAACCTGCCTACAAAACTAATAATTCT
AATGCAGATTTAAAATATATTCTAAAAAGGTACAGCTTTTTAGAGTTAAGGATACAGCCTTA
CTTAGAGAGTAAATATTTATATAAGCCATAGTAGGCCTAGAGGCAGCCATCAATTAAGAAA
GCGTTAAAGCTCAACATCTCTATTAACTTAATACCAAGAATATTTAATCAACTCCTAATGTAT
TACTGGGTCAATCTATTTAAATATAGAAGTGATAATGCTAATATGAGTAACAAGAAATATTT
CTCCCATGCATAAGCTTATAACAGCAACGGATAACCACTGATAGTTAACAACAACATAGAA
ATAACCTAATGATAAAACACCTATTAAATCAATTGTTAGTCCAACACAGGCATGCAATCAGG
GAAAGATTAAAAGAAGTGAAAGGAACTCGGCAAATACAAACCCCGCCTGTTTACCAAAAAC
ATCACCTCCAGCATTTCCAGTATTGGAGGCACTGCCTGCCCGGTGACATCAGTTAAACGGCC
GCGGTATTCTGACCGTGCAAAGGTAGCATAATCATTTGTTCTCTAAATAAGGACTTGTATGA
ATGGCCACACGAGGGTTTAACTGTCTCTTACTTCCAATCAGTGAAATTGACCTCCCCGTGAA
GAGGCGGGGATAAGACAATAAGACGAGAAGACCCTATGGAGCTTTAATTAACTAATTCAAA
AAGAAACTACTAACGACCCAACAGGAATAATATATCTCTTTTATGAATTAGCAATTTAGGTT
GGGGCGACCTCGGAGGACAAAATAGCCTCCGAGTGATTATAAATCTAGACTTACCAGTCAA
AATGCTTAATCACTTATTGATCCAAAAATTCTTTTGATCAACGGAACAAGTTACCCTAGGGA
TAACAGCGCAATCCTATCCGAGAGTCCATATCGACAATAGGGTTTACGACCTCGATGTTGGA
TCAGGACATCCTAATGGTGCAGCAGCTATTAAAGGTTCGTTTGTTCAACGATTAAAGTCCTA
CGTGATCTGAGTTCAGACCGGAGCAATCCAGGTCGGTTTCTATCTATTCAAATAATTTCTCCC
AGTACGAAAGGACAAGAGAAATAAGGCCTACTTCTCTGAAGCGCCTTAAGACCAATAGATG
AATTTATCTAAATCTAGTAAATCTAACTCCAATATTGCCCAAGAGACAGGGCTTTGTTAGGG
TGGCAGAGCCCGGTAATTGTGCAAAACTTAAACTCTTGTGTCCAGAGGTTCAATTCCTCTCC
CTAGCATATGTTTATAATTAACATCTTCTCACTAATTGTACCTATTCTTCTTGCTGTAGCCTTT
CTGACTCTAGTAGAACGAAAAGTACTAGGCTACATACAGCTTCGAAAAGGCCCAAATATTGT
AGGTCCATATGGTCTCCTACAACCAATCGCAGATGCCGTAAAACTCTTTACTAAAGAACCTT
TACGACCACTTACGTCATCCATAACTATATTTATTATAGCCCCTATTCTAGCCTTAACACTAG
CTCTGACCATGTGAGTCCCCTTACCAATACCGCACCCCCTTATTAACATAAACCTAGGAGTA
CTATTCATACTAGCTATATCAAGTCTAGCTGTATACTCTATTCTCTGATCTGGATGAGCCTCA
AACTCAAAATATGCCCTAATTGGAGCCCTACGAGCCGTAGCTCAAACGATCTCATATGAAGT
CACACTAGCCATTATCCTTTTATCCGTTCTACTAATAAATGGCTCATTTACGCTATCCACGCT
AATCATTACCCAAGAACATCTTTGATTAATCTTCCCTGCATGACCACTAGCTATAATATGATT
TATTTCTACCCTAGCAGAAACAAATCGAGCCCCCTTTGACCTAACAGAAGGAGAATCAGAAC
TAGTCTCTGGGTTTAATGTCGAATATGCAGCAGGTCCATTTGCCCTGTTCTTCCTAGCAGAAT
ATGCCAACATCATCATAATAAATGCTCTTACAACTATTCTATTCTTCGGAGCATTTCACAGCC
CCTACATACCAGAATTATACATTGTTAATTTTACCGTAAAAACTTTACTCCTCACAATCTCTT

TTCTATGAATCCGAGCATCATATCCTCGATTCCGATACGACCAACTAATACACCTACTATGA
AAAAACTTTCTACCTCTCACACTAGCTCTATGTATATGACATGTAACTCTACCTATTATTACA
GCAAGCATTCCACCTCAAACGTAAGAAATATGTCTGACAAAAGAATTACTTTGATAGAGTAA
GCCATAGGGGTTTAAGTCCCCTTATTTCTAGAATCATAGGAATCGAACCTAATCTTAAGAAC
TCAAAAATCTTCGTGCTACCTAACTACACCATATCCTAAAGTAAGGTCAGCTAAATAAGCTA
TCGGGCCCATACCCCGAAAATGTTGGTTTATCCCCTTCCCATACTAATAAAACCAGCCATCCT
TATTACCATTACATCCACCGTTGTCTTAGGAACTATAATTGTTCTATTTAGCTCCCACTGATT
CATAATCTGAGTTGGCTTTGAAATAAATATACTAGCCATTATCCCAATTCTAATAAAAAAAT
ATAACCCACGAGCCATAGAAGCTTCCACAAAATATTTCTAACACAAGCTACAGCATCCATG
CTTCTAATGCTAAGCATCATCATCAATCTACTATGCTCAGGACACTGGACAGTTTCAACAATT
CCCAATCCAGTAGCATCTACCATAATTACTATTGCTCTAACAATAAAACTTGGCCTATCACC
ATTTCACTTTTGAGTCCCAGAAGTTACACAAGGCATTTCCTTATCTTCAGGAATAATCCTATT
AACATGACAAAAAATTGCACCTTTATCCATTCTATACCAAATTTCACCATCCGTTAACTCAA
ATCTCTTACTAATAATAGCTATCACATCCATACTGGTAGGAGGCTGAGGAGGATTAAATCAA
ACCCAACTCCGAAAAATCTTGGCATACTCCTCAATTACCCATATAGGCTGAATGGCTGCTAT
TATAGTATATAATCCTACCCTAGCAATTCTTAACCTAACAATTTACATCATAATAACTCTAGG
AACATTTATACTATTCATACATAGCTCATCTACAACAACACTATCACTATCTTATACATGAAA
TAAATTCCCATTAATAGCCCCACTAATCTTAATACTTATACTATCACTAGGAGGCCTCCCTCC
TCTCTCAGGCTTTATCCCCAAATGAATAATTATTCAAGAATTAACAAAAAACGACATAATTA
TTATACCTACACTTATAGCCATTACAGCCCTACTAAACCTATATTTTTACACACGCCTAACAT
ATACTACAGCACTAACTATATTTCCCGCAACAAATAATATAAAAATAAAATGACAATTCGAA
ACCACAAAAAAGACAACTCTCCTAGCCCCTCTAATCGTGACATCAACTATACTACTACCACT
AACCCCAATACTAGCAGCTCTTGATTAGAGATTTAGGCTAAACAGACCAAGGGCCTTCAAAG
CCTCAAGTAAGTGAAAATCCACTTAATCTCTGCAATATTTAAGGACTGCAAGAATCTACCTC
ACATCAACTGAACGCAAAACAATCGCTTTAATTAAGCTAAGCCCTTCTAGATCGGTGGGCTT
TTACCCCACAAAACTTTAGTTAACAGCTAAAAACCCTAGACAACTGGCTTCAATCTACTTCT
CCCGCCGCGTAGGAAAAAAAGGCGGGAGAAGCCCCGGCAGAATTGAAGCTGCTTCTTTGAA
TTTGCAATTCAATATGATTACTCACCACAGAGCCTGGTAAAAAGAGGACTTAACCCCCATTC
TTAGATTTACAGTCTAGTGCTTTTATCAGCCATTTTACCTATGTTCATTAACCGATGACTGTTT
TCCACCAACCACAAAGATATTGGCACTCTTTATCTTCTGTTTGGCGCATGAGCTGGTATAACG
GGCACTGCTCTTAGCCTTCTAATCCGTGCTGAATTAGGTCAGCCTGGAGCTCTGTTAGGAGA
TGACCAAATTTACAATGTAGTTGTAACTGCCCACGCATTCGTAATAATCTTCTTTATAGTTAT
GCCTATTATGATCGGAGGCTTTGGAAATTGACTAGTACCTTTGATAATTGGTGCGCCTGATAT
AGCATTTCCCCGAATAAACAATATGAGTTTCTGATTACTACCTCCATCCTTTCTTCTCCTTCTG
GCTTCTTCCATAGTAGAAGCAGGCGCAGGGACTGGATGAACTGTATACCCTCCTCTAGCAGG
TAACTTAGCCCATGCAGGAGCATCAGTAGACCTAACAATCTTTTCTCTACATCTGGCAGGGG
TTTCTTCTATTTTAGGGGCTATTAACTTTATTACCACTATCATCAACATGAAACCCCCTGCAA
TATCTCAATATCAAACTCCCCTATTCGTGTGATCAGTACTTATCACAGCAGTACTTCTCCTTC
TATCTTTACCAGTCTTAGCAGCTGGAATTACTATACTACTTACAGATCGAAATCTCAATACTA
CTTTCTTCGACCCGGCCGGAGGAGGGGATCCCATTCTATATCAACATCTATTTTGATTCTTTG
GCCATCCTGAAGTCTATATTCTAATCCTCCCTGGGTTTGGAATAATTTCACACATTGTCACAT
ACTATTCAGGAAAGAAAGAGCCCTTTGGTTATATGGGAATAGTGTGGGCAATAATGTCAATT
GGATTCTTAGGATTTATTGTATGGGCCCATCATATATTTACTGTAGGCATAGATGTTGATACA
CGAGCCTATTTTACCTCGGCCACTATAATTATTGCAATCCCGACAGGAGTTAAAGTATTCAG
CTGACTAGCTACCCTGCATGGAGGAAATATTAAGTGATCCCCTGCCATAATGTGAGCCCTAG
GTTTCATTTTTCTATTTACAGTAGGGGGTCTTACAGGCATTGTTCTAGCTAACTCATCTCTGG
ACATTGTCCTCCATGACACATACTACGTAGTAGCACACTTCCACTATGTACTATCAATAGGG
GCCGTGTTCGCTATTATAGGAGGCTTCGTCCATTGATTCCCATTATTTTCGGGCTATACACTT
AATAATACATGAGCAAAAATTCACTTCGTAATTATATTTGTTGGAGTCAATATAACATTCTTC
CCCCAGCACTTCCTAGGTCTATCAGGTATGCCTCGACGATATTCTGATTATCCAGACGCCTAT
ACAGCATGAAACACAGTGTCTTCAATGGGTTCATTCATTTCACTAACAGCAGTTATGTTAAT

AATTTTCATAATTTGAGAAGCCTTCGCATCTAAACGAGAAGTGGCAGTGATTGAGCTTACTA
CAACTAATATTGAATGGCTCCACGGATGCCCTCCCCCATACCATACATTCGAAGAGCCCGCC
CACGTCATACTGAAATAAGAAAGGAAGGAATCGAACCCTCTAGAATTGGTTTCAAGCCAGC
ATCATAACCACTATGTCTCTCTCAATAAAGAGGTATTAGTAAAAATTACATAACTTTGTCAA
AGTTGAATTATAGGTGAAAATCCTTTATATCTCTATGGCGCATCCCTTTCAAACAGGCCTTCA
AGATGCAACTTCCCCCATCATGGAAGAACTCCTACACTTCCATGATCATACACTAATAATTG
TATTCTTAATTAGTTCCTTAGTTCTCTATATCATTTCAATTATATTAACTACTAAATTAACACA
TACAAATACAATAGACGCACAAGAAGTGGAGACAGTATGAACTATTCTACCAGCTATCATCT
TAATTATAATTGCACTTCCATCACTACGAATTCTTTATATGATAGATGAAATTAATAACCCAT
CATTAACTGTAAAGACTATGGGGCATCAATGATACTGAAGCTATGAGTATACAGATTATGAA
GACTTGAGCTTCGATTCTTATATAACCCCAACACAAGAATTAAAGCCCGGAGAGCTGCGATT
ACTAGAGGTAGATAATCGAGTAGTACTACCCATAGAAATAACAATTCGCATATTAATTTCCT
CAGAAGATGTGCTACATTCGTGAGCTGTCCCATCTCTAGGACTAAAAACTGATGCAATCCCG
GGGCGATTAAATCAAACAACTCTTATGGCCATGCGACCAGGACTATATTATGGCCAATGCTC
AGAGATTTGCGGTTCTAATCATAGCTTTATACCCATTGTACTTGAAATAGTTCCGCTGTCTTA
CTTTGAAAAATGATCCGCTTCAATACTATAAAATCATTAAGAAGCTTTATAGCGTTAACCTTT
TAAGTTAAAGACTGAGAGTGCAAGTCTCTCCTTAATGGAAATGCCACAATTAGACACATCCA
CATGATTTATTACAATCCTATCCATGATCTTAACACTATTTATTATATTTCAGTTAAAAGTCT
CAAAACACAACTATCCTGAGTTTCCTGAATTAAAAACAACCCCACTATCAAAAAAACCTATG
CCTTGAGAAGAAAAATGAACGAAAATCTGTTCGCCTCTTTTACTACCCCAATGATAATAGGA
GTACCAATTGTGGTTCTGATTATTATTTTCCCAAGTATCTTATTTCCCTCACCTAATCGATTAA
TCAATAACCGCCTAGCTACTGTCCAACAATGATTAATTCAATTAGTATCTAAGCATATACTGT
CTATTCACAATTATAAAGGACAAACCTGAGCGCTAATACTAATGTCATTAATTTTATTTATTG
GCTCAACTAACCTTTTAGGCCTATTACCACATTCATTCACACCTACTACACAACTATCAATAA
ATTTAGGAATGGCCATTCCTCTGTGAGCAGGTACAGTAGTTATAGGATTTCGACACAAAACT
AAGGCATCTCTGGCCCACTTTCTACCCCAAGGAACACCTCTTCCTCTGATCCCAATGTTAGTA
ATTATCGAGACAATTAGTCTGTTTATTCAACCTGTGGCCCTAGCTGTACGACTGACCGCTAAT
ATTACTGCAGGCCATCTACTTATTCATTTAATCGGAGGGGCTACCCTAGTTTTAATAAACATT
AACCCTGTAACAGCCCTAATTACCTTTATTATTCTTATTCTACTTACTATTCTCGAATTAGCTG
TAGCTCTTATCCAAGCCTATGTTTTCACCCTACTAGTTAGTCTATACTTACATGATAATACCT
AATGATCCACCAAACACATGCATATCATATAGTTAATCCAAGCCCATGGCCACTTACAGGAG
CTCTCTCGGCTCTTCTTATAACATCAGGACTAATTATATGATTTCACTTTAACTCGGTATACTT
ACTAATACTAGGCCTCACCACCAACATACTTACCATGTACCAATGATGACGAGATATCGTCC
GGGAAAGTACCTTCCAAGGCCATCACACCCCAATCGTCCAAAAAGGACTACGATATGGAAT
AGTCTTATTTATTGTATCAGAAGTATTTTTCTTCGCGGGATTCTTTTGGGCCTTCTACCACTCA
AGCCTAGCACCTACTCCTGAATTAGGAGCATGTTGACCACCTACAGGTATCATCCCCTTAAA
TCCGCTAGAAGTTCCACTACTTAACACCTCTGTACTTCTAGCATCTGGGGTATCTATCACTTG
AGCTCATCATAGTCTAATAGAAGGAGACCGCAAACATATGCTCCAAGCCCTATTTATTACTA
TTTCACTAGGAGTTTATTTTACACTTCTACAAGCCTCAGAATACTATGAGACATCCTTTACTA
TTTCAGATGGAGTGTATGGTTCAACCTTCTTTATAGCAACTGGATTTCATGGGCTTCATGTAA
TTATTGGTTCAACTTTTCTTACTGTGTGTTTTCTACGACAACTGCACTTTCACTTTACATCCAA
CCATCACTTTGGCTTTGAAGCGGCAGCATGATACTGACACTTCGTAGACGTTGTATGGCTTTT
CCTTTATGTTTCTATTTACTGATGAGGATCCTACTTCTTTAGTATTAACTAGTACGATTGACCT
CCAATCAATAGGCTCTGGTACAAGCCAGAAAGAAGTAATAAACCTAATTCTAGCACTTCTAA
CCAACGTATTATTAGCCTCTCTGCTCGTTCTTATTGCATTCTGACTGCCACAACTAAATATCT
ATGCAGAAAAAGTAAGTCCTTATGAATGTGGATTTGATCCTATAGGATCAGCACGCCTTCCT
TTCTCTATAAAATTTTTTCTAGTAGCTATTACATTCCTTTTATTCGACTTAGAAATTGCTCTAC
TCCTCCCGCTTCCTTGAGCATTACAGACTAACAACTTAATAACTATACTTATTATAGCTCTAT
TACTCATTTCCCTATTAGCCGCAAGCCTAGCTTATGAGTGAACCGAAAAGGGGCTAGAATGG
GCCGAATATGATAATTAGTTTAAACTAAAATAAGTGATTTCGACTCACTAGATTATGACTAA
CATCATAATTATCAAATGTCTGTGGTCTATATTAATCTCTTTCTAGCCTTTACTATATCCTTAA

TAGGGCTACTCATCTATCGATCCCATTTAATATCCTCCCTATTATGCTTAGAAGGCATGATGC
TGTCACTATTTGTTATACTAGCAGTAACAATCCTAAGTAACCACTTCACACTAGCCAGTATG
ACTCCAATCATTCTATTAGTATTCGCTGCCTGTGAAGCAGCGTTGGGGCTTTCTCTGCTAGTA
ATAGTCTCTAATACCTATGGAACTGACTACGTACAAAATCTTAACCTCCTACAATGCTAAAA
ATTATCCTCCCTACCATAATACTAGTACCCCTTACATGAATATCAAAGCCTAACATAATCTGA
ATTAATACAACAACCTATAGTCTACTCATCAGCCTTATTAGTCTGACATATCTCAATCAACTC
AGTGATAATAGTCTAAACTTCTCATTAACATTCTTTACCGACTCCCTATCAGCCCCCCTGTTA
GTTCTCACAACATGACTACTGCCCTTAATACTTATAGCTAGTCAATTTCACCTGTCAAAAGAG
ACCCTGACCCGGAAAAAATTATATATCACAATACTAATTCTCCTGCAACTATTCTTAATTATA
ACATTTTCTGCTACTGAGTTAATCATATTCTATATTTTATTTGAAGCTACCCTGGTACCGACTT
TAGTTATCATTACTCGATGAGGAAACCAAACAGAACGACTAAACGCGGGCCTCTATTTTTTA
TTCTACACCTTAGTAGGATCCTTGCCCTTACTAGTTGCACTGTTATATGCCCAAAACAGCTTA
GGATCACTAAATTTTCTCATAGTACAGTACTGAACTCAGCCCTTGCCAAATTCCTGATCTAAC
ATTTTCATGTGGCTGGCATGTATAATAGCATTCATAGTAAAAATACCGCTATATGGCTTACA
CCTATGACTACCTAAAGCACATGTAGAAGCTCCTATTGCAGGATCCATAGTACTTGCTGCAG
TACTTCTAAAACTAGGGGGATACGGTATAATGCGGATTACAACAATACTCAACCCTCTAACA
AATTTTATAGCCTACCCCTTCATAATGCTCTCACTATGAGGAATAATCATAACAAGTTCTATT
TGTTTACGCCAGACAGACCTAAAGTCCCTAATTGCATACTCCTCTGTTAGTCACATAGCCCTA
GTCATTGTAGCAATTCTAATCCAATCCCCATGAAGCTACATAGGAGCTACTGCTCTAATAAT
CGCCCATGGGTTAACATCTTCTATGCTATTCTGTCTTGCAAACTCTAACTACGAGCGAACCCA
TAGCCGAACTATAATTCTCGCACGGGGCTTGCAAACGCTTCTTCCACTTATAGCAGCTTGAT
GACTATTAGCAGGCCTAACTAACTTAGCTCTTCCTCCTACAATTAATCTGATTGGGGAACTGT
TTGTAGTGATAGCCACATTTTCATGATCCAATATTACCATTCTCTTAATAGGAGTTAACATTA
CCATTACTGCTCTCTACTCTCTATATATACTAATTTCTACACAGCGAGGCAAGTATACAGACC
ACATCAAAAATATTAAGCCATCTTATACACGAGAAAATGCCTTAATAGCCTTACATTTACTA
CCCCTTTTACTTCTATCTCTTAACCCTAAAGTAGTTCTAGGCTTTATTTACTGTAAATATAGTT
TAACAAAAACATTAGATTGTGAATCTAGTAATAAAAGCTTAAATCTTTTTATTTACCGAAAA
AGCATGCAAGAACTGCTAATTCATGCACCCATGCATAAAATCATGGCTTTTTCAACTTTTAG
GGGATAGAAGTAATCCATTGGTCTTAGGAGCCAAAAAATTGGTGCAACTCCAAATAAAAGT
AATTAACCTACTTACCTCCTTCACCATTACAACCCTATTTATATTAATCATGCCCATTATCCT
AACCTGTACCCCAGCATATGAAAATAAACTTTACCCGCAATATGTAAAAACCACAGTCTCTT
ATGCCTTCACAATTAGCATAATTCCCATAGTAATATTCATCCACTCCGGACAAGAAGCAATC
ATCTCAAACTGACACTGAATAACCATCCAAACCCTAAAACTCAGTCTAAGCTTCAAATTAGA
TTACTTTTCAATAATCTTCGTACCAGTGGCCCTATTCGTCACATGGTCAATTATAGAATTCTC
TATATGATATATACACGCAGATCCTAACATTAACCGATTCTTTAAATATCTACTTATATTCCT
CGTCACGATAATAATTCTAGTAACCGCAAATAATCTATTTCAGCTATTTATCGGCTGAGAGG
GAGTAGGAATCATATCATTTCTCCTCATTGGATGATGATATGGACGAACAGATGCTAATACG
GCTGCCCTACAAGCAGTACTATACAACCGCATTGGAGATGTTGGTTTTATCATGGCCATAGC
TTGATTCTTACTCAATACAAATGCATGGGACTTCCAACAAATCTTTATAATCGAGCATAAAA
ACCTCAATATTCCACTGGCAGGACTACTTCTAGCAGCTACCGGAAAGTCTGCCCAATTTGGA
CTTCACCCATGACTCCCATCAGCCATAGAAGGCCCCACTCCTGTCTCAGCACTACTTCACTCT
AGCACAATAGTAGTAGCAGGCGTATTTCTTCTAGTCCGATTTCACCCCTTAATAGAACATAA
CAAGACAATTCAAACGGTTACCCTATGCTTAGGGGCAATAACAACCTTGTTTACAGCAATCT
GCGCCCTTACCCAAAATGATATCAAAAAAATTATTGCCTTCTCCACCTCGAGCCAACTAGGA
CTAATAATTGTAACAATTGGTATTAATCAGCCTCATCTGGCCTTTCTCCATATCTGCACGCAC
GCATTCTTCAAGGCCGTGCTATTTCTATGCTCCGGATCTATTATTCATAGTCTAAACAACGAA
CAAGATATTCGAAAGATAGGAGGGCTATTTAAAAGTCTACCATTTACCACTACTGCTCTAAT
CATCGGAACTCTAGCACTTACAGGAATGCCCTTCTTAACAGGATTTTACTCCAAAGACTTAA
TTATTGAAACCGCCAATACGTCATACACCAACGCCTGAGCCTTACTAACAACCCTTATCGCC
ACATCCATAACTGCCGCCTACAGTACTCGAATTGTATTTTTTACACTTCTAGGACAACCTCGC
TATAGTCCCATTATCACCATTAACGAGAATAACCAGTTCCTGACTAACTCTGTCAAACGATT

GCTACTAGGAAGTATCTTTGCGGGATTCCTAATCTCTCATAACATTACACCCACCACTGTCCC
ACAAATAACCATGCCCTGTCACCTAAAAATGATAGCCCTTACAGTAACAATCCTAGGCTTTA
TTCTAGCACTAGAACTTAACCTAGCCATACAAAATCTCAAATTTGACTACCCTTTAAGCACG
TTCAAATTTTCAAATCAACTAGGATATTTCCCTACTACTCTGCATCGTCTCGCACCAATAATA
GGTCTATCAATAAGCCAAAAAACAGCATCCATACTGCTAGATGCAATCTGACTAGAGAATGT
TCTACCAAAGTCCATCTCCTACTTTCAAATAAAATCCTCAATCACAGTCTCCAACCAAAAGG
GACTAATTAAACTATACTTTCTCTCCTTTATAATTACACTAGTCTTAAGTCTCTTTATACTTAA
TTTCCACGAGTGACTTCCATAATTACCAATACACCAATAAGGAGAGATCATCCAGTAACAAT
AACTAATCAAGTACCATAACTATATAAAGCTGCAATTCCCATAGCCTCTTCACTAAAAAATC
CTGAATCCCCTGTGTCATAAATCACCCAATCTCCTATTCCATTAAACTTAAATACTACCTCGG
CTTCATCATCCTTTAAAATATAACAAACAGCCAGTAACTCAGACAACAGACCTACAATAAAT
GCACCTAGTACAGCCTTATTAGAAAGTCAAACCTCAGGATATTGCTCAGTGGCTATGGCCGT
AGTGTACCCGAACACTACAAGTATACCCCCTAAATAAATTAAAAAGACCATTAAACCCAAA
AAAGATCCTCCAAAGCTTAACACAATACCACACCCTACACCCCCACTAACAATCAAGACTAA
ACCTCCGTAGATAGGAGAGGGCTTAGAAGAAAACCCCGCAAAACTAACTACAAAAACGATA
CTTAAAATAGATACAATGTATGTTATCATCATTCTTACATGGAATCTAACCATGACCAATGA
CATGAAAAATCATCGTTGTATTTCAACTATAAGAACCCCAATGATCAACATCCGAAAAACTC
ATCCATTAGTTAAAATTATCAACAACTCATTCATTGACCTTCCAACACCATCAAACATTTCAA
CATGATGAAACTTTGGGTCCCTGTTAGGAGTGTGTCTGATCTTGCAAATCTTAACAGGCTTAT
TTCTAGCCATACACTATACATCAGATACAGCTACAGCCTTTTCATCAGTCGCACACATTTGTC
GAGACGTCAACTATGGTTGATTTATCCGATATATACATGCCAATGGGGCCTCTATATTTTTA
TCTGCCTATTTATACACGTAGGGCGAGGCTTATACTATGGATCATACCTATTTCCAGAGACAT
GGAATATCGGAATTATTCTCCTACTTACAGTTATAGCCACAGCATTCATAGGGTATGTACTA
CCTTGAGGACAAATATCCTTCTGAGGAGCAACCGTCATTACTAACCTACTATCAGCAATTCC
TTACATTGGCACTAATCTAGTGGAGTGAGTCTGAGGGGGTTTCTCCGTAGATAAAGCAACAC
TAACCCGATTTTTTGCTTTTCACTTTATCCTTCCATTTATCATCTCAGCACTAGCAATAGTCCA
TCTATTATTCCTTCACGAAACAGGATCTAATAACCCCTCCGGAATTCCATCTGACCCAGACA
AAATCCCATTTTACCCCTATCATACAATTAAAGACATCCTAGGCGTCCTATTTCTTGTCCTCG
CCTTAATAACCCTGGCTTTATTCTCACCAGACCTGTTAGGAGACCCTGATAACTATACCCCTG
CAAATCCACTAAGTACCCCGCCACATATTAAGCCTGAATGGTACTTTCTATTTGCCTACGCTA
TCCTGCGATCTATTCCTAATAAACTAGGAGGGGTGCTAGCTCTAATCTTCTCTATTCTAATTC
TAACTATTATTCCACTATTACATACATCCAAACAACGAAGCATGATATTCCGACCTCTAAGT
CAATGCTTATTCTGACTCCTAGTAGCAGACCTACTCACACTAACATGAATTGGAGGACAGCC
AGTAGAACACCCCTTCATTATTATTGGGCAATTGGCCTCTATTCTCTACTTTACAATTCTTCT
AGTACTTATACCTATCACTAGCATTATTGAGAATAGCCTCTCAAAATGAAGAGTCTTTGTAG
TATATTAATTACTTTGGTCTTGTAAACCAAAAACGGAGATTCCTCTACTTCTCCCTAAGACTC
AAGGAAGGAGCAACAACCCCACTACCAGCACCCAAAGCTGGTGTTCTACTTAAACTATCCCC
TG
>SRR6109211 (circular) ReOrganized
ATACTATAAATCCACCTCTCATTTATTCACTTCATACATGCTATTACACACTCTGTGCCATC
ATAGTATGTTTTCATACATCCTCCCTTCTTTCACACCCTATGTATATCGTACATTAATGGTGTA
CCCCCCCCTCCCCCCTATGTATATCGTGCATTAATGGCGTGCCCCATGCATATAAGCATGTAC
ATACTGTGCTTGGCTTTACATGAGGATACTCATTACAAGAACTTATTTCAAGCGATAGTCTAT
GAGCATGTATTTCACTTAGTCCAAGAGCTTGATCACCAAGCCTCGAGAAACCAGCAATCCTT
GCGAGTACGTGTACCTCTTCTCGCTCCGGGCCCATAATTTGTGGGGGTTTCTATACTGAAACT
ATACCTGGCATCTGGTTCTTACCTCAGGGCCATGTTAGCGTCAACTCAATCCTACTAACCCTT
CAAATGGGACATCTCGATGGACTAATGACTAATCAGCCCATGATCACACATAACTGTGGTGT
CATGCATTTGGTATTTTTTAATTTTAGGGGGGGAACTTGCTATGACTCAGCTATGACCGTAAA
GGTCTCGTCGCAGTCAAATCAGCTGTAGCTGGGCTTATTCATCTTTCGAGGCTCCTCATGGAT
ACCCATAAGGTGCAATTCAGTCAATGGTCACAGGACATAACACTATAGATCACCCGGACTG
GCGTTACGTGTACGTACGTGTACGTACGTGTACGTACGTGTACGCACGTGTACGTACGTGTA

CGCACGTGTACGTACGTGTACGCACGTGTACGCACGTGTACGCACGTGTACGCACGTGTACG
CACGTGTACGTACGTGTACGCGTACGTATTTTAGATACTAAGTTAGCTTAGACAAACCCCCC
TTACCCCCCGTAACTTCAAGAAGCTTACATATACTTATGGATGTCCTGCCAAACCCCAAAAA
CAAGACTAAATATATGCGCAAACATGAAGTCACTTACACCTAAACCCATATAATTAAGCTAA
CCCCCCAGCCAATGTTGCAACAACTACGGACATGGGACTCTAAATTTTAATTTATCTATAGA
TATTTTTCTTTTACTGTGTCTCCCCAGCATTGATTTTTTAATTATCATTATTCCACACCACCAA
TTTCCATTGAGCTATTTCACATGAGTTCCAAATCAATTATGTTCATGTAGCTTAACGAATAAA
GCAAGGTACTGAAAATGCCTAGATGGGTCACGCTACCCCATAGACATAAAGGTTTGGTCCTA
GCCTTCCTATTAGCCATTAACAAGATTACACATGTAAGTCTCCACGCTCCAGTGAAAATGCC
CCTTAAGTCCTCTTAGACGACCTAAAGGAGCGGGTATCAAGCACACCTTATGGTAGCTCACA
ACGCCTTGCTTAGCCACACCCCCACGGGAAACAGCAGTGATAAAAATTAAGCTATGAACGA
AAGTTCGACTAAGCTATGTTAATACTAGGGTTGGTAAATCTCGTGCCAGCCACCGCGGTCAT
ACGATTAACTCGAGTTAATAGGCCTACGGCGTAAAGCGTGTAAAAGAAAAAATCTCCTCTAC
TAAAGTTAAAGTATGATTAAGCTGTAAAAAGCTACCATTAATACTAAAATAAACTACGAAA
GTGACTTTAAAATTTCTGATTACACGATAGCTAGGGCCCAAACTGGGATTAGATACCCCACT
ATGCCTAGCTCTAAACATAGATATTTTACTAAACAAAACTATTCGCCAGAGAACTACTAGCA
ACAGCTTAAAACTCAAAGGACTTGGCGGTGCTTTATATCCCCCTAGAGGAGCCTGTTCTGTA
ATCGATAAACCCCGATAGACCTCACCATCCCTTGCTAATTCAGTTTATATACCGCCATCTTCA
GCAAACCCTTAAAAGGAAAAAAAGTAAGCATAACTACCCTACATAAAAAAGTTAGGTCAAG
GTGTAACCTATGGGCTGGGAAGAAATGGGCTACATTTTCTATTCAAGAACAACTTCTACGAA
AACTTTTATGAAACTAAAAGCTAAAGGCGGATTTAGTAGTAAATTAAGAATAGAGAGCTTA
ATTGAACAGGGCAATGAAGCACGCACACACCGCCCGTCACCCTCCTCGAGTGATATAATTTA
ATTATAACCTATTTAAACTAAGCAAAGCATAAGAGGAGACAAGTCGTAACAAGGTAAGCAT
ACTGGAAAGTGTGCTTGGATGAGCCAAAGTGTAGCTTAAACAAAGCGTCTGGCTTACATCCA
GAAGATTTCATTAATATATGACTACTTTGAACCCAAAGCTAGCCCAAGCAACAATGACTAGT
AAAACCATTATGAAACATTCAAACAAAACATTTAGTAGCATGACTAGAGTATAGGAGATAG
AAATTTTTAACTGGAGCTATAGAGAGAGTACCGCAAGGGAATGATGAAAGATTACCTAAAG
TGATAAACAGCAAAGATTGCCCCTTCTACCTTTTGTATAATGAGTTAGCTAGAAATAACTTA
ACAAAGAGAACTTAAGCTAAGTCCCCCGAAACCAGACGAGCTACCTATGAACAATCCACTG
GGATGAACTCATCTATGTTGCAAAATAGTGAGAAGATCCATAGGTAGAGGTGAAAGGCCTA
ACGAGCCTGGTGATAGCTGGTTGCCCAGAATAGAATTTTAGTTCGACTTTAAACCTGCCTAC
AAAACTAATAATTCTAATGCAGATTTAAAATATATTCTAAAAAGGTACAGCTTTTTAGAGTT
AAGGATACAGCCTTACTTAGAGAGTAAATATTTATATAAGCCATAGTAGGCCTAGAGGCAG
CCATCAATTAAGAAAGCGTTAAAGCTCAACATCTCTATTAACTTAATACCAAGAATATTTAA
TCAACTCCTAATGTATTACTGGGTCAATCTATTTAAATATAGAAGTGATAATGCTAATATGA
GTAACAAGAAATATTTCTCCCATGCATAAGCTTATAACAGCAACGGATAACCACTGATAGTT
AACAACAACATAGAAATAACCTAATGATAAAACACCTATTAAATCAATTGTTAGTCCAACAC
AGGCATGCAATCAGGGAAAGATTAAAAGAAGTGAAAGGAACTCGGCAAATACAAACCCCG
CCTGTTTACCAAAAACATCACCTCCAGCATTTCCAGTATTGGAGGCACTGCCTGCCCGGTGA
CATCAGTTAAACGGCCGCGGTATTCTGACCGTGCAAAGGTAGCATAATCATTTGTTCTCTAA
ATAAGGACTTGTATGAATGGCCACACGAGGGTTTAACTGTCTCTTACTTCCAATCAGTGAAA
TTGACCTCCCCGTGAAGAGGCGGGGATAAGACAATAAGACGAGAAGACCCTATGGAGCTTT
AATTAACTAATTCAAAAAGAAACTACTAACGACCCAACAGGAATAATATATCTCTTTTATGA
ATTAGCAATTTAGGTTGGGGCGACCTCGGAGGACAAAATAGCCTCCGAGTGATTATAAATCT
AGACTTACCAGTCAAAATGCTTAATCACTTATTGATCCAAAAATTCTTTTGATCAACGGAAC
AAGTTACCCTAGGGATAACAGCGCAATCCTATCCGAGAGTCCATATCGACAATAGGGTTTAC
GACCTCGATGTTGGATCAGGACATCCTAATGGTGCAGCAGCTATTAAAGGTTCGTTTGTTCA
ACGATTAAAGTCCTACGTGATCTGAGTTCAGACCGGAGCAATCCAGGTCGGTTTCTATCTAT
TCAAATAATTTCTCCCAGTACGAAAGGACAAGAGAAATAAGGCCTACTTCTCTGAAGCGCCT
TAAGACCAATAGATGAATTTATCTAAATCTAGTAAATCTAACTCCAATATTGCCCAAGAGAC
AGGGCTTTGTTAGGGTGGCAGAGCCCGGTAATTGTGCAAAACTTAAACTCTTGTGTCCAGAG

GTTCAATTCCTCTCCCTAGCATATGTTTATAATTAACATCTTCTCACTAATTGTACCTATTCTT
CTTGCTGTAGCCTTTCTGACTCTAGTAGAACGAAAAGTACTAGGCTACATACAGCTTCGAAA
AGGCCCAAATATTGTAGGTCCATATGGTCTCCTACAACCAATCGCAGATGCCGTAAAACTCT
TTACTAAAGAACCTTTACGACCACTTACGTCATCCATAACTATATTTATTATAGCCCCTATTC
TAGCCTTAACACTAGCTCTGACCATGTGAGTCCCCTTACCAATACCGCACCCCCTTATTAACA
TAAACCTAGGAGTACTATTCATACTAGCTATATCAAGTCTAGCTGTATACTCTATTCTCTGAT
CTGGATGAGCCTCAAACTCAAAATATGCCCTAATTGGAGCCCTACGAGCCGTAGCTCAAACG
ATCTCATATGAAGTCACACTAGCCATTATCCTTTTATCCGTTCTACTAATAAATGGCTCATTT
ACGCTATCCACGCTAATCATTACCCAAGAACATCTTTGATTAATCTTCCCTGCATGACCACTA
GCTATAATATGATTTATTTCTACCCTAGCAGAAACAAATCGAGCCCCCTTTGACCTAACAGA
AGGAGAATCAGAACTAGTCTCTGGGTTTAATGTCGAATATGCAGCAGGTCCATTTGCCCTGT
TCTTCCTAGCAGAATATGCCAACATCATCATAATAAATGCTCTTACAACTATTCTATTCTTCG
GAGCATTTCACAGCCCCTACATACCAGAATTATACATTGTTAATTTTACCGTAAAAACTTTAC
TCCTCACAATCTCTTTTCTATGAATCCGAGCATCATATCCTCGATTCCGATACGACCAACTAA
TACACCTACTATGAAAAAACTTTCTACCTCTCACACTAGCTCTATGTATATGACATGTAACTC
TACCTATTATTACAGCAAGCATTCCACCTCAAACGTAAGAAATATGTCTGACAAAAGAATTA
CTTTGATAGAGTAAGCCATAGGGGTTTAAGTCCCCTTATTTCTAGAATCATAGGAATCGAAC
CTAATCTTAAGAACTCAAAAATCTTCGTGCTACCTAACTACACCATATCCTAAAGTAAGGTC
AGCTAAATAAGCTATCGGGCCCATACCCCGAAAATGTTGGTTTATCCCCTTCCCATACTAAT
AAAACCAGCCATCCTTATTACCATTACATCCACCGTTGTCTTAGGAACTATAATTGTTCTATT
TAGCTCCCACTGATTCATAATCTGAGTTGGCTTTGAAATAAATATACTAGCCATTATCCCAAT
TCTAATAAAAAAATATAACCCACGAGCCATAGAAGCTTCCACAAAATATTTTCTAACACAAG
CTACAGCATCCATGCTTCTAATGCTAAGCATCATCATCAATCTACTATGCTCAGGACACTGG
ACAGTTTCAACAATTCCCAATCCAGTAGCATCTACCATAATTACTATTGCTCTAACAATAAA
ACTTGGCCTATCACCATTTCACTTTTGAGTCCCAGAAGTTACACAAGGCATTTCCTTATCTTC
AGGAATAATCCTATTAACATGACAAAAAATTGCACCTTTATCCATTCTATACCAAATTTCAC
CATCCGTTAACTCAAATCTCTTACTAATAATAGCTATCACATCCATACTGGTAGGAGGCTGA
GGAGGATTAAATCAAACCCAACTCCGAAAAATCTTGGCATACTCCTCAATTACCCATATAGG
CTGAATGGCTGCTATTATAGTATATAATCCTACCCTAGCAATTCTTAACCTAACAATTTACAT
CATAATAACTCTAGGAACATTTATACTATTCATACATAGCTCATCTACAACAACACTATCACT
ATCTTATACATGAAATAAATTCCCATTAATAGCCCCACTAATCTTAATACTTATACTATCACT
AGGAGGCCTCCCTCCTCTCTCAGGCTTTATCCCCAAATGAATAATTATTCAAGAATTAACAA
AAAACGACATAATTATTATACCTACACTTATAGCCATTACAGCCCTACTAAACCTATATTTTT
ACACACGCCTAACATATACTACAGCACTAACTATATTTCCCGCAACAAATAATATAAAAATA
AAATGACAATTCGAAACCACAAAAAAGACAACTCTCCTAGCCCCTCTAATCGTGACATCAAC
TATACTACTACCACTAACCCCAATACTAGCAGCTCTTGATTAGAGATTTAGGCTAAACAGAC
CAAGGGCCTTCAAAGCCTCAAGTAAGTGAAAATCCACTTAATCTCTGCAATATTTAAGGACT
GCAAGAATCTACCTCACATCAACTGAACGCAAAACAATCGCTTTAATTAAGCTAAGCCCTTC
TAGATCGGTGGGCTTTTACCCCACAAAACTTTAGTTAACAGCTAAAAACCCTAGACAACTGG
CTTCAATCTACTTCTCCCGCCGCGTAGGAAAAAAAGGCGGGAGAAGCCCCGGCAGAATTGA
AGCTGCTTCTTTGAATTTGCAATTCAATATGATTACTCACCACAGAGCCTGGTAAAAAGAGG
ACTTAACCCCCATTCTTAGATTTACAGTCTAGTGCTTTTATCAGCCATTTTACCTATGTTCATT
AACCGATGACTGTTTTCCACCAACCACAAAGATATTGGCACTCTTTATCTTCTGTTTGGCGCA
TGAGCTGGTATAACGGGCACTGCTCTTAGCCTTCTAATCCGTGCTGAATTAGGTCAGCCTGG
AGCTCTGTTAGGAGATGACCAAATTTACAATGTAGTTGTAACTGCCCACGCATTCGTAATAA
TCTTCTTTATAGTTATGCCTATTATGATCGGAGGCTTTGGAAATTGACTAGTACCTTTGATAA
TTGGTGCGCCTGATATAGCATTTCCCCGAATAAACAATATGAGTTTCTGATTACTACCTCCAT
CCTTTCTTCTCCTTCTGGCTTCTTCCATAGTAGAAGCAGGCGCAGGGACTGGATGAACTGTAT
ACCCTCCTCTAGCAGGTAACTTAGCCCATGCAGGAGCATCAGTAGACCTAACAATCTTTTCT
CTACATCTGGCAGGGGTTTCTTCTATTTTAGGGGCTATTAACTTTATTACCACTATCATCAAC
ATGAAACCCCCTGCAATATCTCAATATCAAACTCCCCTATTCGTGTGATCAGTACTTATCACA

GCAGTACTTCTCCTTCTATCTTTACCAGTCTTAGCAGCTGGAATTACTATACTACTTACAGAT
CGAAATCTCAATACTACTTTCTTCGACCCGGCCGGAGGAGGGGATCCCATTCTATATCAACA
TCTATTTTGATTCTTTGGCCATCCTGAAGTCTATATTCTAATCCTCCCTGGGTTTGGAATAATT
TCACACATTGTCACATACTATTCAGGAAAGAAAGAGCCCTTTGGTTATATGGGAATAGTGTG
GGCAATAATGTCAATTGGATTCTTAGGATTTATTGTATGGGCCCATCATATATTTACTGTAGG
CATAGATGTTGATACACGAGCCTATTTTACCTCGGCCACTATAATTATTGCAATCCCGACAG
GAGTTAAAGTATTCAGCTGACTAGCTACCCTGCATGGAGGAAATATTAAGTGATCCCCTGCC
ATAATGTGAGCCCTAGGTTTCATTTTTCTATTTACAGTAGGGGGTCTTACAGGCATTGTTCTA
GCTAACTCATCTCTGGACATTGTCCTCCATGACACATACTACGTAGTAGCACACTTCCACTAT
GTACTATCAATAGGGGCCGTGTTCGCTATTATAGGAGGCTTCGTCCATTGATTCCCATTATTT
TCGGGCTATACACTTAATAATACATGAGCAAAAATTCACTTCGTAATTATATTTGTTGGAGTC
AATATAACATTCTTCCCCCAGCACTTCCTAGGTCTATCAGGTATGCCTCGACGATATTCTGAT
TATCCAGACGCCTATACAGCATGAAACACAGTGTCTTCAATGGGTTCATTCATTTCACTAAC
AGCAGTTATGTTAATAATTTTCATAATTTGAGAAGCCTTCGCATCTAAACGAGAAGTGGCAG
TGATTGAGCTTACTACAACTAATATTGAATGGCTCCACGGATGCCCTCCCCCATACCATACA
TTCGAAGAGCCCGCCCACGTCATACTGAAATAAGAAAGGAAGGAATCGAACCCTCTAGAAT
TGGTTTCAAGCCAGCATCATAACCACTATGTCTCTCTCAATAAAGAGGTATTAGTAAAAATT
ACATAACTTTGTCAAAGTTGAATTATAGGTGAAAATCCTTTATATCTCTATGGCGCATCCCTT
TCAAACAGGCCTTCAAGATGCAACTTCCCCCATCATGGAAGAACTCCTACACTTCCATGATC
ATACACTAATAATTGTATTCTTAATTAGTTCCTTAGTTCTCTATATCATTTCAATTATATTAAC
TACTAAATTAACACATACAAATACAATAGACGCACAAGAAGTGGAGACAGTATGAACTATT
CTACCAGCTATCATCTTAATTATAATTGCACTTCCATCACTACGAATTCTTTATATGATAGAT
GAAATTAATAACCCATCATTAACTGTAAAGACTATGGGGCATCAATGATACTGAAGCTATGA
GTATACAGATTATGAAGACTTGAGCTTCGATTCTTATATAACCCCAACACAAGAATTAAAGC
CCGGAGAGCTGCGATTACTAGAGGTAGATAATCGAGTAGTACTACCCATAGAAATAACAAT
TCGCATATTAATTTCCTCAGAAGATGTGCTACATTCGTGAGCTGTCCCATCTCTAGGACTAAA
AACTGATGCAATCCCGGGGCGATTAAATCAAACAACTCTTATGGCCATGCGACCAGGACTAT
ATTATGGCCAATGCTCAGAGATTTGCGGTTCTAATCATAGCTTTATACCCATTGTACTTGAAA
TAGTTCCGCTGTCTTACTTTGAAAAATGATCCGCTTCAATACTATAAAATCATTAAGAAGCTT
TATAGCGTTAACCTTTTAAGTTAAAGACTGAGAGTGCAAGTCTCTCCTTAATGGAAATGCCA
CAATTAGACACATCCACATGATTTATTACAATCCTATCCATGATCTTAACACTATTTATTATA
TTTCAGTTAAAAGTCTCAAAACACAACTATCCTGAGTTTCCTGAATTAAAAACAACCCCACT
ATCAAAAAAACCTATGCCTTGAGAAGAAAAATGAACGAAAATCTGTTCGCCTCTTTTACTAC
CCCAATGATAATAGGAGTACCAATTGTGGTTCTGATTATTATTTTCCCAAGTATCTTATTTCC
CTCACCTAATCGATTAATCAATAACCGCCTAGCTACTGTCCAACAATGATTAATTCAATTAGT
ATCTAAGCATATACTGTCTATTCACAATTATAAAGGACAAACCTGAGCGCTAATACTAATGT
CATTAATTTTATTTATTGGCTCAACTAACCTTTTAGGCCTATTACCACATTCATTCACACCTAC
TACACAACTATCAATAAATTTAGGAATGGCCATTCCTCTGTGAGCAGGTACAGTAGTTATAG
GATTTCGACACAAAACTAAGGCATCTCTGGCCCACTTTCTACCCCAAGGAACACCTCTTCCT
CTGATCCCAATGTTAGTAATTATCGAGACAATTAGTCTGTTTATTCAACCTGTGGCCCTAGCT
GTACGACTGACCGCTAATATTACTGCAGGCCATCTACTTATTCATTTAATCGGAGGGGCTAC
CCTAGTTTTAATAAACATTAACCCTGTAACAGCCCTAATTACCTTTATTATTCTTATTCTACTT
ACTATTCTCGAATTAGCTGTAGCTCTTATCCAAGCCTATGTTTTCACCCTACTAGTTAGTCTA
TACTTACATGATAATACCTAATGATCCACCAAACACATGCATATCATATAGTTAATCCAAGC
CCATGGCCACTTACAGGAGCTCTCTCGGCTCTTCTTATAACATCAGGACTAATTATATGATTT
CACTTTAACTCGGTATACTTACTAATACTAGGCCTCACCACCAACATACTTACCATGTACCAA
TGATGACGAGATATCGTCCGGGAAAGTACCTTCCAAGGCCATCACACCCCAATCGTCCAAAA
AGGACTACGATATGGAATAGTCTTATTTATTGTATCAGAAGTATTTTTCTTCGCGGGATTCTT
TTGGGCCTTCTACCACTCAAGCCTAGCACCTACTCCTGAATTAGGAGCATGTTGACCACCTA
CAGGTATCATCCCCTTAAATCCGCTAGAAGTTCCACTACTTAACACCTCTGTACTTCTAGCAT
CTGGGGTATCTATCACTTGAGCTCATCATAGTCTAATAGAAGGAGACCGCAAACATATGCTC

CAAGCCCTATTTATTACTATTTCACTAGGAGTTTATTTACACTTCTACAAGCCTCAGAATAC
TATGAGACATCCTTTACTATTTCAGATGGAGTGTATGGTTCAACCTTCTTTATAGCAACTGGA
TTTCATGGGCTTCATGTAATTATTGGTTCAACTTTTCTTACTGTGTGTTTTCTACGACAACTGC
ACTTTCACTTTACATCCAACCATCACTTTGGCTTTGAAGCGGCAGCATGATACTGACACTTCG
TAGACGTTGTATGGCTTTTCCTTTATGTTTCTATTTACTGATGAGGATCCTACTTCTTTAGTAT
TAACTAGTACGATTGACCTCCAATCAATAGGCTCTGGTACAAGCCAGAAAGAAGTAATAAA
CCTAATTCTAGCACTTCTAACCAACGTATTATTAGCCTCTCTGCTCGTTCTTATTGCATTCTGA
CTGCCACAACTAAATATCTATGCAGAAAAAGTAAGTCCTTATGAATGTGGATTTGATCCTAT
AGGATCAGCACGCCTTCCTTTCTCTATAAAATTTTTTCTAGTAGCTATTACATTCCTTTTATTC
GACTTAGAAATTGCTCTACTCCTCCCGCTTCCTTGAGCATTACAGACTAACAACTTAATAACT
ATACTTATTATAGCTCTATTACTCATTTCCCTATTAGCCGCAAGCCTAGCTTATGAGTGAACC
GAAAAGGGGCTAGAATGGGCCGAATATGATAATTAGTTTAAACTAAAATAAGTGATTTCGA
CTCACTAGATTATGACTAACATCATAATTATCAAATGTCTGTGGTCTATATTAATCTCTTTCT
AGCCTTTACTATATCCTTAATAGGGCTACTCATCTATCGATCCCATTTAATATCCTCCCTATT
ATGCTTAGAAGGCATGATGCTGTCACTATTTGTTATACTAGCAGTAACAATCCTAAGTAACC
ACTTCACACTAGCCAGTATGACTCCAATCATTCTATTAGTATTCGCTGCCTGTGAAGCAGCGT
TGGGGCTTTCTCTGCTAGTAATAGTCTCTAATACCTATGGAACTGACTACGTACAAAATCTTA
ACCTCCTACAATGCTAAAAATTATCCTCCCTACCATAATACTAGTACCCCTTACATGAATATC
AAAGCCTAACATAATCTGAATTAATACAACAACCTATAGTCTACTCATCAGCCTTATTAGTC
TGACATATCTCAATCAACTCAGTGATAATAGTCTAAACTTCTCATTAACATTCTTTACCGACT
CCCTATCAGCCCCCCTGTTAGTTCTCACAACATGACTACTGCCCTTAATACTTATAGCTAGTC
AATTTCACCTGTCAAAAGAGACCCTGACCCGGAAAAAATTATATATCACAATACTAATTCTC
CTGCAACTATTCTTAATTATAACATTTTCTGCTACTGAGTTAATCATATTCTATATTTTATTTG
AAGCTACCCTGGTACCGACTTTAGTTATCATTACTCGATGAGGAAACCAAACAGAACGACTA
AACGCGGGCCTCTATTTTTTATTCTACACCTTAGTAGGATCCTTGCCCTTACTAGTTGCACTG
TTATATGCCCAAAACAGCTTAGGATCACTAAATTTCTCATAGTACAGTACTGAACTCAGCC
CTTGCCAAATTCCTGATCTAACATTTTCATGTGGCTGGCATGTATAATAGCATTCATAGTAAA
AATACCGCTATATGGCTTACACCTATGACTACCTAAAGCACATGTAGAAGCTCCTATTGCAG
GATCCATAGTACTTGCTGCAGTACTTCTAAAACTAGGGGGATACGGTATAATGCGGATTACA
ACAATACTCAACCCTCTAACAAATTTTATAGCCTACCCCTTCATAATGCTCTCACTATGAGGA
ATAATCATAACAAGTTCTATTTGTTTACGCCAGACAGACCTAAAGTCCCTAATTGCATACTCC
TCTGTTAGTCACATAGCCCTAGTCATTGTAGCAATTCTAATCCAATCCCCATGAAGCTACATA
GGAGCTACTGCTCTAATAATCGCCCATGGGTTAACATCTTCTATGCTATTCTGTCTTGCAAAC
TCTAACTACGAGCGAACCCATAGCCGAACTATAATTCTCGCACGGGGCTTGCAAACGCTTCT
TCCACTTATAGCAGCTTGATGACTATTAGCAGGCCTAACTAACTTAGCTCTTCCTCCTACAAT
TAATCTGATTGGGGAACTGTTTGTAGTGATAGCCACATTTTCATGATCCAATATTACCATTCT
CTTAATAGGAGTTAACATTACCATTACTGCTCTCTACTCTCTATATATACTAATTTCTACACA
GCGAGGCAAGTATACAGACCACATCAAAAATATTAAGCCATCTTATACACGAGAAAATGCC
TTAATAGCCTTACATTTACTACCCCTTTTACTTCTATCTCTTAACCCTAAAGTAGTTCTAGGCT
TTATTTACTGTAAATATAGTTTAACAAAAACATTAGATTGTGAATCTAGTAATAAAAGCTTA
AATCTTTTTATTTACCGAAAAAGCATGCAAGAACTGCTAATTCATGCACCCATGCATAAAAT
CATGGCTTTTTCAACTTTTAGGGGATAGAAGTAATCCATTGGTCTTAGGAGCCAAAAAATTG
GTGCAACTCCAAATAAAAGTAATTAACCTACTTACCTCCTTCACCATTACAACCCTATTTATA
TTAATCATGCCCATTATCCTAACCTGTACCCCAGCATATGAAAATAAACTTTACCCGCAATAT
GTAAAAACCACAGTCTCTTATGCCTTCACAATTAGCATAATTCCCATAGTAATATTCATCCAC
TCCGGACAAGAAGCAATCATCTCAAACTGACACTGAATAACCATCCAAACCCTAAAACTCA
GTCTAAGCTTCAAATTAGATTACTTTTCAATAATCTTCGTACCAGTGGCCCTATTCGTCACAT
GGTCAATTATAGAATTCTCTATATGATATATACACGCAGATCCTAACATTAACCGATTCTTTA
AATATCTACTTATATTCCTCGTCACGATAATAATTCTAGTAACCGCAAATAATCTATTTCAGC
TATTTATCGGCTGAGAGGGAGTAGGAATCATATCATTTCTCCTCATTGGATGATGATATGGA
CGAACAGATGCTAATACGGCTGCCCTACAAGCAGTACTATACAACCGCATTGGAGATGTTGG

TTTTATCATGGCCATAGCTTGATTCTTACTCAATACAAATGCATGGGACTTCCAACAAATCTT
TATAATCGAGCATAAAAACCTCAATATTCCACTGGCAGGACTACTTCTAGCAGCTACCGGAA
AGTCTGCCCAATTTGGACTTCACCCATGACTCCCATCAGCCATAGAAGGCCCCACTCCTGTCT
CAGCACTACTTCACTCTAGCACAATAGTAGTAGCAGGCGTATTTCTTCTAGTCCGATTTCACC
CCTTAATAGAACATAACAAGACAATTCAAACGGTTACCCTATGCTTAGGGGCAATAACAACC
TTGTTTACAGCAATCTGCGCCCTTACCCAAAATGATATCAAAAAAATTATTGCCTTCTCCACC
TCGAGCCAACTAGGACTAATAATTGTAACAATTGGTATTAATCAGCCTCATCTGGCCTTTCTC
CATATCTGCACGCACGCATTCTTCAAGGCCGTGCTATTTCTATGCTCCGGATCTATTATTCAT
AGTCTAAACAACGAACAAGATATTCGAAAGATAGGAGGGCTATTTAAAAGTCTACCATTTA
CCACTACTGCTCTAATCATCGGAACTCTAGCACTTACAGGAATGCCCTTCTTAACAGGATTTT
ACTCCAAAGACTTAATTATTGAAACCGCCAATACGTCATACACCAACGCCTGAGCCTTACTA
ACAACCCTTATCGCCACATCCATAACTGCCGCCTACAGTACTCGAATTGTATTTTTTACACTT
CTAGGACAACCTCGCTATAGTCCCATTATCACCATTAACGAGAATAACCAGTTCCTGACTAA
CTCTGTCAAACGATTGCTACTAGGAAGTATCTTTGCGGGATTCCTAATCTCTCATAACATTAC
ACCCACCACTGTCCCACAAATAACCATGCCCTGTCACCTAAAAATGATAGCCCTTACAGTAA
CAATCCTAGGCTTTATTCTAGCACTAGAACTTAACCTAGCCATACAAAATCTCAAATTTGACT
ACCCTTTAAGCACGTTCAAATTTTCAAATCAACTAGGATATTTCCCTACTACTCTGCATCGTC
TCGCACCAATAATAGGTCTATCAATAAGCCAAAAAACAGCATCCATACTGCTAGATGCAATC
TGACTAGAGAATGTTCTACCAAAGTCCATCTCCTACTTTCAAATAAAATCCTCAATCACAGT
CTCCAACCAAAAGGGACTAATTAAACTATACTTTCTCTCCTTTATAATTACACTAGTCTTAAG
TCTCTTTATACTTAATTTCCACGAGTGACTTCCATAATTACCAATACACCAATAAGGAGAGAT
CATCCAGTAACAATAACTAATCAAGTACCATAACTATATAAAGCTGCAATTCCCATAGCCTC
TTCACTAAAAAATCCTGAATCCCCTGTGTCATAAATCACCCAATCTCCTATTCCATTAAACTT
AAATACTACCTCGGCTTCATCATCCTTTAAAATATAACAAACAGCCAGTAACTCAGACAACA
GACCTACAATAAATGCACCTAGTACAGCCTTATTAGAAAGTCAAACCTCAGGATATTGCTCA
GTGGCTATGGCCGTAGTGTACCCGAACACTACAAGTATACCCCCTAAATAAATTAAAAAGAC
CATTAAACCCAAAAAAGATCCTCCAAAGCTTAACACAATACCACACCCTACACCCCCACTAA
CAATCAAGACTAAACCTCCGTAGATAGGAGAGGGCTTAGAAGAAAACCCCGCAAAACTAAC
TACAAAAACGATACTTAAAATAGATACAATGTATGTTATCATCATTCTTACATGGAATCTAA
CCATGACCAATGACATGAAAAATCATCGTTGTATTTCAACTATAAGAACCCCAATGATCAAC
ATCCGAAAAACTCATCCATTAGTTAAAATTATCAACAACTCATTCATTGACCTTCCAACACC
ATCAAACATTTCAACATGATGAAACTTTGGGTCCCTGTTAGGAGTGTGTCTGATCTTGCAAA
TCTTAACAGGCTTATTTCTAGCCATACACTATACATCAGATACAGCTACAGCCTTTTCATCAG
TCGCACACATTTGTCGAGACGTCAACTATGGTTGATTTATCCGATATATACATGCCAATGGG
GCCTCTATATTTTTTATCTGCCTATTTATACACGTAGGGCGAGGCTTATACTATGGATCATAC
CTATTTCCAGAGACATGGAATATCGGAATTATTCTCCTACTTACAGTTATAGCCACAGCATTC
ATAGGGTATGTACTACCTTGAGGACAAATATCCTTCTGAGGAGCAACCGTCATTACTAACCT
ACTATCAGCAATTCCTTACATTGGCACTAATCTAGTGGAGTGAGTCTGAGGGGGTTTCTCCG
TAGATAAAGCAACACTAACCCGATTTTTTGCTTTTCACTTTATCCTTCCATTTATCATCTCAGC
ACTAGCAATAGTCCATCTATTATTCCTTCACGAAACAGGATCTAATAACCCCTCCGGAATTC
CATCTGACCCAGACAAAATCCCATTTTACCCCTATCATACAATTAAAGACATCCTAGGCGTC
CTATTTCTTGTCCTCGCCTTAATAACCCTGGCTTTATTCTCACCAGACCTGTTAGGAGACCCT
GATAACTATACCCCTGCAAATCCACTAAGTACCCCGCCACATATTAAGCCTGAATGGTACTT
TCTATTTGCCTACGCTATCCTGCGATCTATTCCTAATAAACTAGGAGGGGTGCTAGCTCTAAT
CTTCTCTATTCTAATTCTAACTATTATTCCACTATTACATACATCCAAACAACGAAGCATGAT
ATTCCGACCTCTAAGTCAATGCTTATTCTGACTCCTAGTAGCAGACCTACTCACACTAACATG
AATTGGAGGACAGCCAGTAGAACACCCCTTCATTATTATTGGGCAATTGGCCTCTATTCTCT
ACTTTACAATTCTTCTAGTACTTATACCTATCACTAGCATTATTGAGAATAGCCTCTCAAAAT
GAAGAGTCTTTGTAGTATATTAATTACTTTGGTCTTGTAAACCAAAAACGGAGATTCCTCTAC
TTCTCCCTAAGACTCAAGGAAGGAGCAACAACCCCACTACCAGCACCCAAAGCTGGTGTTCT
ACTTAAACTATCCCCTG

>SRR6109216 (circular)
ATACTATAAATCCACCTCTCATTTTATTCACTTCATACATGTTATTACACACTCTGTGCCATC
ATAGTATGTTTTCATACATCCTCCCTTCTTTCACACCCTATGTATGTCGTACATTAATGGTGTA
CCCCCCCTCCCCCCTATGTATATCGTGCATTAATGGCGTGCCCCATGCATATAAGCATGTACA
TACTGTGCTTGGCTTTACATGAGGATACTCATTACAAGAACTTATTTCAAGCGATAGTCTATG
AGCATGTATTTCACTTAGTCCAAGAGCTTGATCACCAAGCCTCGAGAAACCAGCAATCCTTG
CGAGTACGTGTACCTCTTCTCGCTCCGGGCCCATAATTTGTGGGGGTTTCTATACTGAAACTA
TACCTGGCATCTGGTTCTTACTTCAGGGCCATGTTAGCGTCAACTCAATCCTACTAACCCTTC
AAATGGGACATCTCGATGGACTAATGACTAATCAGCCCATGATCACACATAACTGTGGTGTC
ATGCATTTGGTATTTTTTAATTTTTAGGGGGGGAACTTGCTATGACTCAGCTATGACCGTAAA
GGTCTCGTCGCAGTCAAATCAGCTGTAGCTGGGCTTATTCATCTTTCGAGGCTCCTCATAGAC
ACCCATAAGGTGCAATTCAGTCAATGGTCACAGGACATAACACTATAGATCACCCGGACTG
GCGTTACGTGTACGTACGTGTACGTACGTGTACGTACGTGTACGTACGTGTACGTACGTGTA
CGCACGTGTACGTACGTGTACGCACGTGTACGTACGTGTACGCACGTGTACGTACGTGTACG
TACGTGTACGTACGTGTACGCACGTGTACGTACGTGTACGCACGTGTACGTACGTGTACGTA
CGTGTACGCACGTGTACGCACGTGTACGCACGTGTACGCACGTGTACGCACGTGTACGCACG
TGTACGCACGTGTACGTACGTGTACGTACGTGTACGTACGTGTACGCGTACGTATTTTAGAT
ACTAAGTTAGCTTAGACAAACCCCCCTTACCCCCCGTAACTTCAAGAAGCTTACATATACTT
ATGGATGTCCTGCCAAACCCCAAAAACAAGACTAAATATATGCGCAAACATGAAGTCACTT
ACACCTAAACCCATATAATTAAGCTAACCCCCCAGCCAATGTTGCAACAACTACGGACATGG
GACTCTAAATTTTAATTTATCTATAGATATTTTTCTTTTACTGTGTCTCCCCAGCATTGATTTT
TTAATTATCATTATTCCACACCACCAATTTCCATTGAGCTATTTCACATGAGTTCCAAATCAA
TTATGTTCATGTAGCTTAACGAATAAAGCAAGGTACTGAAAATGCCTAGATGGGTCACGCTA
CCCCATAGACATAAAGGTTTGGTCCTAGCCTTCCTATTAGCCATTAACAAGATTACACATGT
AAGTCTCCACGCTCCAGTGAAAATGCCCCTTAAGTCCTCTTAGACGACCTAAAGGAGCGGGT
ATCAAGCACACCTTATGGTAGCTCACAACGCCTTGCTTAGCCACACCCCCACGGGAAACAGC
AGTGATAAAAATTAAGCTATGAACGAAAGTTCGACTAAGCTATGTTAATACTAGGGTTGGTA
AATCTCGTGCCAGCCACCGCGGTCATACGATTAACTCGAGTTAATAGGCCTACGGCGTAAAG
CGTGTAAAAGAAAAAATCTCCTCTACTAAAGTTAAAGTATGATTAAGCTGTAAAAAGCTACC
ATTAATACTAAAATAAACTACGAAAGTGACTTTAAAATTTCTGATTACACGATAGCTAGGGC
CCAAACTGGGATTAGATACCCCACTATGCCTAGCTCTAAACATAGATATTTTACTAAACAAA
ACTATTCGCCAGAGAACTACTAGCAACAGCTTAAAACTCAAAGGACTTGGCGGTGCTTTATA
TCCCCCTAGAGGAGCCTGTTCTGTAATCGATAAACCCCGATAGACCTCACCATCCCTTGCTA
ATTCAGTTTATATACCGCCATCTTCAGCAAACCCTTAAAAGGAAAAAAAGTAAGCATAACTA
CCCTACATAAAAAAGTTAGGTCAAGGTGTAACCTATGGGCTGGGAAGAAATGGGCTACATT
TTCTATTCAAGAACAACTTCTACGAAAACTTTTATGAAACTAAAAGCTAAAGGCGGATTTAG
TAGTAAATTAAGAATAGAGAGCTTAATTGAACAGGGCAATGAAGCACGCACACACCGCCCG
TCACCCTCCTCGAGTGATATAATTTAATTATAACCTATTTAAACTAAGCAAAGCATAAGAGG
AGACAAGTCGTAACAAGGTAAGCATACTGGAAAGTGTGCTTGGATGAGCCAAAGTGTAGCT
TAAACAAAGCGTCTGGCTTACATCCAGAAGATTTCATTAATATATGACTACTTTGAACCCAA
AGCTAGCCCAAGCAACAATGACTAGTAAAACCATTATGAAACATTCAAACAAAACATTTAG
TAGCATGACTAGAGTATAGGAGATAGAAATTTTTAACTGGAGCTATAGAGAGAGTACCGCA
AGGGAATGATGAAAGATTACCTAAAGTGATAAACAGCAAAGATTGCCCCTTCTACCTTTTGT
ATAATGAGTTAGCTAGAAATAACTTAACAAAGAGAACTTAAGCTAAGTCCCCCGAAACCAG
ACGAGCTACCTATGAACAATCCACTGGGATGAACTCATCTATGTTGCAAAATAGTGAGAAG
ATCCATAGGTAGAGGTGAAAGGCCTAACGAGCCTGGTGATAGCTGGTTGCCCAGAATAGAA
TTTTAGTTCGACTTTAAACCTGCCTACAAAACTAATAATTCTAATGCAGATTTAAAATATATT
CTAAAAAGGTACAGCTTTTTAGAGTTAAGGATACAGCCTTACTTAGAGAGTAAATATTTATA
TAAGCCATAGTAGGCCTAGAGGCAGCCATCAATTAAGAAAGCGTTAAAGCTCAACATCTCT
ATTAACTTAATACCAAGAATATTTAATCAACTCCTAATGTATTACTGGGCCAATCTATTTAAA
TATAGAAGTGATAATGCTAATATGAGTAACAAGAAATATTTCTCCCATGCATAAGCTTATAA

CAGCAACGGATAACCACTGATAGTTAACAACAACATAGAAATAACCTAATGATAAAACACC
TATTAAATCAATTGTTAGTCCAACACAGGCATGCAATCAGGGAAAGATTAAAAGAAGTGAA
AGGAACTCGGCAAACACAAACCCCGCCTGTTTACCAAAAACATCACCTCCAGCATTTCCAGT
ATTGGAGGCACTGCCTGCCCGGTGACATCAGTTAAACGGCCGCGGTATTCTGACCGTGCAAA
GGTAGCATAATCATTTGTTCTCTAAATAAGGACTTGTATGAACGGCCACACGAGGGTTTAAC
TGTCTCTTACTTCCAATCAGTGAAATTGACCTCCCCGTGAAGAGGCGGGGATAAGACAATAA
GACGAGAAGACCCTATGGAGCTTTAATTAACTAATTCAAAAAGAAACTACTAACGACCCAA
CAGGAATAATATATCTCTTTTATGAATTAGCAATTTAGGTTGGGGCGACCTCGGAGGACAAA
ATAGCCTCCGAGTGATTATAAATCTAGACTTACCAGTCAAAATGCTTAATCACTTATTGATCC
AAAAATTCTTTTGATCAACGGAACAAGTTACCCTAGGGATAACAGCGCAATCCTATCCGAGA
GTCCATATCGACAATAGGGTTTACGACCTCGATGTTGGATCAGGACATCCTAATGGTGCAGC
AGCTATTAAAGGTTCGTTTGTTCAACGATTAAAGTCCTACGTGATCTGAGTTCAGACCGGAG
CAATCCAGGTCGGTTTCTATCTATTCAAATAATTTCTCCCAGTACGAAAGGACAAGAGAAAT
AAGGCCTACTTCTCTGAAGCGCCTTAAGACCAATAGATGAATTTATCTAAATCTAGTAAATC
TAACTCCAATATTGCCCAAGAGACAGGGCTTTGTTAGGGTGGCAGAGCCCGGTAATTGTGCA
AAACTTAAACTCTTGTGTCCAGAGGTTCAATTCCTCTCCCTAGCATATGTTTATAATTAACAT
CTTCTCACTAATTGTACCTATTCTTCTTGCTGTAGCCTTTCTGACTCTAGTAGAACGAAAAGT
ACTAGGCTACATACAGCTTCGAAAAGGCCCAAATATTGTAGGTCCATATGGTCTCCTACAAC
CAATCGCAGATGCCGTAAAACTCTTTACTAAAGAACCTTTACGACCACTTACGTCATCCATA
ACTATATTTATTATAGCCCCTATTCTAGCCTTAACACTAGCTCTGACCATGTGAGTCCCCTTA
CCAATACCGCACCCCCTTATTAACATAAACCTAGGAGTACTATTCATACTAGCTATATCAAG
TCTAGCTGTATACTCTATTCTCTGATCTGGATGAGCCTCAAACTCAAAATATGCCCTAATTGG
AGCCCTACGAGCCGTAGCTCAAACGATCTCATATGAAGTCACACTAGCCATTATCCTTTTAT
CCGTTCTACTAATAAATGGCTCATTTACGCTATCCACGCTAATCATTACCCAAGAACGTCTTT
GATTAATCTTCCCTGCATGACCACTAGCTATAATATGATTTATTTCTACCCTAGCAGAAACAA
ATCGAGCCCCCTTTGACCTAACAGAAGGAGAATCAGAACTAGTCTCTGGGTTTAATGTCGAA
TATGCAGCAGGTCCATTTGCCCTGTTCTTCCTAGCAGAATATGCCAACATCATCATAATAAAT
GCTCTTACAACTATTCTATTCTTCGGAGCATTTCACAGCCCCTACATACCAGAATTATATATT
GTTAATTTTACCGTAAAAACTTTACTCCTCACAATCTCTTTTCTATGAATCCGAGCATCATAT
CCTCGATTCCGATATGACCAACTAATACACCTACTATGAAAAAACTTTCTACCTCTCACACTA
GCTCTATGTATATGACATGTAACTCTACCTATTATTACAGCAAGCATTCCACCTCAAACGTAA
GAAATATGTCTGACAAAAGAATTACTTTGATAGAGTAAGCCATAGGGGTTTAAGTCCCCTTA
TTTCTAGAATCATAGGAATCGAACCTAATCTTAAGAACTCAAAAATCTTCGTGCTACCTAAC
TACACCATATCCTAAAGTAAGGTCAGCTAAATAAGCTATCGGGCCCATACCCCGAAAATGTT
GGTTTATCCCCTTCCCATACTAATAAAACCAGCCATCCTTATTACCATTACATCCACCGTTGT
CTTAGGAACTATAATTGTTCTATTTAGCTCCCACTGATTCATAATCTGAGTTGGCTTTGAAAT
AAATATACTAGCCATTATCCCAATTCTAATAAAAAAATATAACCCACGAGCCATAGAAGCTT
CCACAAAATATTTTCTAACACAAGCTACAGCATCCATGCTTCTAATGCTAAGCATCATCATC
AATCTACTATGCTCAGGACACTGGACAGTTTCAACAATTCCCAATCCAGTAGCATCTACCAT
AATTACTATTGCTCTAACAATAAAACTTGGCCTATCACCATTTCACTTTTGAGTCCCAGAAGT
TACACAAGGCATTTCCTTATCTTCAGGAATAATCCTATTAACATGACAAAAAATTGCACCTTT
ATCCATTCTATACCAAATTTCACCATCCGTTAACTCAAATCTCTTACTAATAATAGCTATCAC
ATCCATACTGGTAGGAGGCTGAGGAGGATTAAATCAAACCCAACTCCGAAAAATCTTGGCA
TACTCCTCAATTACCCATATAGGCTGAATGGCTGCTATTATAGTATATAATCCTACCCTAGCA
ATTCTTAACCTAACAATTTACATCATAATAACTCTAGGAACATTTATACTATTCATACATAGC
TCATCTACAACAACACTATCACTATCTTATACATGAAATAAATTCCCATTAATAGCCCCACTA
ATCTTAATACTTATACTATCACTAGGAGGCCTCCCTCCTCTCTCAGGCTTTATCCCCAAATGA
ATAATTATTCAAGAATTAACAAAAAACGACATAATTATTATACCTACACTTATAGCCATTAC
AGCCCTACTAAACCTATATTTTTACACACGCCTAACATATACTACAGCACTAACTATATTTCC
CGCAACAAATAATATAAAAATAAAATGACAATTCGAAACCACAAAAAAGACAACTCTCCTA
GCCCCTCTAATCGTGACATCAACTATACTACTACCACTAACCCCAATACTAGCAGCTCTTGAT

TAGAGATTTAGGCTAAACAGACCAAGGGCCTTCAAAGCCTCAAGTAAGTGAAAATCCACTT
AATCTCTGCAATATTTAAGGACTGCAAGAATCTATCTCACATCAACTGAACGCAAAACAATC
GCTTTAATTAAGCTAAGCCCTTCTAGATCGGTGGGCTTTTACCCCACAAAACTTTAGTTAACA
GCTAAAAACCCTAGACAACTGGCTTCAATCTACTTCTCCCGCCGCGTAGGAAAAAAAGGCG
GGAGAAGCCCCGGCAGAATTGAAGCTGCTTCTTTGAATTTGCAATTCAATATGATTACTCAC
CACAGAGCCTGGTAAAAAGAGGACTTAACCCCCATTCTTAGATTTACAGTCTAGTGCTTTTA
TCAGCCATTTTACCTATGTTCATTAACCGATGACTGTTTTCCACCAACCACAAAGATATTGGC
ACTCTTTATCTTCTGTTTGGCGCATGAGCTGGTATAACGGGCACTGCTCTTAGCCTTCTAATC
CGTGCTGAATTAGGTCAGCCTGGAGCTCTGTTAGGAGATGACCAAATTTACAATGTAGTTGT
AACTGCCCACGCATTCGTAATAATCTTCTTTATAGTTATGCCTATTATGATCGGAGGCTTTGG
AAATTGACTAGTACCTTTGATAATTGGTGCGCCTGATATAGCATTTCCCCGAATAAACAATA
TGAGTTTCTGATTACTACCTCCATCCTTTCTTCTCCTTCTGGCTTCTTCCATAGTAGAAGCAGG
CGCAGGGACTGGATGAACTGTATACCCCCCTCTAGCAGGTAACTTAGCCCATGCAGGAGCAT
CAGTAGACCTAACAATCTTTTCTCTACATCTGGCAGGGGTTTCTTCTATTTTAGGGGCTATTA
ACTTTATTACCACTATCATCAACATGAAACCCCCTGCAATATCTCAATATCAAACTCCCCTAT
TCGTGTGATCAGTACTTATCACAGCAGTACTTCTCCTTCTATCTTTACCAGTCTTAGCAGCTG
GAATTACTATACTACTTACAGATCGAAATCTCAATACTACTTTCTTCGACCCGGCCGGAGGA
GGGGATCCCATTCTATATCAACATCTATTTTGATTCTTTGGCCATCCTGAAGTCTATATTCTA
ATCCTCCCTGGGTTTGGAATAATTTCACACATTGTCACATACTATTCAGGAAAGAAAGAGCC
CTTTGGTTATATAGGAATAGTGTGGGCAATAATGTCAATTGGGTTCTTAGGATTTATTGTATG
GGCCCATCATATATTTACTGTAGGCATAGATGTTGATACACGAGCCTATTTTACCTCGGCCAC
TATAATTATTGCAATCCCGACAGGAGTTAAAGTATTCAGCTGACTAGCTACCCTGCATGGAG
GAAATATTAAGTGATCCCCTGCCATAATGTGAGCCCTAGGTTTCATTTTTCTATTTACAGTAG
GGGGTCTTACAGGCATTGTTCTAGCTAACTCATCTCTGGACATTGTCCTCCATGACACATACT
ACGTAGTAGCACACTTCCACTATGTACTATCAATAGGGGCCGTGTTCGCTATTATAGGAGGC
TTCGTCCATTGATTCCCATTATTTTCGGGCTATACACTTAATAATACATGAGCAAAAATTCAC
TTCGTAATTATATTTGTTGGAGTCAATATAACATTCTTCCCCCAGCACTTCCTAGGTCTATCA
GGTATGCCTCGACGATATTCTGATTATCCAGACGCCTATACAGCATGAAACACAGTGTCTTC
AATGGGTTCATTCATTTCACTAACAGCAGTTATGTTAATAATTTTCATAATTTGAGAAGCCTT
CGCATCTAAACGAGAAGTGGCAGTGATTGAGCTTACTACAACTAATATTGAATGGCTCCACG
GATGTCCTCCCCCATACCATACATTCGAAGAGCCCGCCTACGTCATACTGAAATAAGAAAGG
AAGGAATCGAACCCTCTAGAATTGGTTTCAAGCCAACATCATAACCACTATGTCTCTCTCAA
TAAAGAGGTATTAGTAAAAATTACATAACTTTGTCAAAGTTGAATTATAGGTGAAAATCCTT
TATATCTCTATGGCGCATCCCTTTCAAACAGGCCTTCAAGATGCAACTTCCCCCATCATGGAA
GAACTCCTACACTTCCATGATCATACACTAATAATTGTATTCTTAATTAGCTCCTTAGTTCTC
TATATCATTTCAATTATATTAACTACTAAATTAACACATACAAATACAATAGACGCACAAGA
AGTGGAGACAGTATGAACTATTCTACCAGCTATCATCTTAATTATAATTGCACTTCCATCACT
ACGAATTCTTTATATGATAGATGAAATTAATAGCCCATCATTAACTGTAAAGACTATGGGGC
ATCAATGATACTGAAGCTATGAGTATACAGATTATGAAGACTTGAGCTTCGATTCTTATATA
ACCCCAACACAAGAATTAAAGCCCGGAGAGCTGCGATTACTAGAGGTAGATAATCGAGTAG
TACTACCCATAGAAATAACAATTCGCATATTAATTTCCTCAGAAGATGTGCTACATTCGTGA
GCTGTCCCATCTCTAGGACTAAAAACTGATGCAATCCCGGGGCGATTAAATCAAACAACTCT
TATGGCCATGCGACCAGGACTATATTATGGCCAATGCTCAGAGATTTGCGGTTCTAATCATA
GCTTTATACCCATTGTACTTGAAATAGTTCCGCTGTCTTACTTTGAAAAATGATCCGCTTCAA
TACTATAAAATCATTAAGAAGCTTTATAGCGTTAACCTTTTAAGTTAAAGACTGAGAGTGCA
AGTCTCTCCTTAATGGAAATGCCACAATTAGACACATCCACATGATTTATTACAATCCTATCC
ATGATCTTAACACTATTTATTATATTTCAGTTAAAAGTCTCAAAACACAACTATCCTGAGTTT
CCTGAATTAAAAACAACCCCACTATCAAAAAAACCTATGCCTTGAGAAGAAAAATGAACGA
AAATCTGTTCGCCTCTTTTACTACCCCAATGATAATAGGAGTACCAATTGTGGTTCTGATTAT
TATTTTCCCAAGTATCTTATTTCCCTCACCTAATCGATTAATCAATAACCGCCTAGCTACTGT
CCAACAATGATTAATTCAATTAGTATCTAAGCATATACTGTCTATTCACAATTATAAAGGAC

AAACCTGAGCGCTAATACTAATGTCATTAATTTTATTTATTGGCTCAACTAACCTTTTAGGCC
TATTACCACATTCATTCACACCTACTACACAACTATCAATAAATTTAGGAATGGCCATTCCTC
TGTGAGCAGGTACAGTAGTTATAGGATTTCGACACAAAACTAAGGCATCTCTGGCCCACTTT
CTACCCCAAGGAACACCTCTTCCTCTGATCCCAGTGTTAGTAATTATCGAGACAATTAGTCTG
TTTATTCAACCTGTGGCCCTAGCTGTACGACTGACCGCTAATATTACTGCAGGCCATCTACTT
ATTCATTTAATCGGAGGGGCTACCCTAGTTTTAATAAACATTAACCCTGTAACAGCCCTAATT
ACCTTTATTATTCTTATTCTACTTACTATTCTCGAATTAGCTGTAGCTCTTATCCAAGCCTATG
TTTTCACCCTACTAGTTAGTCTATACTTACATGATAATACCTAATGATCCACCAAACACATGC
ATATCATATAGTTAACCCAAGCCCATGGCCACTTACAGGAGCTCTCTCGGCTCTTCTTATAAC
ATCAGGACTAATTATATGATTTCACTTTAACTCGGTATACTTACTAATACTAGGCCTCACCAC
CAACATACTTACCATGTACCAATGATGACGAGATATCGTCCGGGAAAGTACCTTCCAAGGCC
ATCACACCCCAATCGTCCAAAAAGGACTACGATATGGAATAGTCTTATTTATTGTATCAGAA
GTATTTTTCTTCGCGGGATTCTTTTGGGCCTTCTACCACTCAAGCCTAGCACCTACTCCTGAA
TTAGGAGCATGTTGACCACCTACAGGTATCATCCCCTTAAATCCGCTAGAAGTTCCACTACTT
AACACCTCTGTACTTCTAGCATCTGGGGTATCTATCACTTGAGCTCATCATAGTCTAATAGAA
GGAGACCGTAAACATATGCTCCAAGCCCTATTTATTACTATTTCACTAGGAGTTTATTTTACA
CTTCTACAAGCCTCAGAATACTATGAGACATCCTTTACTATTTCAGATGGAGTGTATGGTTCA
ACCTTCTTTATAGCAACTGGATTTCATGGGCTTCATGTAATTATTGGTTCAACTTTTCTTACTG
TGTGTTTTCTACGACAACTGCACTTTCACTTTACATCCAACCATCACTTTGGCTTTGAAGCGG
CAGCATGATACTGACACTTCGTAGACGTTGTATGGCTTTTCCTTTATGTTTCTATTTACTGAT
GAGGATCCTACTTCTTTAGTATTAACTAGTACGATTGACTTCCAATCAATAGGCTCTGGTACA
AGCCAGAAAGAAGTAATAAACCTAATTCTAGCACTTCTAACCAACGTATTATTAGCCTCTCT
GCTCGTTCTTATTGCATTCTGACTGCCACAACTAAATATCTATGCAGAAAAAGTAAGTCCTTA
TGAATGTGGATTTGATCCTATAGGATCAGCACGCCTTCCTTTCTCTATAAAATTTTTTCTAGT
AGCTATTACATTCCTTTTATTCGACTTAGAAATTGCTCTACTCCTCCCGCTTCCTTGAGCATTA
CAGACTAACAACTTAATAACTATGCTTATTATAGCTCTATTACTCATTTCCCTATTAGCCGCA
AGCCTAGCTTATGAGTGAACCGAAAAGGGGCTAGAATGGGCCGAATATGATAATTAGTTTA
AACTAAAATAAGTGATTTCGACTCACTAGATTATGACTAACATCATAATTATCAAATGTCTG
TGGTCTATATTAATCTCTTTCTAGCCTTTACTATATCCTTAATAGGGCTACTCATCTACCGATC
CCATTTAATATCCTCCCTATTATGCTTAGAAGGCATGATGCTGTCACTATTTGTTATACTAAC
AGTAACAATCCTAAGTAACCACTTCACACTAGCCAGTATGACTCCAATCATTCTATTAGTATT
CGCTGCCTGTGAAGCAGCGTTGGGGCTTTCTCTGCTAGTAATAGTCTCTAATACCTATGGAA
CTGACTACGTACAAAATCTTAACCTCCTACAATGCTAAAAATTATCCTCCCTACCATAATACT
AGTACCCCTTACATGAATATCAAAGCCTAACATAATCTGAATTAATACAACAACCTATAGTC
TACTCATCAGCCTTATTAGTCTGACATATCTCAATCAACTCAGTGATAATAGTCTAAACTTCT
CATTAACATTCTTTACCGACTCCCTATCAGCCCCCCTGTTAGTTCTCACAACATGACTACTGC
CCTTAATACTTATAGCTAGTCAATTTCACCTGTCAAAAGAGACCCTGACCCGGAAAAAATTA
TATATCACAATACTAATTCTCCTGCAACTATTCTTAATTATAACATTTTCCGCTACTGAGTTA
ATCATATTCTATATTTTATTTGAAGCTACCCTGGTACCGACTTTAGTTATCATTACTCGATGA
GGAAACCAAACAGAACGACTAAACGCGGGCCTCTATTTTTTATTCTACACCTTAGTAGGATC
CTTGCCCTTACTAGTTGCACTGTTATATGCCCAAAACAGCTTAGGATCACTAAATTTTCTCAT
AGTACAGTACTGAACTCAGCCCTTGCCAAATTCCTGATCTAACATTTTCATGTGGCTGGCATG
TATAATAGCATTCATAGTAAAAATACCGCTATATGGCTTACACCTATGACTACCCAAAGCAC
ATGTAGAAGCTCCTATTGCAGGATCCATAGTACTTGCTGCAGTACTTCTAAAACTAGGGGGA
TACGGTATAATGCGGATTACAACAATACTCAACCCTCTAACAAATTTTATAGCCTACCCCTTC
ATAATGCTCTCACTATGAGGAATAATCATAACAAGTTCTATTTGTTTACGCCAGACAGACCT
AAAGTCCCTAATTGCATACTCCTCTGTTAGTCACATAGCCCTAGTCATTGTAGCAATTCTAAT
CCAATCCCCATGAAGCTACATAGGAGCTACTGCTCTAATAATCGCCCATGGGTTAACATCTT
CTATGCTATTCTGTCTTGCAAACTCTAACTACGAGCGAACCCATAGCCGAACTATAATTCTCG
CACGGGGCTTGCAAACGCTTCTTCCACTTATAGCAGCTTGATGACTATTAGCAGGCCTAACT
AACTTAGCTCTTCCTCCTACAATTAATCTGATTGGGGAACTGTTTGTAGTGATAGCCACATTT

TCATGATCCAATATTACCATTCTCTTAATAGGAGTTAACATTACCATTACTGCTCTCTACTCT
CTATATATACTAATTTCTACACAGCGAGGCAAGTATACAGACCACATCAAAAATATTAAGCC
ATCTTATACACGAGAAAATGCCTTAATAGCCTTACATTTACTACCCCTTTTACTTCTATCTCTT
AACCCTAAAGTAGTTCTAGGCTTTATTTACTGTAAATATAGTTTAACAAAAACATTAGATTGT
GAATCTAGTAATAAAAGCTTAAATCTTTTTATTTACCGAAAAAGCATGCAAGAACTGCTAAT
TCATGCACCCATGCATAAAATCATGGCTTTTTCAACTTTTAGGGGATAGAAGTAATCCATTG
GTCTTAGGAGCCAAAAAATTGGTGCAACTCCAAATAAAAGTAATTAACCTACTTACCTCCTT
CACCATTACAACCCTATTTATATTAATCATGCCCATTATCCTAACCTGTACCCCAGCATATGA
AAATAAACTTTACCCGCAATATGTAAAAACCACAGTCTCTTATGCCTTCACAATTAGCATAA
TTCCCATAGTAATATTCATCCACTCCGGACAAGAAGCAATCATCTCAAACTGACACTGAATA
ACCATCCAAACCCTAAAACTCAGTCTAAGCTTCAAATTAGATTACTTTTCAATAATCTTCGTA
CCAGTGGCCCTATTCGTCACATGGTCAATTATAGAATTCTCTATATGATATATACACGCAGAT
CCTAACATTAACCGATTCTTTAAATATCTACTTATATTCCTCGTCACGATAATAATTCTAGTA
ACCGCAAATAATCTATTTCAGCTATTCATCGGCTGAGAGGGAGTAGGAATCATATCATTTCT
CCTCATTGGATGATGATATGGACGAACAGATGCTAATACGGCTGCCCTACAAGCAGTACTAT
ACAACCGCATTGGAGATGTTGGTTTTATCATGGCCATAGCTTGATTCTTACTCAATACAAATG
CATGGGACTTCCAACAAATCTTTATAATCGAACATAAAAACCTCAATATTCCACTGGCAGGA
CTACTTCTAGCAGCTACCGGAAAGTCTGCCCAATTTGGACTTCATCCATGACTCCCATCAGCC
ATAGAAGGCCCCACTCCTGTCTCAGCACTACTTCACTCTAGCACAATAGTAGTAGCAGGCGT
ATTTCTTCTAGTCCGATTTCACCCCTTAATAGAACATAACAAGACAATTCAAACGGTTACCCT
ATGCTTAGGGGCAATAACAACCTTGTTTACAGCAATCTGCGCCCTTACCCAAAATGATATCA
AAAAAATTATTGCCTTCTCCACCTCGAGCCAACTAGGACTAATAATTGTAACAATTGGTATT
AATCAGCCTCATCTGGCCTTTCTCCATATCTGCACGCACGCATTCTTCAAGGCCATGCTATTT
CTATGCTCCGGATCTATTATTCATAGTCTAAACAACGAACAAGATATTCGAAAGATAGGAGG
GCTATTTAAAAGTCTACCATTTACCACTACTGCTCTAATCATCGGAACTCTAGCACTTACAGG
AATGCCCTTCTTAACAGGATTTTACTCCAAAGACTTAATTATTGAAACCGCCAATACGTCAT
ACACCAACGCCTGAGCCTTACTAACAACCCTTATCGCCACATCCATAACTGCCGCCTACAGT
ACTCGAATTGTATTTTTTACACTTCTAGGACAACCTCGCTATAGTCCCATTATCACCATTAAC
GAGAATAACCAGTTCCTGACTAACTCTGTCAAACGATTGCTACTAGGAAGTATCTTTGCGGG
ATTCCTAATCTCTCATAACATTACACCCACCACTGTCCCACAAATAACCATGCCCTGTCACCT
AAAAATGATAGCCCTTACAGTAACAATCCTAGGCTTTATTCTAGCACTAGAACTTAACCTAG
CCATACAAAATCTCAAATTTGACTACCCGTTAAGCACGTTCAAATTTTCAAATCAACTAGGA
TATTTCCCTACTACTCTGCATCGTCTCGCACCAATAATAGGTCTATCAATAAGCCAAAAAAC
AGCATCCATACTGCTAGATGCAATCTGACTAGAGAATGTTCTACCAAAGTCCATCTCCTACT
TTCAAATAAAATCCTCAATCACAGTCTCCAACCAAAAGGGACTAATTAAACTATACTTTCTC
TCCTTTATAATTACACTAATCTTAAGTCTCTTTATACTTAATTTCCACGAGTGACTTCCATAAT
TACCAATACACCAATAAGGAGAGATCATCCAGTAACAATAACTAATCAAGTACCATAACTA
TATAAAGCTGCAATTCCCATAGCCTCTTCACTAAAAAATCCTGAATCCCCTGTATCATAAATC
ACCCAATCTCCTATTCCATTAAACTTAAATACTACCTCGGCTTCATCATCCTTTAAAATATAA
CAAACAGCCAGTAACTCAGACAACAGACCTACAATAAATGCACCTAGTACAGCCTTATTAG
AAAGTCAAACCTCAGGATATTGCTCAGTGGCTATGGCCGTAGTGTACCCGAACACTACAAGT
ATACCCCCTAAATAAATTAAAAAGACCATTAAACCCAAAAAAGATCCTCCAAAGCTTAACA
CAATACCACACCCTACACCCCCACTAACAATCAAGACTAAACCTCCGTAGATAGGAGAGGG
CTTAGAAGAAAACCCCGCAAAACTAACTACAAAAACGATACTTAAAATAGATACAATGTAT
GTTATCATCATTCTTACATGGAATCTAACCATGACCAATGACATGAAAAATCATCGTTGTATT
TCAACTATAAGAACCCCAATGATCAACATCCGAAAAACTCATCCATTAGTTAAAATTGTCAA
CAACTCATTCATTGACCTTCCAACACCATCAAACATTTCAACATGATGGAACTTTGGGTCCCT
GTTAGGAGTGTGTCTGATCTTGCAAATCTTAACAGGCTTATTTCTAGCCATACACTATACATC
AGATACAGCTACAGCCTTTTCATCAGTCGCACACATTTGTCGAGACGTCAACTATGGTTGAT
TTATCCGATATATACATGCCAATGGGGCCTCTATATTTTTTATCTGCCTATTTATACACGTAG
GGCGAGGCTTATACTATGGATCATACCTATTTCCAGAGACATGGAATATCGGAATTATTCTC

CTACTTACAGTTATAGCCACAGCATTCATAGGATATGTACTACCTTGAGGACAAATATCCTT
CTGAGGAGCAACCGTCATTACTAACCTACTATCAGCAATTCCTTACATTGGCACTAATCTAG
TGGAGTGAATCTGAGGGGGTTTCTCCGTAGATAAAGCAACACTAACCCGATTTTTTGCTTTTC
ACTTTATCCTTCCATTTATCATCTCAGCACTAGCAATAGTCCATCTATTATTCCTTCACGAAA
CAGGATCTAATAACCCCTCCGGAATTCCATCTGACCCAGACAAAATCCCATTTTACCCCTAT
CATACAATTAAAGACATCCTAGGCGTCCTATTTCTTGTCCTCGCCTTAATAACTCTGGCTTTA
TTCTCACCAGACCTGTTAGGAGACCCTGATAACTATACCCCTGCAAACCCACTAAGTACCCC
GCCACATATTAAGCCTGAATGGTACTTTCTATTTGCCTACGCTATCCTGCGATCTATCCCTAA
TAAACTAGGAGGGGTGCTAGCTCTAATCTTCTCTATTCTAATTCTAACTATTATTCCACTATT
ACATACATCCAAACAACGAAGCATGATATTCCGACCTCTAAGTCAATGCTTATTCTGACTCC
TAGTAGCAGACCTACTCACACTAACATGAATTGGAGGACAGCCAGTAGAACACCCCTTCATT
ATTATTGGGCAATTGGCCTCTATTCTCTACTTTACAATTCTTCTAGTACTTATACCTATCACTA
GCATTATTGAGAATAGCCTCTCAAAATGAAGAGTCTTTGTAGTATATTAATTACTTTGGTCTT
GTAAACCAAAAACGGAGATTCCTCTACTTCTCCCTAAGACTCAAGGAAGGAGCAACAACCC
CACTACCAGCACCCAAAGCTGGTGTTCTACTTAAACTATCCCCTG
